# Efficient Dehydration of 1-Phenylethanol to Styrene by Copper(II) and Zinc(II) Fused-Bisoxazolidine Catalysts

**DOI:** 10.1021/acscatal.4c04572

**Published:** 2024-10-11

**Authors:** Aurodeep Panda, Caroline R. Wood, William W. Brennessel, William D. Jones

**Affiliations:** Department of Chemistry, University of Rochester, Rochester, New York 14627, United States

**Keywords:** copper, dehydration, alcohols, styrene, olefins

## Abstract

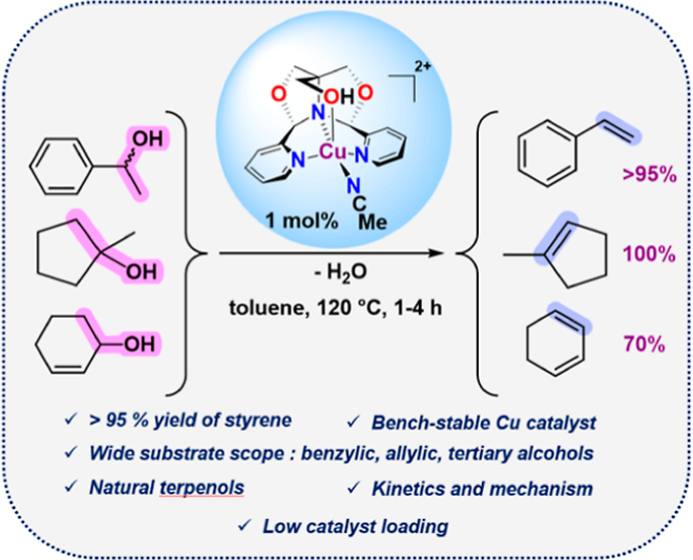

The air-stable copper(II) catalyst [Cu(^meso^FOX-L1)(MeCN)][OTf]_2_ (**2a**) is prepared from a fused meso-dipyridylbisoxazolidine (^meso^FOX-L1) and CuBr_2_, followed by treatment with AgOTf. The compound is a catalyst for the dehydration of 1-phenylethanol to styrene (+H_2_O) in over 95% yield at 1 mol % catalyst loading. Other alcohols (benzylic, tertiary, or allylic) are also efficiently dehydrated by this catalyst. Water separation in toluene solvent is found to play an important role in the reaction. Related compounds with Zn(II) have been prepared and display reduced dehydration activity.

## Introduction

Dehydration of biomass-derived alcohols represents a valuable route to access various important olefins. This reaction appears widely in the literature and typically involves one of the following methods: (a) neat thermolysis at high temperature,^1^ (b) catalytic dehydration by heterogeneous metal oxides such as silica or alumina,^2^ (c) catalytic dehydration by zeolites,^3,4^ (d) catalytic dehydration by a Brønsted acid,^5,6^ or (e) use of homogeneous metal complexes.^7−9^ Though there are several reports of heterogeneous catalytic dehydration, they generally suffer from the lack of product selectivity due to the elevated temperatures used in the processes. Thus, dehydration of alcohols at lower temperatures provides a novel pathway for obtaining industrially relevant olefins with less energy consumption and lower carbon footprint.^7^

One of the industrially and synthetically most important olefins is styrene, which is currently produced mainly via the dehydrogenation of ethylbenzene, and to a lesser extent (∼15%), dehydration of 1-phenylethanol.^10,11^ The reactive vinyl moiety of styrene makes it prone to polymerization, and competitive formation of α-methylbenzyl ethers (AMEs) via nucleophilic substitution reactions are often observed. The development of catalysts for selective alcohol dehydration could lead to a new route to olefin formation.

In 2010, Klein-Gebbink and co-workers demonstrated that suspended Re_2_O_7_ gave highly selective conversion of 1-phenylethanol to styrene in toluene at 100 °C in the absence of Brønsted acid.^12^ Espenson and Zhu showed that methyltrioxorhenium catalyzes dehydration of various alcohols to give the corresponding ethers in moderate to good yields.^13^ With the impetus to use nonprecious metal based catalysts, Laali et al. found that 10 mol % Cu(OTf)_2_ dehydrates various primary, secondary and tertiary alcohols at <160 °C to their corresponding olefins in yields ranging from 30 to 92%,^14^ and Gröger recently used Cu(OTf)_2_ to dehydrate primary alcohols in 73% yield at 150–180 °C.^15^ Hoffmann and co-workers demonstrated the use of stoichiometric anhydrous CuSO_4_ in dehydrating neat alcohols to their corresponding olefins (120–160 °C) with styrene being produced in 65% yield (at 120 °C).^16^

In 2021, our group showed that Fe(^meso^FOX-L1)(OTf)_2_ can be used to catalyze the dehydration of 1-phenylethanol to styrene in up to 74% yield at moderate temperatures (^meso^FOX-L1 = fused meso-dipyridylbisoxazolidine).^17^ Also seen in the reaction were the meso- and rac-AMEs, as well as small amounts of a styrene dimer at higher conversions (eq 1). The success of this ^meso^FOX supported catalyst led us to investigate the reactivity of other first-row metal complexes. In this finding, we report a novel copper-FOX catalyst that dehydrates 1-phenylethanol to styrene in high yield and correlate metal–ligand electronic effects in the dehydration reaction. Several similar derivatives were prepared with zinc(II) and were examined for comparison to copper(II).
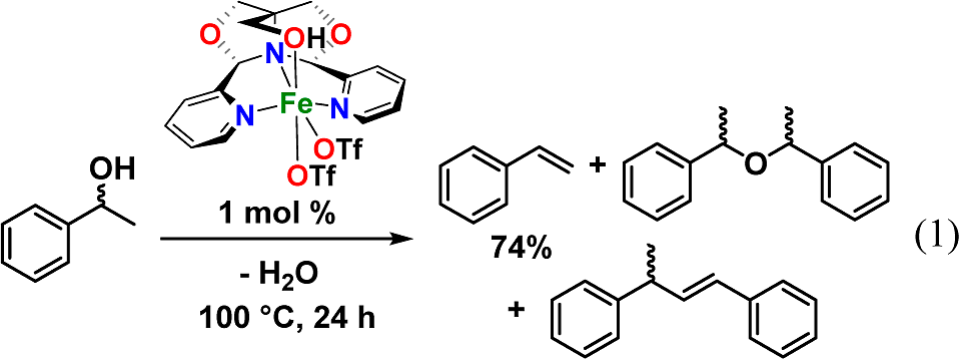
1

## Results and Discussion

### Preparation of Copper(II) Complexes

**Scheme 1:**
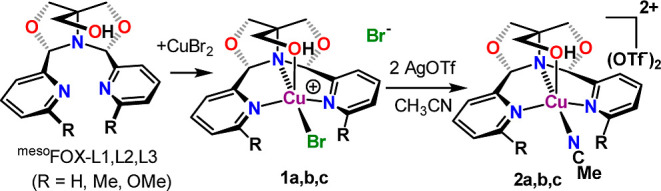
Synthesis of Copper(II) Complexes

**Figure 1:**
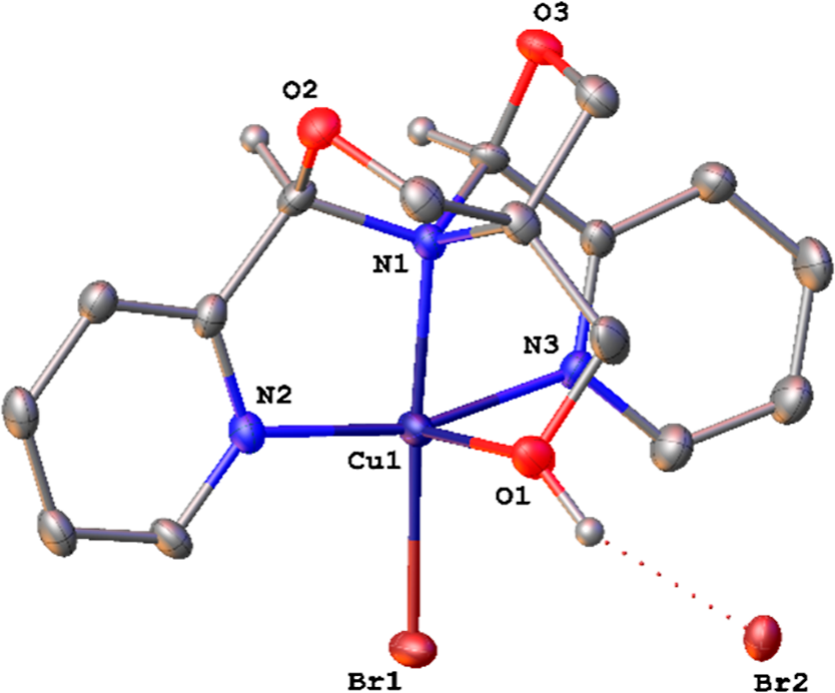
X-ray structure of **1a**. Selected distances (Å) and angles (deg): Cu1–N1, 2.060(5); Cu1–N2, 1.986(5); Cu1–N3, 2.004(5); Cu1–O1, 2.399(4); Cu1–Br1, 2.4023(10); N2–Cu1–N3, 163.5(2); N1–Cu1–Br1, 176.60(14). Ellipsoids are shown at the 30% probability level.

Scheme 1 shows the synthetic sequence for the preparation of the copper compounds presented in this study. Reaction of the ^meso^FOX-L1 ligand with one equiv of CuBr_2_ in acetonitrile leads to the formation of (^meso^FOX-L1)CuBr_2_, **1a**. A single crystal X-ray structure of this molecule showed it to be a cation, with one bromide bound to the copper(II) and a second bromide hydrogen bonded to the hydroxymethyl OH group (Figure 1). The structure is best described as a square pyramid with the OH group in the apical position. The trans pyridines appear at an angle of 163.5(2)°. The copper–oxygen bond to the hydroxyl group is very long [2.399(4) Å], indicative of a weak interaction.

**Figure 2:**
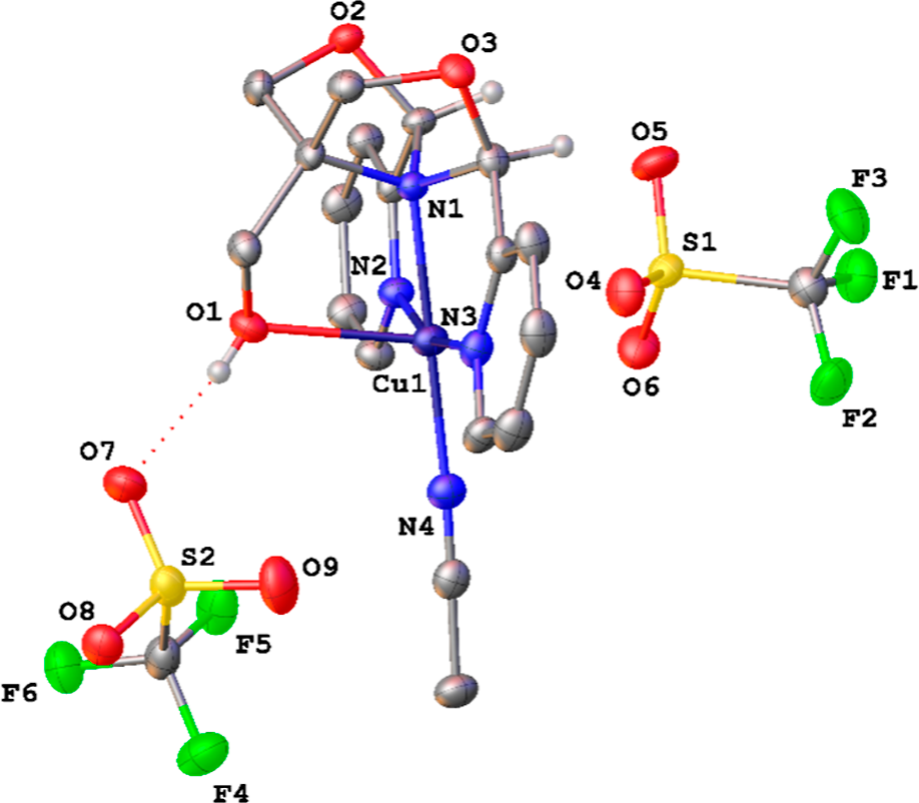
X-ray structure of [Cu(^meso^FOX-L1)(MeCN)][OTf]_2_ (**2a**). Selected distances (Å) and angles (deg): Cu1–N1, 2.036(2); Cu1–N2, 1.982(2); Cu1–N3, 1.994(2); Cu1–O1, 2.377(2); Cu1–N4, 1.977(2); N2–Cu1–N3, 164.44(10); N1–Cu1–N4, 178.48(9). Ellipsoids are shown at the 30% probability level.

Treatment of **1a** with two equiv of silver triflate in acetonitrile leads to the precipitation of AgBr and the formation of the bis-triflate salt, **2a**. The single crystal X-ray structure of **2a** shows that the two triflates are outer sphere, and that the dication is solvated by one acetonitrile (Figure 2). The structure is also described as a square pyramid with an apical OH group, which is also weakly bound to the copper [2.377(2) Å] with slightly bent *trans*-pyridines [164.44(10)°]. **2a** is paramagnetic, and the magnetic moment was determined to be 2.23 B.M. using the Evans method with a trifluorotoluene capillary as a standard in the ^19^F nuclear magnetic resonance (NMR) spectrum (see Supporting Information Figure S30). This value is consistent with a *d*^9^ Cu(II) formulation. The related derivatives with methyl (**1b**, **2b**) and methoxy (**1c**, **2c**) substituted FOX ligands were prepared similarly.

### Dehydration with Copper-FOX

**Scheme 2:**
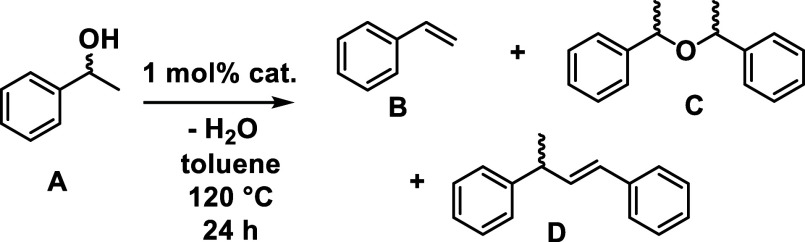
Reaction Conditions for Dehydration of 1-Phenylethanol

The Cu(II) bis-triflate complex **2a** was found to be an efficient catalyst for the dehydration of 1-phenylethanol (**A**) to give styrene (**B**). Initial experiments were conducted at 120 °C for 24 h at high alcohol concentrations, as done previously with iron, and are summarized in Table 1. At a substrate concentration of 4.5 M in toluene, 22% of styrene was obtained with the product profile being dominated by rac- and meso-AMEs (**C**) and styrene dimer (**D**) (Scheme 2). At lower alcohol concentrations, the product mixture shifted to more styrene and less AME. When the substrate loading was decreased to 2.0 M, 78% of **B** was obtained. Finally, at a substrate concentration of 0.83 M **A** in toluene, an 85% yield of **B** was obtained after 24 h. To further demonstrate that the catalysis was by the Cu-based FOX catalyst, a control reaction was performed with 1 mol % of Cu(OTf)_2_ under similar conditions. Only trace amounts of **B** and unreacted **A** were observed.^18^ A similar reaction with Cu(OTf)_2_ and bipy did produce a 31% yield of styrene.^19^ Furthermore, water inhibits the reaction. Heating a solution of 1-phenylethanol (0.83 M) in water with 1 mol % **2a** showed formation of only traces of styrene [see gas chromatography (GC) chromatograms in Figures S1–S6 in the Supporting Information].

**Table 1:** Dehydration of 1-Phenylethanol Using **2a**^a^

[PhEtOH], M	conversion %	% styrene	% ethers	% styrene dimer^b^
4.5	95	22	26	1.3
2.0	100	78	<1	9.7
0.83	100	85	<1	10.5

a Reactions carried out at 120 °C in toluene with 1 mol % **2a**, 24 h.

b About 9% acetophenone is seen in these reactions; about 3% acetophenone was present in the starting material.

**Figure 3:**
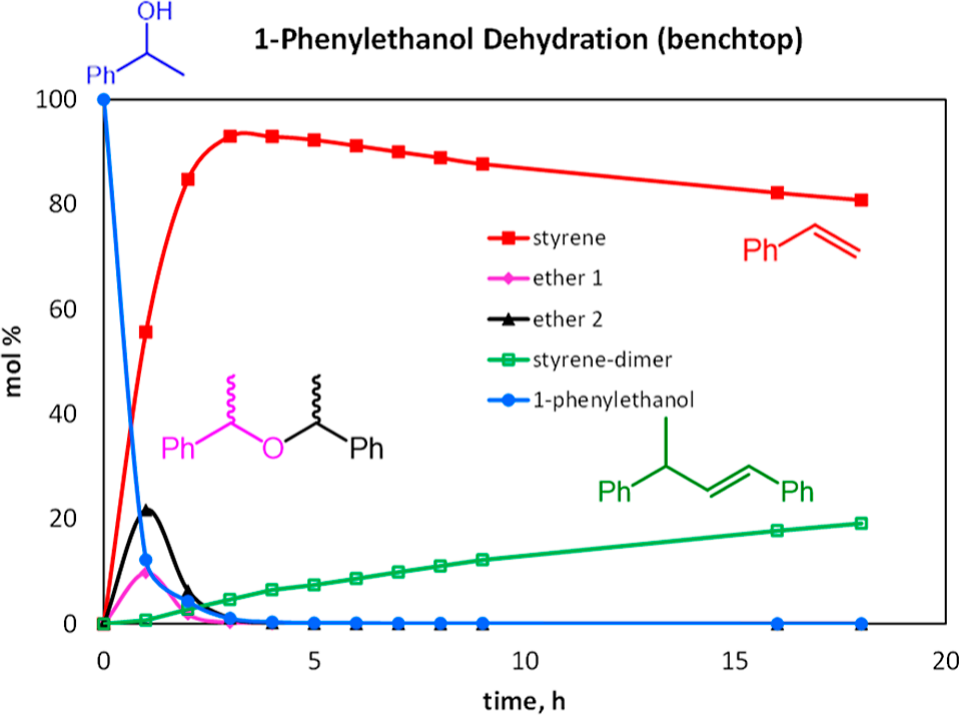
Distribution of species by ^1^H NMR spectroscopy in toluene-*d*_8_, 0.55 M 1-phenylethanol, 1 mol % **2a**, 120 °C.

Examination of the time course of the reaction at 120 °C was carried out by monitoring via ^1^H NMR spectroscopy, using 0.55 M 1-phenylethanol and 1 mol % **2a** in toluene-*d*_8_ in sealed NMR tubes. Remarkably, only styrene and styrene dimer were observed after 18 h (see Supporting Information, Figures S13.1–S13.5). Monitoring the reaction every hour showed that the α-methyl benzyl ethers formed but went away within 4 h. At 5 h, reaction was complete, and styrene was formed in >95% yield. Further heating leads to slow dimerization of styrene (Figure 3).

It was also found that this reaction only works well in toluene solvent. Other solvents, including tetrahydrofuran, acetonitrile, dioxane, benzene, or *o*-dichlorobenzene, showed little reaction of the phenylethanol. It was noticed that in toluene, the water that forms from the dehydration is immiscible. As tiny water droplets form, they turn deep blue indicating the Cu(II) catalyst is dissolved in the aqueous phase. During the reaction at 120 °C, the toluene is cloudy, with only a faint blue color (see pictures in the Supporting Information, Figure S29). Once cool, the toluene appears clear with a blue droplet of water at the bottom of the tube. Thus, it appears that the high selectivity observed is a result of the water separating from the toluene solvent, driving the dehydration reaction to completion.

**Figure 4:**
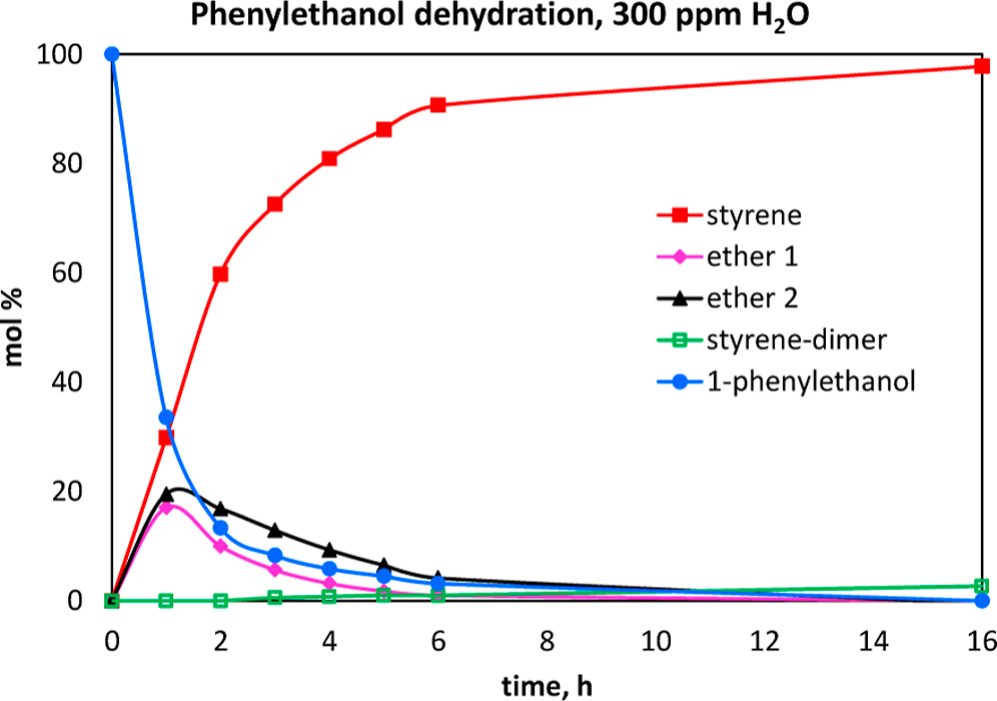
Plots of ^1^H NMR data for conversion of 1-phenylethanol (**A**) to styrene (**B**) at 0.55 M concentration of **A** in toluene-*d*_8_ at shorter reaction times. The ethers and styrene dimer were observed as byproducts of the reaction. 1 mol % **2a**, 120 °C.

Furthermore, if both the solvent and 1-phenylethanol are rigorously dried, the reaction is inhibited. Small quantities of water are required, and it was found that about 300 ppm water in the phenylethanol (3 μL H_2_O/10 mL **A**) gives results as shown in Figure 4. Large quantities of water inhibit the reaction. (See Supporting Information for results with 600, 1000 ppm, and neat water). It is also worth noting that the Cu^II^ catalyst **2a** is stable in air, so that these reactions can be prepared on the benchtop provided water content is kept at 300–1000 ppm.

Derivatives **2b** and **2c** were also examined for dehydration of 1-phenylethanol under the same conditions (0.83 M 1-phenylethanol, 120 °C, 1 mol % cat.). After 24 h, compound **2b** showed 99% conversion and styrene was obtained in 82% yield. Only traces of the ethers were seen. Compound **2c** showed 96% conversion with a 71% yield of styrene and 25% ethers. Therefore, catalyst **2a** is the best catalyst for styrene formation.

### Kinetic and Mechanistic Investigations

**Figure 5:**
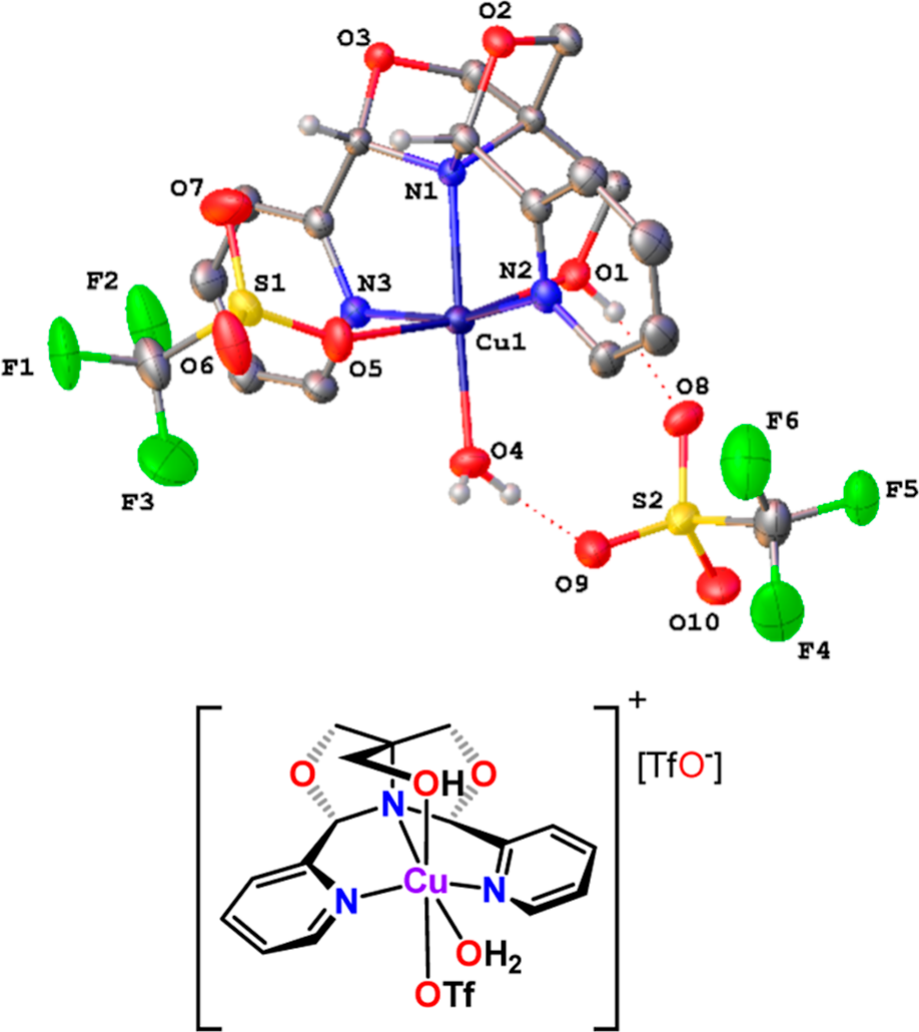
Depictions of the homogeneous catalyst [Cu(^meso^FOX-L1)(H_2_O)(OTf)][OTf] (**3a**). Crystal structure of **3a** (top) and a simplified structure showing the coordination environment of **3a** (bottom).

Our initial results with [(^meso^FOX-L1)Cu(CH_3_CN)](OTf)_2_ catalyst **2a** showed that the conversion of **A** as well as the formation and product distribution of **B**, **C**, and **D** were dependent on the substrate concentration, reaction time, and water content. After the completion of the reaction, [Cu(^meso^FOX-L1)(H_2_O)(OTf)][OTf] (**3a**) was isolated by crystallization from the reaction mixture. Figure 5 shows that a water has displaced the acetonitrile ligand in **2a**. Complex **3a** was then employed as catalyst for the dehydration of **A**. It was found that **3a** dehydrated a 0.83 M solution of **A** in toluene to afford ∼70% of **B**. A similar product distribution was observed when the reaction was conducted using complex **2a** with an equimolar amount of water (70% GC yield of styrene). When the reaction was conducted in the presence of water as a solvent, the reaction shut down completely, and almost all of the starting substrate **A** was recovered.

**Scheme 3:**
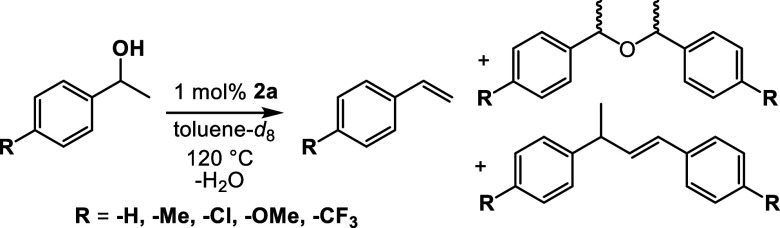
Reaction Conditions for Dehydration of Substituted Benzyl Alcohols

**Figure 6:**
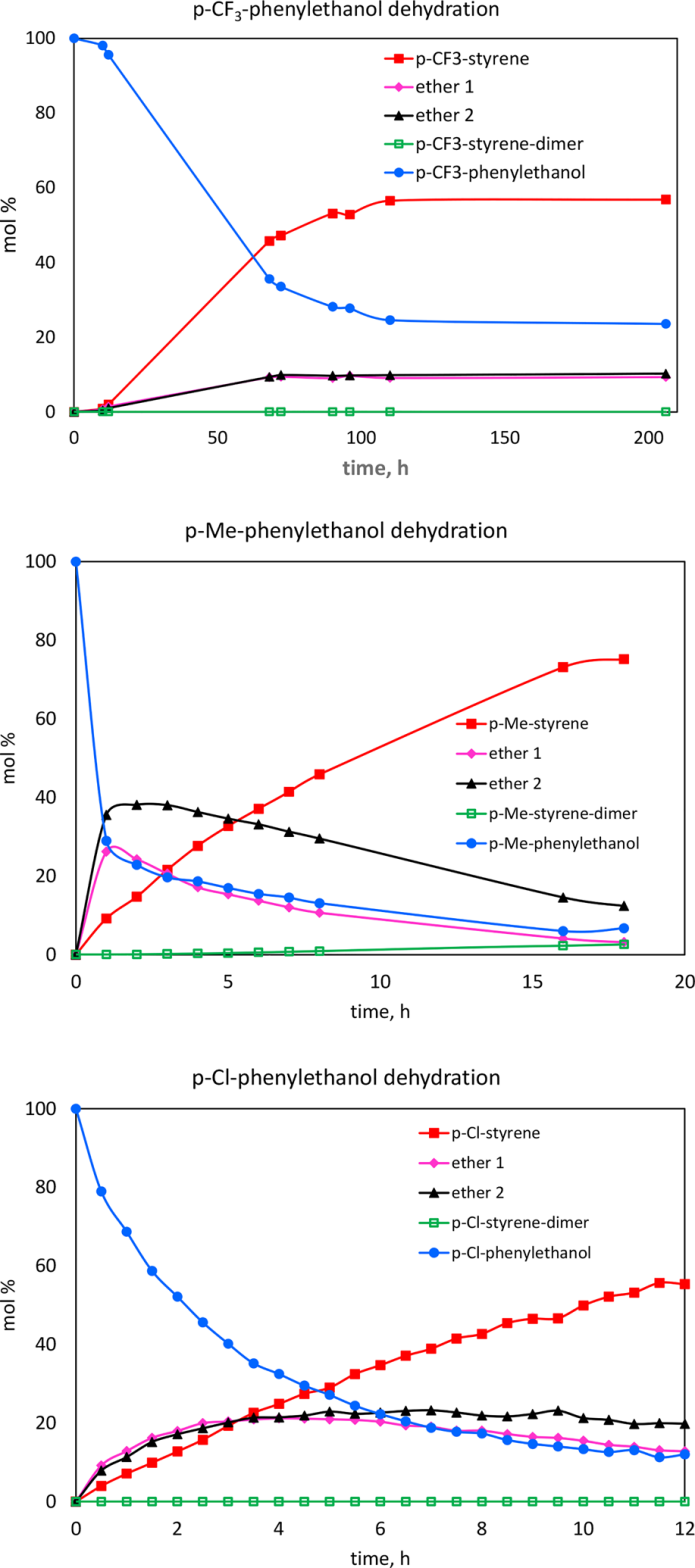
Plots of ^1^H NMR data recorded for dehydration of different substituted benzylic alcohols to their corresponding styrene analogs as depicted in Scheme 3. 0.55 M substrate, 120 °C, 1 mol % **2a**.

Following the above results and to elucidate the mechanism further, the substrate scope was examined (Scheme 3). It was found that upon substitution with electron-donating groups such as para-Me, the reaction rate decreased compared to the parent 1-phenylethanol, and an 80% maximum yield of 4-methylstyrene was observed. In reactions with substitution of the aromatic ring with electron-withdrawing groups such as 1-(4-chlorophenyl)ethanol, the yields of the corresponding styrene products were considerably lower (45% NMR yields) with the corresponding AMEs being formed as the major products. With 1-(4-trifluoromethylphenyl)ethanol, the reaction was extremely slow and appeared to reach equilibrium. Figure 6 shows the distribution of products observed over time with these substrates.

Interestingly, it was observed that for 4-methoxy-1-phenylethanol, the corresponding ethers were the major products, and 4-methoxy-styrene was observed as a minor product in trace amounts after 3 days of reaction. Additionally, in all these kinetic ^1^H NMR experiments, it was observed that the ethers were formed first but then re-entered the catalytic cycle to produce the styrene products. This was not initially apparent in the reaction of parent 1-phenylethanol due to the extremely fast reaction rate with the (^meso^FOX)Cu^II^ catalyst.

**Scheme 4:**
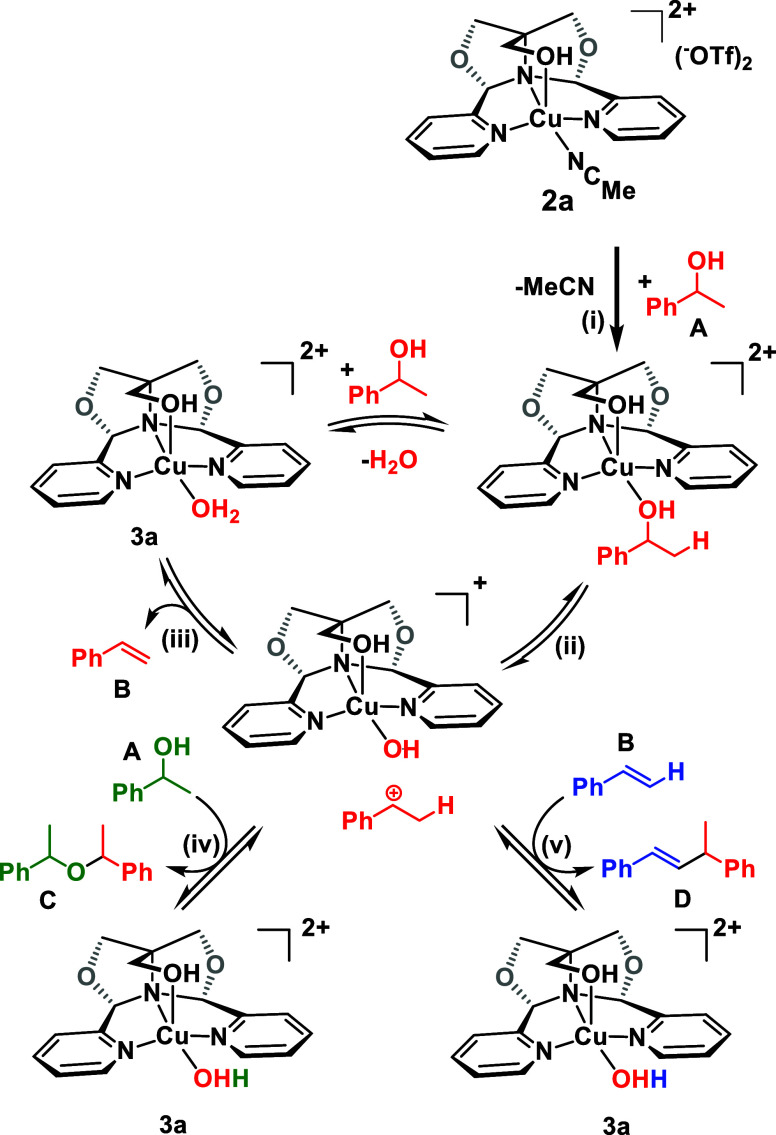
Proposed Mechanism for Alcohol Dehydration

These reactions are proposed to proceed via coordination of the phenylethanol to the Cu^II^ center (i), followed by dissociation of a benzylic carbocation (ii), as proposed for the Fe^II^ analog (Scheme 4).^17^ The carbocation can lose a proton to give styrene (iii), or react with phenylethanol to give the AMEs (iv), or react with styrene to give the observed styrene dimer **D** (v). Note that the formation of a disubstituted alkene is consistent with the carbocation pathway, and that Brønsted acids would isomerize this to the trisubstituted alkene. The observation of only **D** indicates that the dehydration is not being catalyzed by Brønsted acids. The rates for each of these steps is strongly affected by the para-substituent on the phenyl ring, as seen by the variation in rates seen in Figure 6.

These dehydrations all involve the release of a stable carbonium ion. Therefore, other alcohol substrates that would produce stabilized carbonium ions were examined. It was found that complex **2a** catalyzes the dehydration of benzylic, allylic, and tertiary alcohols specifically, which is consistent with the reaction proceeding through a carbocation intermediate. Given that these alcohols gave rise to more stabilized carbocations compared to secondary and primary alcohols, the reactions proceeded to give dehydrated products. Secondary alcohols such as cyclohexanol and cyclopentanol did not undergo dehydration and unreacted alcohols were recovered.

**Scheme 5:**
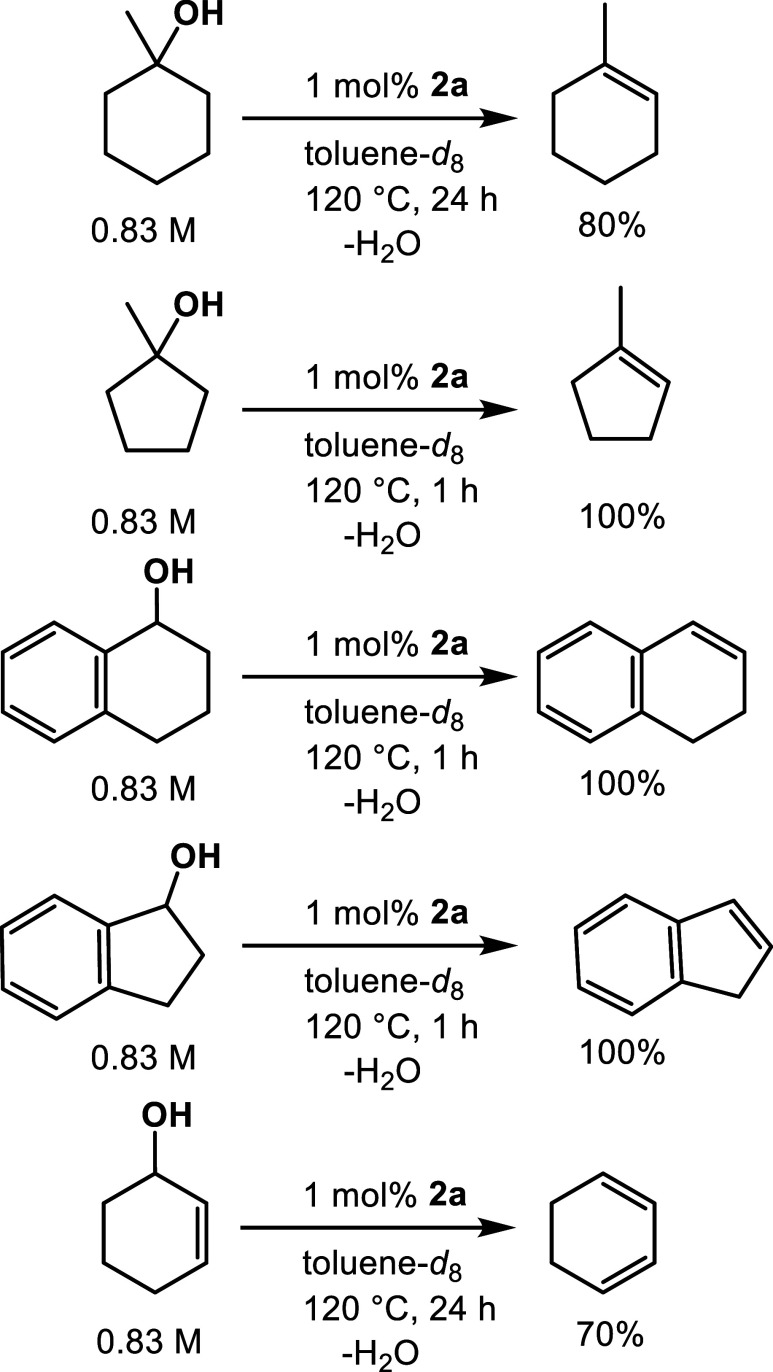
Conversion of Tertiary and Allylic Alcohols to Their Corresponding Alkenes (0.83 M Substrate in Toluene)

Further exploration of the scope of the reaction was undertaken. 1-Methylcyclohexanol was dehydrated to 1-methylcyclohexene in good yield. None of the exocyclic double bond material was observed. Similarly, 1-methylcyclopentanol gave 1-methylcyclopentene in quantitative yield. Reactions of both 1-tetralol and 1-indanol were followed by NMR spectroscopy and found to go to completion in quantitative yield in 1 h. No evidence for ethers or styrene-dimers was seen. 2-Cyclohexen-1-ol was dehydrated selectively to 1,3-hexadiene (Scheme 5).

**Scheme 6:**
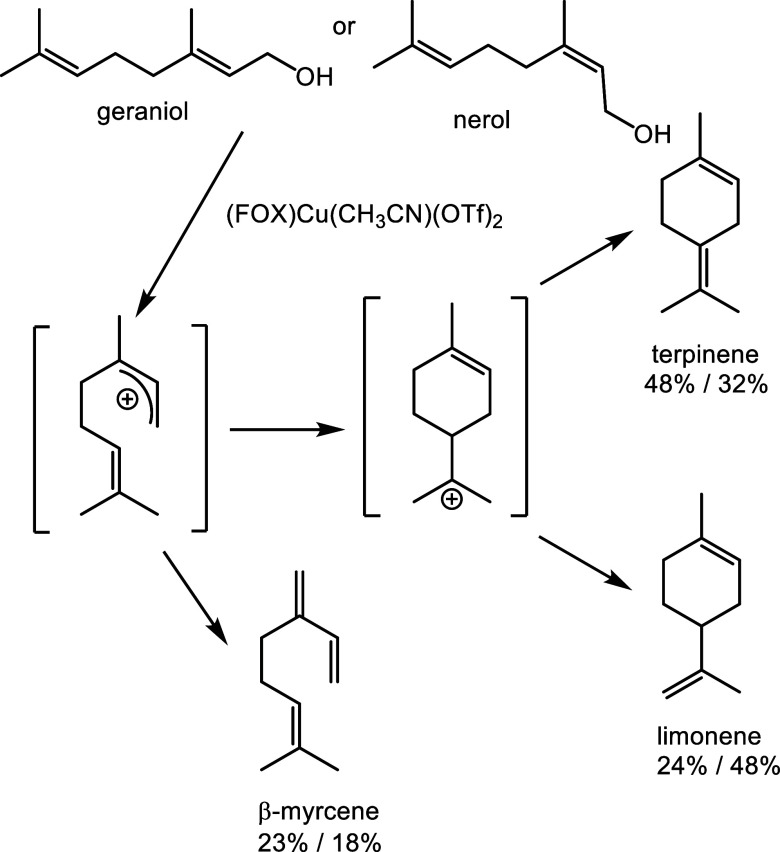
Dehydration Products Arising from Geraniol and Nerol

To test the hypothesis further, we tried the natural terpinols geraniol and nerol as substrates. These terpinols are geometric isomers and possess an allylic alcohol moiety. As expected, these alcohols dehydrated to give a mixture of limonene, terpinene, and β-myrcene (Scheme 6).

### Preparation of Zinc(II) FOX Complexes

**Scheme 7:**
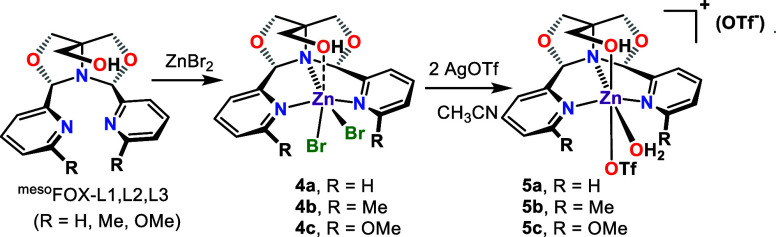
Preparation of Zn(II) Complexes

For comparison with the copper(II) FOX catalyst, a series of zinc(II) derivatives was prepared using the same method as shown for copper in Scheme 1. Three derivatives were examined as shown in Scheme 7, in which the pyridine is substituted adjacent to the nitrogen with either H, Me, or OMe (**4a–5c**). Single crystal X-ray structures were obtained for all but **4c** (see Supporting Information).

The first complex is formed by reaction of ZnBr_2_ with ^meso^FOX-L1, Zn(^meso^FOX-L1)Br_2_ (**4a**), which reacts with AgOTf to give Zn(^meso^FOX-L1)(H_2_O)(OTf)_2_ (**5a**). The single crystal X-ray structure of **5a** is analogous to that of **3a**, with a water and a triflate bound to the zinc. The second derivative has methyl groups adjacent to the pyridine nitrogen of the FOX ligand. Here, ^meso^FOX-L2 reacts with ZnBr_2_ to give Zn(^meso^FOX-L2)Br_2_ (**4b**), which then reacts with AgOTf to give Zn(^meso^FOX-L2)(H_2_O)(OTf)_2_ (**5b**), analogous to complexes **1b** and **2b**. A third derivative has methoxy groups adjacent to the pyridine nitrogen of the FOX ligand. The dibromide Zn(^meso^FOX-L3)Br_2_ (**4c**) reacts with AgOTf to give Zn(^meso^FOX-L3)(H_2_O)(OTf)_2_ (**5c**).

The zinc-FOX complexes **5a**, **5b**, and **5c** were examined for 1-phenylethanol dehydration under conditions similar to those used to examine the copper-FOX complex **2a**. The parent derivative, Zn(^meso^FOX-L1)(H_2_O)(OTf)_2_, showed little reaction in toluene after 24 h. The methyl derivative Zn(^meso^FOX-L2)(H_2_O)(OTf)_2_ showed 87% conversion of **A** and formation of styrene **B** in 28% yield. The methoxy derivative Zn(^meso^FOX-L3)(H_2_O)(OTf)_2_ showed 97% conversion of **A** and formation of styrene **B** in 44% yield. Similar results were observed in o-dichlorobenzene solvent. In addition to styrene, the α-methylbenzylethers were also observed as byproducts. Overall, the reactions with the zinc catalysts proved to be much slower than with copper catalyst **2a** when examined by NMR spectroscopy. Distribution of species plots over 120 h are shown in the Supporting Information for both catalysts **5b** and **5c**.

## Conclusions

The air stable copper(II) complex **2a** reported here is an effective catalyst for alcohol dehydration, being both faster than the previous Fe(FOX) catalyst (4 h vs 24 h) and giving much higher yields of styrene (>95% vs 74%). Alcohols that form stabilized carbonium ions (e.g., benzylic, tertiary, or allylic) are readily converted to olefins plus water. The rate of benzylic alcohol dehydration is found to be highly dependent upon the aryl substituents present. The intermediacy of ethers is seen with benzylic alcohols, but not with other alcohols. Derivative (FOX)M catalysts prepared using Zn(II) show much reduced activity compared to Cu(II) or Fe(II).

## Experimental Section

Elemental compositions were determined using a PerkinElmer 2400 Series II analyzer. NMR data were recorded on Bruker AVANCE 400 and AVANCE 500 spectrometers. GC–MS data were recorded on a Hewlett-Packard 5890 GC Series II equipped with a Hewlett-Packard 5970 Series mass selective detector. Quantitative GC was performed on a Shimadzu GC-2010 equipped with a flame ionization detector and an autosampler. A Rigaku Synergy-S diffractometer with a dual PhotonJet-S microfocus X-ray sources (Cu Kα, Mo Kα) and a HyPix-6000HE HPC detector was used for crystallographic structure determinations. NMR spectra and chromatograms are provided in the Supporting Information. ^1^H NMR spectra were recorded using a long relaxation delay (30 s) to ensure integrations were accurate (measured T1 values varied from 3 to 6 s).

All commercial reagents were used as received without further purification. All air and moisture-sensitive manipulations were performed using standard Schlenk techniques or in an inert atmosphere of purified nitrogen. Diethyl ether, toluene, and acetonitrile were used directly from an Innovative Technology, Inc., PS-MD-6 solvent purification system.

### Synthesis of Cu(^meso^FOX-L1)Br_2_ (**1a**)

To a clean dry 20 mL scintillation vial in a nitrogen-filled glovebox, ^meso^FOX-L1 (100 mg, 0.33 mmol) and CuBr_2_ (82 mg, 0.37 mmol) were dissolved in 5 mL of anhydrous acetonitrile. The reaction mixture was stirred for 30 min to precipitate a pale blue solid. The solid was collected by filtration, washed with anhydrous diethyl ether (6 mL), and dried in vacuo to afford a pale blue solid **1a** (148 mg, 0.284 mmol, 84%). Single crystals of the product were obtained by layering a saturated methanol solution of the complex with diethyl ether on the benchtop. A single crystal X-ray structure was obtained, showing one bound bromide and one outer sphere bromide, along with a water of crystallization. A ^1^H NMR spectrum showed only two broad resonances at δ 10.1 and −2.0. Anal. Calcd (found) for C_16_H_19_Br_2_N_3_O_5_Cu: 36.77 (36.24): % C, 3.28 (3.22): % H, 8.04: (7.79) %N.

### Synthesis of [Cu(^meso^FOX-L1)(MeCN)][OTf]_2_ (**2a**)

To a clean dry 20 mL scintillation vial in a nitrogen-filled glovebox, Cu(^meso^FOX-L1)Br_2_ (100 mg, 0.19 mmol) and AgOTf (98.3 mg, 0.38 mmol) were dissolved in 5 mL of anhydrous acetonitrile. The reaction mixture was stirred for 30 min to obtain a clear, blue-colored solution with the precipitation of yellow solid of AgBr. The yellow solid was discarded and the blue solution was reduced in vacuo and layered with anhydrous diethyl ether to afford single crystals of **2a** (107 mg, 0.15 mmol, 80%). Anal. calcd(found) for C_20_H_20_CuF_6_N_4_O_9_S_2_: 34.22 (33.94) %C, 2.87 (2.67) %H, 7.98 (7.87). μ_eff_ = 2.23 B.M. (Evans method^20^). A single crystal X-ray structure was obtained, showing a bound acetonitrile molecule with both triflates outer sphere. A ^1^H NMR spectrum showed 2 broad resonances at δ 10.7 and −0.1.

### Synthesis of Cu(^meso^FOX-L2)Br_2_ (**1b**)

To a clean dry 20 mL scintillation vial in a nitrogen-filled glovebox, ^meso^FOX-L2 (100 mg, 0.31 mmol) and CuBr_2_ (76 mg, 0.34 mmol) were dissolved in 5 mL of anhydrous acetonitrile. The reaction mixture was stirred for 45 min to precipitate a dark green solid. The solid was collected by filtration, washed with anhydrous diethyl ether (6 mL), and dried in vacuo to afford a pale blue solid (151 mg, 88%). Anal. Calcd (found) for C_18_H_21_Br_2_N_3_O_3_Cu: 39.26 (39.38) % C, 3.84 (4.02) % H, 7.63: (7.14) % N.

### Synthesis of [Cu(^meso^FOX-L2)(MeCN)][OTf_2_] (**2b**)

To a clean dry 20 mL scintillation vial in a nitrogen-filled glovebox, Cu(^meso^FOX-L2)Br_2_ (55 mg, 0.1 mmol) and AgOTf (51 mg, 0.2 mmol) were dissolved in 5 mL of anhydrous acetonitrile. The reaction mixture was stirred for 45 min to obtain a clear dark blue solution with the precipitation of yellow solid of AgBr. The yellow solid was discarded and the blue solution was reduced in vacuo and layered with anhydrous diethyl ether to afford single crystals (54 mg, 76%). Anal. Calcd (found) for C_22_H_24_CuF_6_N_4_O_8_S_2_: 37.00 (36.68) % C, 3.39 (3.26) % H, 7.85 (7.81) % N.

### Synthesis of Cu(^meso^FOX-L3)(MeCN)Br_2_ (**1c**)

To a clean dry 20 mL scintillation vial in a nitrogen-filled glovebox, ^meso^FOX-L3 (100 mg, 0.28 mmol) and CuBr_2_ (69 mg, 0.31 mmol) were dissolved in 5 mL of anhydrous acetonitrile. The reaction mixture was stirred for 45 min to precipitate a dark green solid. The solid was collected by filtration, washed with anhydrous diethyl ether (6 mL), and dried in vacuo to afford a lime green solid (145 mg, 89%). Anal. Calcd (found) for C_18_H_21_Br_2_N_3_O_5_Cu: 38.51 (38.10) % C, 3.84 (3.84) % H, 8.98 (8.91) % N.

### Synthesis of [Cu(^meso^FOX-L3)(MeCN)][OTf_2_] (**2c**)

To a clean dry 20 mL scintillation vial in a nitrogen-filled glovebox, Cu(^meso^FOX-L3)Br_2_ (55 mg, 0.094 mmol) and AgOTf (49 mg, 0.19 mmol) were dissolved in 5 mL of anhydrous acetonitrile. The reaction mixture was stirred for 45 min to obtain a clear dark blue solution with the precipitation of yellow solid of AgBr. The yellow solid was discarded and the blue solution was reduced in vacuo and layered with anhydrous diethyl ether to afford single crystals (55 mg, 78%). Anal. Calcd (found) for C_22_H_24_CuF_6_N_4_O_8_S_2_: 35.42 (35.64) % C, 3.24 (3.30) % H, 7.51(7.25) % N.

### Dehydration Studies with Cu Compounds

To a clean dry 5 mL Schlenk tube containing 1 mol % of **2a** (5.8 mg, 0.0083 mmol) and 1 mL of anhydrous toluene was added 0.83 mmol (0.1 mL) of 1-phenylethanol under a positive pressure of nitrogen. The tube was sealed using a Teflon stopper. The tube was then heated in an aluminum heating block at 120 °C for 24 h. The same reaction procedure was used for the reactions containing 2.0 and 4.5 M 1-phenylethanol substrate loadings. A crystal of the catalyst was isolated at the end of the reaction, and a single crystal X-ray structure of **3a** was obtained, showing a bound water molecule, a bound triflate, and an outer sphere triflate. Similar reactions were carried out with **2b** and **2c**.

### Kinetic Study Using Unsubstituted and Substituted 1-Phenylethanols

To a clean dry J-Young NMR tube containing 1 mol % of **2a** (2.9 mg, 0.0041 mmol) and 0.7 mL of toluene-*d*_8_ was added 0.05 mL (0.414 mmol) of 1-phenylethanol under a nitrogen atmosphere in a glovebag. The tube was then sealed with a Teflon stopper and heated in an aluminum heating block at 120 °C. ^1^H NMR spectra were recorded at various time points to monitor the progress of the reaction. Some experiments were also conducted in sealed NMR tubes.

### Dehydration Experiment to Check Activity of the Water Phase

To a clean dry J-Young NMR tube, 1 mol % of **2a** (2.9 mg, 0.0041 mmol) and 0.7 mL of toluene-*d*_8_ were added under an inert atmosphere. The tube was then introduced into a dinitrogen filled glovebag in which 0.414 mmol (50 μL) of 1-phenylethanol was added along with a known volume (15 μL) of TMS (as an internal standard). The tube was then stoppered and heated in an aluminum heating block at 120 °C with ^1^H NMR spectra recorded at various times over 5 h (complete consumption of substrate with 98% yield of styrene). The tube was then reintroduced into a dinitrogen filled glovebag and the liquid was decanted carefully. The tube was then attached to the vacuum line and the remaining liquid was removed under reduced pressure cautiously so that the catalyst stays at the bottom. The tube was then introduced into dinitrogen filled glovebag in which 0.414 mmol (∼50 μL) of 1-phenylethanol was added along with known volume (∼15 μL) of TMS (as an internal standard) and toluene-*d*_8_ (0.7 mL). The tube was then stoppered and heated in an aluminum heating block at 120 °C with ^1^H NMR spectra recorded at various times. Styrene was observed in 74% yield.

## Supporting Information Available

Includes additional experimental details, synthesis of the zinc complexes, GC traces, NMR spectra, and tables of distribution of species for reactions; X-ray data for **1a–5c** (PDF)
The X-ray structural data are available from the Cambridge Structural Database (CCDC #2369770–2369777) (CIF)
(CIF)
(CIF)
(CIF)
(CIF)
(CIF)
(CIF)
(CIF)

## Supplementary Material

cs4c04572_si_001.pdf

cs4c04572_si_002.cif

cs4c04572_si_003.cif

cs4c04572_si_004.cif

cs4c04572_si_005.cif

cs4c04572_si_006.cif

cs4c04572_si_007.cif

cs4c04572_si_008.cif

cs4c04572_si_009.cif

## References

[ref1] Bockisch C.; Lorance E. D.; Hartnett H. E.; Shock E. L.; Gould I. R. Kinetics and Mechanisms of Dehydration of Secondary Alcohols Under Hydrothermal Conditions. ACS Earth Space Chem. 2018, 2, 821–832. 10.1021/acsearthspacechem.8b00030.

[ref2] Adkins H.; Perkins P. P. Dehydration of Alcohols over Alumina. J. Am. Chem. Soc. 1925, 47, 1163–1167. 10.1021/ja01681a036.

[ref3] Zhang W.; Wang B.; Yang J.; Rui P.; Fan N.; Liao W.; Shu X. Zeolite Fe-MFI as Catalysts in the Selective Liquid-Phase Dehydration of 1-Phenylethanol. Catal. Commun. 2018, 110, 97–101. 10.1016/j.catcom.2018.03.018.

[ref4] Bertero N. M.; Apesteguía C. R.; Marchi A. J. Liquid-Phase Dehydration of 1-Phenylethanol over HZSM-5 Kinetic Modeling. Catal. Commun. 2009, 10, 1339–1344. 10.1016/j.catcom.2009.02.018.

[ref5] Zhang W.; Cheng G.; Haller G. L.; Liu Y.; Lercher J. A. Rate Enhancement of Acid-Catalyzed Alcohol Dehydration by Supramolecular Organic Capsules. ACS Catal. 2020, 10, 13371–13376. 10.1021/acscatal.0c03625.

[ref6] Khan N. A.; Hwang J.-S.; Jhung S.-H. Liquid-Phase Dehydration of 1-Phenylethanol to Styrene over an Acidic Resin Catalyst. Bull. Korean Chem. Soc. 2011, 32, 1327–1330. 10.5012/bkcs.2011.32.4.1327.

[ref7] Ward D. J.; Saccomando D. J.; Walker G.; Mansell S. M. Sustainable Routes to Alkenes: Applications of Homogeneous Catalysis to the Dehydration of Alcohols to Alkenes. Catal. Sci. Technol. 2023, 13, 2638–2647. 10.1039/D2CY01690G.

[ref8] Petersen A. R.; Fristrup P. New Motifs in Deoxydehydration: Beyond the Realms of Rhenium. Chem.–Eur. J. 2017, 23, 10235–10243. 10.1002/chem.201701153. 28423204

[ref9] Raju S.; Moret M.-E.; Klein Gebbink R. J. M. Rhenium-Catalyzed Dehydration and Deoxydehydration of Alcohols and Polyols: Opportunities for the Formation of Olefins from Biomass. ACS Catal. 2015, 5, 281–300. 10.1021/cs501511x.

[ref10] Lange J.-P.; Otten V. Dehydration of Phenyl Ethanol to Styrene under Reactive Distillation Conditions: Understanding the Catalyst Deactivation. Ind. Eng. Chem. Res. 2007, 46, 6899–6903. 10.1021/ie070397g.

[ref11] 1-Phenylethanol is produced as a co-product with propylene oxide in the Shell SMPO Process. The reaction of O_2_ with ethylbenzene forms ethylbenzene hydroperoxide which then transfers oxygen to propylene. See: Buijink J. K. F.; Lange J. P.; Bos A. N. R.; Horton A. D.; Niele F. G. M. Propylene Epoxidation via Shell’s SMPO Process: 30 Years of Research and Operation. In Mechanisms in Homogeneous and Heterogeneous Epoxidation Catalysis; Oyama S. T., Ed.; Elsevier Science & Technology: The Netherlands, 2008; pp 355–372.

[ref12] Korstanje T. J.; Jastrzebski J. T. B. H.; Klein Gebbink R. J. M. Catalytic Dehydration of Benzylic Alcohols to Styrenes by Rhenium Complexes. ChemSusChem 2010, 3, 695–697. 10.1002/cssc.201000055. 20468027

[ref13] Zhu Z.; Espenson J. H. Organic Reactions Catalyzed by Methylrhenium Trioxide: Dehydration, Amination, and Disproportionation of Alcohols. J. Org. Chem. 1996, 61, 324–328. 10.1021/jo951613a.

[ref14] Laali K.; Gerzina R. J.; Flajnik C. M.; Geric C. M.; Dombroski A. M. Copper(II) Triflate, a New Reagent for Mild Dehydration of Alcohols: Synthetic Usefulness and Mechanistic Insight. Helv. Chim. Acta 1987, 70, 607–611. 10.1002/hlca.19870700314.

[ref15] Allahverdiyev A.; Yang J.; Gröger H. Catalyst Screening for Dehydration of Primary Alcohols from Renewable Feedstocks under Formation of Alkenes at Energy-Saving Mild Reaction Conditions. Green Chem. 2024, 26, 7869–7878. 10.1039/D4GC01038H.

[ref16] Hoffman R. V.; Bishop R. D.; Fitch P. M.; Hardenstein R. Anhydrous Copper(II) Sulfate: An Efficient Catalyst for the Liquid-Phase Dehydration of Alcohols. J. Org. Chem. 1980, 45, 917–919. 10.1021/jo01293a030.

[ref17] Nachtigall O.; VanderWeide A. I.; Brennessel W. W.; Jones W. D. An Iron-Based Dehydration Catalyst for Selective Formation of Styrene. ACS Catal. 2021, 11, 10885–10891. 10.1021/acscatal.1c03037.

[ref18] It has been reported that Cu(OTf)_2_ does catalyze dehydration of 1-phenylethanol at 160 °C and 10% catalyst loading. See: Laali K.; Gerzina R. J.; Flajnik C. M.; Geric C. M.; Dombroski A. M. Copper(II) Triflate, a New Reagent for Mild Dehydration of Alcohols: Synthetic Usefulness and Mechanistic Insight. Helv. Chim. Acta 1987, 70, 607–611. 10.1002/hlca.19870700314.

[ref19] A reaction using 0.83 M 1-phenylethanol in toluene with 1% Cu(OTf)_2_ and 2 equiv bipyridine showed 86% conversion and a 31% yield of styrene after 24 h at 120 °C.

[ref20] Evans D. F. The Determination of the Paramagnetic Susceptibility of Substances in Solution by Nuclear Magnetic Resonance. J. Chem. Soc. 1959, 2003–2005.

