# Supplementary material for: Efficient Dehydration of 1-Phenylethanol to Styrene by Copper(II) and Zinc(II) Fused-Bisoxazolidine Catalysts
Source: ACS Catal. 2024 Oct 11;14(21):15927–34. doi: 10.1021/acscatal.4c04572 (PMC11536342; doi:10.1021/acscatal.4c04572)
Supplement: Supplementary file 1 — cs4c04572_si_001.pdf [file cs4c04572_si_001.pdf]

Supporting Information for

Efficient Dehydration of 1-Phenylethanol to Styrene by Copper(II) and Zinc(II) Fused-bisoxazolidine Catalysts

Aurodeep Panda,[a] Caroline R. Wood,[b] William W. Brennessel,[a] and William D. Jones[a]*

[a] Department of Chemistry, University of Rochester, Rochester, New York 14627, United States; orcid.org/0000-0003-1932-0963
[b] Department of Chemistry, State University of New York at New Paltz, New Paltz, New York 12561, United States

Email: jones@chem.rochester.edu

Experimental procedures for dehydrations followed by GC and NMR spectroscopy S-2
Experimental procedures for preparation of zinc-FOX compounds S-4
Scale up of dehydration of 1-phenylethanol using **2a** S-5
Figure S1-3. GC traces for dehydration of 1-phenylethanol at 4.5-0.83 M by **2a** in toluene S-6
Figure S4. GC traces for dehydration of 1-phenylethanol at 0.83 M by $Cu(OTf)_2$ in toluene S-9
Figure S5. GC trace for dehydration of 1-phenylethanol at 0.83 M by $Cu(OTf)_2$/bipy in toluene S-10
Figure S6. GC traces for dehydration of 1-phenylethanol at 0.83 M by **2a** in water S-11
Figure S7. GC traces for dehydration of 1-methylcyclohexanol at 0.83 M by **2a** in toluene S-12
Figure S8. GC traces for dehydration of 2-cyclohexene-1-ol at 0.83 M by **2a** in toluene S-13
Figure S9. GC traces for dehydration of nerol at 0.72 M by **2a** in toluene S-14
Figure S10. GC traces for dehydration of geraniol at 0.72 M by **2a** in toluene S-15
Figure S11. GC traces for dehydration of 1-phenylethanol at 0.83 M by **2b** in toluene S-16
Figure S12. GC traces for dehydration of 1-phenylethanol at 0.83 M by **2c** in toluene S-17
Figure S13.1-13.4 ^{1}H NMR spectra of 1-PhEtOH dehydration, styrene dimer, & simulations S-18
Figure S13.5. Distribution of species plots–Phenylethanol dehydration (benchtop PhEtOH) S-22
Figure S14.1–17.4. Distribution of species plots–PhEtOH dehydration (300-1000 ppm H_2O) S-23
Figure S18.1-18.4. Distribution of species plots–Substituted p-CF_3PhEtOH dehydration S-39
Figure S19.1-19.4. Distribution of species plots–Substituted p-CH_3PhEtOH dehydration S-43
Figure S20.1-20.4. Distribution of species plots–Substituted p-ClPhEtOH dehydration S-47
Figure S21.1-21.3. Distribution of species plots–Substituted p-MeOPhEtOH dehydration S-51
Figure S22.1-22.4. Distribution of species plots–1-phenylpropanol dehydration S-54
Figure S23.1-23.4. Distribution of species plots–1-indanol dehydration S-58
Figure S24.1-24.4. Distribution of species plots–1-tetralol dehydration S-62
Figure S25.1-25.3. Distribution of species plots–1-methylcyclopentanol dehydration S-66
Figure S26. Dehydration of 1-phenylethanol (0.55 M) by **2a** in toluene at 120 °C - recycle S-69
Figure S27. Distribution of species plots–1-phenylethanol dehydration with Zn(FOX-L2)$(OTf)_2$ S-70
Figure S28. Distribution of species plots–1-phenylethanol dehydration with Zn(FOX-L3)$(OTf)_2$ S-71
Figure S29. Photos of dehydration reaction of [(^{meso}FOX-L1)Cu(CH_3CN)]$(OTf)_2$ S-72
Figure S30. Evans method ^{19}F NMR spectrum of **2a** S-73
Figures S31-S36. ^{1}H NMR spectra of compounds **4a-5c** S-74
X-ray data for Complexes:
X-ray data for (^{meso}FOX-L1)$CuBr_2$ (**1a**) S-80
X-ray data for [(^{meso}FOX-L1)Cu(CH_3CN)]$(OTf)_2$ (**2a**) S-94
X-ray data for [(^{meso}FOX-L1)Cu(H_2O)(OTf)](OTf) (**3a**) S-111
X-ray data for (^{meso}FOX-L1)$ZnBr_2$ (**4a**) S-130
X-ray data for (^{meso}FOX-L2)$ZnBr_2$ (**4b**) S-144
X-ray data for [(^{meso}FOX-L1)Zn(H_2O)(OTf)](OTf) (**5a**) S-161
X-ray data for [(^{meso}FOX-L2)Zn(OTf)](OTf) (**5b**) S-181
X-ray data for [(^{meso}FOX-L3)Zn(H_2O)(OTf)](OTf) (**5c**) S-191

Experimental Procedures for Dehydrations followed by GC and NMR Spectroscopy

Dehydration of phenylethanol by GC. To a clean dry Schlenk tube fitted with a Teflon stopper and sidearm, 1 mol% of complex **2a** (2.9 mg, 0.0041 mmol) and 0.7 mL of toluene were added under inert atmosphere. The tube was then attached to the Schlenk line. Under a positive pressure of dinitrogen, 0.414 mmol (50 μL) of substrate was added. The tube was then heated in an aluminium heating block at 120 °C. After 24 h the tube was cooled in an ice bath and the contents transferred to a vial. 40 μL n-decane was added as internal standard. 50 μL of this solution was diluted with 1 mL toluene and then analyzed by GC. Column: DB-WAXETR, 30 m, 0.25 mm, 0.50 μm film. GC Program: 2 mL/min He flow, 50° → 230° over 35 min. Reponse factors for styrene and acetophenone measured vs. decane. Response factor for styrene dimer obtained using trisubstituted isomer. Response factor for α-methylbenzylethers assumed to be the same as for styrene dimer (both C_{16}).

Dehydrations were examined in toluene at 4.5, 2.0, and 0.83 M 1-phenylethanol. In a separate example, the procedure was modified by using water as the solvent with 0.83 M 1-phenylethanol. The water was extracted with diethyl ether before analysis by GC. In another example, the procedure was modified by using 1% $Cu(OTf)_2$ as catalyst. See Supporting Information for GC chromatograms (Figures S1-5).

Detailed GC program: 20.3 psi He, linear velocity – 41.2 cm/s; T-program: 50 °C for 5 min, ramp at 15°C/min to 145 °C and hold 3 min, ramp at 5°C/min to 230 °C and hold for 5 min. 36.3 min total.

1-methylcyclohexanol. The reaction was carried out in a sealed Schlenk tube as above and analyzed by GC after 17 h at 120 °C. Decane was added as internal standard. GC showed formation of 1-methylcyclohexene in 80 % yield and no 1-methylcyclohexanol (Figure S6).

2-cyclohexen-1-ol. The reaction was carried out in a sealed Schlenk tube as above and analyzed by GC after 13 h at 120 °C. Decane was added as internal standard. GC showed formation of cyclohexa-1,3-diene in 70% yield (Figure S7).

Dehydration of geraniol and nerol using complex 2a. The following procedure was employed to investigate the dehydration of nerol and geraniol, which were used as received without any modifications and thus contained impurities. To a clean dry 5 mL Schlenk tube containing 1 mol % of **2a** (5.8 mg, 0.0083 mmol) and 1 mL of anhydrous toluene was added 0.83 mmol (0.144 mL) of the substrate under a positive pressure of nitrogen. The tube was sealed using a Teflon stopper and then heated in an aluminum heating block at 120 °C for 24 h. After the reaction was completed, the reaction mixture was passed through a short plug of celite and was analyzed using GC with n-decane as the internal standard (Figures S8 & S9).

Kinetic study of dehydration of alcohols by ^{1}H NMR spectroscopy by 2a. The following procedure was followed to examine dehydrations of 1-phenylethanol, 1-(p-tolyl)ethanol, 1-(p-CF_3-phenyl)ethanol, 1-(p-Cl-phenyl)ethanol, 1-(p-MeO-phenyl)ethanol, 1-phenylpropanol, 1-methylcyclohexanol, 1-methylcyclopentanol, 2-cyclohexen-1-ol, 1-indanol, and 1-tetralol. To a clean dry NMR tube with a 14/20 ground glass joint, 1 mol% of complex **2** (2.9 mg, 0.0041 mmol) and 0.7 mL of toluene-d_8 were added under inert atmosphere. The tube was then attached to the vacuum line with the help of a 180° adapter. Under a positive pressure of dinitrogen, 0.414 mmol (~50-60 μL) of substrate was added along with a known volume (~15 μL) of TMS (as internal standard). The tube was flame sealed and then heated in an aluminium heating block at 120 °C. ^{1}H NMR spectra were recorded at various time points using a long relaxation

delay (30 s) to ensure integrations were accurate (measured T_1 values varied from 3-6 s). In some reactions, the reaction was carried out in a J-Young NMR tube with no internal standard. The spectra were carefully baseline corrected and integrated to determine the distribution of species vs. time. Specific comments for the substrates are given below.

1-Phenylethanol. The reactions proceeded to give both rac- and meso-α-methylbenzyl ethers as well as styrene (Figures S13.1-S13.4). After 18 h, all alcohol substrate had been converted to styrene or styrene-dimer. Simulation of the styrene-dimer ^{1}H NMR spectrum shows it to be the disubstituted olefin isomer (Figure S13.5). Reactions were also conducted using carefully dried substrate and toluene solvent. Little or no reaction occurred. Reactions were also conducted by adding known amounts of water to dry 1-phenylethanol (300 ppm, 600 ppm, 1000 ppm, Figures S14.1-S17.4). These all showed accelerated rates that were not entirely reproducible due to the heterogeneous nature of the reaction. For example, see two plots @600 ppm H_2O (Figures S15.4 and S16.4). One took 16 h to go to completion, whereas run#2 was complete in only 1 h.

1-(p-CF_3phenyl)ethanol. The reaction was very slow, taking ~100 h to go to ~55% completion. The reaction appeared to reach equilibrium. Both ethers were observed (~10%), but the concentrations of all species remained unchanged after 100 h. No styrene dimer was seen (Figures S18.1-S18.4).

1-(p-tolyl)ethanol. The reaction proceeded at a rate similar to that seen for 1-phenylethanol. The ethers and phenylethanol appeared to reach equilibrium and then slowly convert to p-methylstyene over 20 h (Figures S19.1-S19.4).

1-(p-Clphenyl)ethanol. The reaction produced p-chlorostyrene and the ethers which only slowly converted to p-chlorostyrene.~50% conversion to the styrene was seen after 12 h (Figures S20.1-S20.4).

1-(p-MeOphenyl)ethanol. The reaction proceded to make the ethers, but little styrene was observed (Figures S21.1-S21.3).

1-(phenyl)propanol. The reaction proceded over 18 h to give trans-β-methylstyrene (94%) and cis-β-methylstyrene (6%). A small quantity (9%) of one ether was observed after 1 h that converted to styrene by the end of the reaction (Figures S22.1-S22.4).

1-indanol. The reaction was complete (95%) within 1 h. No alcohol substrate remained, and trace ether was observed. An indene-dimer (13%) was observed to slowly form over 17 h (Figures S23.1-S23.4).

1-tetralol. The reaction was complete (100%) to give dihydronaphthalene in 1 h. No other intermediates or products were observed (Figures S24.1-S24.4).

1-methylcyclopentanol. The reaction was complete after 1 h to give exclusively 1-methylcyclopentene (Figures S25.1-S25.3).

Synthesis of 1-tetralol. In a 100 mL round-bottomed flask with a side arm, 1.1 g (7.52 mmol) of tetralone was dissolved in 20 mL dry MeOH and cooled in an ice bath. To the cooled orange colored solution, 1.70 g (45.1 mmol) of $NaBH_4$ was added under a positive pressure of nitrogen. This resulted in a color change to yellow and the reaction was stirred for 2 h. The reaction was worked up using a saturated NH_4Cl

solution and the aqueous layer was extracted with EtOAc (3 × 10 mL). The solvent was removed under vacuum and the product dried under vacuum. A ^{1}H-NMR spectum matched that reported for this compound.[1]

Experimental Procedures for Preparation of Zinc-FOX Compounds

Preparation of Zinc-FOX complexes 4a-5c. The preparations of the meso-FOX ligand (L1) and the dimethyl (L2) and dimethoxy (L3) derivatives have been reported previously.[2]

Zn(FOX-L1)Br$_2$ (4a). $ZnBr_2$ (0.1660 g, 0.735 mmol) was added to a 20 mL scintillation vial and dissolved with minimal acetonitrile. A stir bar was added to the vial and stirring was started. FOX-L1 (0.2206 g, 0.737 mmol) was added to a separate 20 mL scintillation vial with minimal acetonitrile. The contents of the second scintillation vial were added into the first scintillation vial dropwise. Stirring continued at room temperature overnight. The product was isolated as a solid through gravity filtration. Recrystallization from a mixture of methanol and diethyl ether yielded a white crystalline solid (0.340 g, 88%). Anal calcd(found) for $C_{16}H_{17}Br_2N_3O_3Zn$: C, 36.64 (36.35); H, 3.27 (3.17); N, 8.01 (7.87).

Zn(FOX-L2)Br$_2$ (4b): $ZnBr_2$ (0.3446 g, 1.53 mmol) was added to a 20 mL scintillation vial and dissolved with minimal acetonitrile. A stir bar was added to the vial and stirring was started. FOX-L2 (0.5000 g, 1.53 mmol) was added to a separate 20 mL scintillation vial with minimal acetonitrile. The contents of the second scintillation vial were added into the first scintillation vial dropwise. Stirring continued at room temperature overnight. The product was isolated as a solid through gravity filtration. Recrystallization from a mixture of methanol and diethyl ether yielded a white crystalline solid (0.690 g, 82%). Anal calcd(found) for $C_{18}H_{21}Br_2N_3O_3Zn$: C, 39.13 (39.01); H, 3.83 (3.83); N, 7.60 (7.48).

Zn(FOX-L3)Br$_2$ (4c): $ZnBr_2$ (0.0627 g, 0.278 mmol) was added to a 20 mL scintillation vial and dissolved with minimal acetonitrile. A stir bar was added to the vial and stirring was started. FOX-L3 (0.1000 g, 0.278 mmol) was added to a separate 20 mL scintillation vial with minimal acetonitrile. The contents of the second scintillation vial were added into the first scintillation vial dropwise. Stirring continued at room temperature overnight. The product was isolated as a solid through gravity filtration. Recrystallization from a mixture of methanol and diethyl ether yielded a white crystalline solid (0.117 g, 72%). Anal calcd(found) for $C_{18}H_{21}Br_2N_3O_5Zn$: 36.98 (37.14); H, 3.62 (3.31); N, 7.19 (7.87).

[Zn(FOX-L1)(OTf)(H$_2$O)](OTf) (5a): Zn(FOX-L1)Br_2 (0.3236 g, 0.617 mmol) was added to a 20 mL scintillation vial and dissolved with minimal acetonitrile. A stir bar was added to the vial and stirring was started. AgOTf (0.3170 g, 0.123 mmol) was added to a separate 20 mL scintillation vial with minimal acetonitrile. The contents of the second scintillation vial were added into the first scintillation vial dropwise. Stirring continued at room temperature overnight. The product was isolated as a clear solution, and the waste, AgBr, crashed out as a yellow solid. Recrystallization from a mixture of acetonitrile and diethyl

---

(1) Funk, P.; Richrath, R. B.; Bohle, F.; Grimme, S.; Gansäuer, A. Oxidation Under Reductive Conditions: From Benzylic Ethers to Acetals with Perfect Atom-Economy by Titanocene(III) Catalysis. *Angew.Chem. Int. Ed.* **2021**, *60*, 5482–5488 (see p.S-34). https://doi.org/10.1002/anie.202013561.

(2) Nachtigall, O.; VanderWeide, A. I.; Brennessel, William. W.; Jones, W. D. First-Row Transition Metals Complexes with Fused Oxazolidine (FOX) Ligands. *Z. Anorg. Allg. Chem.* **2021**, *647*, 1442–1448.

ether yielded a white crystalline solid (0.376 g, 81%). Anal calcd(found) for $C_{18}H_{19}F_6N_3O_{10}S_2Zn$: C,32.62 (32.24); H, 2.59 (2.88); N, 6.34 (6.59).

[Zn(FOX-L2)(OTf)](OTf) (5b): Zn(FOX-L2)Br_2 (0.6899 g, 1.25 mmol) was added to a 20 mL scintillation vial and dissolved with minimal acetonitrile. A stir bar was added to the vial and stirring was started. AgOTf (0.6416 g, 2.50 mmol) was added to a separate 20 mL scintillation vial with minimal acetonitrile. The contents of the second scintillation vial were added into the first scintillation vial dropwise. Stirring continued at room temperature overnight. The product was isolated as a clear solution, and the waste, AgBr, crashed out as a yellow solid. Recrystallization from a mixture of acetonitrile and diethyl ether yielded a white crystalline solid (0.599 g, 65%). Anal calcd(found) for $C_{20}H_{21}F_6N_3O_9S_2Zn$: C,34.77 (34.68); H, 3.06 (2.99); N, 6.08 (6.04).

[Zn(FOX-L3)(H_2O)(OTf)](OTf) (5c) : Zn(FOX-L3)Br_2 (0.1170 g, 0.200 mmol) was added to a 20 mL scintillation vial and dissolved with minimal acetonitrile. A stir bar was added to the vial and stirring was started. AgOTf (0.1028 g, 0.400 mmol) was added to a separate 20 mL scintillation vial with minimal acetonitrile. The contents of the second scintillation vial were added into the first scintillation vial dropwise. Stirring continued at room temperature overnight. The product was isolated as a clear solution, and the waste, AgBr, crashed out as a yellow solid. Recrystallization from a mixture of acetonitrile and diethyl ether yielded a white crystalline solid (0.102 g, 69%). Anal calcd(found) for $C_{20}H_{23}F_6N_3O_{12}S_2Zn$: C,33.23 (33.08); H, 2.93 (3.30); N, 5.81 (5.15).

Procedure for dehydration of 1-phenylethanol by zinc-FOX compounds 5a, 5b, and 5c. The zinc catalyst (0.0083 mmol) was added to a 5 mL Schlenk tube with 1-phenylethanol (0.101 g, 0.83 mmol). Then 1 mL of toluene was added to the Schlenk tube as well. The reaction was heated at 120 °C for 24 hours. 50 μL n-decane was added as internal standard, and the sample was analyzed by GC.

Kinetic study of dehydration of alcohols by ^{1}H NMR spectroscopy using zinc complexes 5a, 5b & 5c. A solution of zinc catalyst (0.0032 g, 0.00414 mmol) and 1-phenylethanol (0.0506 g, 0.414 mmol) was prepared in 0.7 mL toluene-d_8 and sealed in a J-Young NMR tube. The sample was heated to 120 °C, and NMR spectra recorded at several intervals over a few days. Complex **5a** showed no reaction, but **5b** and **5c** both produced styrene (96% and 35%, respectively, Figures S27 and S28).

Scale up reaction of styrene from dehydration of 1-phenylethanol. To a clean dry 300 mL Schlenk flask, 1 mol % of **2a** (0.0872 mg), 15 mL of anhydrous toluene and 1.5 mL (0.0124 mol) 1-phenylethanol were added under a positive pressure of dinitrogen. The flask was then sealed using a Teflon coated stopper. The flask was then heated in an oil-bath at 120 °C for 38 h. After 38 h, the solution was evaporated under reduced pressure (20 mm Hg) and chromatographed on neutral alumina using ethyl acetate in hexanes (1.5%, v:v). The combined fractions containing only styrene were treated with BHT (20 mg) to prevent polymerization. Evaporation under reduced pressure gave a crude product of 1.462 g containing styrene, ethyl acetate and hexanes (+BHT). Isolated yield of styrene, 0.513 g (40 %).

Figure S1. Dehydration of 1-phenolethanol (4.5M), 1% catalyst, toluene, 120 °C, 24 h

SHIMADZU LabSolutions

Analysis Report

<Sample Information>

Sample Name	: AP-03-014-15h-crm		
Sample ID	: AP-03-014-15h-crm		
Data Filename	: AP-03-014-15h-crm01.gcd		
Method Filename	: Styrene.gcm		
Batch Filename	:		
Vial #	: 1	Sample Type	: Unknown
Injection Volume	: 1 uL		
Date Acquired	: 11/4/2022 10:04:23 AM	Acquired by	: wdjgroup
Date Processed	: 6/24/2024 12:08:58 PM	Processed by	: wdjgroup

<Chromatogram>

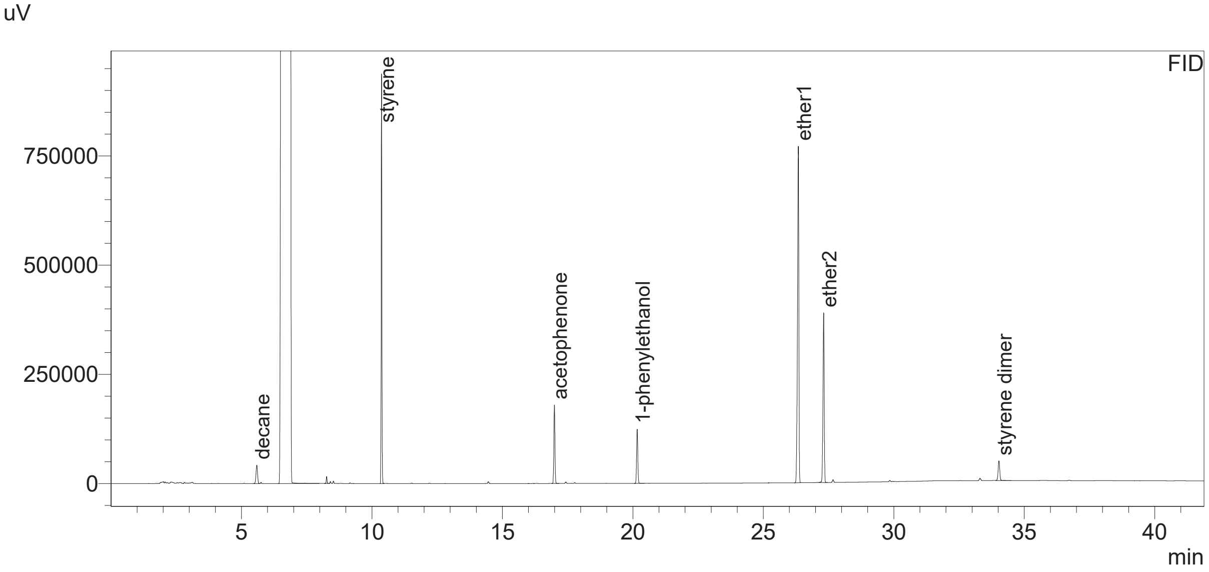

<Peak Table>

FID

Peak#	Ret. Time	Area	Height	Conc.	Unit	Mark	Name
1	5.588	175047	42132	-0.000	ppm		decane
2	6.859	354914713	30490090	0.000		S	toluene
3	8.264	34544	16058	0.000		V	
4	10.368	1706216	931318	-0.000	ppm		styrene
5	16.993	514953	178318	-0.000	ppm		acetophenone
6	20.165	399088	123260	-0.000	ppm	S	1-phenylethanol
7	26.341	2766122	768498	-0.000	ppm		ether1
8	27.313	1280286	387830	-0.000	ppm	V	ether2
9	34.028	201356	45365	-0.000	ppm	V	styrene dimer
Total		361992325	32982868				

C:\LabSolutions\Data\Aurodeep\alcohol_dehydration\100C-reacn\AP-03-014-15h-crm01.gcd

Figure S2. Dehydration of 1-phenolethanol (2.0 M), 1% catalyst, toluene, 120 °C, 24 h

SHIMADZU LabSolutions

Analysis Report

<Sample Information>

Sample Name	: AP-03-013-15h-crm		
Sample ID	: AP-03-013-15h-crm		
Data Filename	: AP-03-013-15h-crm01.gcd		
Method Filename	: Styrene.gcm		
Batch Filename	:		
Vial #	: 1	Sample Type	: Unknown
Injection Volume	: 1 uL		
Date Acquired	: 11/4/2022 9:11:36 AM	Acquired by	: wdjgroup
Date Processed	: 6/24/2024 12:04:30 PM	Processed by	: wdjgroup

<Chromatogram>

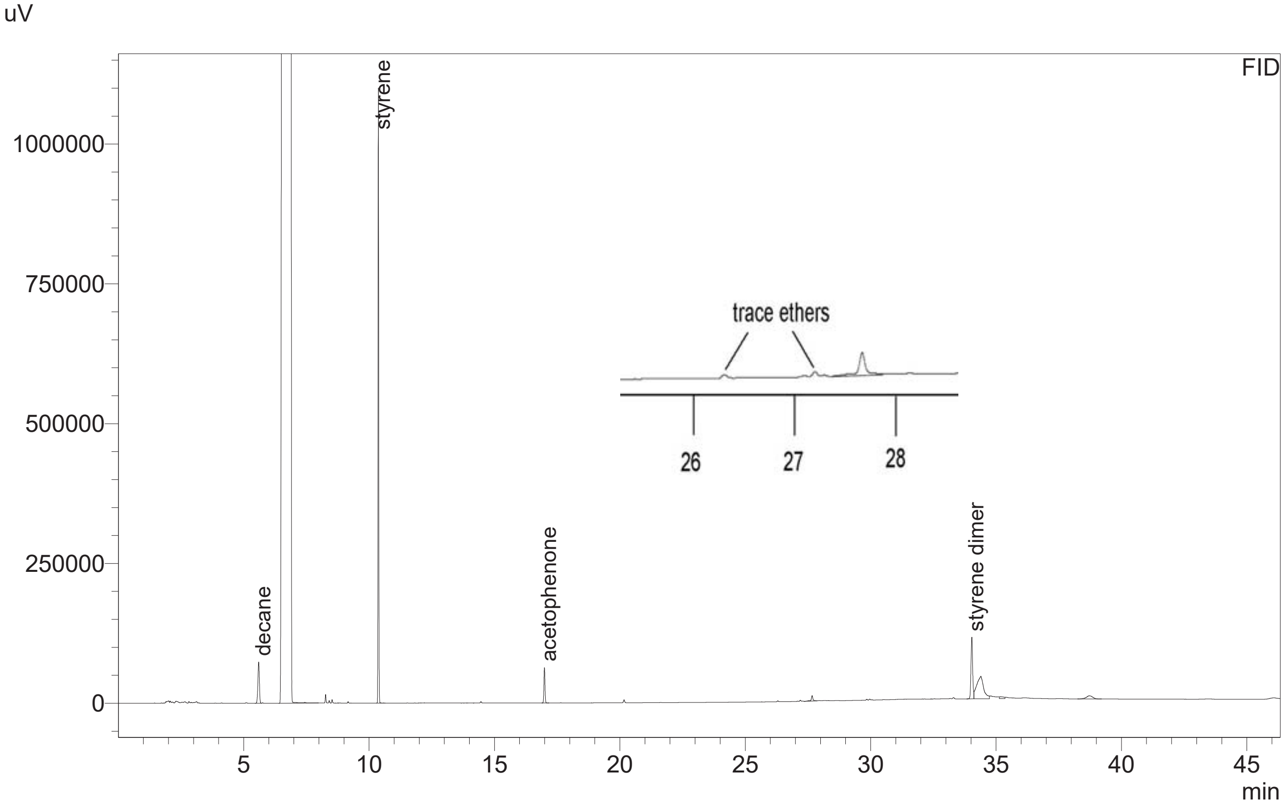

<Peak Table>

FID

Peak#	Ret. Time	Area	Height	Conc.	Unit	Mark	Name
1	5.598	305092	73714	-0.000	ppm		decane
2	6.862	354006785	31122149	0.000		S	toluene
3	10.370	2044292	1077792	-0.000	ppm	S	styrene
4	16.992	186569	62865	-0.000	ppm		acetophenone
5	27.666	51415	9898	0.000			
6	34.034	511264	110003	-0.000	ppm		styrene dimer
7	34.394	778270	40406	0.000		V	
8	35.189	41827	3441	0.000		V	
9	38.715	103434	5684	0.000			

C:\LabSolutions\Data\Aurodeep\alcohol_dehydration\100C-reacn\AP-03-013-15h-crm01.gcd

Figure S3. Dehydration of 1-phenolethanol (0.83 M), 1% catalyst, toluene,120 °C, 24 h

SHIMADZU LabSolutions

Analysis Report

<Sample Information>

Sample Name : AP-03-010-22h-crm
Sample ID : AP-03-010-22h-crm
Data Filename : AP-03-010-22h-crm01.gcd
Method Filename : Styrene.gcm
Batch Filename :
Vial # : 1
Sample Type : Unknown
Injection Volume : 1 uL
Date Acquired : 11/1/2022 2:44:51 PM
Acquired by : wdjgroup
Date Processed : 11/1/2022 3:21:14 PM
Processed by : wdjgroup

<Chromatogram>

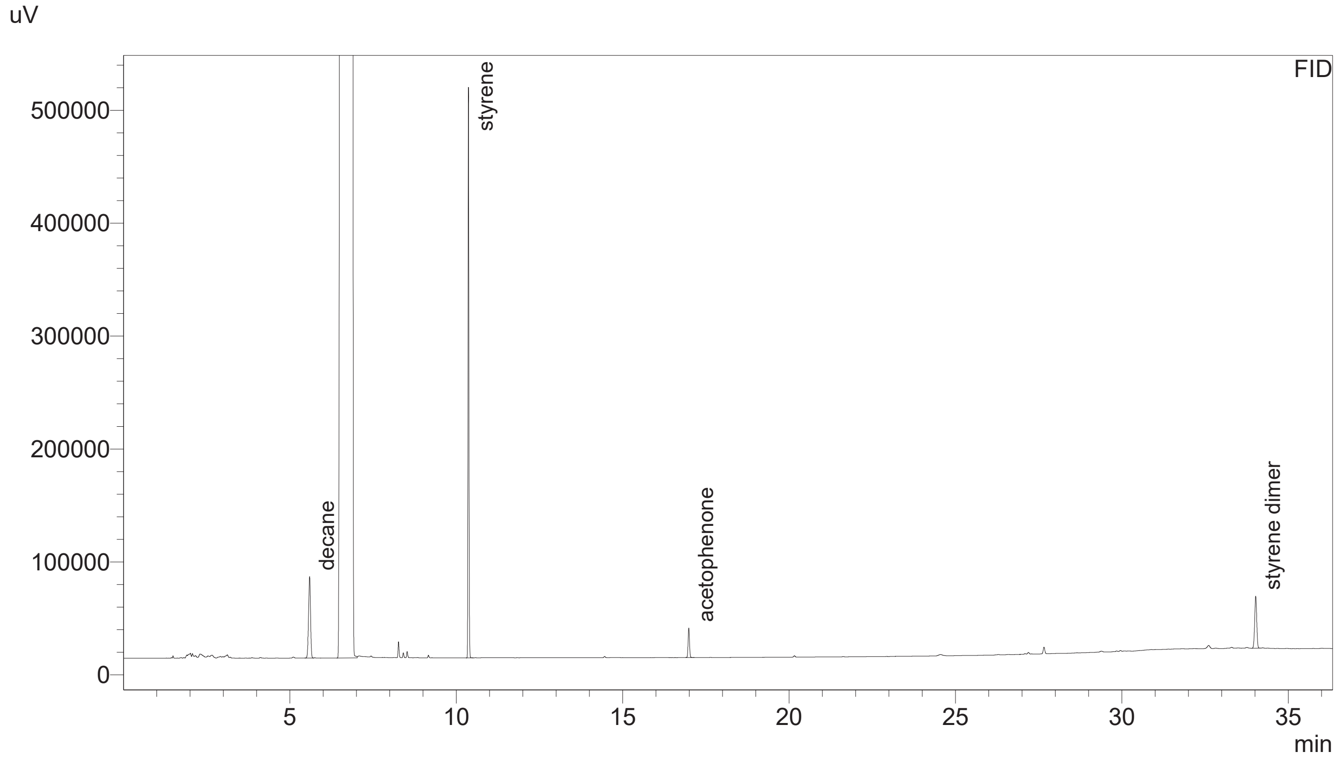

<Peak Table>

FID

Peak#	Ret. Time	Area	Height	Conc.	Unit	Mark	Name
1	5.592	299267	71913	-0.000	ppm		decane
2	6.858	349195527	30841649	0.000			toluene
3	10.364	924746	500890	-0.000	ppm		styrene
4	16.985	77525	26227	-0.000	ppm		acetophenone
5	34.021	194827	45897	-0.000	ppm		styrene dimer
Total		350691892	31486576				

C:\LabSolutions\Data\Aurodeep\alcohol_dehydration\100C-reacn\AP-03-010-22h-crm01.gcd

Figure S4. Dehydration of 1-phenolethanol (0.83 M), 1 mol% $Cu(OTf)_2$, toluene, 120 °C, 24 h -CONTROL

SHIMADZU LabSolutions

Analysis Report

<Sample Information>

Sample Name	: AP-03-024-control		
Sample ID	: AP-03-024-control		
Data Filename	: AP-03-024-control02.gcd		
Method Filename	: Styrene.gcm		
Batch Filename	:		
Vial #	: 1	Sample Type	: Unknown
Injection Volume	: 1 uL		
Date Acquired	: 12/5/2022 4:45:33 PM	Acquired by	: wdjgroup
Date Processed	: 12/5/2022 5:10:36 PM	Processed by	: wdjgroup

<Chromatogram>

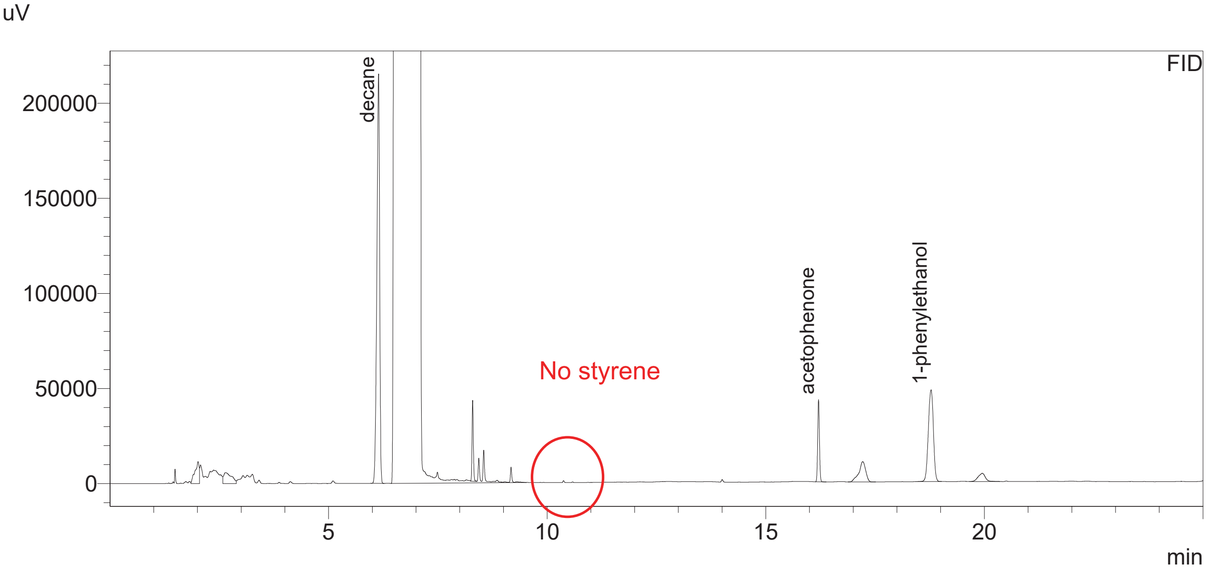

<Peak Table>

FID

Peak#	Ret. Time	Area	Height	Conc.	Unit	Mark	Name
1	2.014	77107	11554	0.000		V	
2	2.639	73780	5720	0.000		V	
3	6.143	950402	214897	-0.000	ppm		decane
4	7.061	881615312	54839995	0.000		S	toluene
5	8.295	88895	42342	0.000		TV	
6	16.207	120881	42917	-0.000	ppm		acetophenone
7	17.215	116761	10712	0.000			
8	18.782	389321	48268	-0.000	ppm		1-phenylethanol
9	19.952	50512	4366	0.000		V	
Total		883482970	55220772				

C:\LabSolutions\Data\Aurodeep\alcohol_dehydration\100C-reacn\AP-03-024-control02.gcd

Figure S5. Dehydration of 1-phenolethanol (0.83 M), 1 mol% Cu(OTf)2, 2 mol% bipy, toluene, 120 °C, 24 h

SHIMADZU LabSolutions

Analysis Report

<Sample Information>

Sample Name	: AP-04-099-24h		
Sample ID	: AP-04-099-24h		
Data Filename	: AP-04-099-24h.gcd		
Method Filename	: Alcohols (FID ONLY)-35 min run.gcm		
Batch Filename	:		
Vial #	: 1	Sample Type	: Unknown
Injection Volume	: 1 uL		
Date Acquired	: 9/7/2024 9:16:51 PM	Acquired by	: wdjgroup
Date Processed	: 9/10/2024 1:16:52 PM	Processed by	: wdjgroup

<Chromatogram>

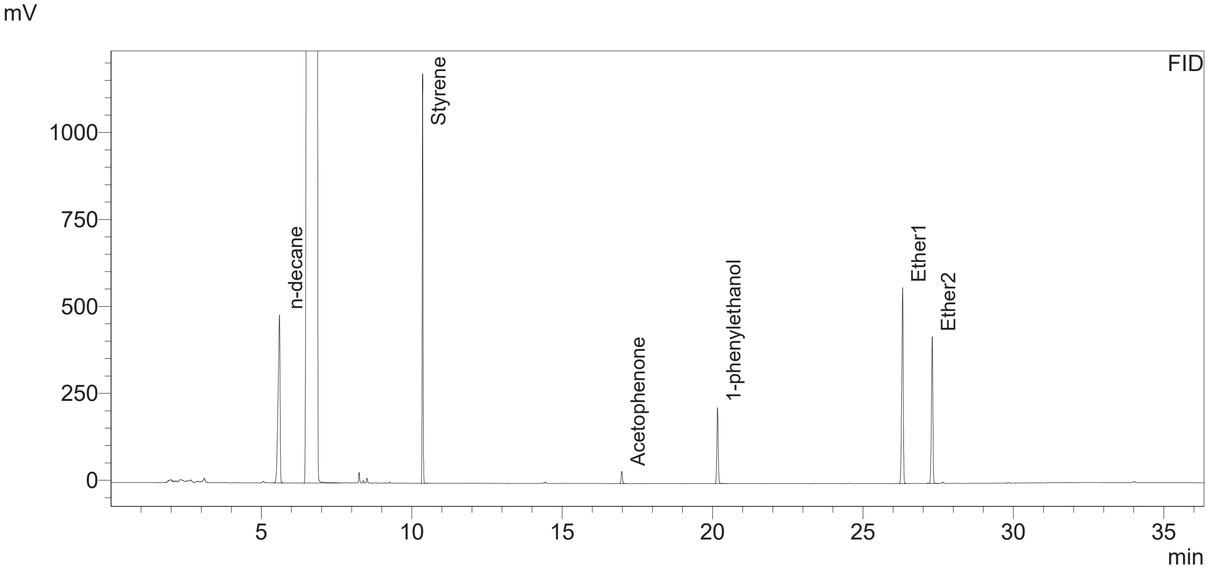

<Peak Table>

FID

Peak#	Ret. Time	Area	Height	Conc.	Unit	Mark	Name
1	5.601	2212902	480335	0.000	ppm		n-decane
2	6.838	340037363	30284580	0.000		SV	
3	10.359	2162294	1165754	0.000	ppm		Styrene
4	16.981	101324	34750	0.000	ppm		Acetophenone
5	20.164	687312	217715	0.000	ppm	V	1-phenylethanol
6	26.318	1943714	562027	0.000	ppm		Ether1
7	27.301	1389698	418917	0.000	ppm	SV	Ether2
Total		348534607	33164079				

C:\LabSolutions\Data\Aurodeep\alcohol_dehydration\100C-reacn\AP-04-099-24h.gcd

Figure S6. Dehydration of 1-phenolethanol (0.83 M), 1% catalyst, water solvent, 120 °C, 24 h

SHIMADZU LabSolutions

Analysis Report

<Sample Information>

Sample Name	: AP-03-034-20h-et2o		
Sample ID	: AP-03-034-20h-et2o		
Data Filename	: AP-03-034-20h-et2o01.gcd		
Method Filename	: Styrene.gcm		
Batch Filename	:		
Vial #	: 1	Sample Type	: Unknown
Injection Volume	: 1 uL		
Date Acquired	: 12/22/2022 4:13:50 PM	Acquired by	: wdjgroup
Date Processed	: 12/22/2022 4:38:52 PM	Processed by	: wdjgroup

<Chromatogram>

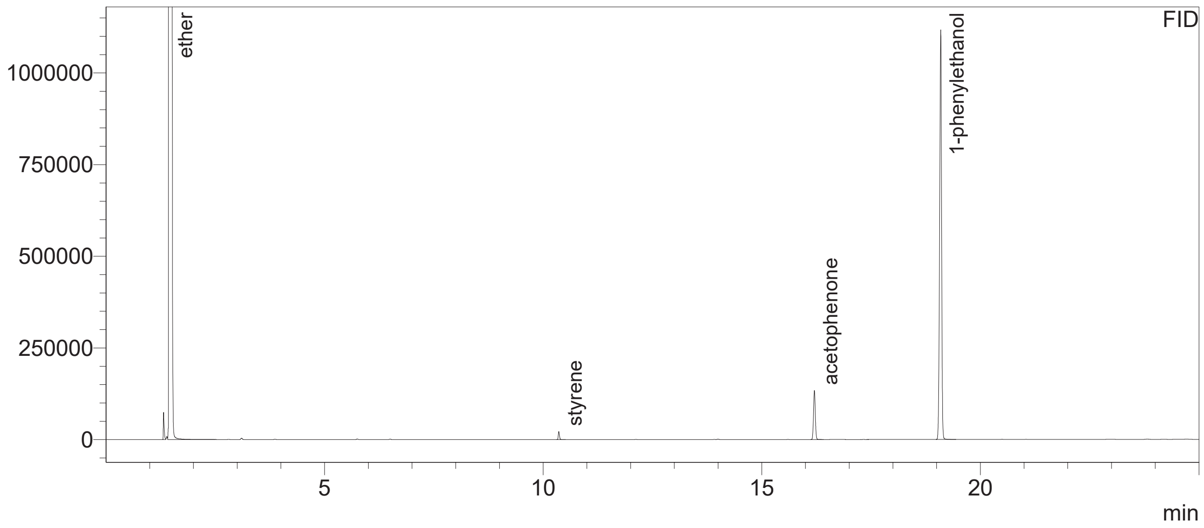

<Peak Table>

FID

Peak#	Ret. Time	Area	Height	Conc.	Unit	Mark	Name
1	1.318	98664	71772	0.000			
2	1.448	373583152	157885214	-0.000	ppm	SV	ether
3	10.358	41835	22072	-0.000	ppm		styrene
4	16.204	377836	132872	-0.000	ppm		acetophenone
5	19.094	3444178	1113578	-0.000	ppm		1-phenylethanol
Total		377545665	159225509				

C:\LabSolutions\Data\Aurodeep\alcohol_dehydration\100C-reacn\AP-03-034-20h-et2o01.gcd

Figure S7. Dehydration of 1-methylcyclohexanol (0.83 M), 1% catalyst, toluene, 120 °C, 24 h

SHIMADZU LabSolutions

Analysis Report

<Sample Information>

Sample Name	: AP-03-018-17h-crm		
Sample ID	: AP-03-018-17h-crm		
Data Filename	: AP-03-018-17h-crm01.gcd		
Method Filename	: Alcohols (FID ONLY)-45 min run.gcm		
Batch Filename	:		
Vial #	: 1	Sample Type	: Unknown
Injection Volume	: 1 uL		
Date Acquired	: 11/8/2022 12:49:28 PM	Acquired by	: wdjgroup
Date Processed	: 7/1/2024 4:07:30 PM	Processed by	: wdjgroup

<Chromatogram>

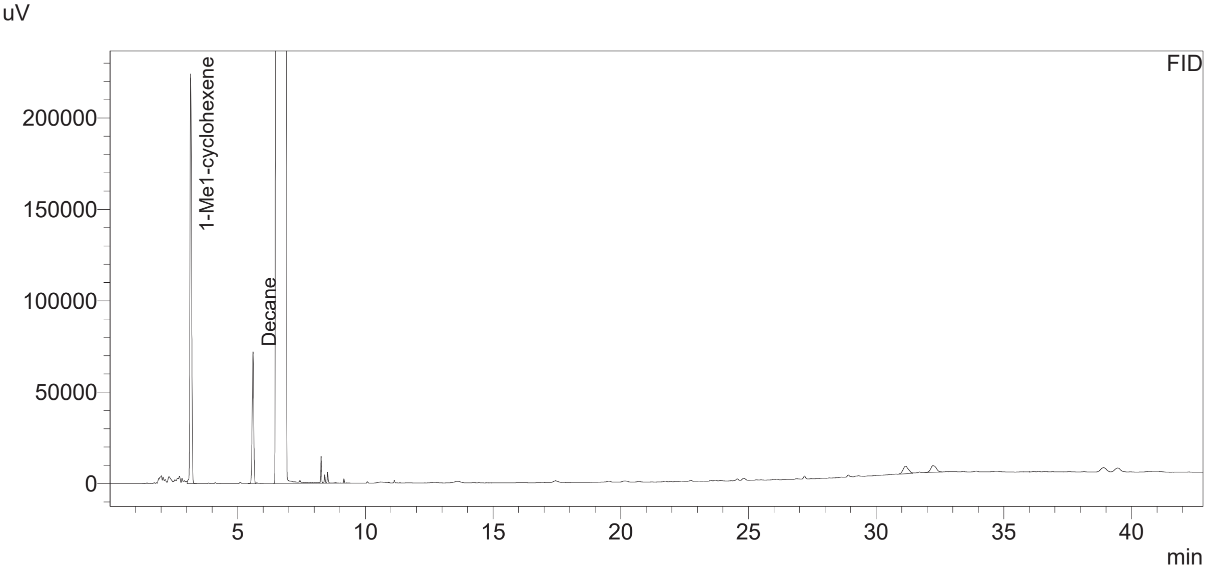

<Peak Table>

FID

Peak#	Ret. Time	Area	Height	Conc.	Unit	Mark	Name
1	3.159	995457	223716	-0.000	ppm	V	1-Me1-cyclohexene
2	5.601	300021	71736	0.000	ppm		Decane
3	6.863	355920803	30646599	0.000		S	
4	31.168	61436	4128	0.000		V	
5	32.220	55620	3637	0.000			
Total		357333337	30949816				

C:\LabSolutions\Data\Aurodeep\alcohol_dehydration\100C-reacn\AP-03-018-17h-crm01.gcd

Figure S8. Dehydration of 2-cyclohexene-1-ol (0.83M), 1% catalyst, toluene, 120 °C, 24 h

SHIMADZU LabSolutions

Analysis Report

<Sample Information>

Sample Name	: AP-03-028-13h		
Sample ID	: AP-03-028-13h		
Data Filename	: AP-03-028-13h01.gcd		
Method Filename	: Alcohols (FID ONLY)-25 min run.gcm		
Batch Filename	:		
Vial #	: 1	Sample Type	: Unknown
Injection Volume	: 1 uL		
Date Acquired	: 12/9/2022 9:58:45 AM	Acquired by	: wdjgroup
Date Processed	: 7/1/2024 4:37:54 PM	Processed by	: wdjgroup

<Chromatogram>

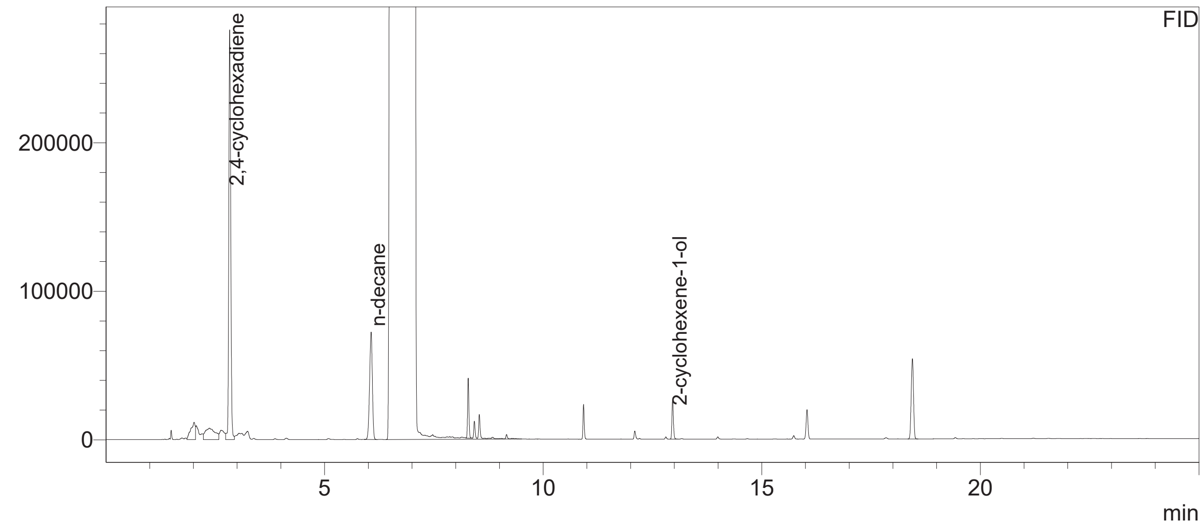

<Peak Table>

FID

Peak#	Ret. Time	Area	Height	Conc.	Unit	Mark	Name
1	2.015	81955	11670	0.000		V	
2	2.365	121956	7739	0.000		V	
3	2.830	860856	274669	-0.000	ppm	V	2,4-cyclohexadiene
4	6.065	317139	72231	0.000	ppm		n-decane
5	7.039	818461554	52163000	0.000		S	
6	8.285	83998	40047	0.000		TV	
7	12.961	62253	26139	-0.000	ppm		2-cyclohexene-1-ol
8	18.447	198538	53972	0.000			
Total		820188249	52649468				

C:\LabSolutions\Data\Aurodeep\alcohol_dehydration\100C-reacn\AP-03-028-13h01.gcd

Figure S9. Dehydration of nerol (0.72M), 1% catalyst, toluene, 120 °C, 24 h

SHIMADZU LabSolutions

Analysis Report

<Sample Information>

Sample Name	: AP-04-087-B-nerol-18h		
Sample ID	: AP-04-087-B-nerol-18h		
Data Filename	: AP-04-087-B-nerol-18h.gcd		
Method Filename	: Alcohols (FID ONLY)--terpenols-28 min run.gcm		
Batch Filename	:		
Vial #	: 1	Sample Type	: Unknown
Injection Volume	: 1 uL		
Date Acquired	: 7/19/2024 2:58:11 PM	Acquired by	: wdjgroup
Date Processed	: 7/23/2024 11:48:14 AM	Processed by	: wdjgroup

<Chromatogram>

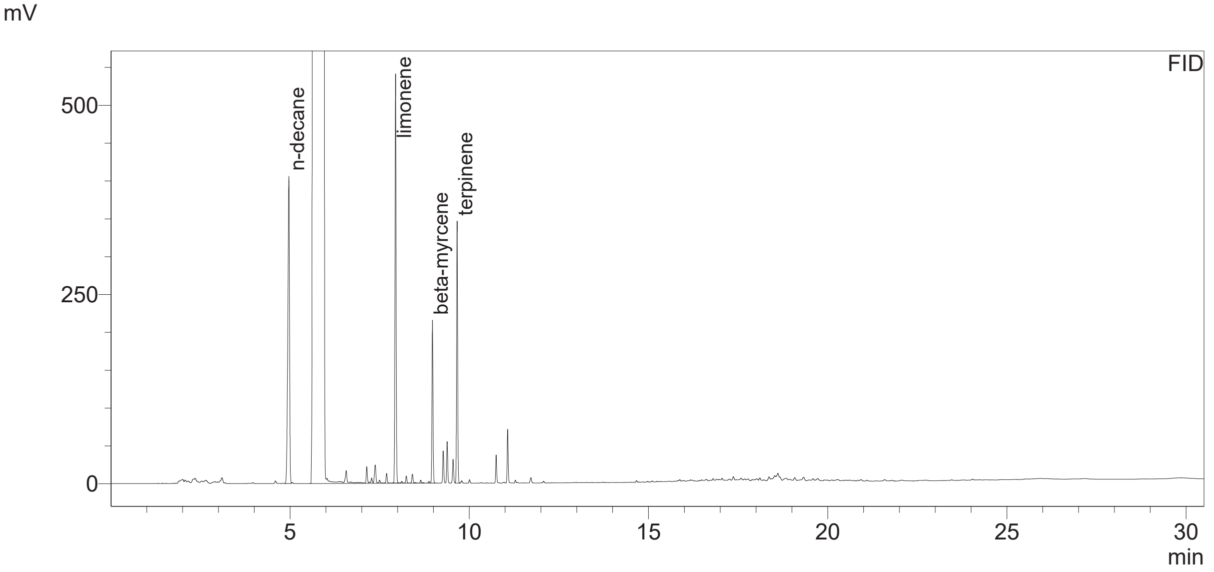

<Peak Table>

FID

Peak#	Ret. Time	Area	Height	Conc.	Unit	Mark	Name
1	4.964	1333216	404908	0.000	ppm	V	n-decane
2	5.915	353214112	37015762	0.000		S	
3	7.942	1133345	537655	-0.000	ppm	T	limonene
4	8.971	427375	212614	-0.000	ppm	TV	beta-myrcene
5	9.659	684562	343880	-0.000	ppm	V	terpinene
Total		356792610	38514819				

C:\LabSolutions\Data\Aurodeep\alcohol_dehydration\100C-reacn\AP-04-087-B-nerol-18h.gcd

Figure S10. Dehydration of gerianol (0.72M), 1% catalyst, toluene, 120 °C, 24 h

SHIMADZU LabSolutions

Analysis Report

<Sample Information>

Sample Name	: AP-03-056-20.5h		
Sample ID	: AP-03-056-20.5h		
Data Filename	: AP-03-056-20.5h.gcd		
Method Filename	: Alcohols (FID ONLY)--terpenols-28 min run.gcm		
Batch Filename	:		
Vial #	: 1	Sample Type	: Unknown
Injection Volume	: 1 uL		
Date Acquired	: 3/29/2023 12:45:46 PM	Acquired by	: wdjgroup
Date Processed	: 7/29/2024 12:37:55 PM	Processed by	: wdjgroup

<Chromatogram>

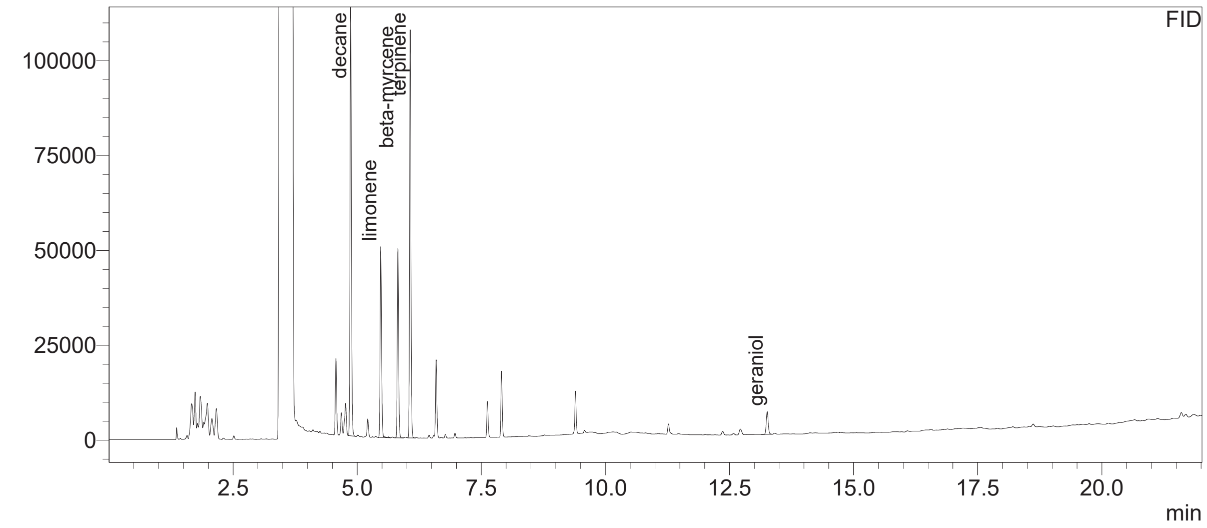

<Peak Table>

FID

Peak#	Ret. Time	Area	Height	Conc.	Unit	Mark	Name
1	4.868	216086	110803	-0.000	ppm		decane
2	5.475	100299	49223	-0.000	ppm	S	limonene
3	5.820	93207	49028	-0.000	ppm		beta-myrcene
4	6.067	197161	105103	-0.000	ppm	V	terpinene
5	13.260	16738	5892	-0.000	ppm		geraniol
Total		623491	320049				

C:\LabSolutions\Data\Aurodeep\alcohol_dehydration\100C-reacn\AP-03-056-20.5h.gcd

Figure S11. GC traces for dehydration of 1-phenylethanol at 0.83M by **2b** in toluene

SHIMADZU LabSolutions

Analysis Report

<Sample Information>

Sample Name	: AP-04-103A-24h		
Sample ID	: AP-04-103A-24h		
Data Filename	: AP-04-103A-24h.gcd		
Method Filename	: Alcohols (FID ONLY)-35 min run.gcm		
Batch Filename	:		
Vial #	: 1	Sample Type	: Unknown
Injection Volume	: 1 uL		
Date Acquired	: 9/10/2024 1:14:38 PM	Acquired by	: wdjgroup
Date Processed	: 9/10/2024 1:54:30 PM	Processed by	: wdjgroup

<Chromatogram>

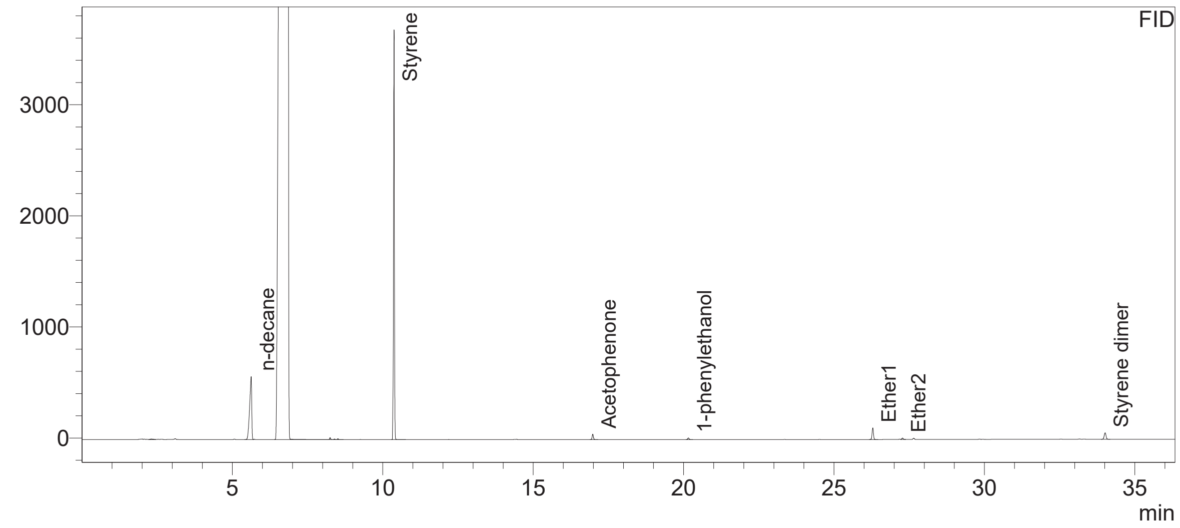

<Peak Table>

FID

Peak#	Ret. Time	Area	Height	Conc.	Unit	Mark	Name
1	2.313	47033	5424	0.000		V	
2	5.624	2731772	564918	0.000	ppm		n-decane
3	6.842	346978451	30085231	0.000		S	
4	10.373	7742784	3651851	-0.000	ppm		Styrene
5	16.980	141860	48932	-0.000	ppm		Acetophenone
6	20.159	46252	14682	-0.000	ppm		1-phenylethanol
7	26.290	337374	104948	-0.000	ppm		Ether1
8	27.277	42034	12663	-0.000	ppm	V	Ether2
9	34.013	245059	58016	-0.000	ppm		Styrene dimer
Total		358312619	34546665				

C:\LabSolutions\Data\Aurodeep\alcohol_dehydration\100C-reacn\AP-04-103A-24h.gcd

Figure S12. GC traces for dehydration of 1-phenylethanol at 0.83M by **2c** in toluene

SHIMADZU LabSolutions

Analysis Report

<Sample Information>

Sample Name : AP-04-103B-24h
Sample ID : AP-04-103B-24h
Data Filename : AP-04-103B-24h.gcd
Method Filename : Alcohols (FID ONLY)-35 min run.gcm
Batch Filename :
Vial # : 1
Sample Type : Unknown
Injection Volume : 1 uL
Date Acquired : 9/10/2024 11:51:10 AM
Acquired by : wdjgroup
Date Processed : 9/10/2024 1:22:23 PM
Processed by : wdjgroup

<Chromatogram>

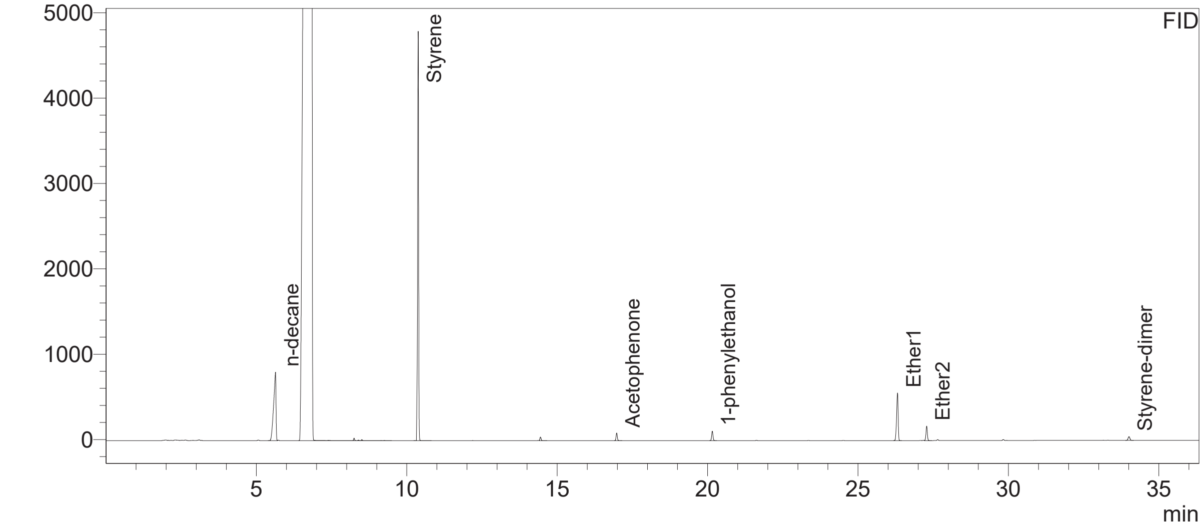

<Peak Table>

FID

Peak#	Ret. Time	Area	Height	Conc.	Unit	Mark	Name
1	5.634	4356700	801212	0.000	ppm		n-decane
2	6.833	331715493	29174370	0.000		S	
3	10.379	10822150	4716585	-0.000	ppm	SV	Styrene
4	14.443	120665	43117	0.000		V	
5	16.977	260526	90123	-0.000	ppm	V	Acetophenone
6	20.156	348827	109923	-0.000	ppm		1-phenylethanol
7	26.313	1990238	556578	-0.000	ppm	V	Ether1
8	27.282	534266	168734	-0.000	ppm	V	Ether2
9	34.008	184614	44484	-0.000	ppm	V	Styrene-dimer
Total		350333478	35705125				

C:\LabSolutions\Data\Aurodeep\alcohol_dehydration\100C-reacn\AP-04-103B-24h.gcd

Figure S13.1 1-phenylethanol dehydration by **2a** at 120°C in toluene-d_8

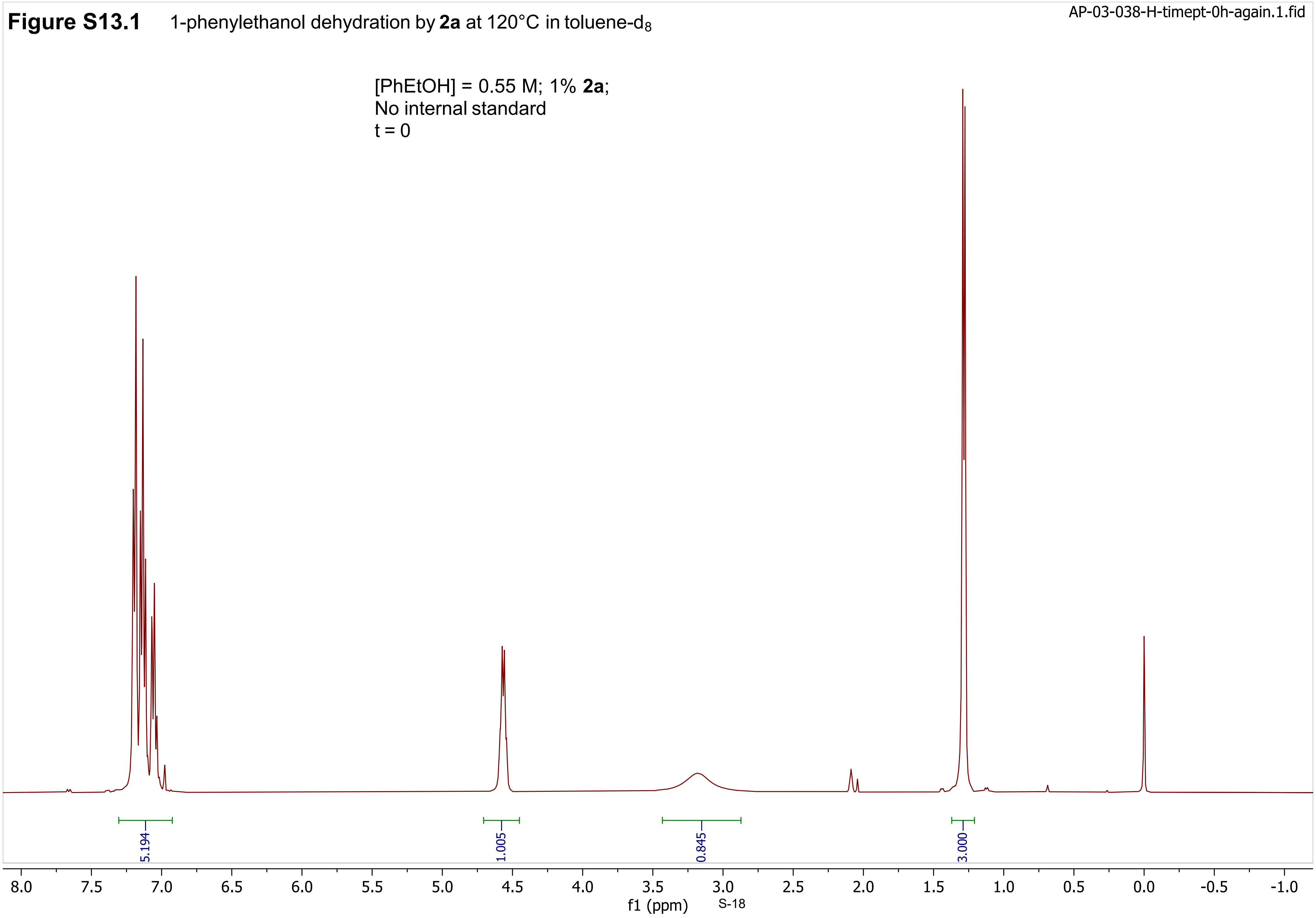

Figure S13.2 1-phenylethanol dehydration by **2a** at 120°C in toluene-d_8

[PhEtOH] = 0.55 M; 1% **2a**;
No internal standard
t = 1 h

A = 1-phenylethanol
S = styrene
E1 = ether1
E2 = ether2
SD = styrene dimer

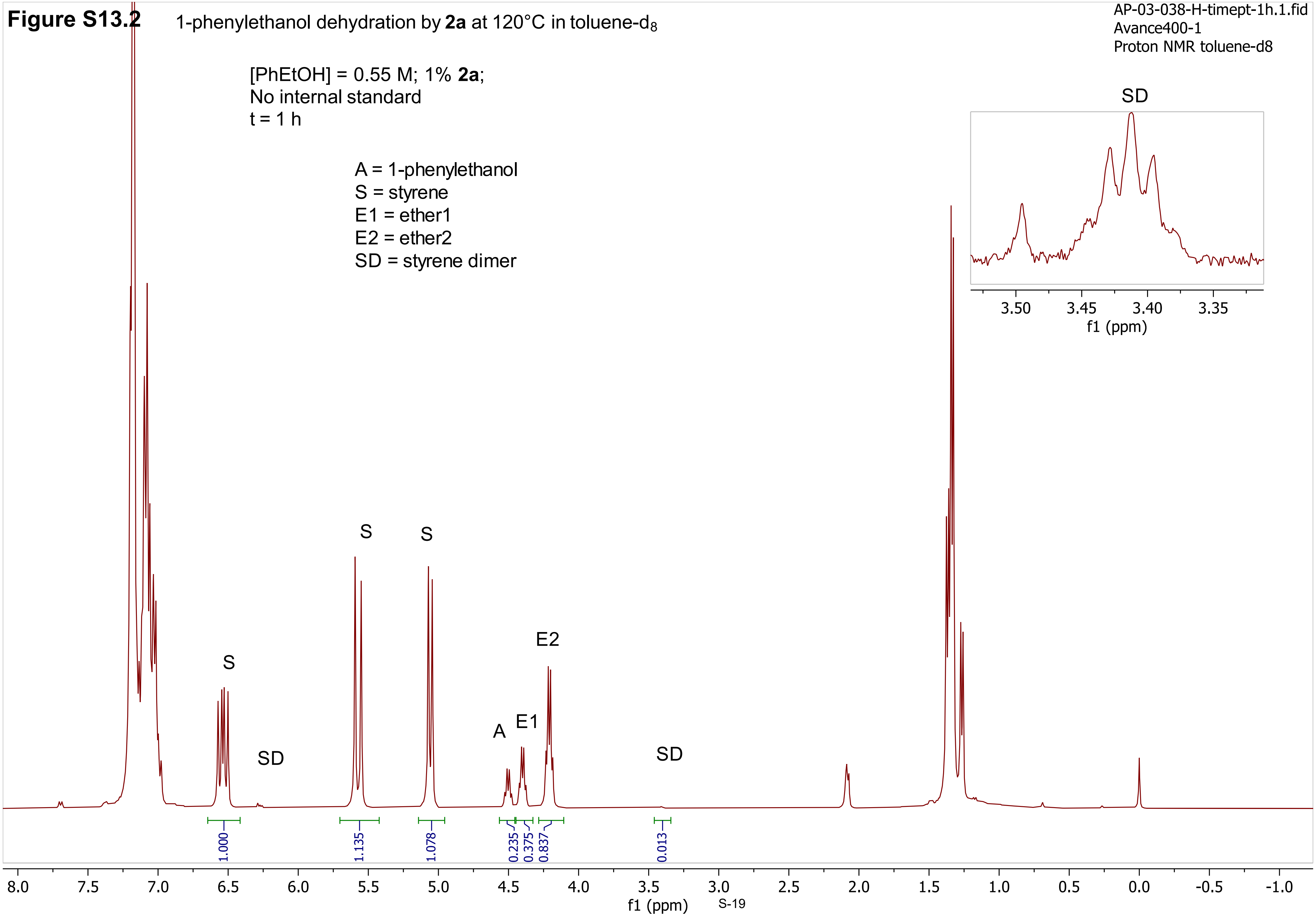

Figure S13.3 1-phenylethanol dehydration by **2a** at 120°C in toluene-d_8
[PhEtOH] = 0.55 M; 1% **2a**;
No internal standard
t = 18 h

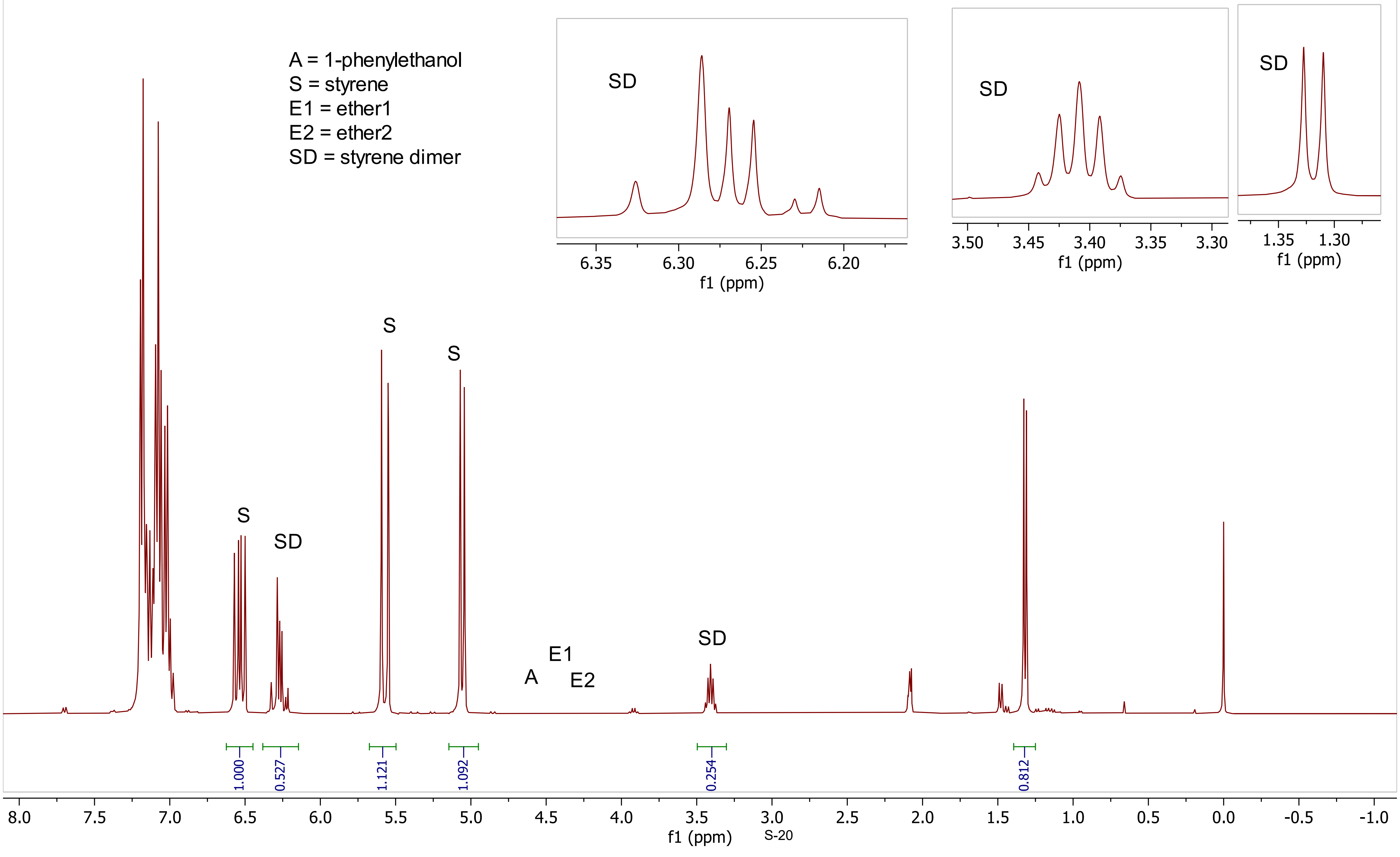

Figure S13.4 **Styrene dimer simulation (and ChemDraw prediction)**

$ABXY_3$ simulation: PhCH=CH-CHMePh

second order

J(AB) = J(AD) = 7 Hz

J(AC) = J(BD) = J(CD) = 0.01

J(BC) = 12 Hz

LB = 1.2 Hz

ChemNMR ^{1}H Estimation

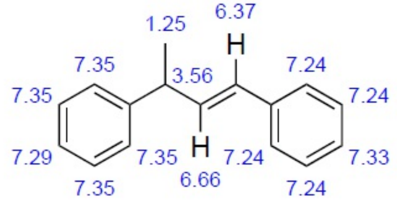

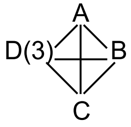

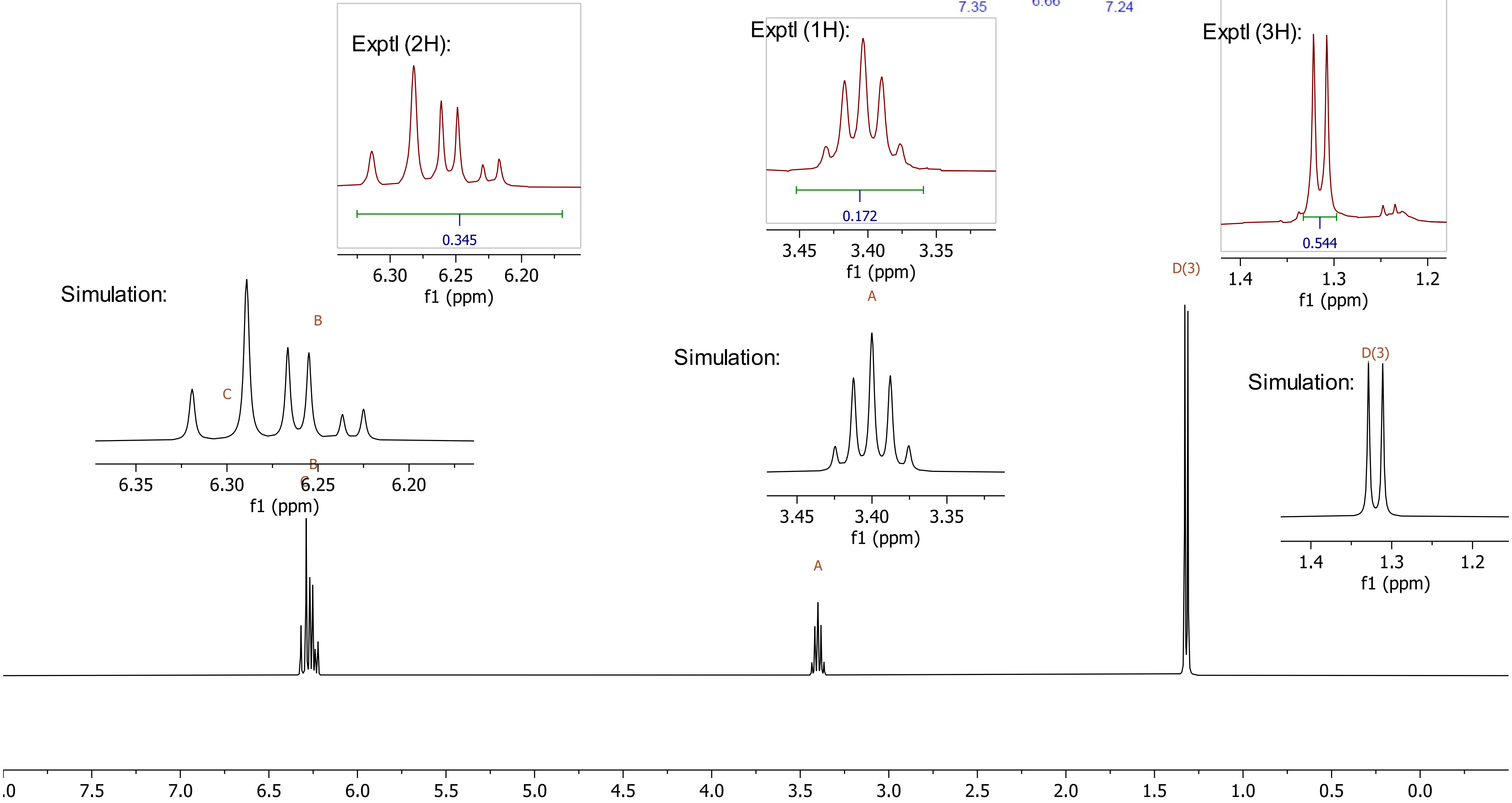

Figure S13.5

PhEtOH Dehydration - benchtop PhEtOH

AP-03-038-XXXppm

Reaction conditions: [1-PhEtOH] = 0.55 M (benchtop PhEtOH); toluene-d8; 1% catalyst **2a**; 120 °C.

J-Young NMR tube

time (h)	area phenylethanol(1H)	area styrene(1H)	area ether1(2H)	area ether2(2H)	area styrene-dimer(1H)	total weighted area
0	1.000	0.000	0.000	0.000	0.000	1.000
1	0.235	1.071	0.375	0.837	0.013	1.925
2	0.055	1.077	0.046	0.161	0.035	1.271
3	0.012	1.084	0.006	0.025	0.054	1.165
4	0.004	1.087	0.000	0.005	0.076	1.170
5	0.002	1.082	0.000	0.002	0.087	1.172
6	0.002	1.082	0.000	0.001	0.102	1.187
7	0.001	1.087	0.000	0.001	0.119	1.207
8	0.001	1.081	0.000	0.000	0.134	1.216
9	0.001	1.085	0.000	0.000	0.151	1.237
16	0.000	1.068	0.000	0.000	0.231	1.299
18	0.000	1.071	0.000	0.000	0.254	1.325
	δ 4.50	δ 6.54,5.56,5.05	δ 4.39	δ 4.21	δ 3.41	

time (h)	mol% phenylethanol	mol% styrene	mol% ether1	mol% ether2	mol% styrene-dimer	mol% total
0	100.000	0.000	0.000	0.000	0.000	100.000
1	12.208	55.636	9.740	21.740	0.675	100.000
2	4.329	84.770	1.810	6.336	2.755	100.000
3	1.030	93.005	0.257	1.073	4.635	100.000
4	0.342	92.948	0.000	0.214	6.497	100.000
5	0.171	92.321	0.000	0.085	7.423	100.000
6	0.169	91.193	0.000	0.042	8.597	100.000
7	0.083	90.018	0.000	0.041	9.858	100.000
8	0.082	88.901	0.000	0.000	11.017	100.000
9	0.081	87.712	0.000	0.000	12.207	100.000
16	0.000	82.222	0.000	0.000	17.778	100.000
18	0.000	80.830	0.000	0.000	19.170	100.000

Area $(PhEtOH)_0$ or styrene set to area 1.00.
mol% = area/#H/total area* 100%

Ethers are area 2H each; PhEtOH & styrene-dimer 1H each
styrene area = average of 3 vinylic peaks

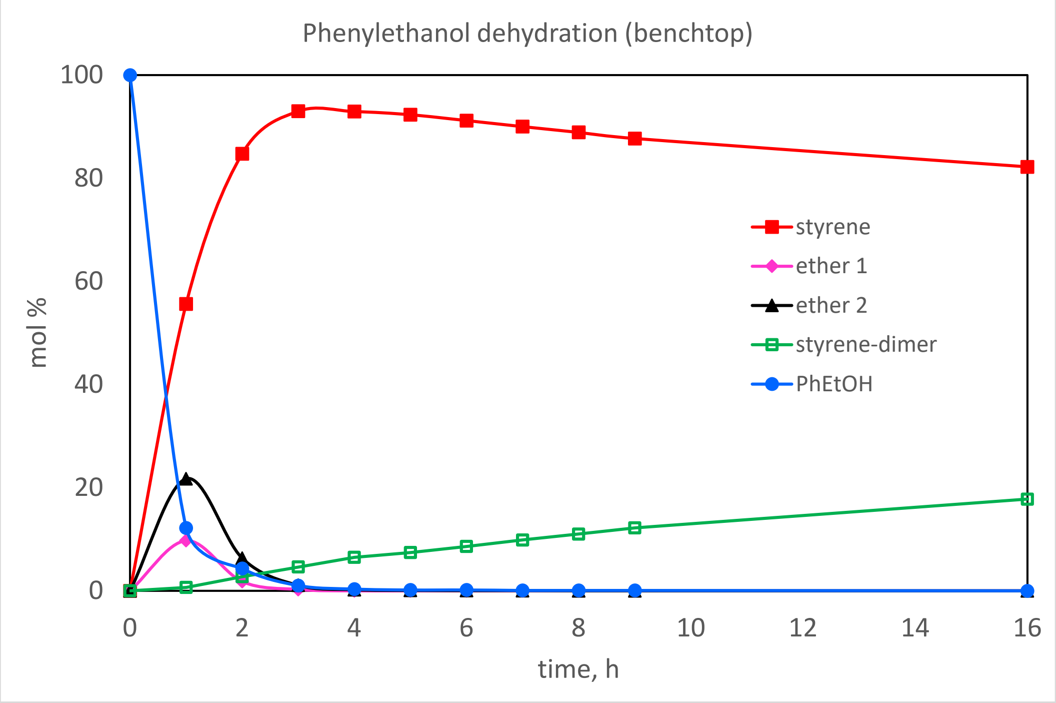

Figure S14.1 1-phenylethanol (300 ppm H_2O) dehydration by **2a** at 120°C in toluene-d_8

[PhEtOH] = 0.55 M; 1% **2a**;
TMS internal standard, sealed NMR tube
t = 0

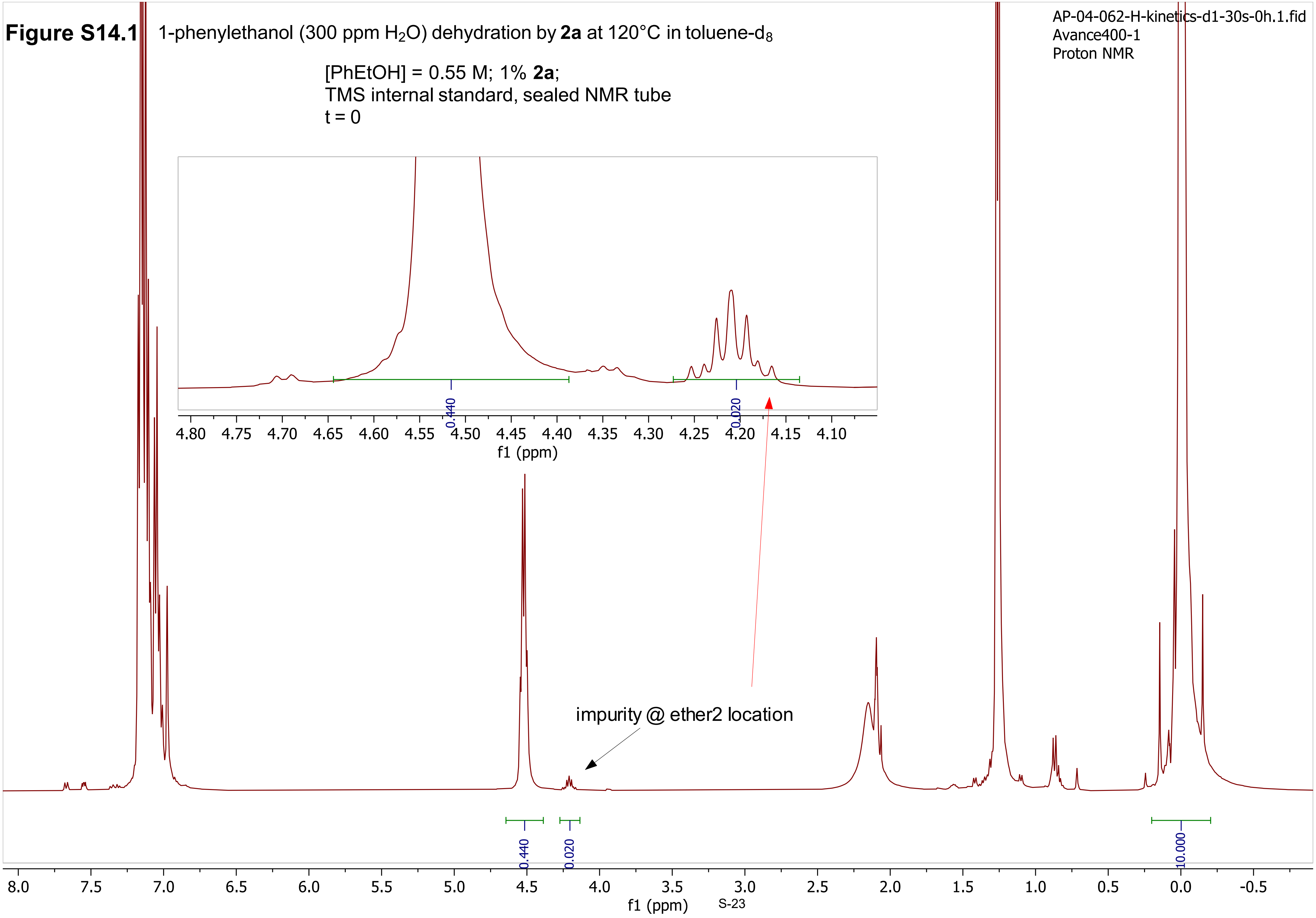

Figure S14.2

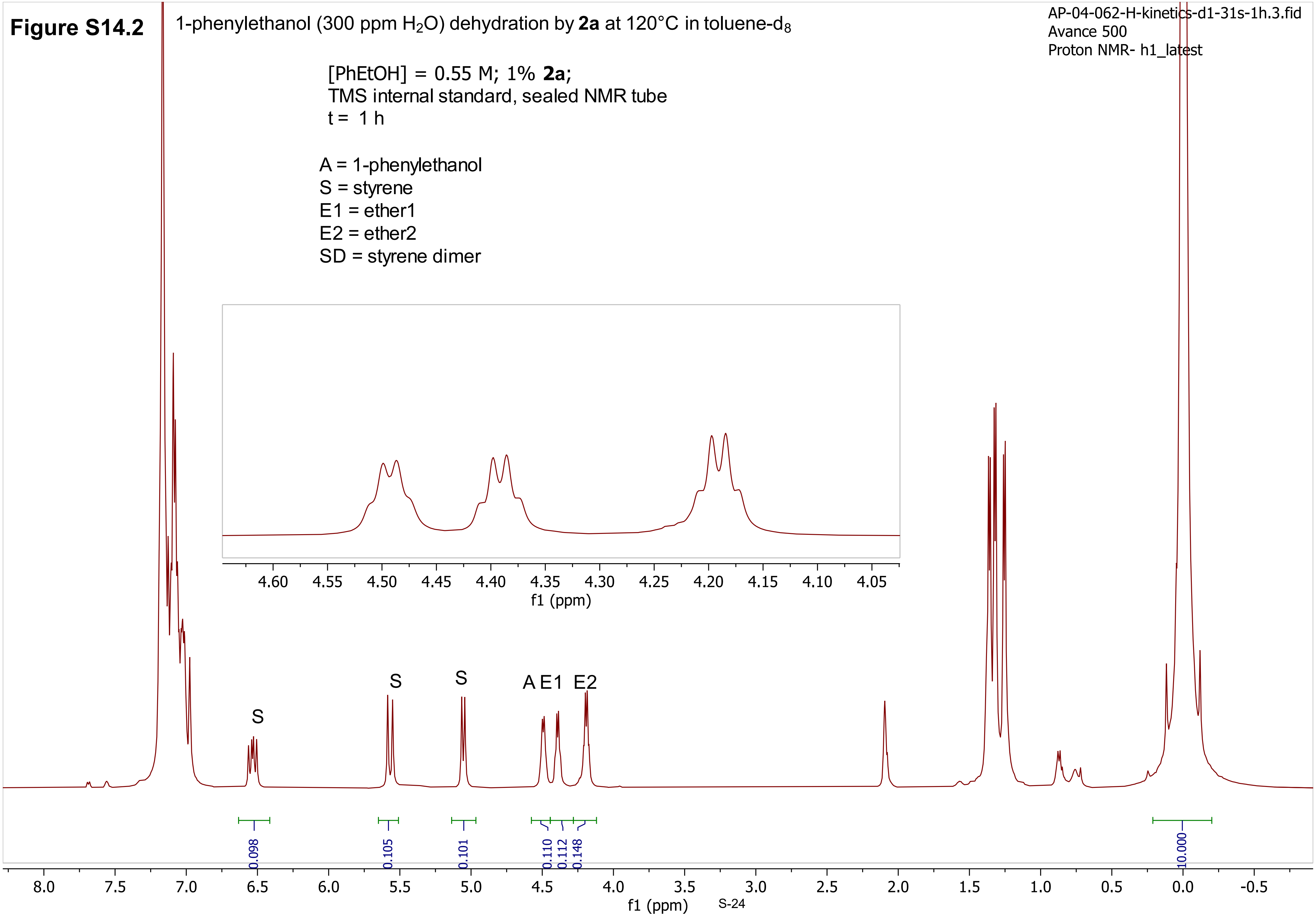
1-phenylethanol (300 ppm H_2O) dehydration by **2a** at 120°C in toluene-d_8
AP-04-062-H-kinetics-d1-31s-1h.3.fid
Avance 500
Proton NMR- h1_latest
[PhEtOH] = 0.55 M; 1% **2a**;
TMS internal standard, sealed NMR tube
t = 1 h
A = 1-phenylethanol
S = styrene
E1 = ether1
E2 = ether2
SD = styrene dimer
S
S
S
A E1 E2
0.098
0.105
0.101
0.110
0.112
0.148
10.000
f1 (ppm)

Figure S14.3 1-phenylethanol (300 ppm H_2O) dehydration by **2a** at 120°C in toluene-d_8

[PhEtOH] = 0.55 M; 1% **2a**;
TMS internal standard, sealed NMR tube
t = 16 h

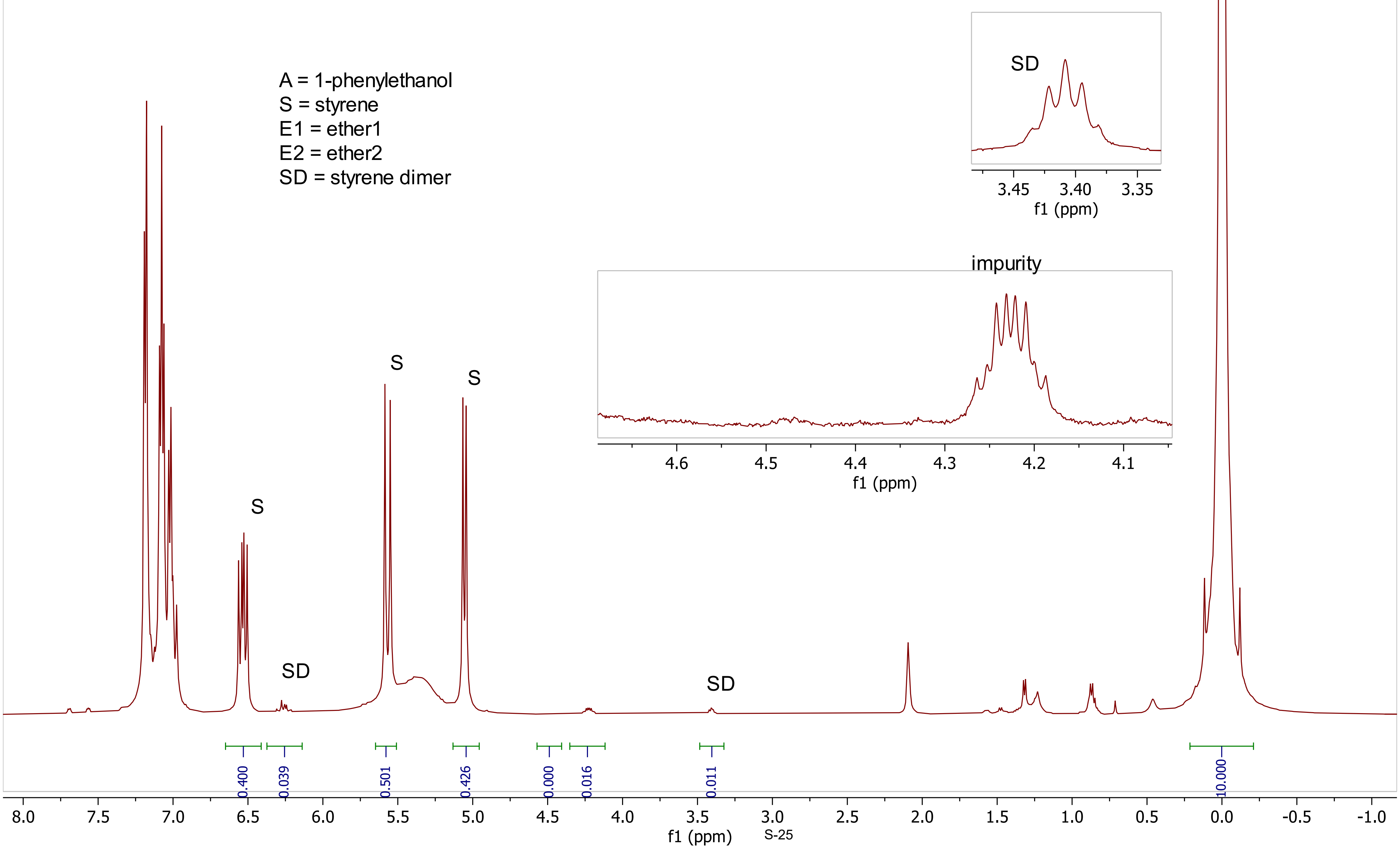

Figure S14.4

PhEtOH Dehydration - 300 ppm H_2O

AP-04-062-Hkinetics

Reaction conditions: [1-PhEtOH] = 0.55 M (contains 300 ppm H_2O); toluene-d_8; 1% catalyst **2a**; 120 °C.

Flame-sealed NMR tube

time (h)	area phenylethanol	area styrene	area ether1	area* ether2	area styrene-dimer	total weighed area
0	0.440	0.000	0.000	0.000	0.000	0.440
1	0.110	0.098	0.112	0.128	0.000	0.328
2	0.046	0.206	0.069	0.116	0.000	0.345
3	0.030	0.262	0.041	0.093	0.002	0.361
4	0.023	0.317	0.025	0.073	0.003	0.392
5	0.018	0.344	0.014	0.052	0.004	0.399
6	0.013	0.369	0.008	0.034	0.004	0.407
16	0.000	0.400	0.000	-0.004	0.011	0.409
	δ 4.50	δ 6.54	δ 4.39	δ 4.21	δ 3.41	

time (h)	mol% phenylethanol	mol% styrene	mol% ether1	mol% ether2	mol% styrene-dimer	mol% total
0	100.000	0.000	0.000	0.000	0.000	100.00
1	33.537	29.878	17.073	19.512	0.000	100.00
2	13.353	59.797	10.015	16.836	0.000	100.00
3	8.310	72.576	5.679	12.881	0.554	100.00
4	5.867	80.867	3.189	9.311	0.765	100.00
5	4.511	86.216	1.754	6.516	1.003	100.00
6	3.194	90.663	0.983	4.177	0.983	100.00
16	0.000	97.800	0.000	-0.489	2.689	100.00

*note: impurity at δ 4.21 area = .020; subtracted from ether2 peak

TMS internal standard set to area 10.00

Ethers are area 2H each; PhEtOH & styrene-dimer 1H each

mol% = area/#H/total weighted area * 100%

styrene area = 1H @ δ 6.54

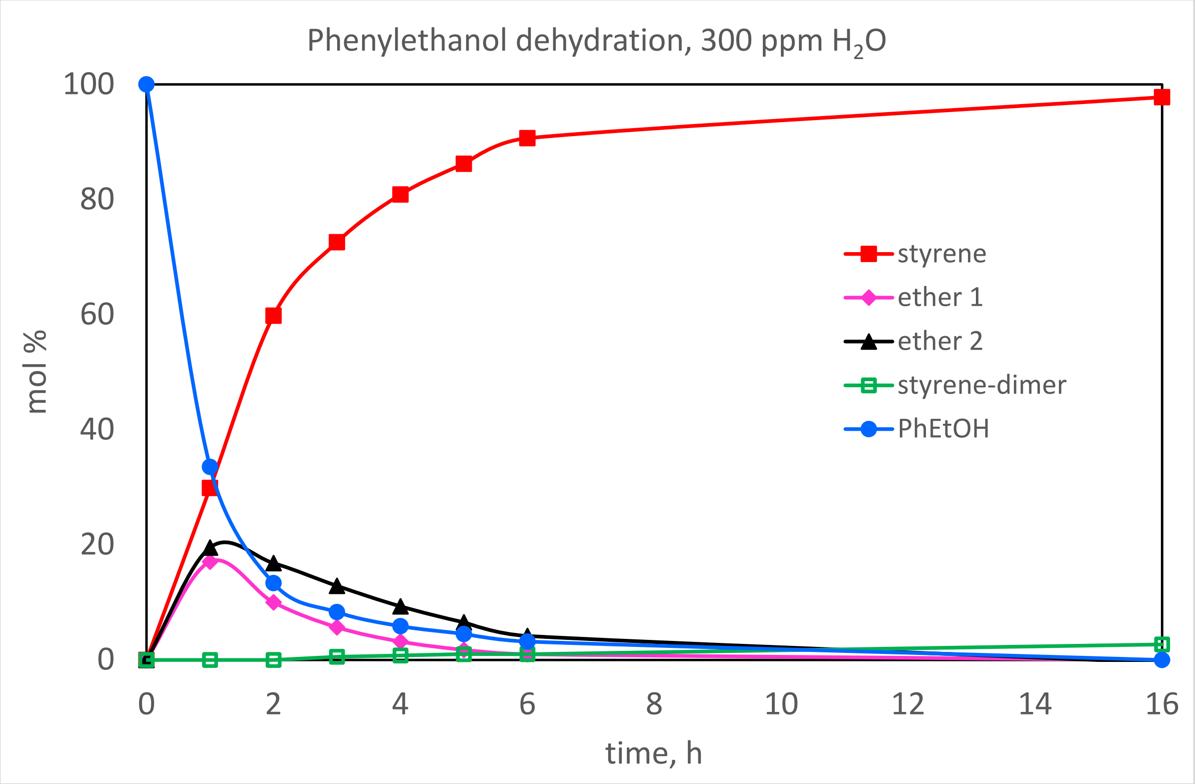

Figure S15.1 1-phenylethanol (600 ppm H_2O) dehydration by a**2** at 120°C in toluene-d_8

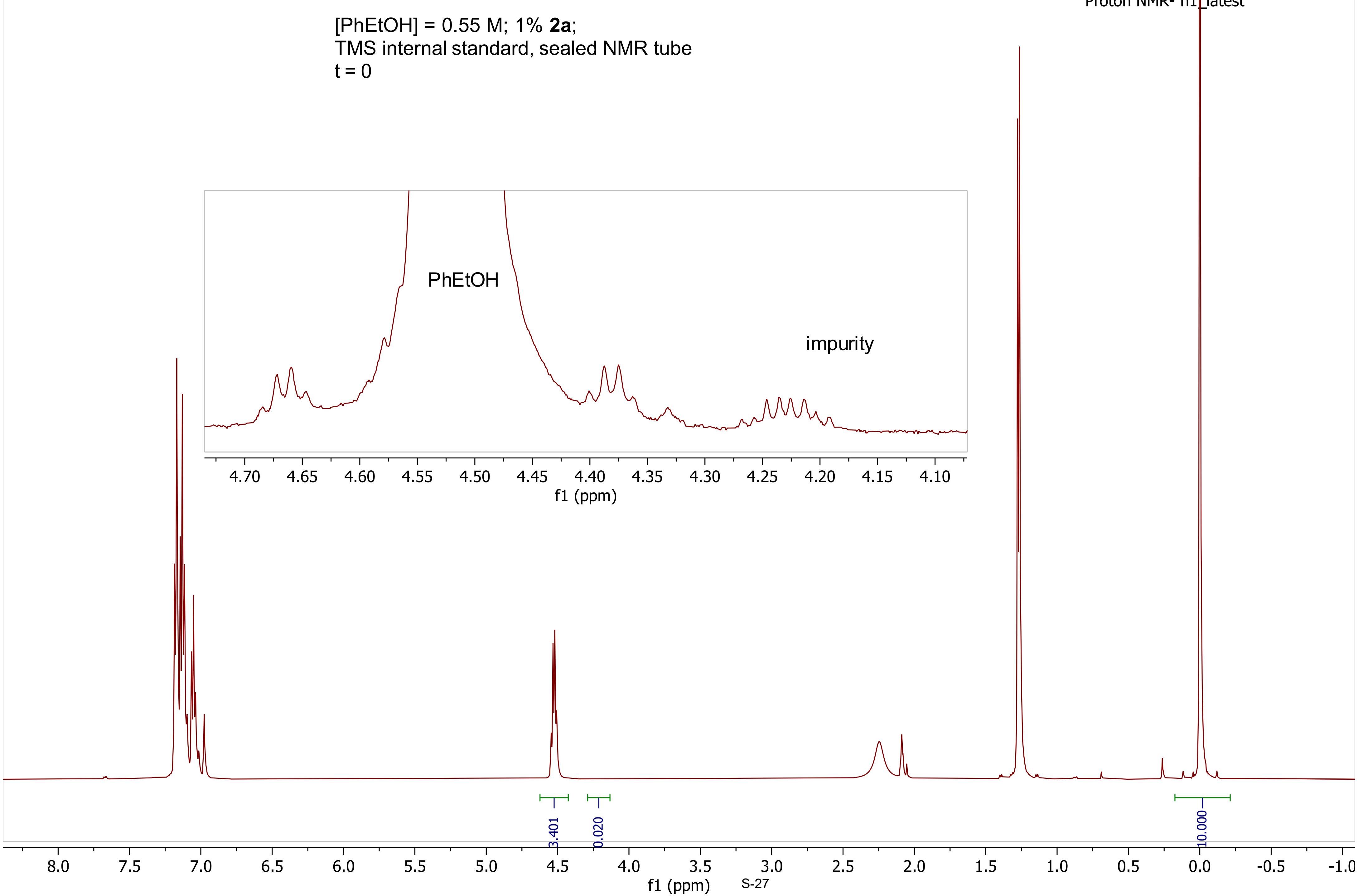

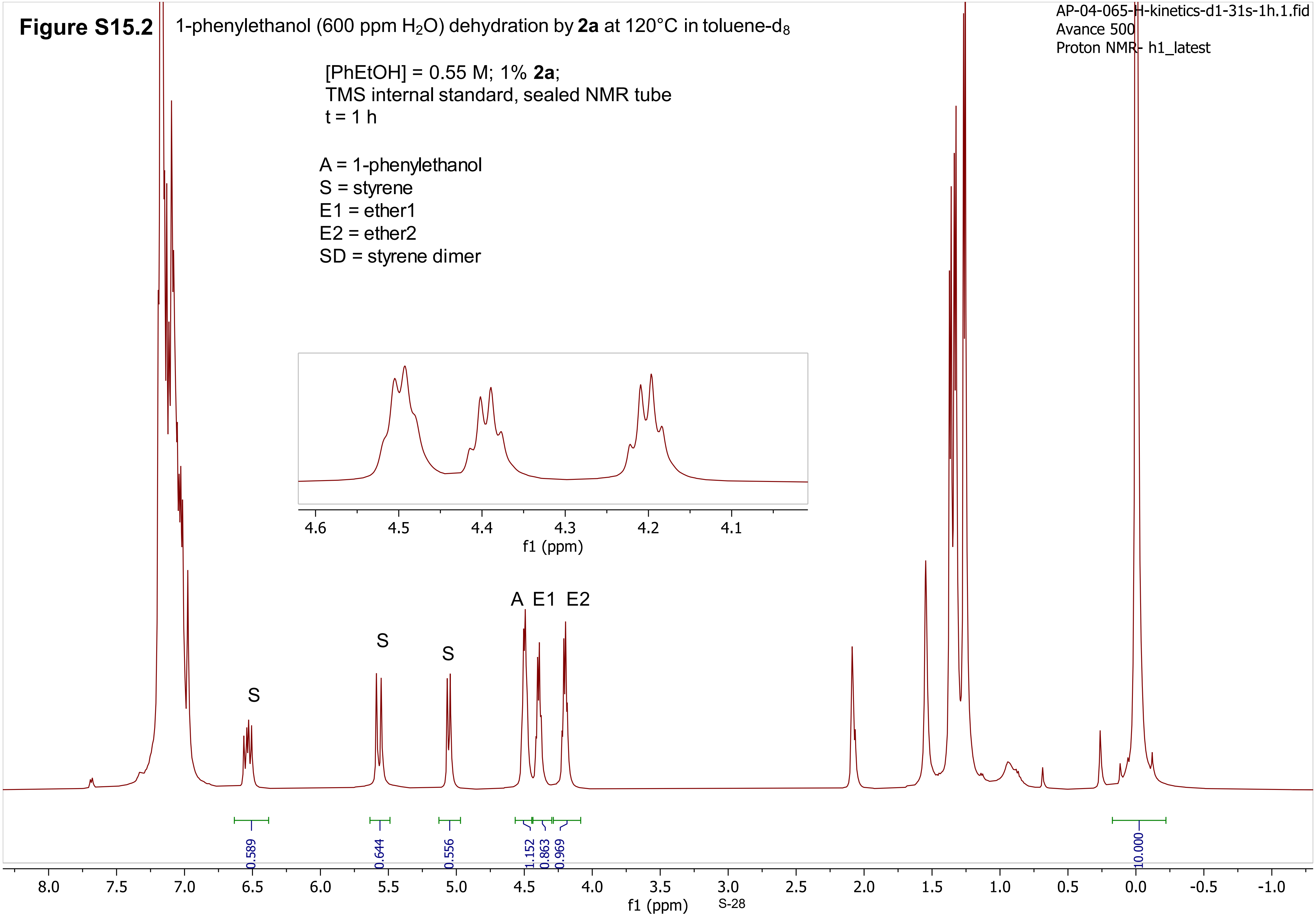

Figure S15.2
1-phenylethanol (600 ppm H_2O) dehydration by **2a** at 120°C in toluene-d_8
[PhEtOH] = 0.55 M; 1% **2a**;
TMS internal standard, sealed NMR tube
t = 1 h
A = 1-phenylethanol
S = styrene
E1 = ether1
E2 = ether2
SD = styrene dimer
AP-04-065-H-kinetics-d1-31s-1h.1.fid
Avance 500
Proton NMR- h1_latest
S
S
S
A
E1
E2
0.589
0.644
0.556
1.152
0.863
0.969
10.000
f1 (ppm)
f1 (ppm)

Figure S15.3 1-phenylethanol (600 ppm H_2O) dehydration by a**2** at 120°C in toluene-d_8

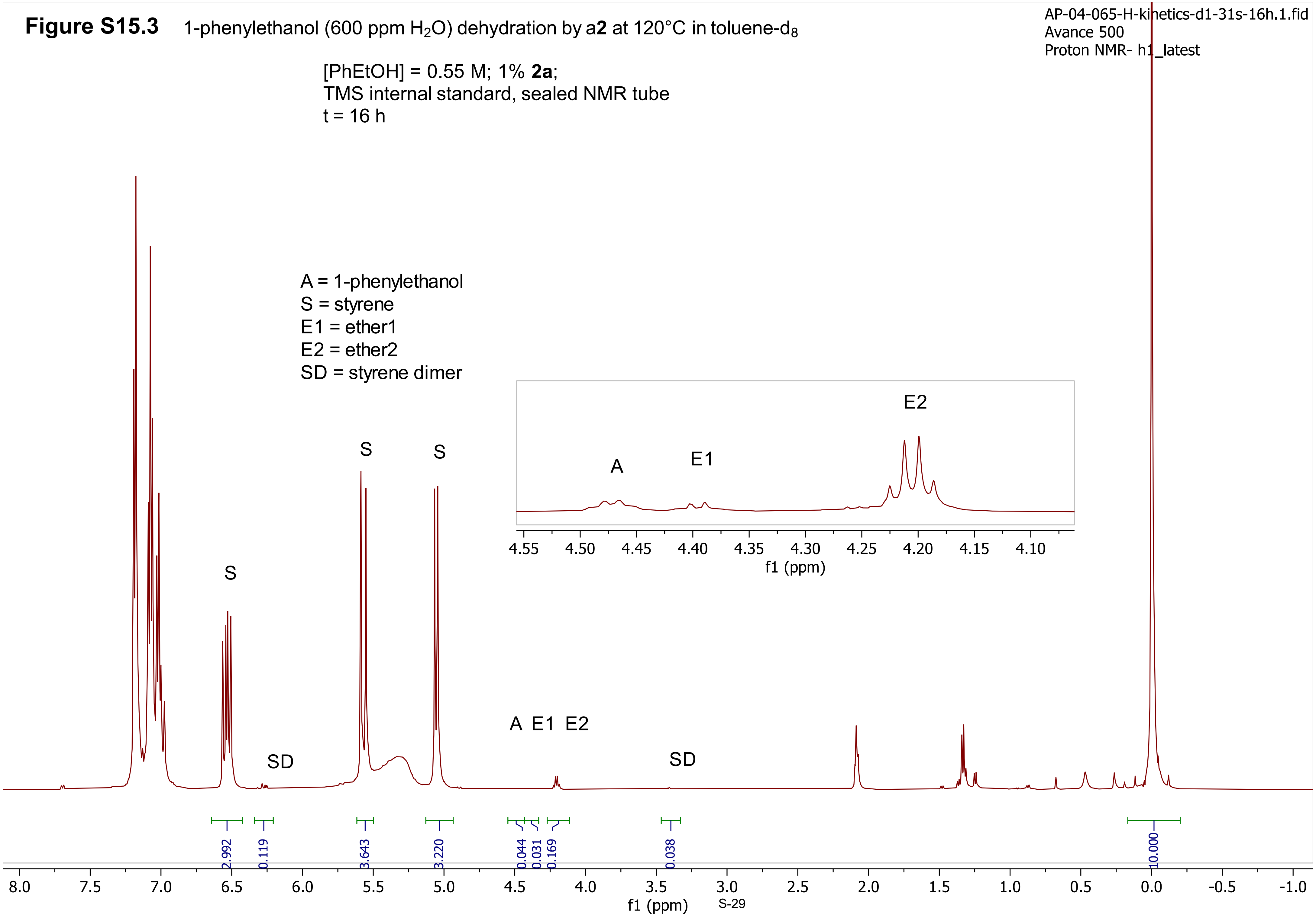

Figure S15.4

PhEtOH Dehydration - 600 ppm H_2O

AP-04-065-Hkinetics

Reaction conditions: [1-PhEtOH] = 0.55 M (contains 600 ppm H2O); toluene-d8; 1% catalyst **2a**; 120 °C.

Flame-sealed NMR tube

time (h)	area phenylethanol	area styrene	area ether1	area* ether 2	area styrene-dimer	total weighed area
0	3.401	0.000	0.000	0.000	0.000	3.401
1	1.152	0.589	0.863	0.949	0.000	2.647
2	0.655	0.995	0.787	1.021	0.000	2.554
3	0.528	1.161	0.736	1.042	0.000	2.578
4	0.455	1.243	0.655	0.993	0.000	2.522
16	0.044	2.992	0.031	0.149	0.119	3.245

time (h)	% phenylethanol	% styrene	% ether1	% ether 2	% styrene-dimer	mol% total
0	100.000	0.000	0.000	0.000	0.000	100.00
1	43.521	22.252	16.301	17.926	0.000	100.00
2	25.646	38.958	15.407	19.988	0.000	100.00
3	20.481	45.035	14.275	20.209	0.000	100.00
4	18.041	49.286	12.986	19.687	0.000	100.00
16	1.356	92.203	0.478	2.296	3.667	100.00

*note: impurity at δ 4.21 area = .020; subtracted from ether2 peak

TMS internal standard set to area 10.00

Ethers are area 2H each; PhEtOH & styrene-dimer 1H each

mol% = area/#H/total weighted area * 100%

styrene area = 1H @ δ 6.54

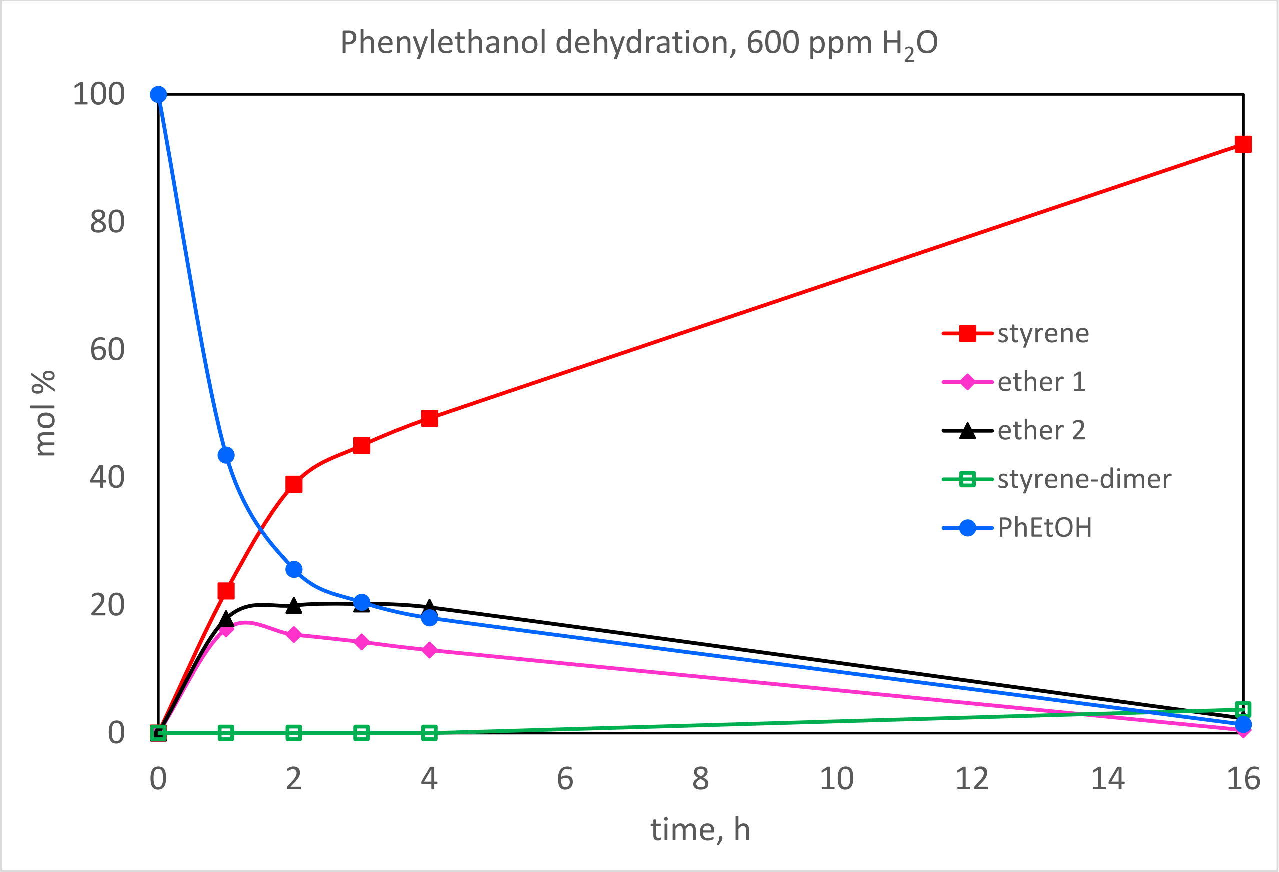

Figure S16.1 1-phenylethanol (600 ppm H_2O run#2) dehydration by a**2** at 120°C in toluene-d_8

[PhEtOH] = 0.55 M; 1% **2a**;
TMS internal standard, sealed NMR tube
t = 0 h

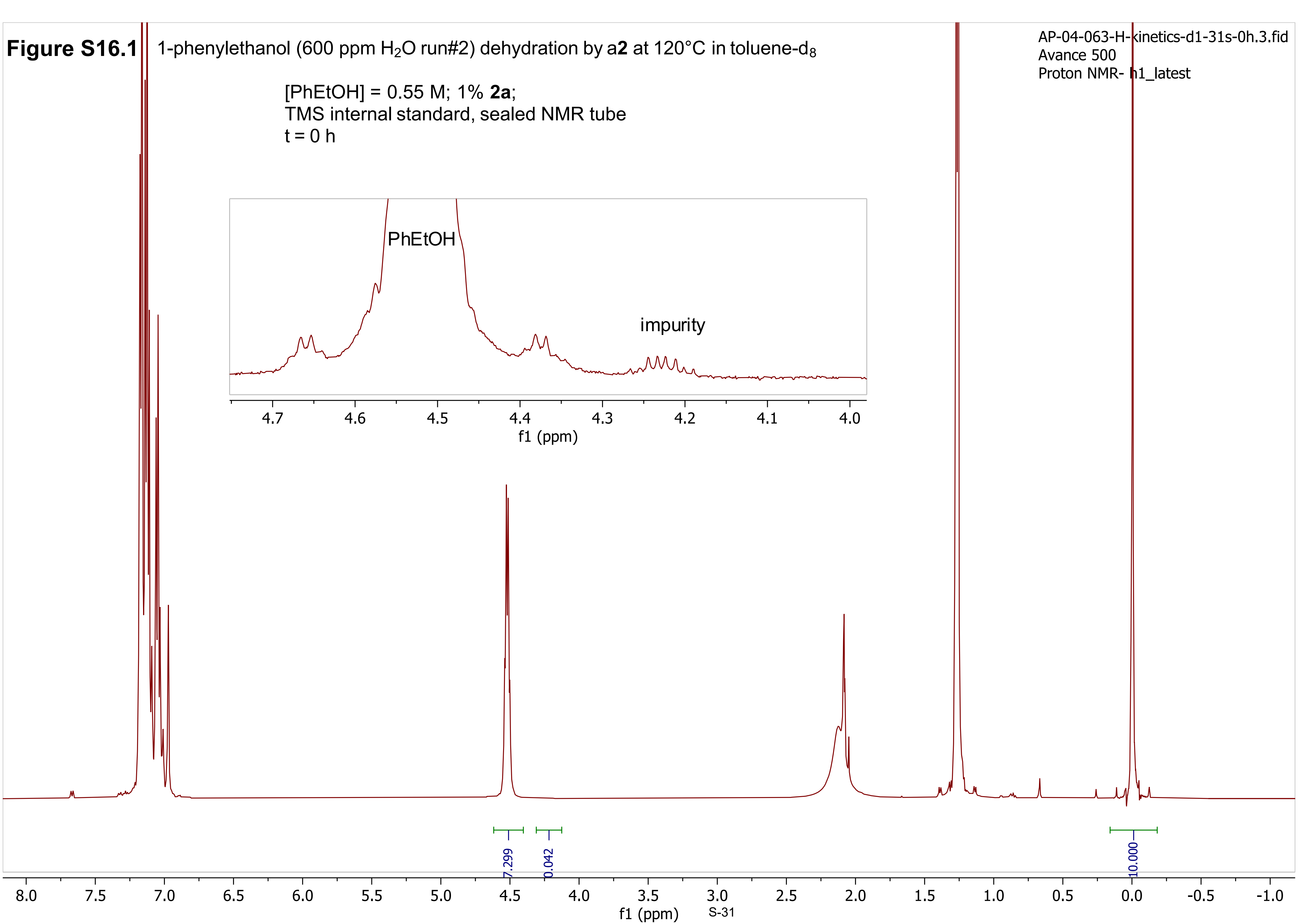

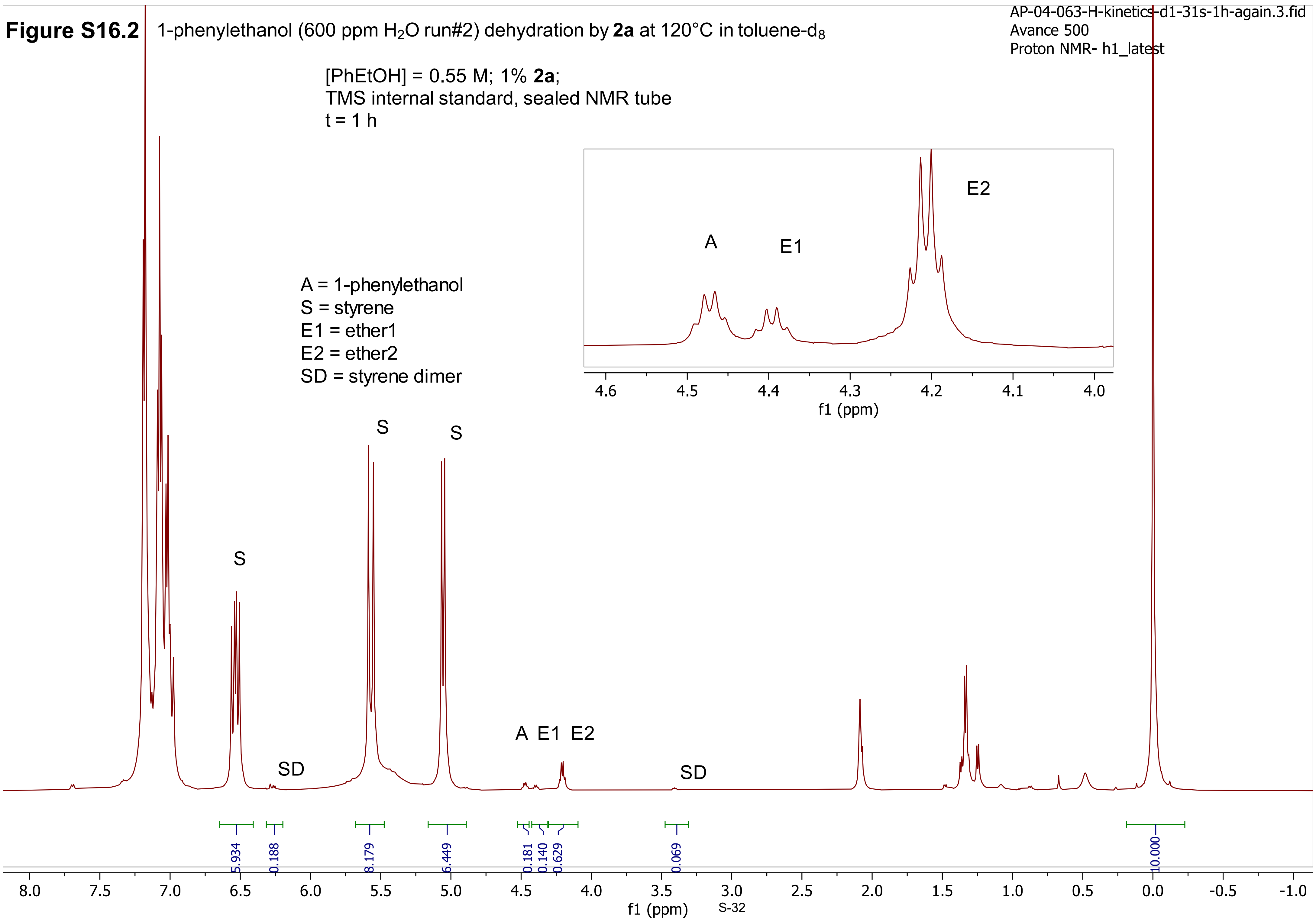

Figure S16.2 1-phenylethanol (600 ppm H2O run#2) dehydration by 2a at 120°C in toluene-d8
AP-04-063-H-kinetics-d1-31s-1h-again.3.fid
Avance 500
Proton NMR- h1_latest
[PhEtOH] = 0.55 M; 1% 2a;
TMS internal standard, sealed NMR tube
t = 1 h
A = 1-phenylethanol
S = styrene
E1 = ether1
E2 = ether2
SD = styrene dimer
A
E1
E2
4.6
4.5
4.4
4.3
4.2
4.1
4.0
f1 (ppm)
S
S
S
SD
A E1 E2
SD
5.934
0.188
8.179
6.449
0.181
0.140
0.629
0.069
10.000
8.0
7.5
7.0
6.5
6.0
5.5
5.0
4.5
4.0
3.5
3.0
2.5
2.0
1.5
1.0
0.5
0.0
-0.5
-1.0
f1 (ppm)

Figure S16.3 1-phenylethanol (600 ppm H_2O run#2) dehydration by **2a** at 120°C in toluene-d_8

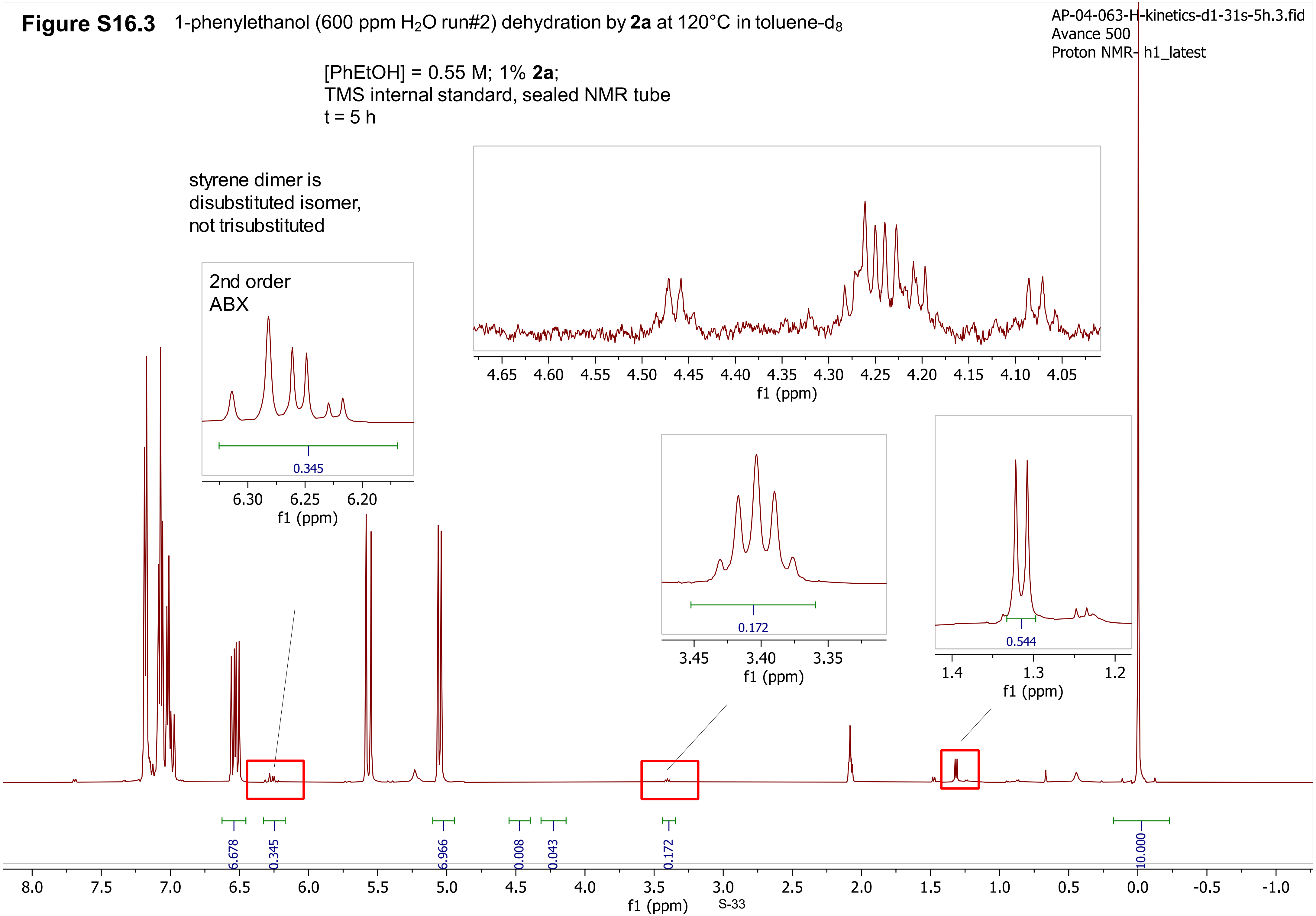

Figure S16.4

PhEtOH Dehydration - 600 ppm H_2O (run#2)

AP-04-063-Hkinetics

Reaction conditions: [1-PhEtOH] = 0.55 M (contains 600 ppm H2O); toluene-d8; 1% catalyst **2a**; 120 °C. Flame-sealed NMR tube

time (h)	area phenylethanol	area styrene	area ether1	area* ether 2	area styrene-dimer	total weighed area
0	7.299	0.000	0.000	0.000	0.000	7.299
1	0.181	5.934	0.140	0.587	0.069	6.548
2	0.029	6.509	0.013	0.111	0.126	6.726
3	0.015	6.703	0.005	0.029	0.144	6.879
4	0.006	6.780	0.000	0.005	0.169	6.958
5	0.008	6.678	0.000	0.001	0.172	6.859

time (h)	% phenylethanol	% styrene	% ether1	% ether 2	% styrene-dimer	mol% total
0	100.000	0.000	0.000	0.000	0.000	100.000
1	2.764	90.630	1.069	4.483	1.054	100.000
2	0.431	96.774	0.097	0.825	1.873	100.000
3	0.218	97.441	0.036	0.211	2.093	100.000
4	0.086	97.449	0.000	0.036	2.429	100.000
5	0.117	97.368	0.000	0.007	2.508	100.000

*note: impurity at δ 4.21 area = .020; subtracted from ether2 peak

TMS internal standard set to area 10.00

Ethers are area 2H each; PhEtOH & styrene-dimer 1H each

mol% = area/#H/total weighted area * 100%

styrene area = 1H @ δ 6.54

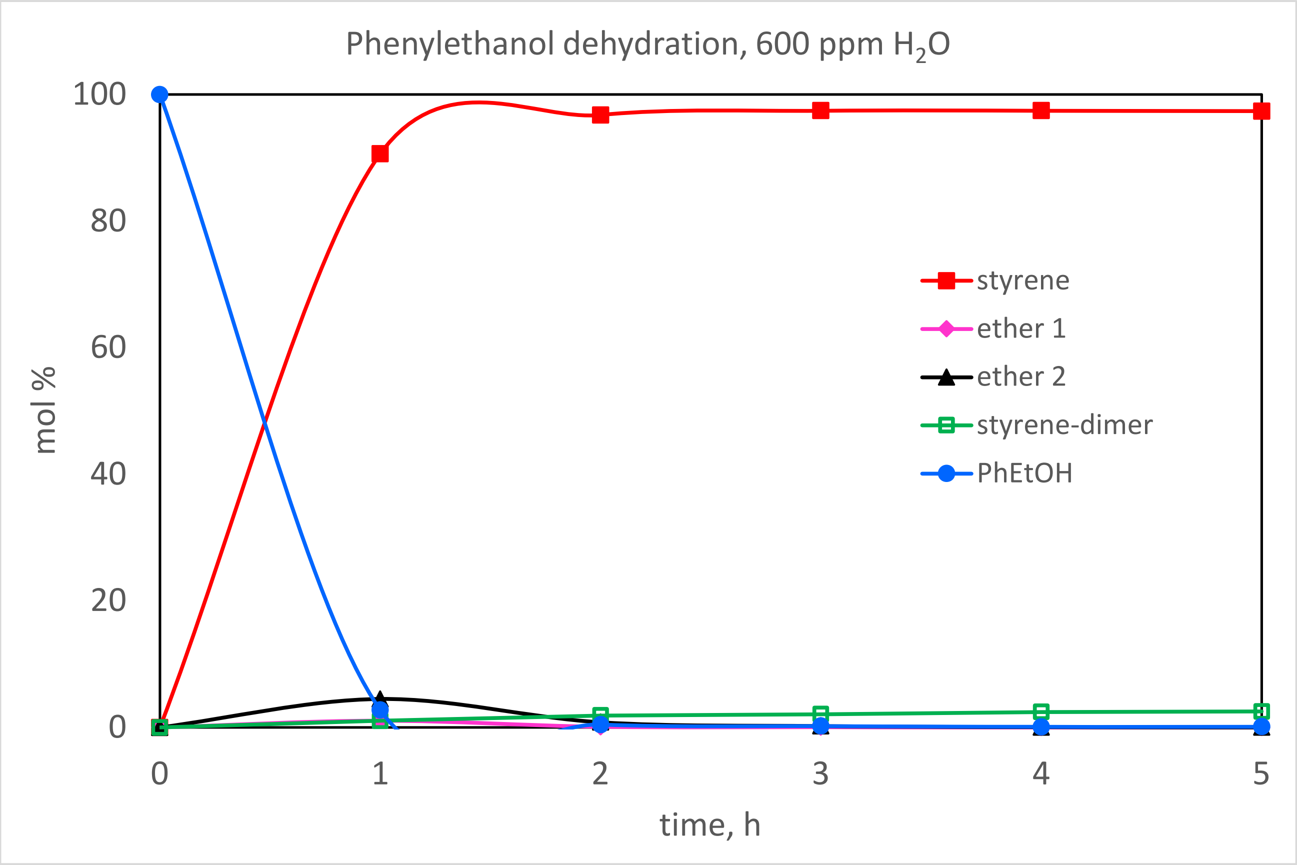

Figure S17.1. 1-phenylethanol (1000 ppm H_2O) dehydration by **2a** at 120°C in toluene-d_8
[PhEtOH] = 0.55 M; 1% **2a**;
TMS internal standard, sealed NMR tube
t = 0 h

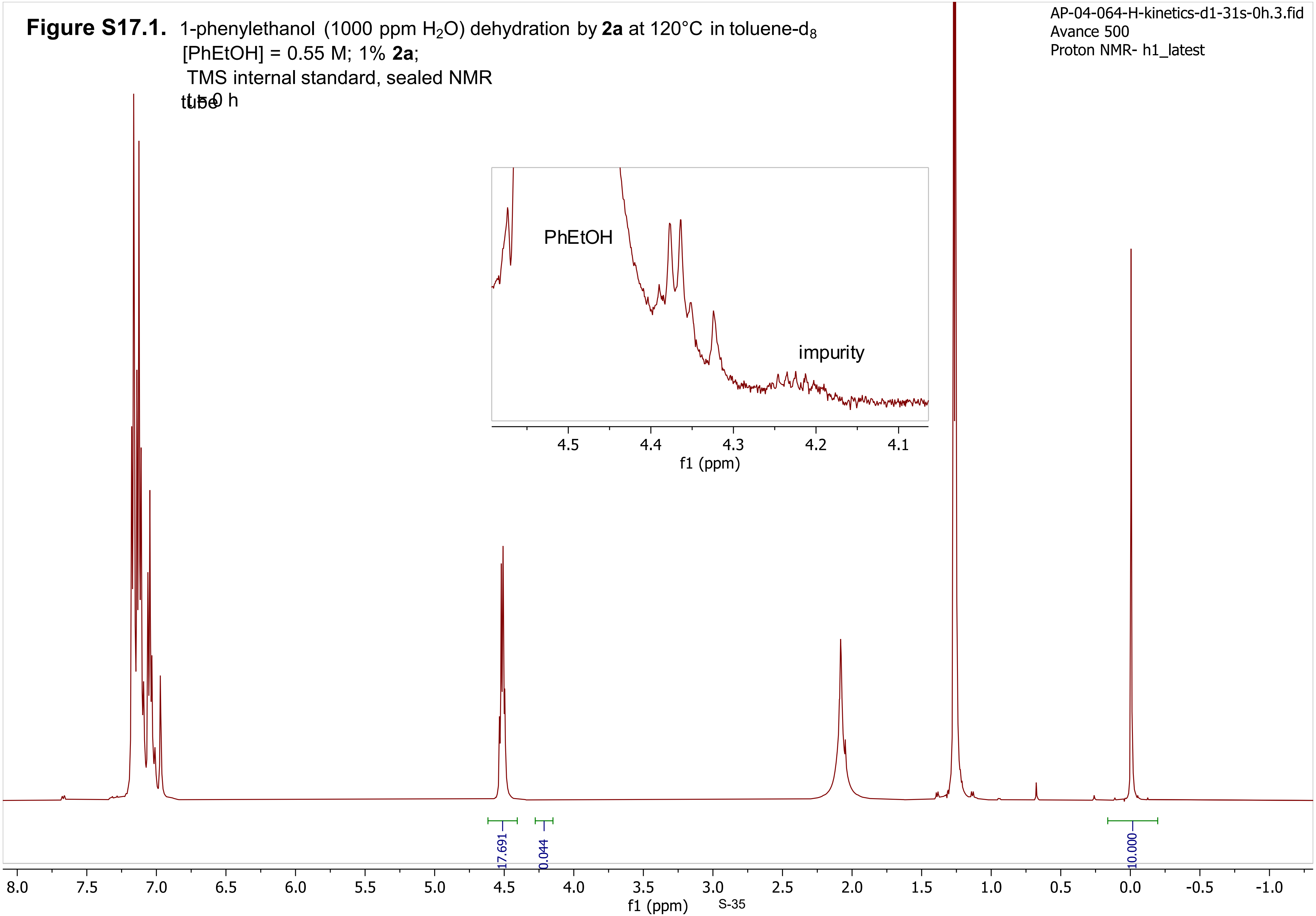

Figure S17.2

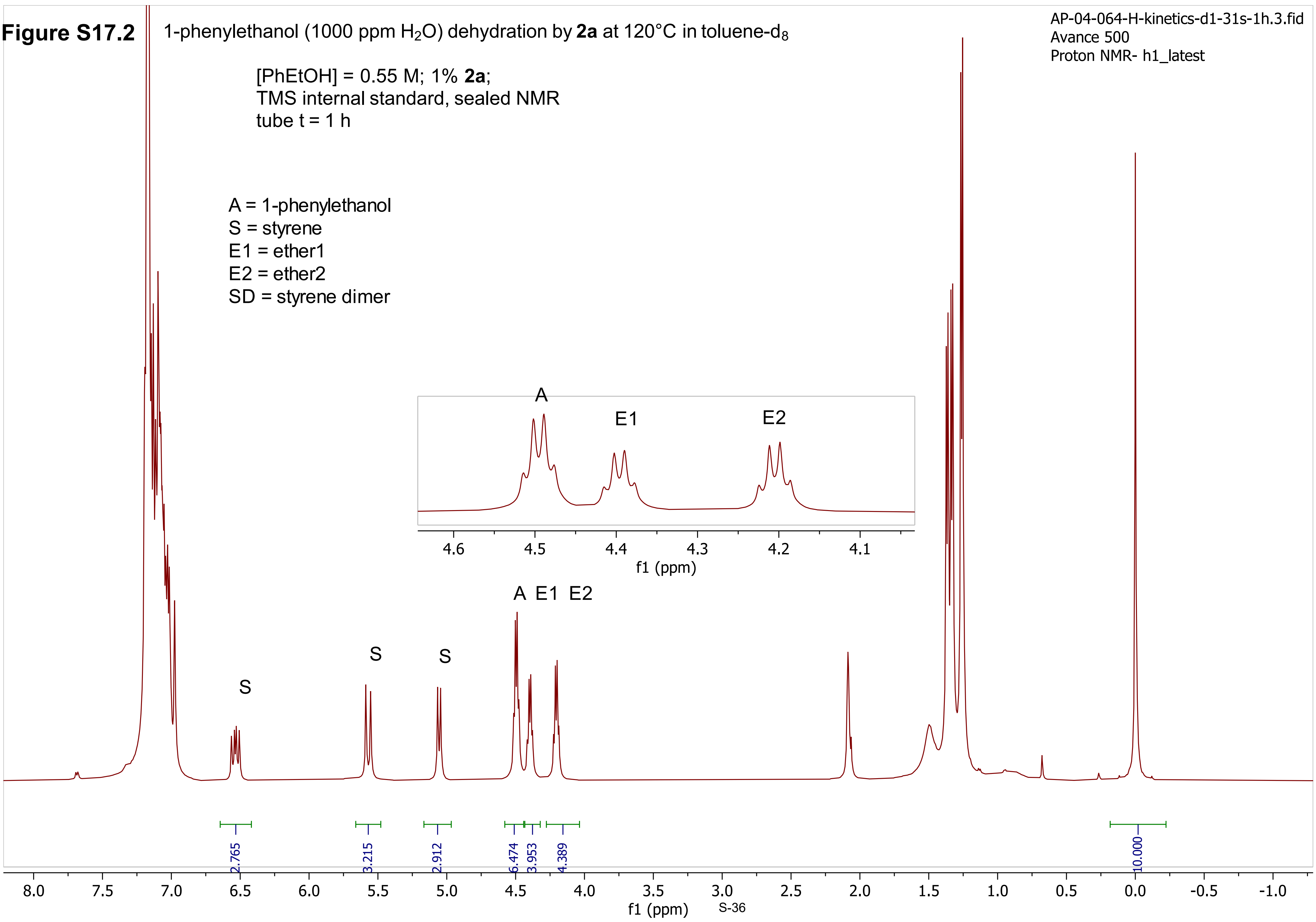
1-phenylethanol (1000 ppm H$_2$O) dehydration by **2a** at 120°C in toluene-d$_8$
AP-04-064-H-kinetics-d1-31s-1h.3.fid
Avance 500
Proton NMR- h1_latest
[PhEtOH] = 0.55 M; 1% **2a**;
TMS internal standard, sealed NMR
tube t = 1 h
A = 1-phenylethanol
S = styrene
E1 = ether1
E2 = ether2
SD = styrene dimer
A
E1
E2
4.6 4.5 4.4 4.3 4.2 4.1
f1 (ppm)
S S S A E1 E2
2.765 3.215 2.912 6.474 3.953 4.389 10.000
8.0 7.5 7.0 6.5 6.0 5.5 5.0 4.5 4.0 3.5 3.0 2.5 2.0 1.5 1.0 0.5 0.0 -0.5 -1.0
f1 (ppm)

Figure S17.3 1-phenylethanol (1000 ppm H_2O) dehydration by a**2** at 120°C in toluene-d_8

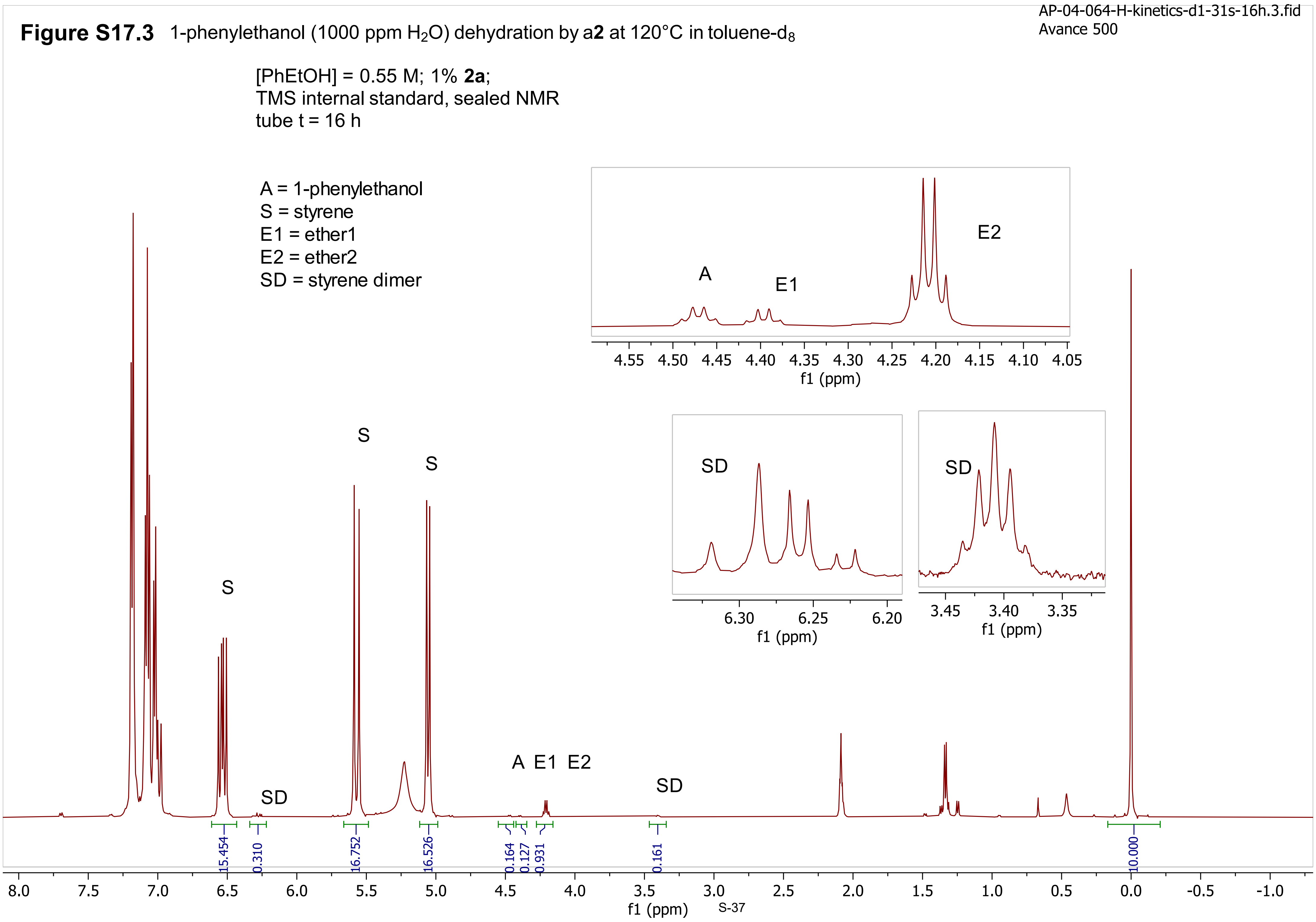

Figure S17.4

PhEtOH Dehydration - 1000 ppm H_2O

AP-04-064-Hkinetics

Reaction conditions: [1-PhEtOH] = 0.55 M (contains 1000 ppm H2O); toluene-d8; 1% catalyst **2a**; 120 °C.

Flame-sealed NMR tube

time (h)	area phenylethanol	area styrene	area ether1	area* ether 2	area styrene-dimer	total weighed area
0	17.691	0.000	0.000	0.000	0.000	17.691
1	6.474	2.765	3.953	4.345	0.000	13.388
2	2.603	7.670	3.753	5.047	0.000	14.673
3	1.751	9.363	2.843	4.734	0.039	14.942
4	1.347	10.427	2.213	4.179	0.046	15.016
5	1.143	11.613	1.912	3.915	0.056	15.726
16	0.164	15.454	0.127	0.887	0.161	16.286

time (h)	mol % phenylethanol	mol % styrene	mol % ether1	mol % ether 2	mol % styrene-dimer	mol% total
0	100.000	0.000	0.000	0.000	0.000	100.00
1	48.357	20.653	14.763	16.227	0.000	100.00
2	17.740	52.273	12.789	17.198	0.000	100.00
3	11.719	62.664	9.514	15.842	0.261	100.00
4	8.970	69.439	7.369	13.915	0.306	100.00
5	7.268	73.848	6.079	12.448	0.356	100.00
16	1.007	94.891	0.390	2.723	0.989	100.00

*note: impurity at δ 4.21 area = .044; subtracted from ether2 peak

TMS internal standard set to area 10.00

Ethers are area 2H each; PhEtOH & styrene-dimer 1H each

mol% = area/#H/total weighted area * 100%

styrene area = 1H @ δ 6.54

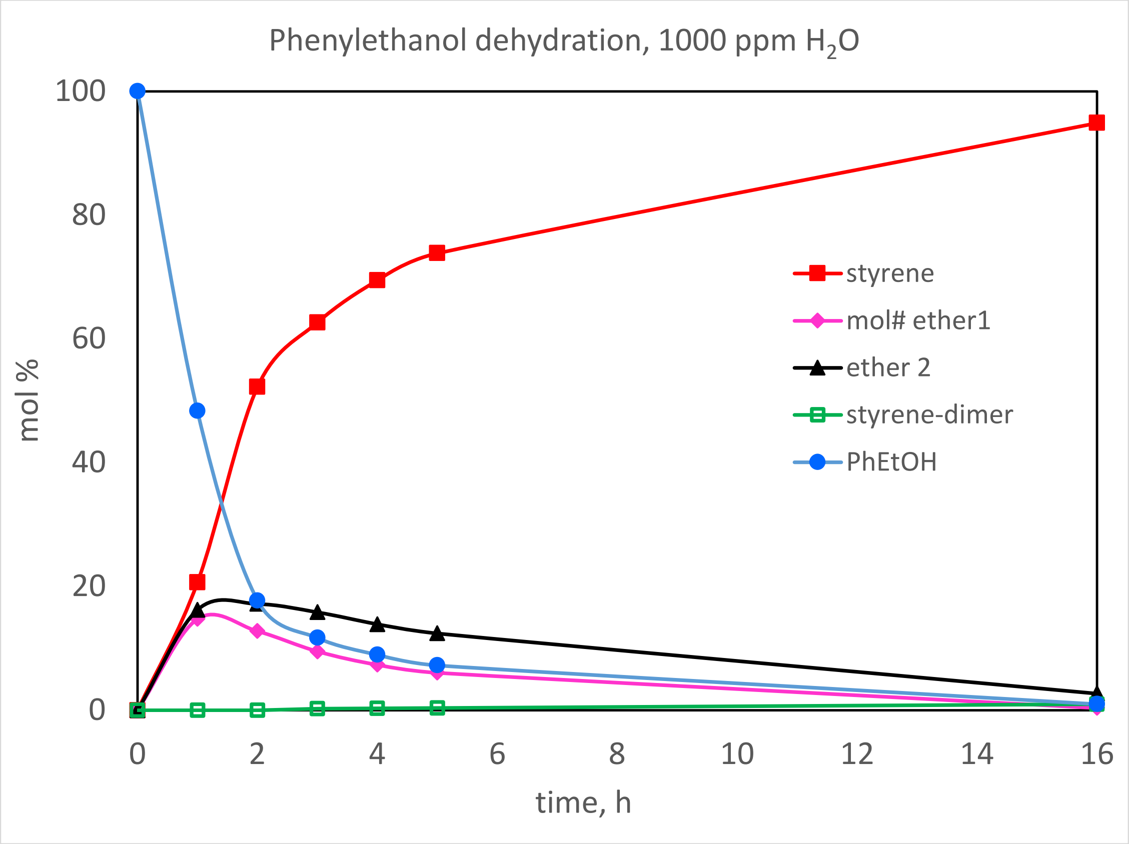

Figure S18.1 1-(p-CF_3phenyl)ethanol dehydration by **2a** at 120°C in toluene-d_8

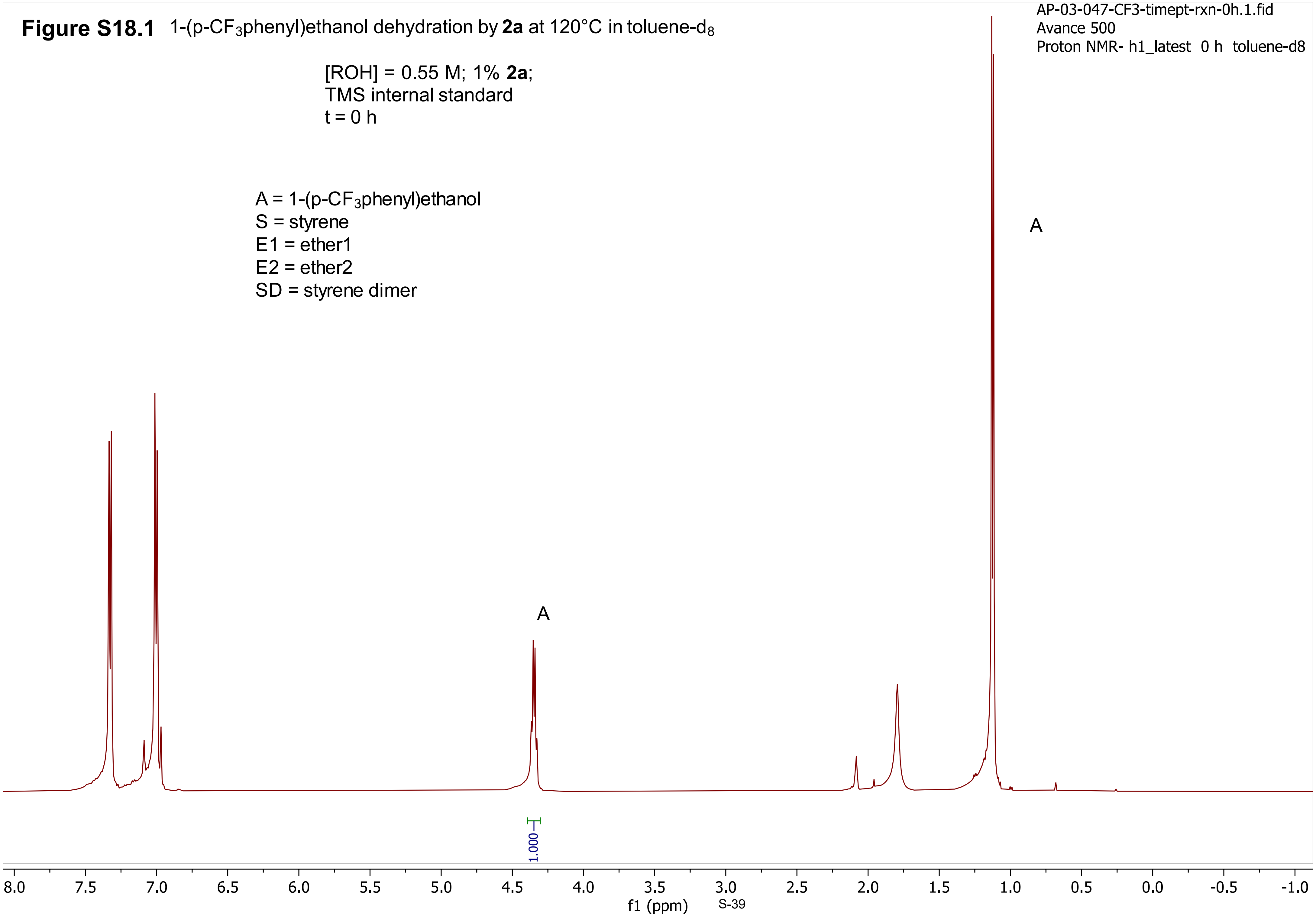

Figure S18.2 1-(p-CF_3phenyl)ethanol dehydration by **2a** at 120°C in toluene-d_8

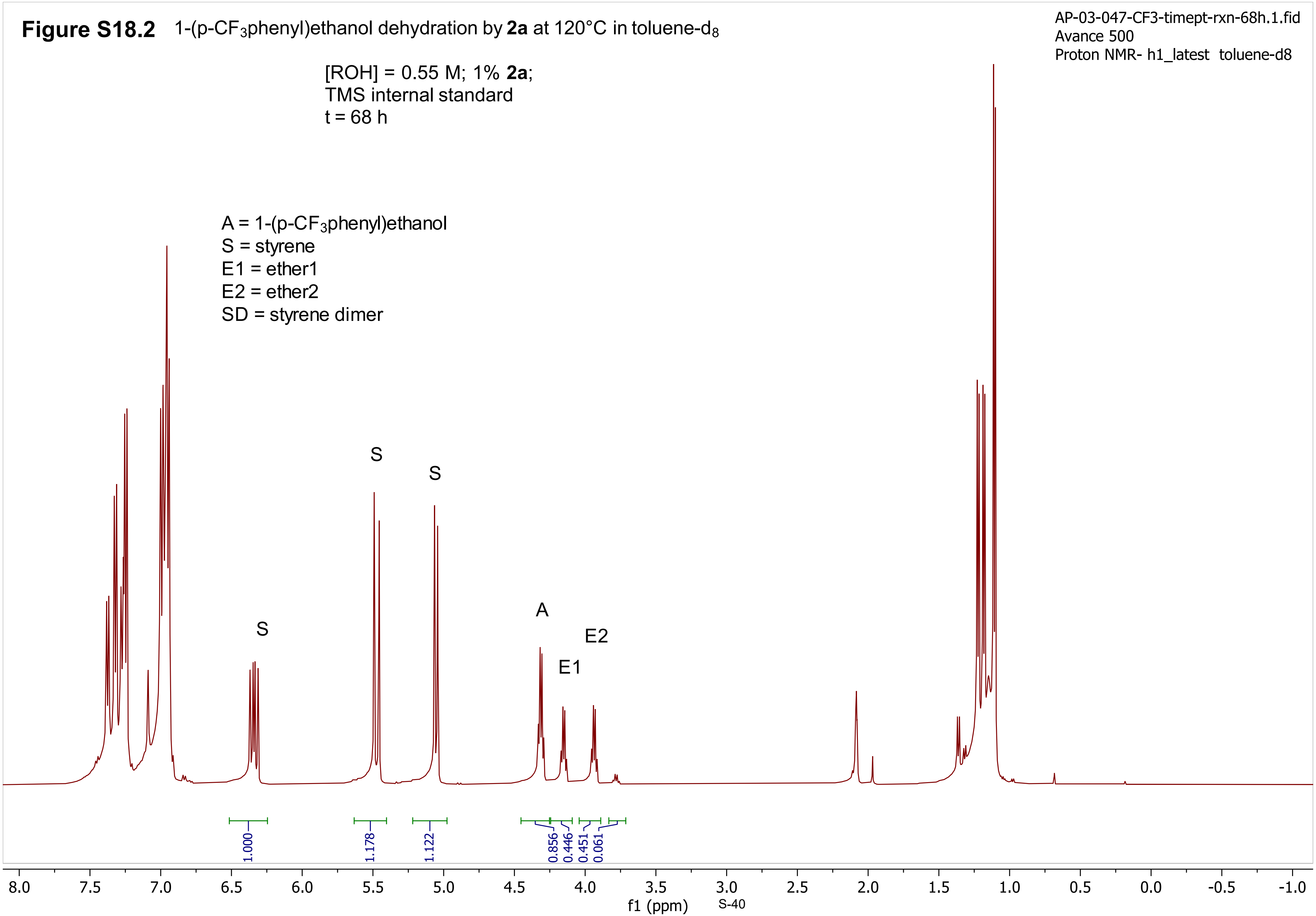

Figure S18.3 1-(p-CF_3phenyl)ethanol dehydration by **2a** at 120°C in toluene-d_8

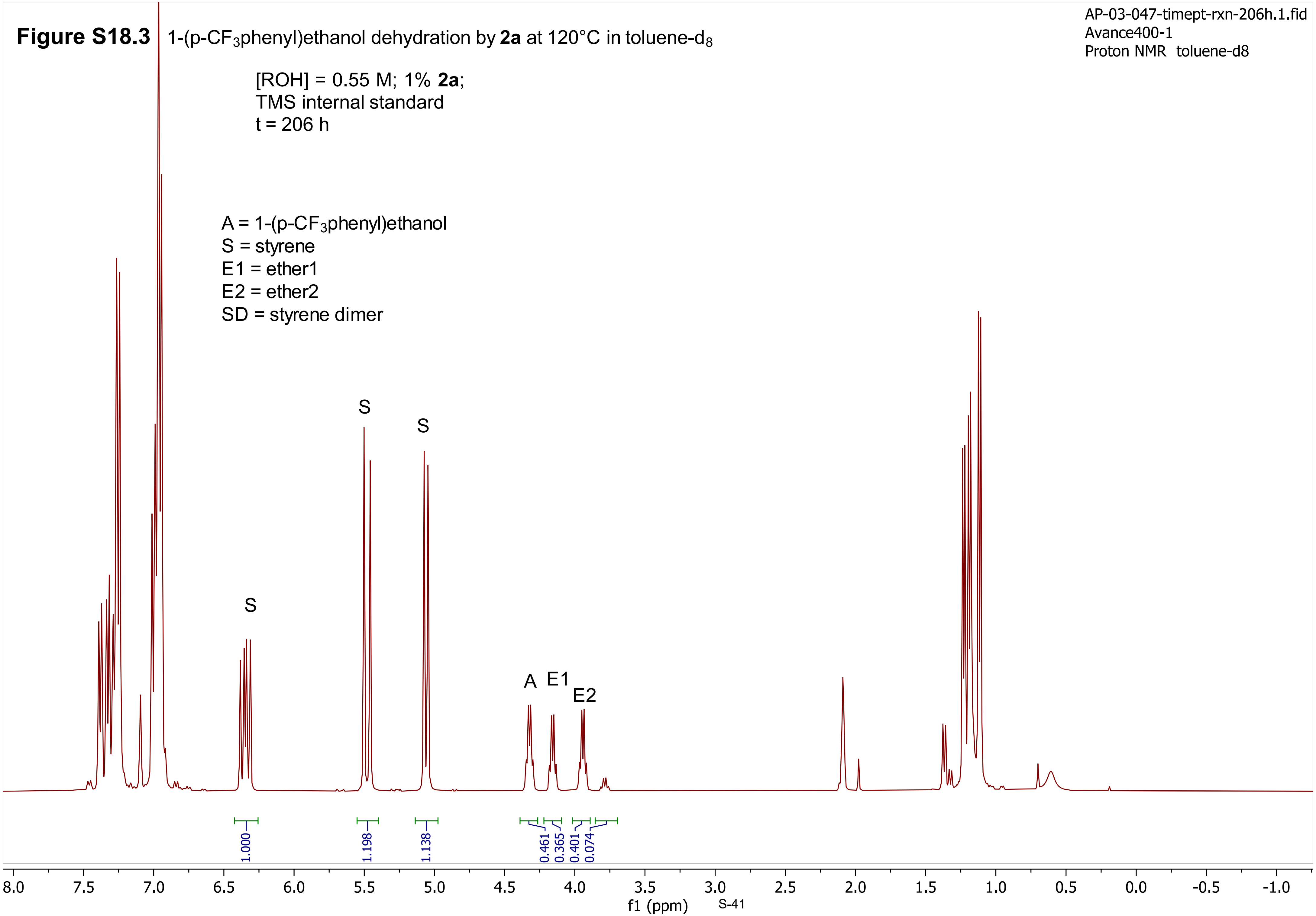

Figure S18.4

p-CF_3-PhEtOH Dehydration

AP-03-047

Reaction conditions: [1-p-CF3-PhEtOH] = 0.55 M; toluene-d8; 1% catalyst **2a**; 120 °C.

J-Young NMR tube

time (h)	area CF3-PhEtOH(1H)	area styrene(1H)	area ether1(2H)	area ether2(2H)	area styrene-dimer(1H)	total weighted area
0	1.000	0.000	0.000	0.000	0.000	1.000
5	1.000	0.009	0.014	0.008	0.000	1.020
12	1.000	0.021	0.030	0.021	0.000	1.046
68	0.856	1.100	0.446	0.451	0.000	2.405
72	0.792	1.115	0.445	0.467	0.000	2.363
90	0.563	1.062	0.362	0.387	0.000	2.000
96	0.572	1.089	0.399	0.402	0.000	2.062
110	0.462	1.062	0.341	0.369	0.000	1.879
206	0.461	1.112	0.365	0.401	0.000	1.956
	δ 4.31	δ 6.34,5.48,5.06	δ 4.15	δ 3.94	na	

time (h)	mol% CF3-PhEtOH	mol% styrene	mol% ether1	mol% ether2	mol% styrene-dimer	mol% total
0	100.000	0.000	0.000	0.000	0.000	100.000
10	98.039	0.882	0.686	0.392	0.000	100.000
12	95.587	1.975	1.434	1.004	0.000	100.000
68	35.600	45.748	9.274	9.378	0.000	100.000
72	33.521	47.178	9.417	9.883	0.000	100.000
90	28.157	53.113	9.052	9.677	0.000	100.000
96	27.742	52.833	9.676	9.749	0.000	100.000
110	24.592	56.512	9.076	9.821	0.000	100.000
206	23.569	56.851	9.330	10.251	0.000	100.000

Area $(PhEtOH)_0$ or styrene set to area 1.00.

mol% = area/#H/total area* 100%

Ethers are area 2H each; PhEtOH & styrene-dimer 1H each

styrene area = average of 3 vinylic peaks

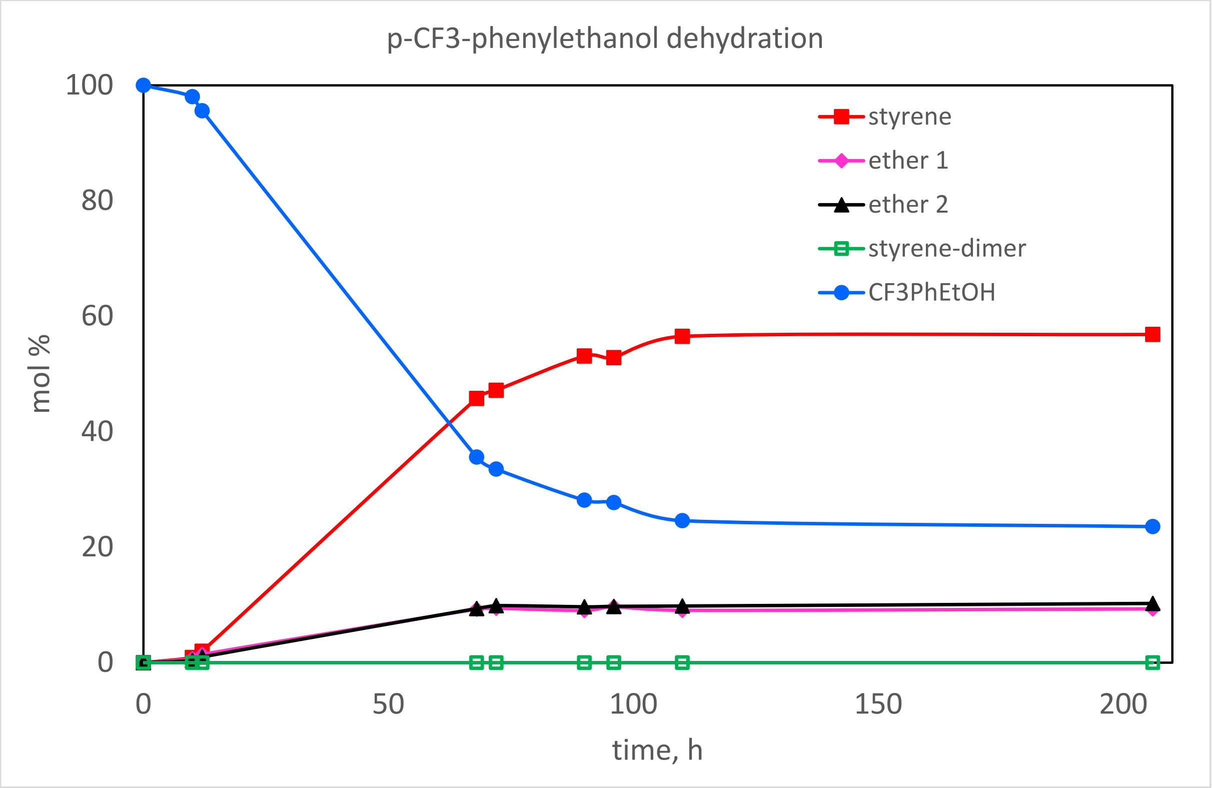

Figure S19.1 1-(p-tolyl)ethanol dehydration by **2a** at 120°C in toluene-d_8

[ROH] = 0.55 M; 1% **2a**;
TMS internal standard
t = 0 h

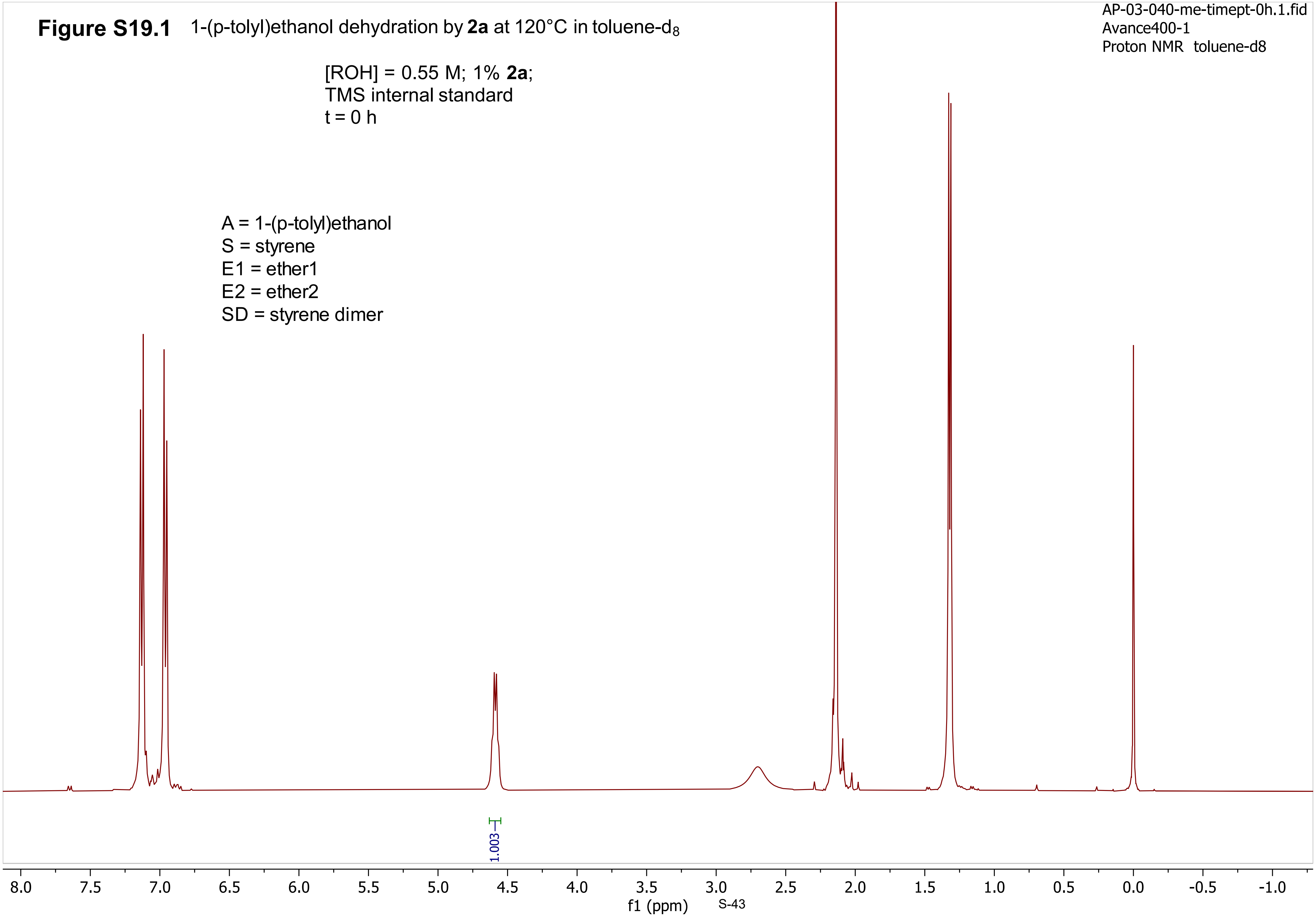

Figure S19.2 1-(p-tolyl)ethanol dehydration by **2a** at 120°C in toluene-d_8

[ROH] = 0.55 M; 1% **2a**;
TMS internal standard
t = 1 h

A = 1-(p-tolyl)ethanol
S = styrene
E1 = ether1
E2 = ether2
SD = styrene dimer

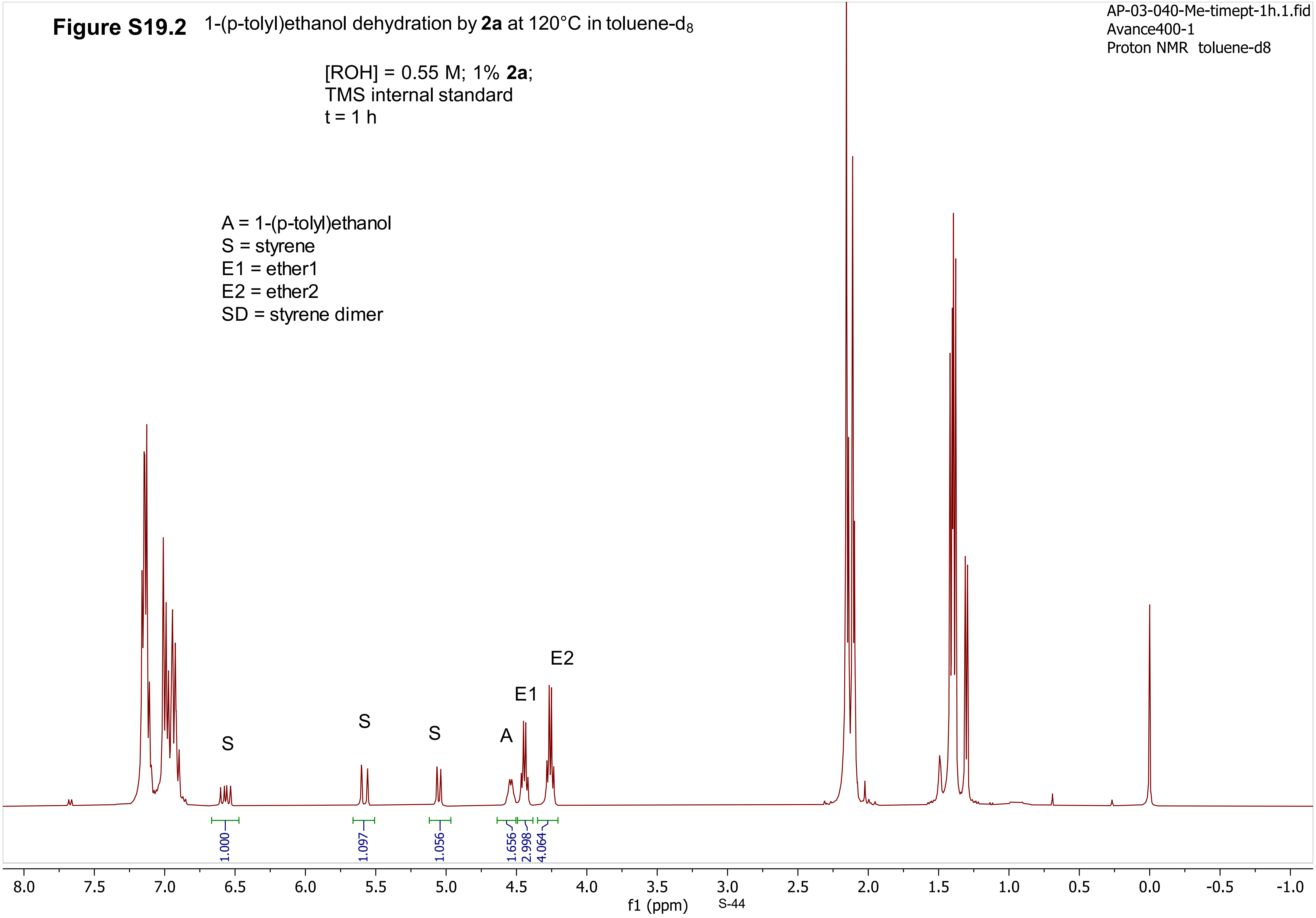

Figure S19.3 1-(p-tolyl)ethanol dehydration by **2a** at 120°C in toluene-d_8

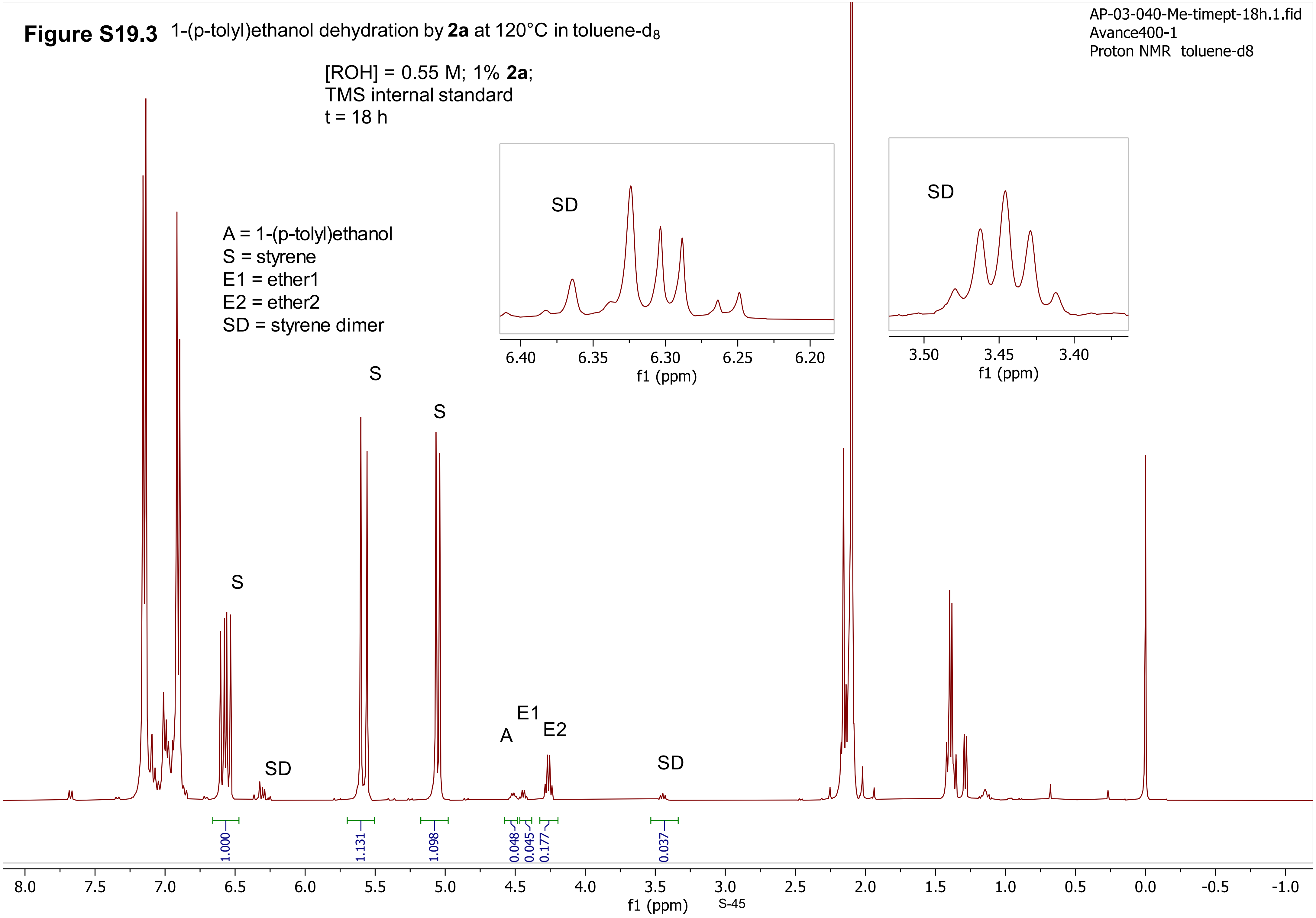

Figure S19.4

p-CH_3-PhEtOH Dehydration

AP-03-040

Reaction conditions: [1-p-CH3-PhEtOH] = 0.55 M; toluene-d8; 1% catalyst **2a**; 120 °C.

J-Young NMR tube

time (h)	area CH3-PhEtOH(1H)	area styrene(1H)	area ether1(2H)	area ether2(2H)	area styrene-dimer(1H)	total weighted area
0	1.000	0.000	0.000	0.000	0.000	1.000
1	1.656	1.051	2.998	4.064	0.000	5.713
2	0.823	1.061	1.747	2.744	0.000	3.599
3	0.488	1.072	1.023	1.889	0.007	2.484
4	0.363	1.077	0.666	1.412	0.011	1.946
5	0.280	1.080	0.506	1.142	0.012	1.650
6	0.224	1.075	0.397	0.960	0.017	1.449
7	0.189	1.077	0.313	0.813	0.019	1.300
8	0.154	1.080	0.251	0.696	0.020	1.178
16	0.044	1.076	0.060	0.214	0.033	0.736
18	0.048	1.076	0.045	0.177	0.037	0.716

time (h)	mol% CH3-PhEtOH	mol% styrene	mol% ether1	mol% ether2	mol% styrene-dimer	mol% total
0	100.000	0.000	0.000	0.000	0.000	100.000
1	28.989	9.199	26.241	35.571	0.000	100.000
2	22.867	14.740	24.271	38.122	0.000	100.000
3	19.648	21.588	20.595	38.028	0.141	100.000
4	18.655	27.666	17.113	36.283	0.283	100.000
5	16.968	32.734	15.332	34.603	0.364	100.000
6	15.462	37.115	13.702	33.134	0.587	100.000
7	14.538	41.423	12.038	31.269	0.731	100.000
8	13.079	45.860	10.658	29.554	0.849	100.000
16	5.981	73.154	4.078	14.545	2.243	100.000
18	6.707	75.198	3.144	12.366	2.585	100.000

Area $(PhEtOH)_0$ or styrene set to area 1.00.
mol% = area/#H/total area* 100%

Ethers are area 2H each; PhEtOH & styrene-dimer 1H each
styrene area = average of 3 vinylic peaks

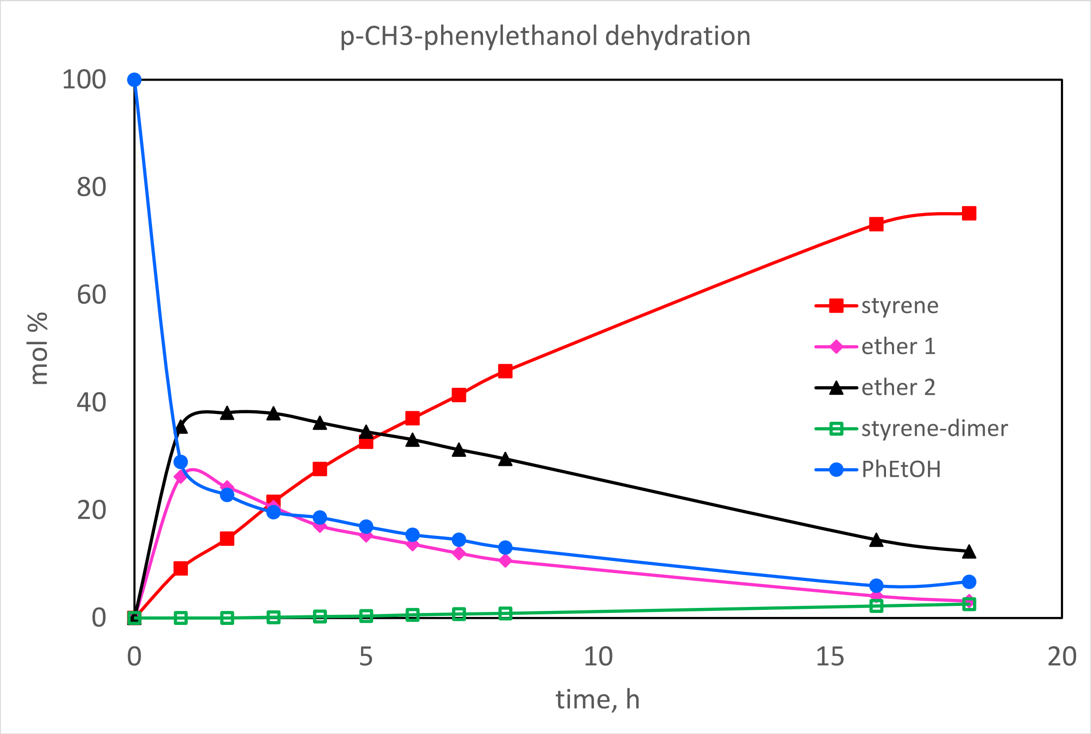

Figure S20.1 1-(p-Clphenyl)ethanol dehydration by **2a** at 120°C in toluene-d_8

[ROH] = 0.55 M; 1% **2a**;
TMS internal standard
t = 0 h

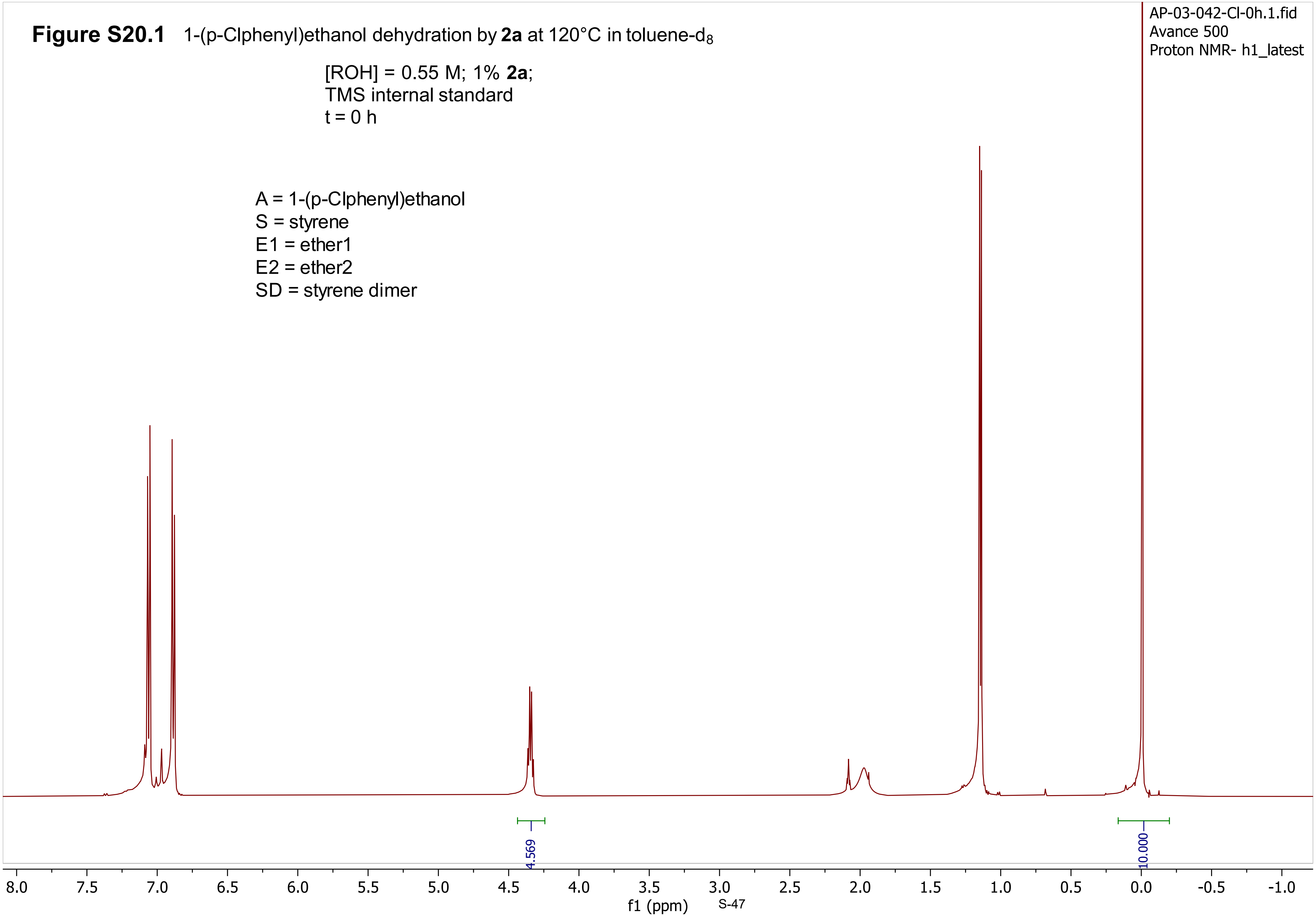

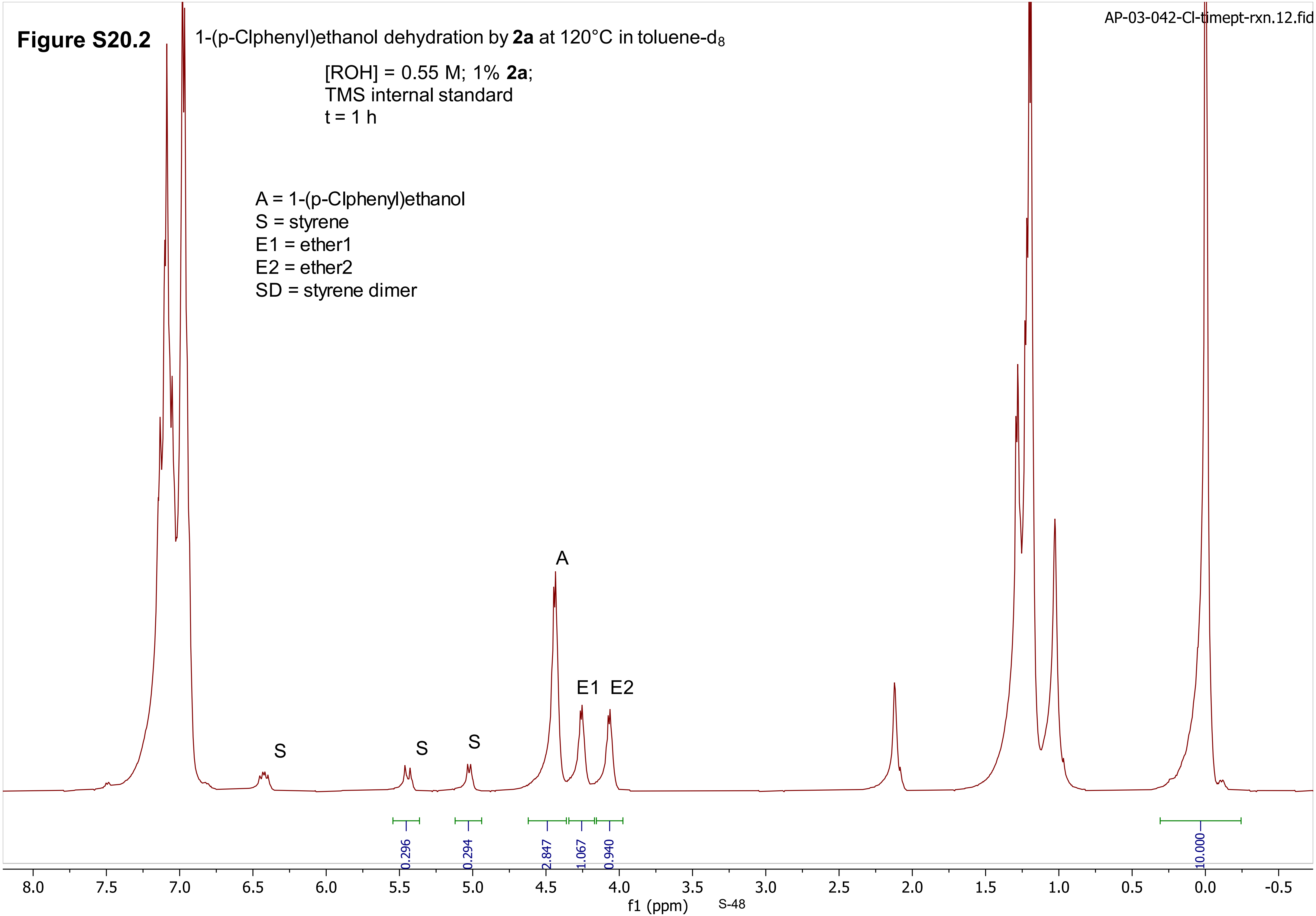
Figure S20.2
1-(p-Clphenyl)ethanol dehydration by 2a at 120°C in toluene-d8
[ROH] = 0.55 M; 1% 2a;
TMS internal standard
t = 1 h
A = 1-(p-Clphenyl)ethanol
S = styrene
E1 = ether1
E2 = ether2
SD = styrene dimer
AP-03-042-Cl-timept-rxn.12.fid
S
S
S
A
E1
E2
0.296
0.294
2.847
1.067
0.940
10.000
8.0
7.5
7.0
6.5
6.0
5.5
5.0
4.5
4.0
3.5
3.0
2.5
2.0
1.5
1.0
0.5
0.0
-0.5
f1 (ppm)

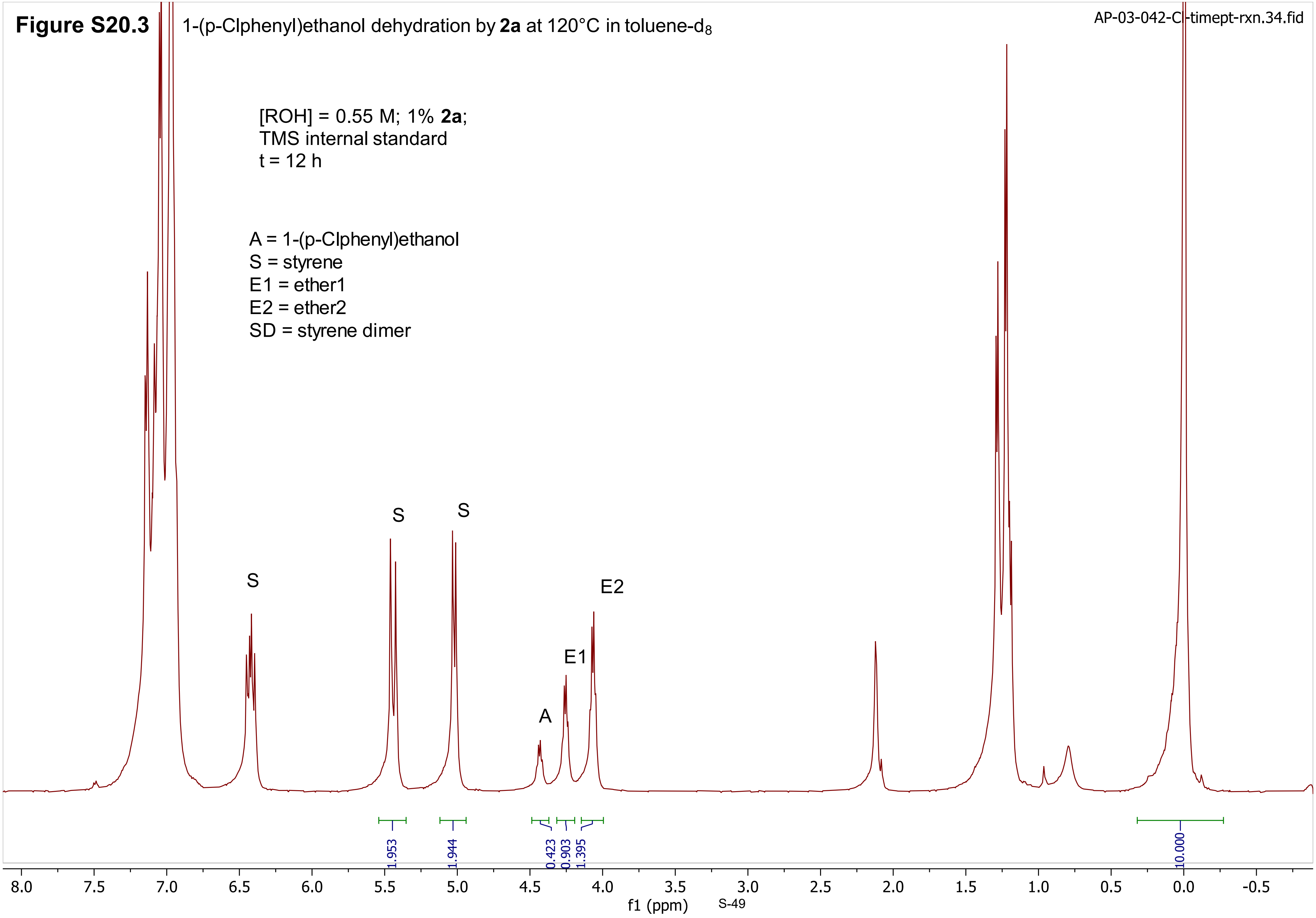

Figure S20.3
1-(p-Clphenyl)ethanol dehydration by 2a at 120°C in toluene-d8
AP-03-042-Cl-timept-rxn.34.fid
[ROH] = 0.55 M; 1% 2a;
TMS internal standard
t = 12 h
A = 1-(p-Clphenyl)ethanol
S = styrene
E1 = ether1
E2 = ether2
SD = styrene dimer
S
S
S
A
E1
E2
1.953
1.944
0.423
0.903
1.395
10.000
8.0
7.5
7.0
6.5
6.0
5.5
5.0
4.5
4.0
3.5
3.0
2.5
2.0
1.5
1.0
0.5
0.0
-0.5
f1 (ppm)

Figure S20.4

p-Cl-PhEtOH Dehydration

AP-03-042

Reaction conditions: [1-p-Cl-PhEtOH] = 0.55 M; toluene-d8; 1% catalyst **2a**; 120 °C.

J-Young NMR tube

time (h)	area Cl-PhEtOH(1H)	area styrene(1H)	area ether1(2H)	area ether2(2H)	area styrene-dimer(1H)	total weighted area
0	4.569	0.000	0.000	0.000	0.000	4.569
0.5	3.563	0.179	0.829	0.710	0.000	4.512
1	2.847	0.295	1.067	0.940	0.000	4.146
1.5	2.265	0.381	1.252	1.171	0.000	3.858
2	1.889	0.462	1.303	1.241	0.000	3.623
2.5	1.612	0.557	1.412	1.318	0.000	3.534
3	1.400	0.672	1.423	1.404	0.000	3.486
3.5	1.179	0.757	1.400	1.431	0.000	3.351
4	1.070	0.819	1.397	1.413	0.000	3.294
4.5	0.992	0.922	1.420	1.471	0.000	3.359
5	0.908	0.971	1.402	1.532	0.000	3.346
5.5	0.851	1.133	1.450	1.558	0.000	3.488
6	0.784	1.222	1.432	1.594	0.000	3.519
6.5	0.730	1.326	1.384	1.649	0.000	3.572
7	0.628	1.300	1.272	1.551	0.000	3.339
7.5	0.608	1.420	1.236	1.553	0.000	3.423
8	0.585	1.441	1.220	1.478	0.000	3.375
8.5	0.536	1.553	1.176	1.483	0.000	3.419
9	0.496	1.571	1.112	1.504	0.000	3.375
9.5	0.446	1.483	1.032	1.470	0.000	3.180
10	0.435	1.628	1.011	1.387	0.000	3.262
10.5	0.418	1.729	0.959	1.380	0.000	3.317
11	0.474	1.927	1.011	1.431	0.000	3.622
11.5	0.398	1.965	0.923	1.409	0.000	3.529
12	0.423	1.949	0.903	1.395	0.000	3.521
	δ 4.42	δ 5.44,5.01	δ 4.25	δ 4.07		

time (h)	mol% Cl-PhEtOH(1H)	mol% styrene	mol% ether1	mol% ether2	mol% styrene-dimer	mol% total
0	100.000	0.000	0.000	0.000	0.000	100.000
0.5	78.976	3.968	9.188	7.869	0.000	100.000
1	68.677	7.116	12.869	11.338	0.000	100.000
1.5	58.717	9.877	16.228	15.178	0.000	100.000
2	52.146	12.740	17.985	17.129	0.000	100.000
2.5	45.620	15.749	19.980	18.650	0.000	100.000
3	40.166	19.280	20.413	20.141	0.000	100.000
3.5	35.184	22.575	20.889	21.352	0.000	100.000
4	32.488	24.852	21.208	21.451	0.000	100.000
4.5	29.533	27.434	21.137	21.896	0.000	100.000
5	27.141	29.009	20.954	22.896	0.000	100.000
5.5	24.401	32.473	20.789	22.337	0.000	100.000
6	22.279	34.726	20.347	22.648	0.000	100.000
6.5	20.437	37.108	19.373	23.082	0.000	100.000
7	18.808	38.919	19.048	23.226	0.000	100.000
7.5	17.765	41.490	18.057	22.688	0.000	100.000
8	17.333	42.696	18.074	21.896	0.000	100.000
8.5	15.679	45.429	17.201	21.691	0.000	100.000
9	14.698	46.540	16.477	22.285	0.000	100.000
9.5	14.025	46.635	16.226	23.113	0.000	100.000
10	13.335	49.908	15.497	21.260	0.000	100.000
10.5	12.604	52.133	14.458	20.805	0.000	100.000
11	13.087	53.203	13.956	19.754	0.000	100.000
11.5	11.278	55.681	13.077	19.963	0.000	100.000
12	12.015	55.347	12.825	19.813	0.000	100.000

Area TMS set to area 10.00

mol% = area/#H/total area* 100%

Ethers are area 2H each. No styrene-dimer seen

styrene area = average of 2 vinylic peaks

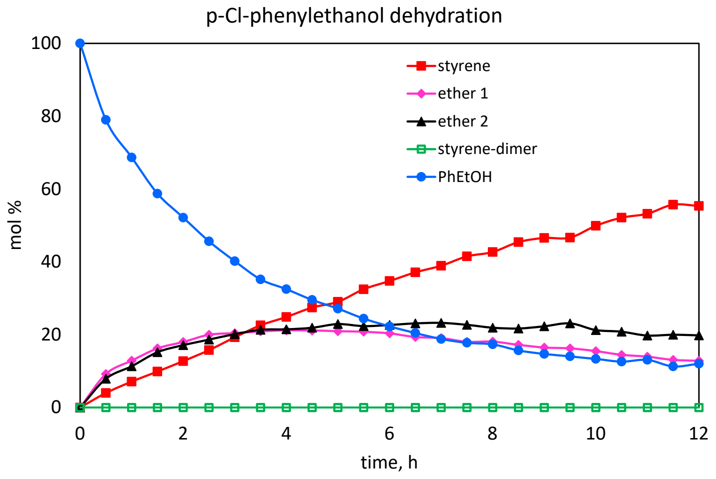

Figure S21.1 1-(p-MeOphenyl)ethanol dehydration by **2a** at 120°C in toluene-d_8

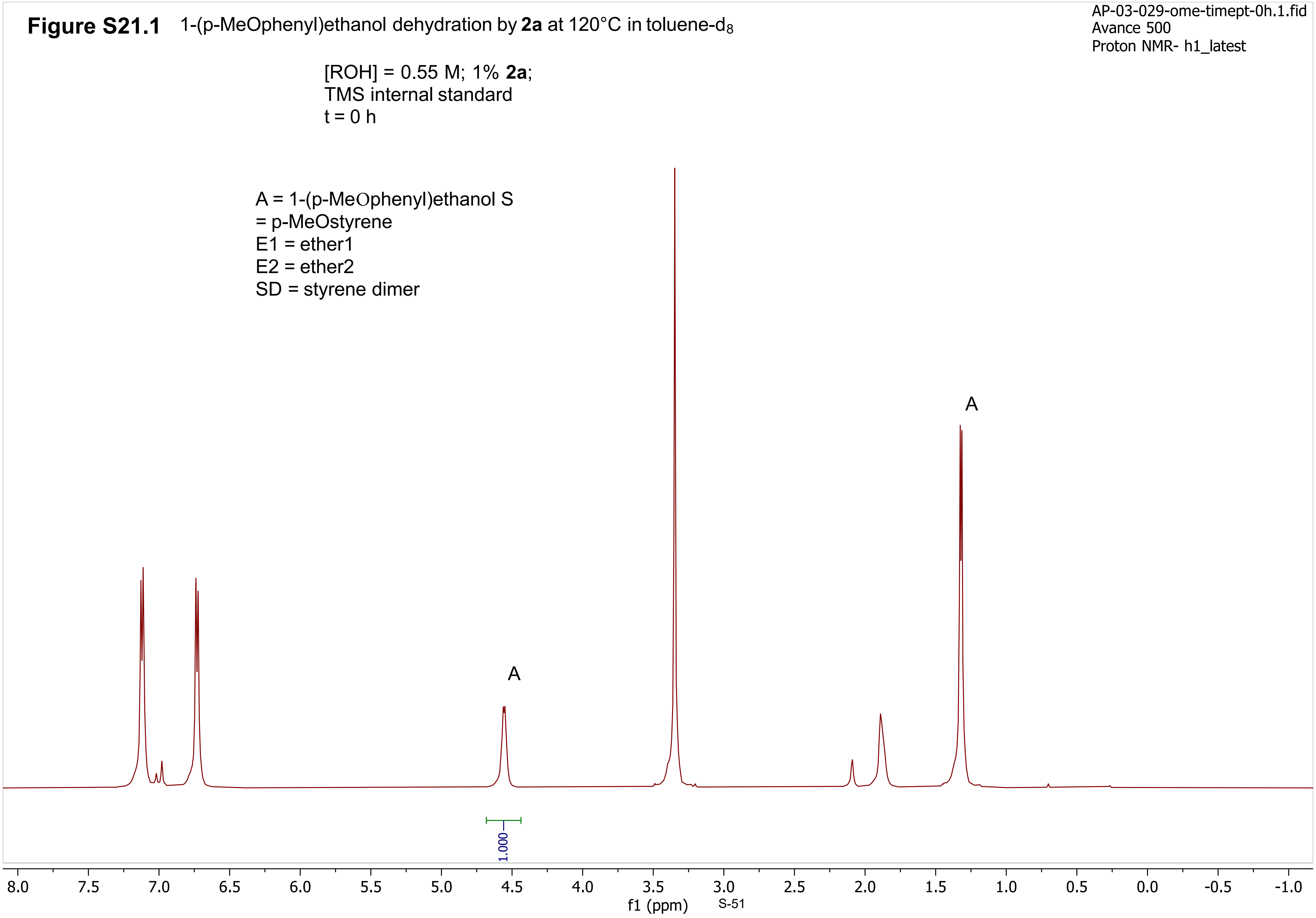

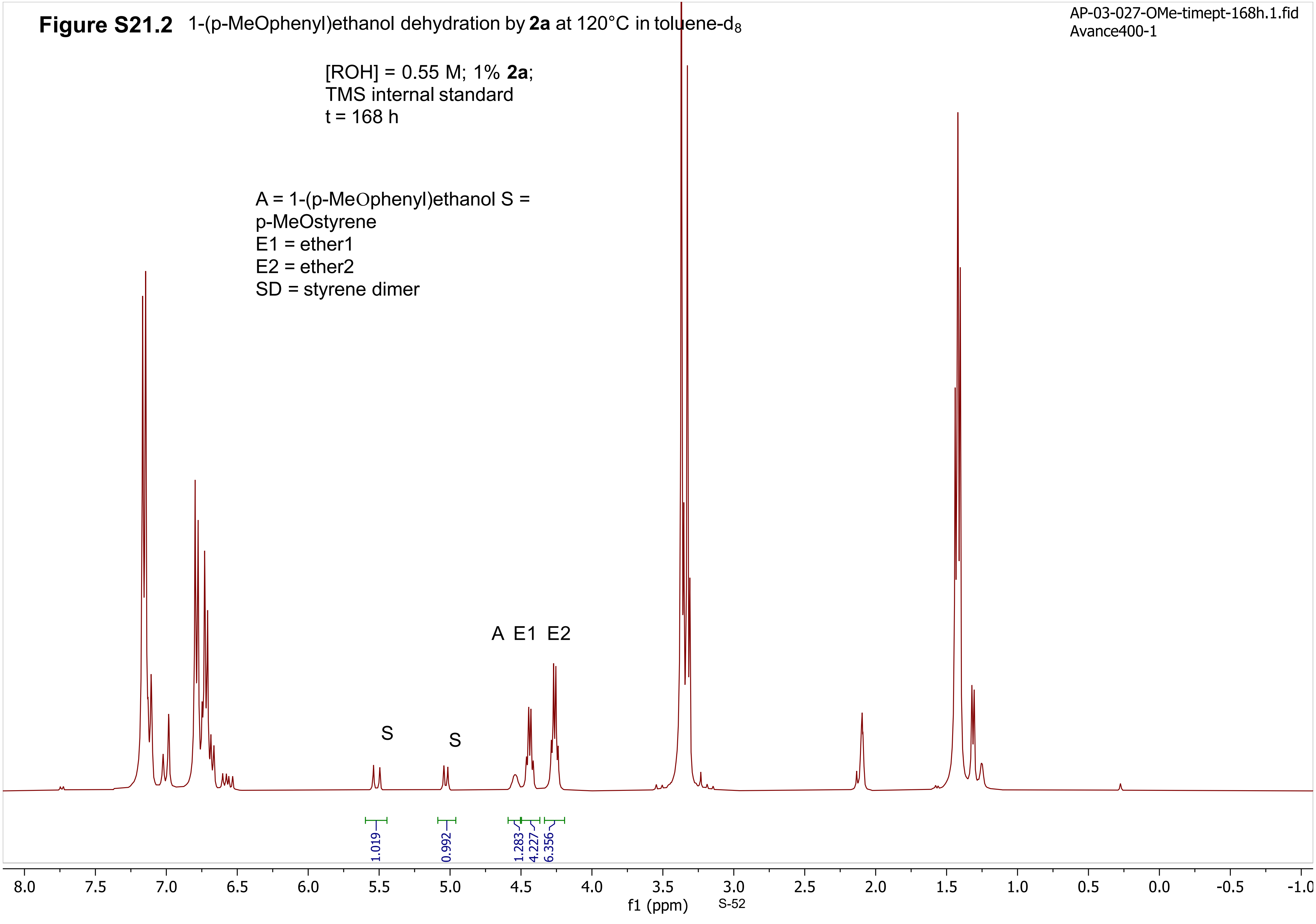
Figure S21.2 1-(p-MeOphenyl)ethanol dehydration by **2a** at 120°C in toluene-d8
AP-03-027-OMe-timept-168h.1.fid
Avance400-1
[ROH] = 0.55 M; 1% **2a**;
TMS internal standard
t = 168 h
A = 1-(p-MeOphenyl)ethanol S = p-MeOstyrene
E1 = ether1
E2 = ether2
SD = styrene dimer
S
S
A E1 E2
1.019
0.992
1.283
4.227
6.356
8.0 7.5 7.0 6.5 6.0 5.5 5.0 4.5 4.0 3.5 3.0 2.5 2.0 1.5 1.0 0.5 0.0 -0.5 -1.0
f1 (ppm)

Figure S21.3

p-MeO-PhEtOH Dehydration

AP-03-029

Reaction conditions: [1-p-MeO-PhEtOH] = 0.55 M; toluene-d8; 1% catalyst **2s**; 120 °C.

J-Young NMR tube

time (h)	area MeO-PhEtOH(1H)	area styrene(1H)	area ether1(2H)	area ether2(2H)	area styrene-dimer(1H)	total weighted area
0	1.000	0.000	0.000	0.000	0.000	1.000
2	1.000	0.000	0.039	0.028	0.000	1.034
17	1.000	0.000	0.075	0.055	0.000	1.065
36	1.000	0.000	0.173	0.143	0.000	1.158
48	1.000	0.014	0.419	0.403	0.000	1.425
55	1.000	0.018	0.468	0.454	0.000	1.479
72	1.000	0.029	0.653	0.666	0.000	1.689
120	1.000	0.419	2.620	3.647	0.000	4.553
144	1.000	0.550	2.818	4.140	0.000	5.029
168	1.000	0.784	3.294	4.954	0.000	5.908
	δ 4.52	δ 5.51,5.03	δ 4.43	δ 4.26		

time (h)	mol% MeO-PhEtOH	mol% styrene	mol% ether1	mol% ether2	mol% styrene-dimer	mol% total
0	100.000	0.000	0.000	0.000	0.000	100.000
2	96.759	0.000	1.887	1.355	0.000	100.000
17	93.897	0.000	3.521	2.582	0.000	100.000
36	86.356	0.000	7.470	6.174	0.000	100.000
48	70.175	0.982	14.702	14.140	0.000	100.000
55	67.636	1.184	15.827	15.353	0.000	100.000
72	59.224	1.718	19.337	19.722	0.000	100.000
120	21.966	9.204	28.775	40.055	0.000	100.000
144	19.885	10.937	28.017	41.161	0.000	100.000
168	16.926	13.270	27.877	41.926	0.000	100.000

Area $(PhEtOH)_0$ set to area 1.00

mol% = area/#H/total area* 100%

Ethers 2H each. No styrene dimer

styrene area = average of 2 vinylic peaks

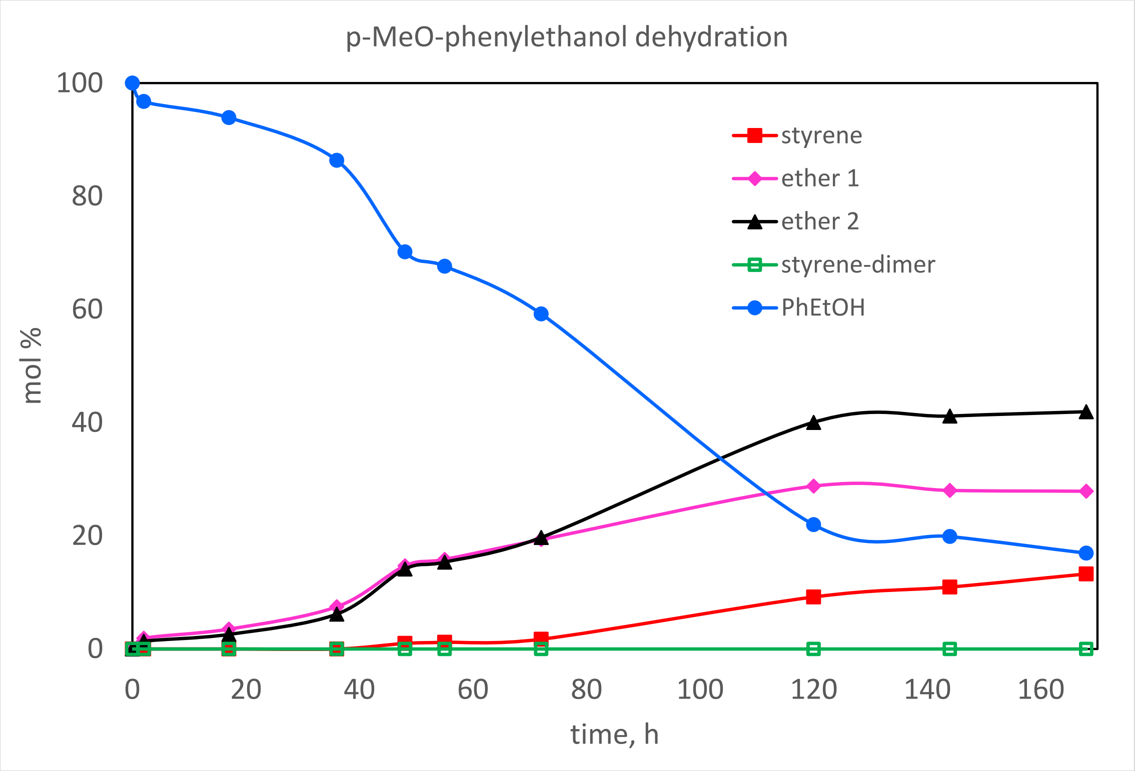

Figure S22.1 1-phenylpropanol dehydration by **21** at 120°C in toluene-d_8

[ROH] = 0.55 M; 1% **21**;
TMS internal standard, sealed NMR tube
t = 0 h

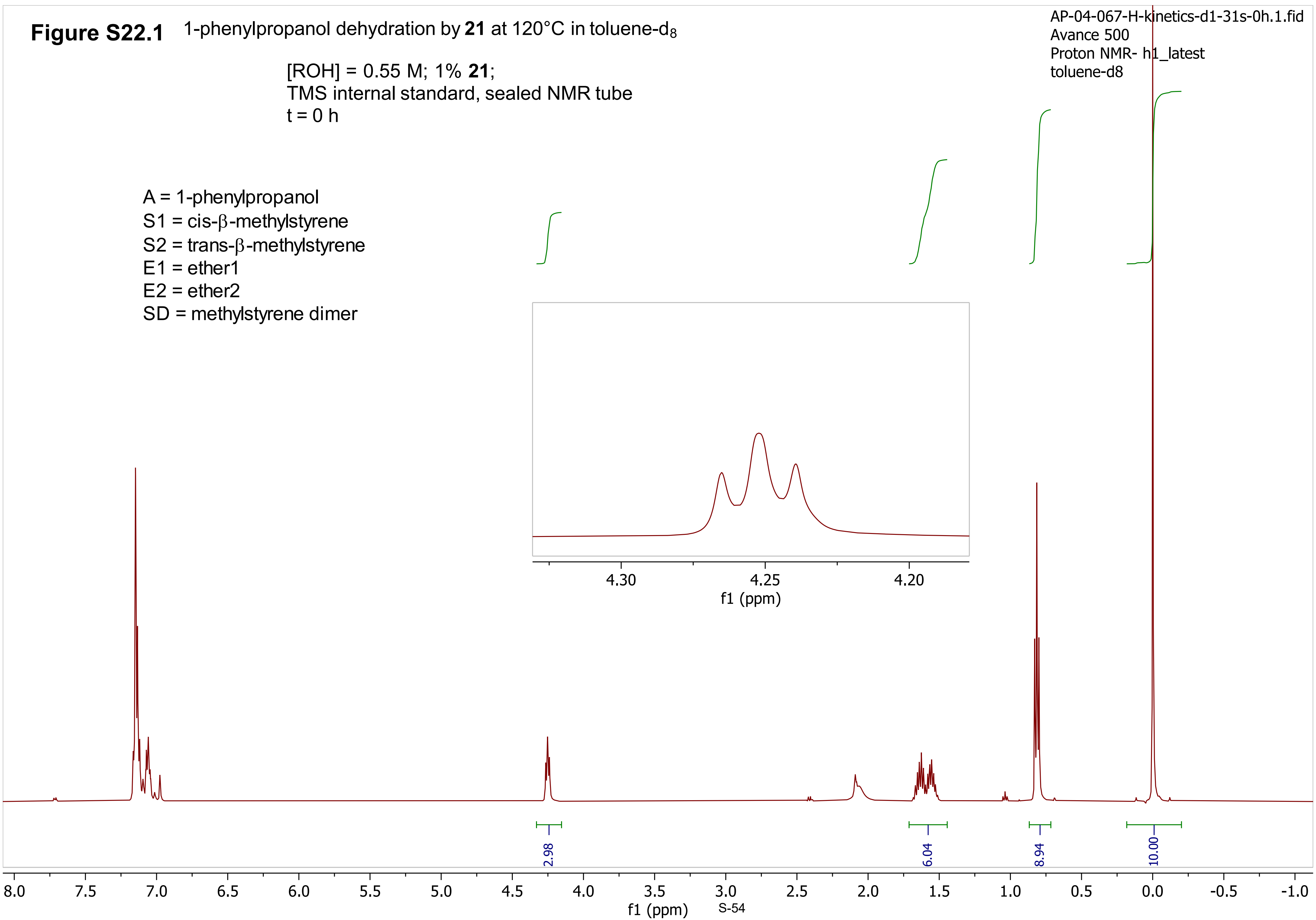

Figure S22.2 1-phenylpropanol dehydration by **2a** at 120°C in toluene-d_8

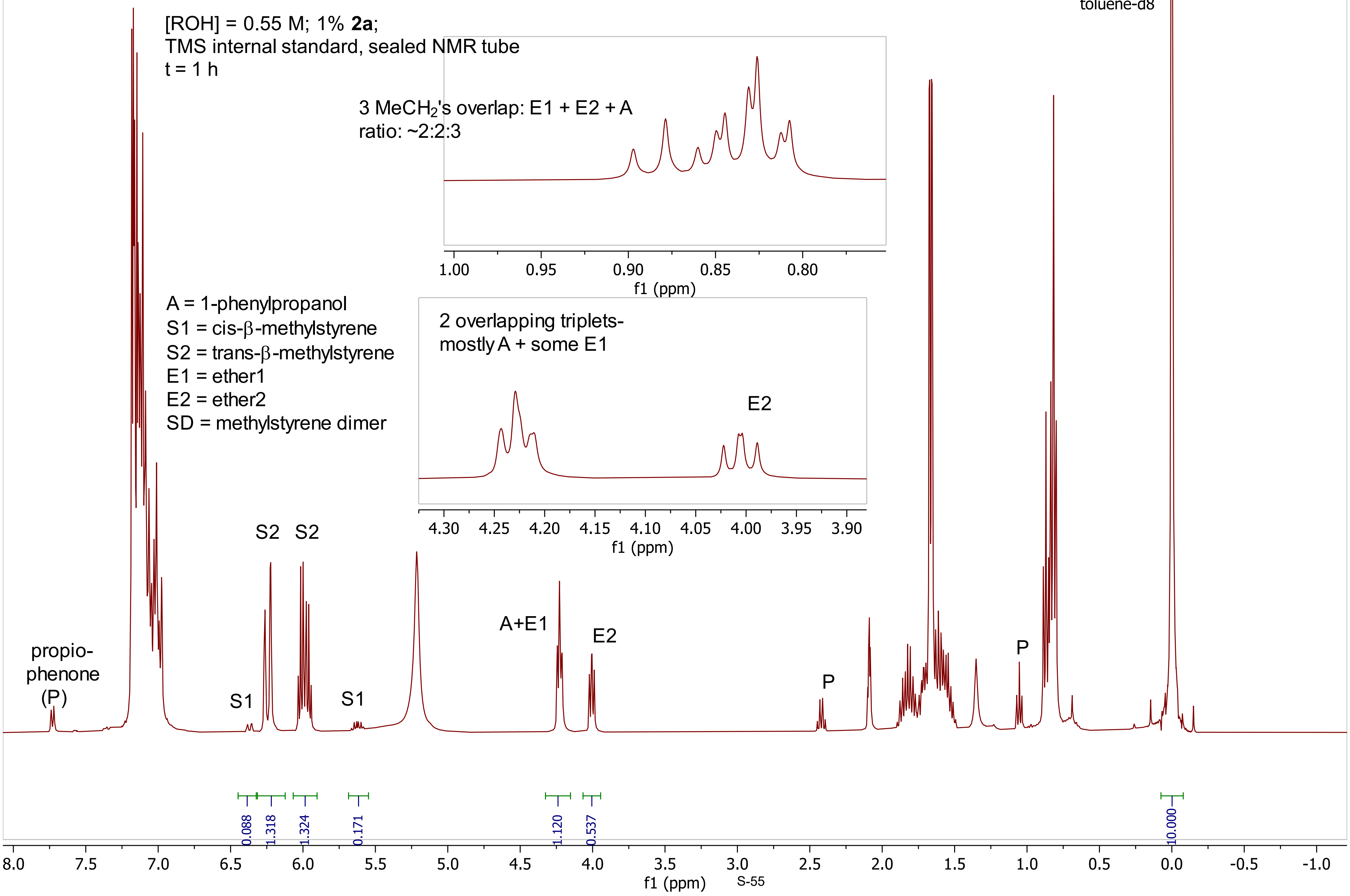

Figure S22.3 1-phenylpropanol dehydration by **2a** at 120°C in toluene-d_8

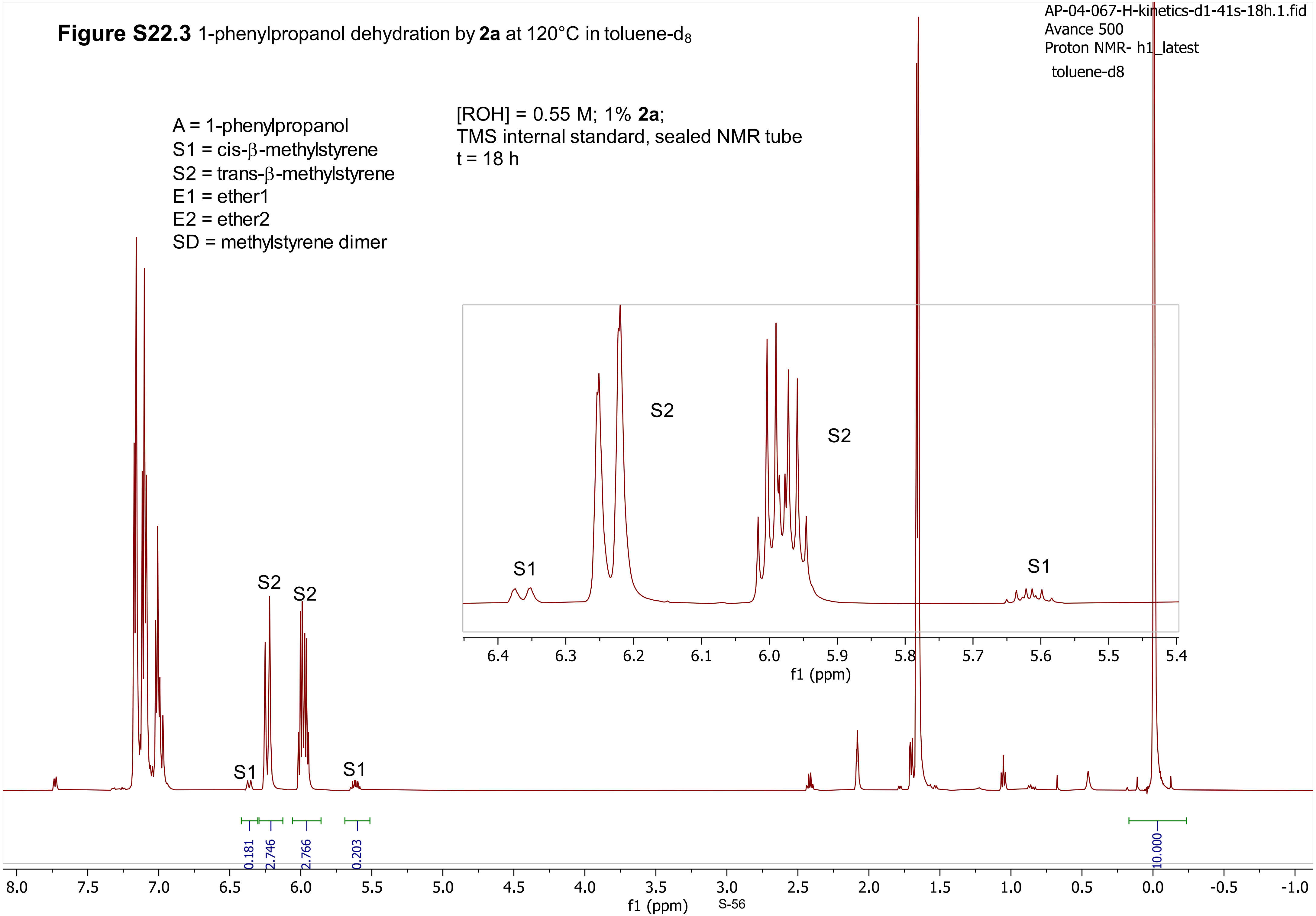

Figure S22.4

1-Phenylpropanol Dehydration

AP-04-067

Reaction conditions: [1-PhPrOH] = 0.55 M; toluene-d8; 1% catalyst **2a**; 120 °C.

Sealed tube

time (h)	area 1-PhPrOH(1H)	area trans-β-MeStyrene	area cis-β-MeStyrene	area ether1(2H)	area ether1(2H)	total weighted area
0	2.980	0.000	0.000	0.000	0.000	2.980
1	0.583	1.591	0.156	0.537	0.537	2.867
2	0.276	1.804	0.130	0.334	0.501	2.627
3	0.120	1.856	0.132	0.240	0.480	2.468
4	0.112	1.900	0.130	0.223	0.446	2.476
5	0.114	1.904	0.152	0.227	0.454	2.510
18	0.000	2.756	0.181	0.000	0.000	2.937
	δ 4.25	δ 6.24,5.99	δ 6.36	δ 4.21	δ 4.00	

time (h)	mol% 1-PhPrOH	mol% trans-β-MeStyrene	mol% cis-β-MeStyrene	mol% ether1	mol% ether2	mol% total
0	100.000	0.000	0.000	0.000	0.000	100.000
1	20.338	55.486	5.442	9.367	9.367	100.000
2	10.506	68.652	4.949	6.357	9.536	100.000
3	4.863	75.198	5.350	4.863	9.726	100.000
4	4.503	76.737	5.250	4.503	9.006	100.000
5	4.522	75.857	6.056	4.522	9.044	100.000
18	0.000	93.837	6.163	0.000	0.000	100.000

Area TMS set to area 10.00

mol% = area/#H/total area* 100%

Ethers, are area 2H each; no styrene dimer

trans-styrene is average of 2 vinylic peaks; cis-styrene is δ 6.36 peak

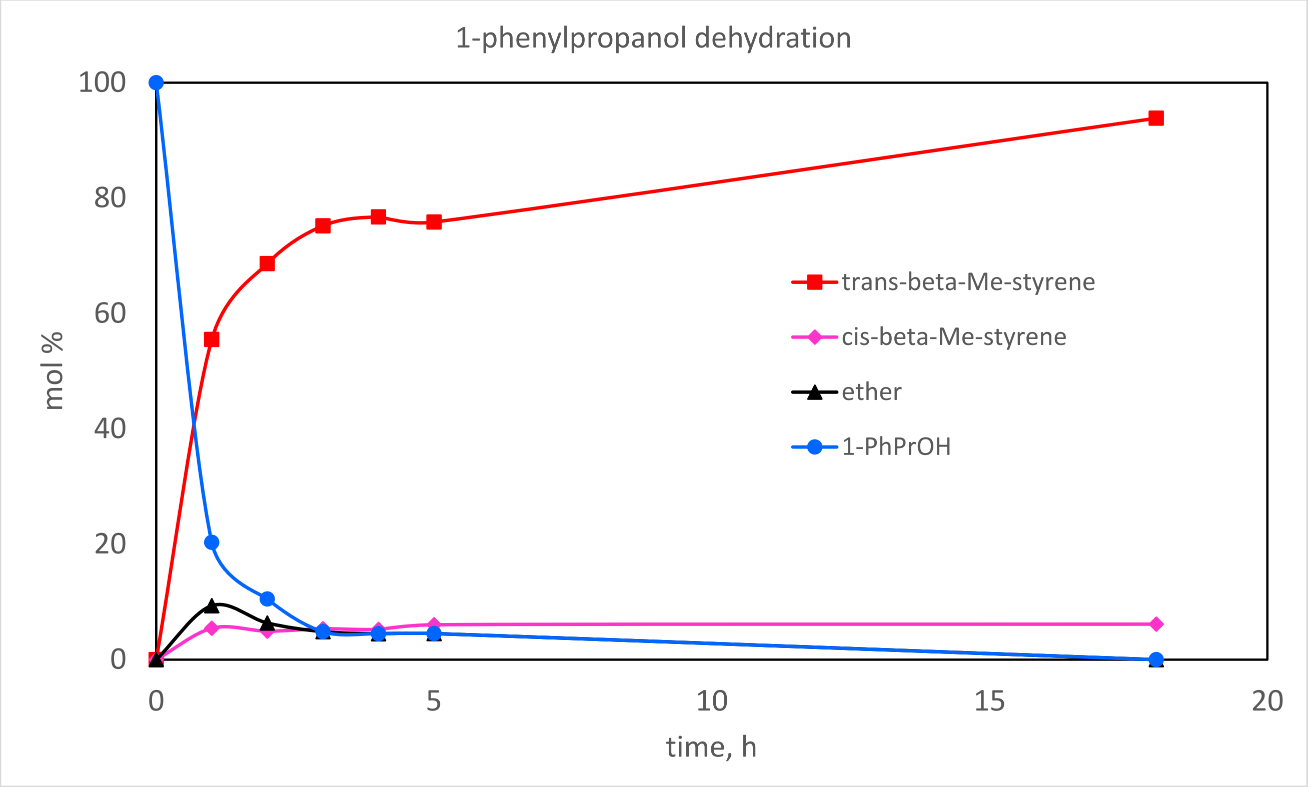

Figure S23.1 1-indanol dehydration by **2a** at 120°C in toluene-d_8

[ROH] = 0.55 M; 1% **2a**;
TMS internal standard, sealed NMR tube
t = 0 h

A = 1-indanol
S = indene
SD = indene dimer

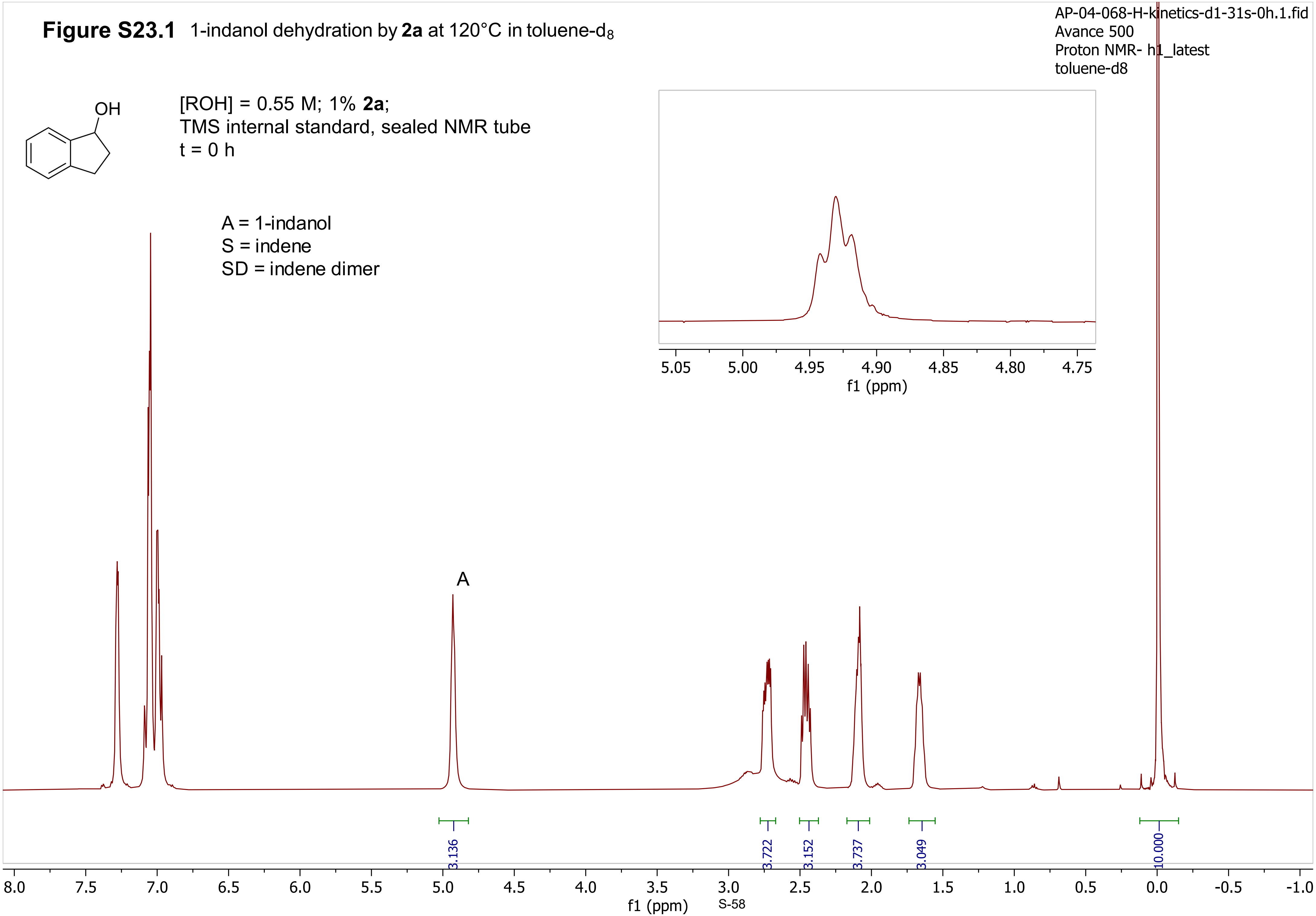

Figure S23.2 1-indanol dehydration by **2a** at 120°C in toluene-d_8

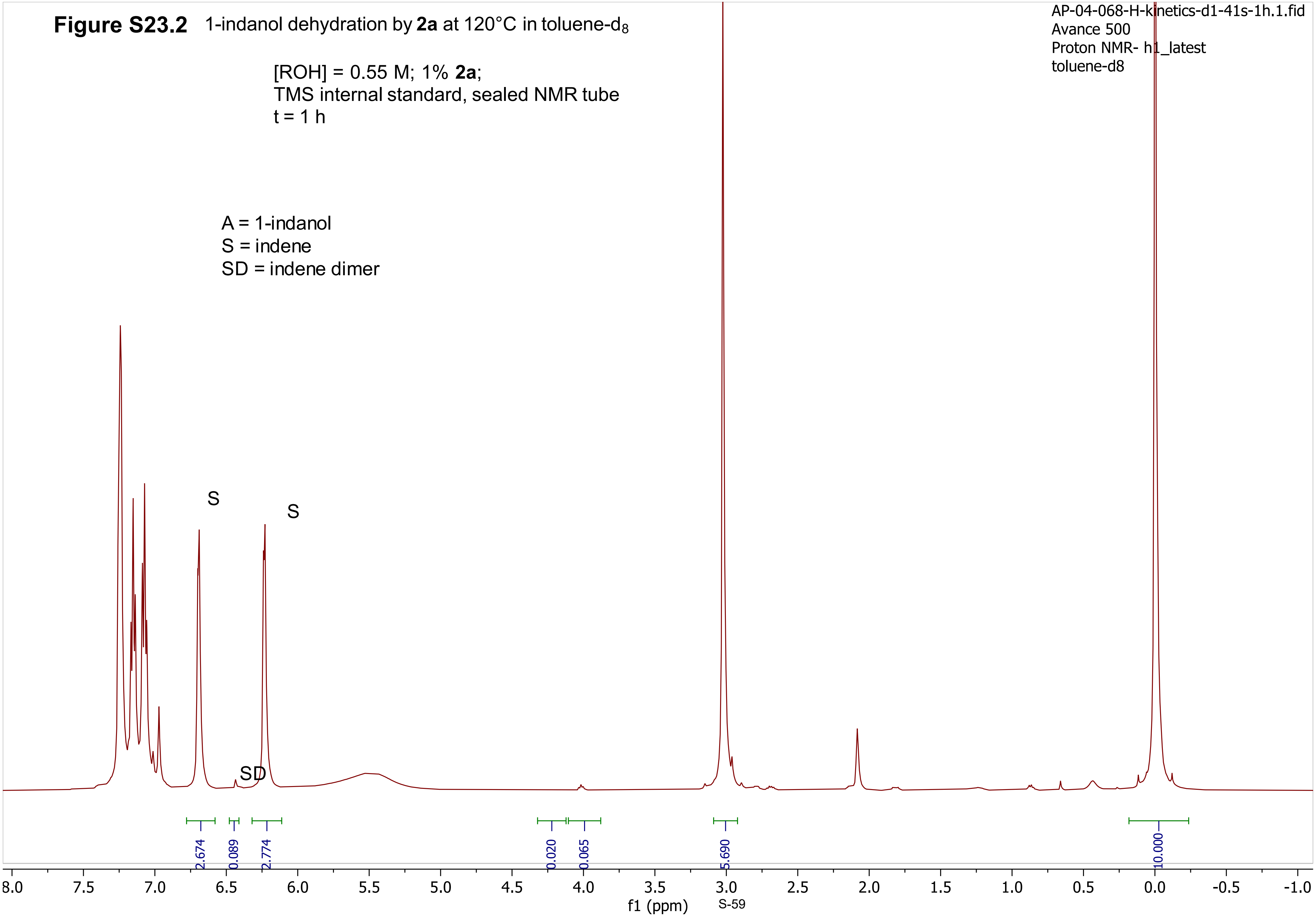

Figure S23.3 1-indanol dehydration by **2a** at 120°C in toluene-d_8

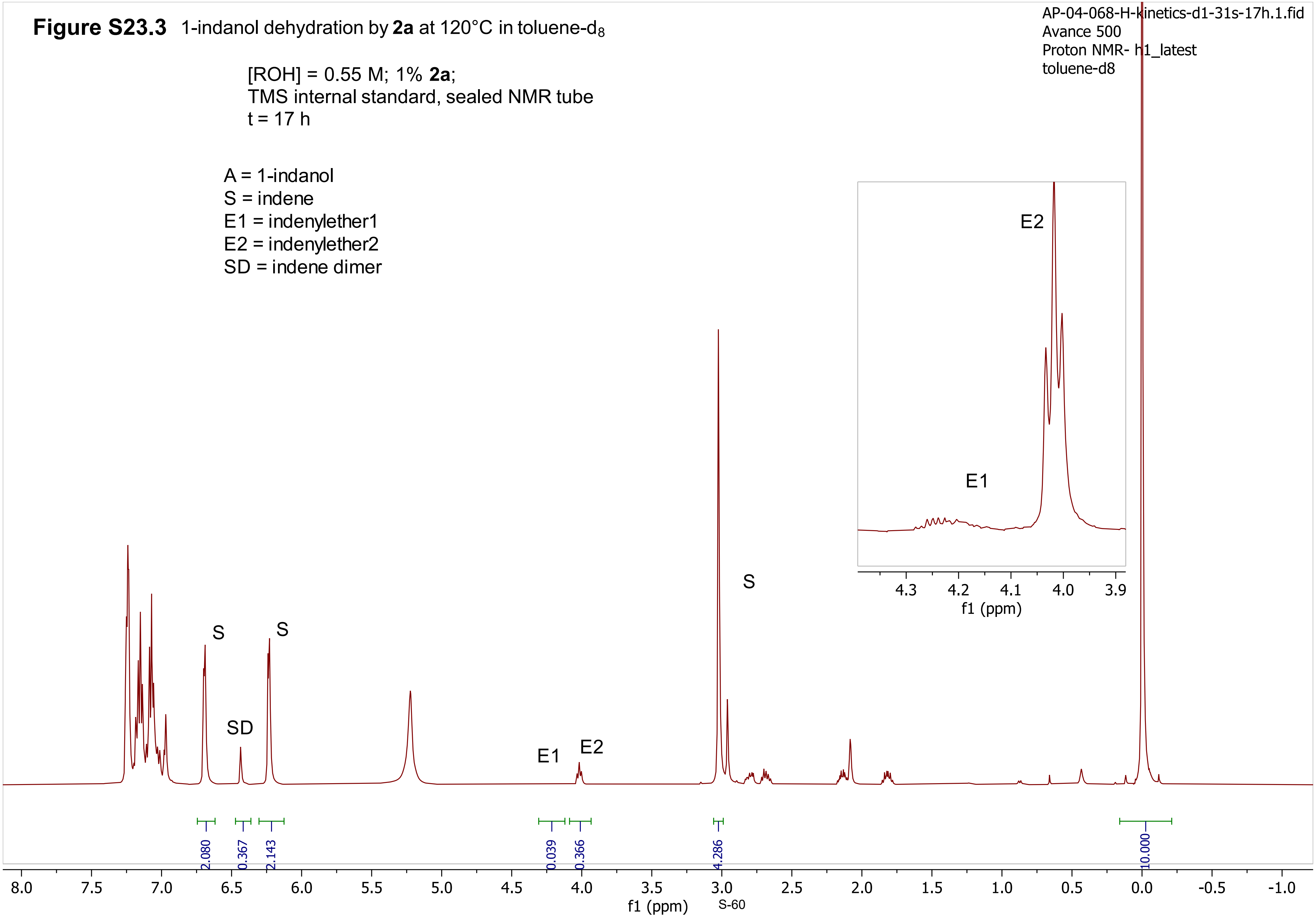

Figure S23.4

1-Indanol Dehydration

AP-04-068

Reaction conditions: [1-indanol] = 0.55 M; toluene-d8; 1% catalyst **2a**; 120 °C.

Sealed tube

time (h)	area 1-indanol(1H)	area indene(1H)	area indenylether1(2H)	area indenylether2(2H)	area indene-dimer(1H)	total weighted area
0	3.136	0.000	0.000	0.000	0.000	3.136
1	0.000	2.724	0.020	0.065	0.089	2.856
2	0.000	2.681	0.022	0.090	0.090	2.827
3	0.000	2.665	0.038	0.116	0.170	2.912
17	0.000	2.112	0.039	0.366	0.37	2.681
	δ 4.93	δ 6.88,6.22	δ 4.22	δ 4.02	δ 6.43	

time (h)	mol% 1-indanol	mol% indene	mol% indenylether1(2H)	mol% indenylether2(2H)	mol% indene-dimer	mol% total
0	100.000	0.000	0.000	0.000	0.000	100.000
1	0.000	95.395	0.350	1.138	3.117	100.000
2	0.000	94.835	0.389	1.592	3.184	100.000
3	0.000	91.516	0.653	1.992	5.839	100.000
17	0.000	78.758	0.727	6.826	13.689	100.000

Area TMS set to area 10.00.

mol% = area/#H/total area* 100%

Indenylethers 2H each; indene dimer, indanol and indene 1H each.

indene area = average of 2 vinylic peaks

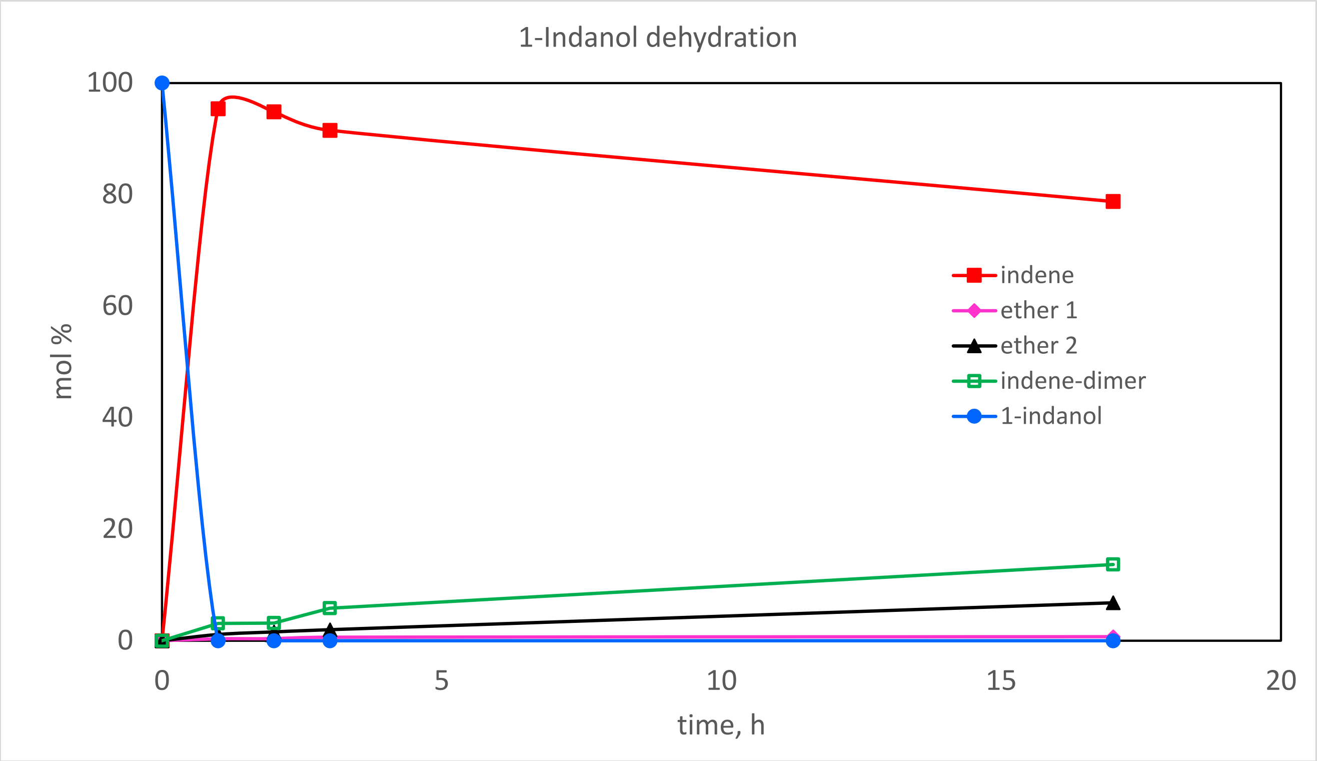

Figure S24.1 1-tetralol dehydration by **2a** at 120°C in toluene-d_8

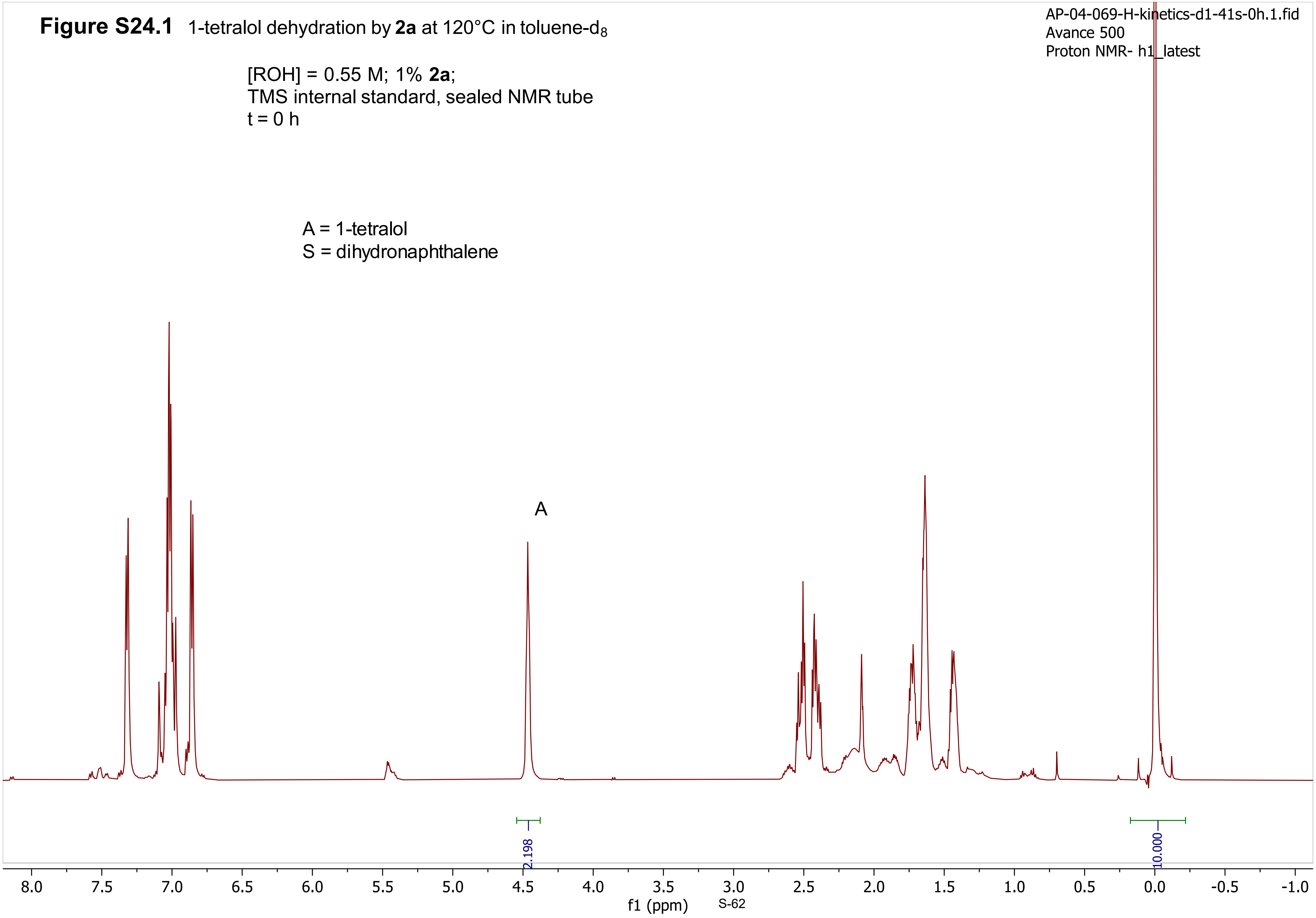

Figure S24.2 1-indanol dehydration by **2a** at 120°C in toluene-d_8

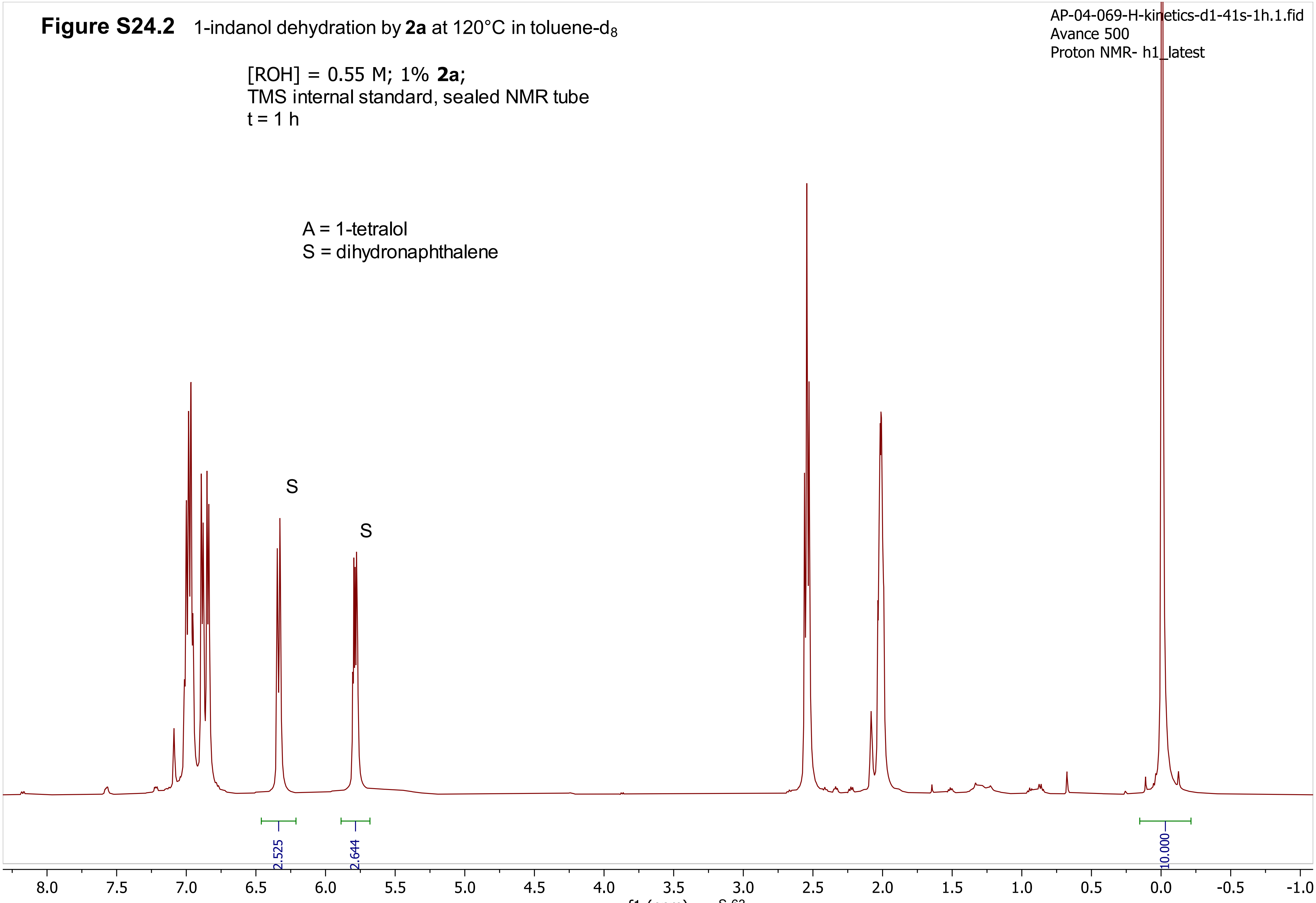

Figure S24.3 1-tetralol dehydration by **2a** at 120°C in toluene-d_8

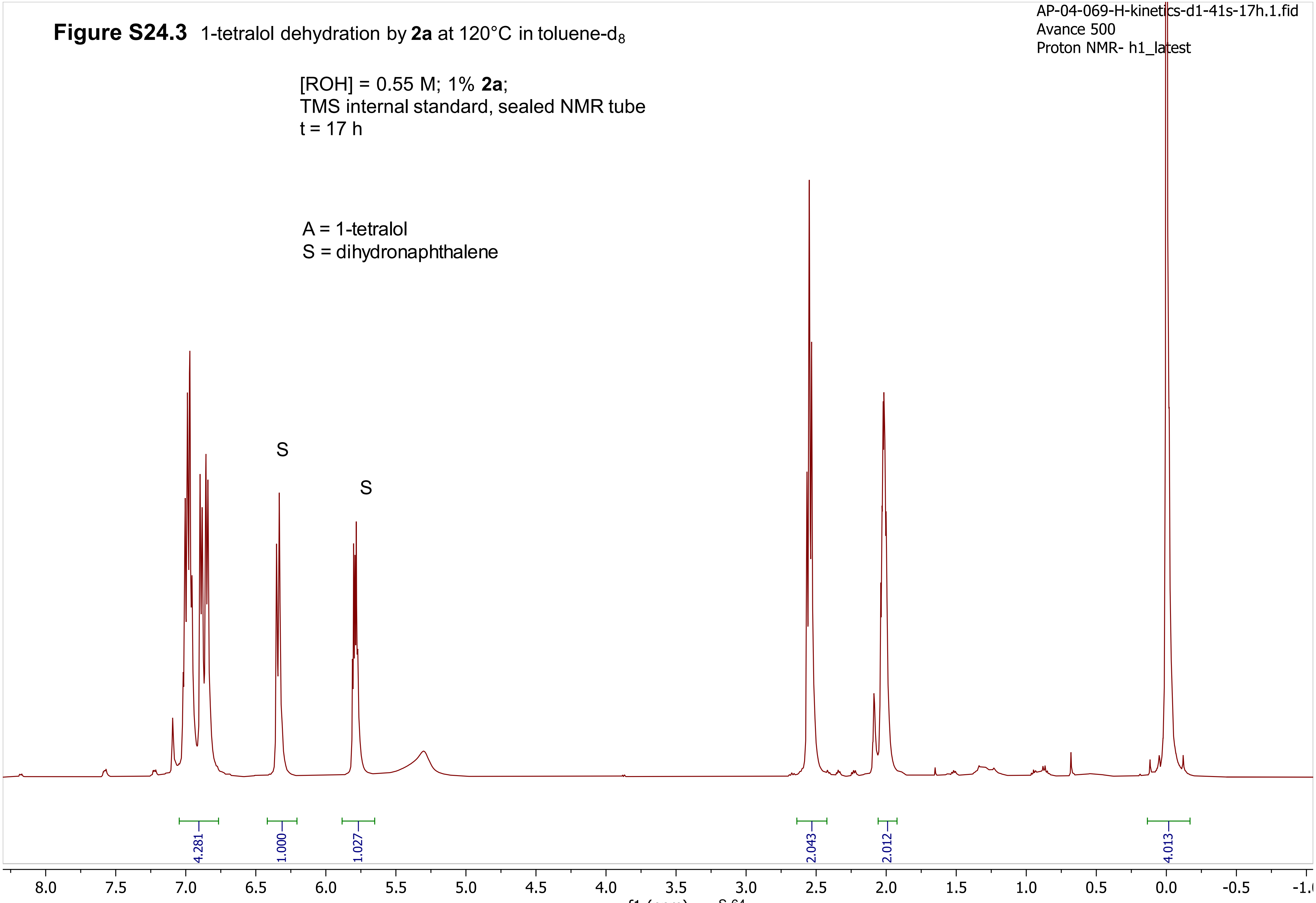

Figure S24.4

1-Tetralol Dehydration

AP-04-069

Reaction conditions: [1-tetralol] = 0.55 M; toluene-d8; 1% catalyst **2a**; 120 °C.

Sealed NMR tube

time (h)	area 1-tetralol(1H)	area dihydronaththalene(1H)	area ether1(2H)	area ether2(2H)	area naphth-dimer(1H)	total weighted area
0	2.198	0.000	0.000	0.000	0.000	2.198
1	0.000	2.525	0.000	0.000	0.000	2.525
2	0.000	2.472	0.000	0.000	0.000	2.472
3	0.000	2.548	0.000	0.000	0.000	2.548
17	0.000	2.492	0.000	0.000	0.00	2.492
	δ 4.46	δ 6.33				

time (h)	mol% 1-tetralol(1H)	mol% dihydronaththalene(1H)	mol% ether1(2H)	mol% ether2(2H)	mol% naphth-dimer(1H)	mol% total
0	100.000	0.000	0.000	0.000	0.000	100.000
1	0.000	100.000	0.000	0.000	0.000	100.000
2	0.000	100.000	0.000	0.000	0.000	100.000
3	0.000	100.000	0.000	0.000	0.000	100.000
17	0.000	100.000	0.000	0.000	0.000	100.000

Area TMS set to area 10.00.

no ethers or dimer seen, tatralol and dihydronaphthalene 1H each.

mol% = area/#H/total area* 100%

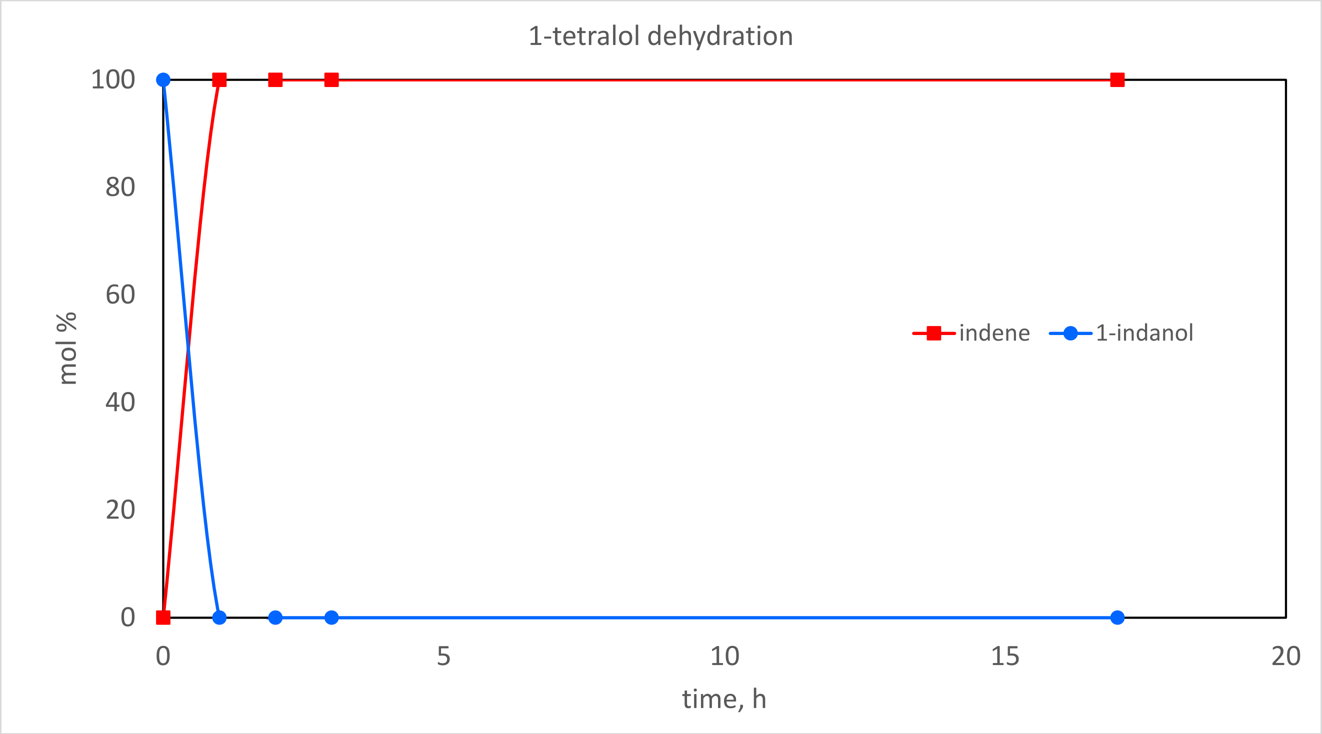

Figure S25.1 1-methylcyclopentanol dehydration by **2a** at 120°C in toluene-d_8

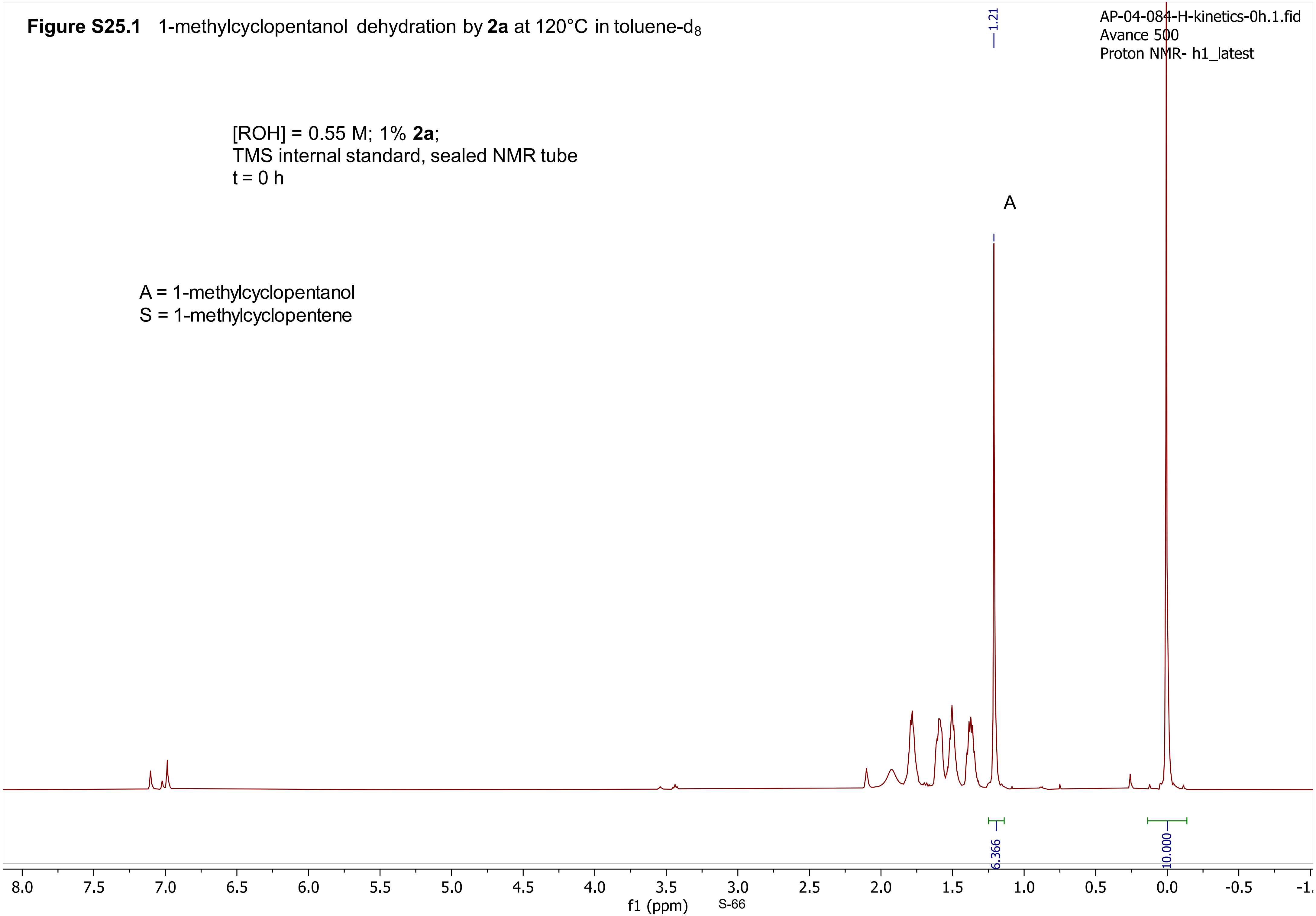

Figure S25.2 1-methylcyclopentanol dehydration by **2a** at 120°C in toluene-d_8

[ROH] = 0.55 M; 1% **2a**;
TMS internal standard, sealed NMR tube
t = 1 h

A = 1-methylcyclopentanol
S = 1-methylcyclopentene

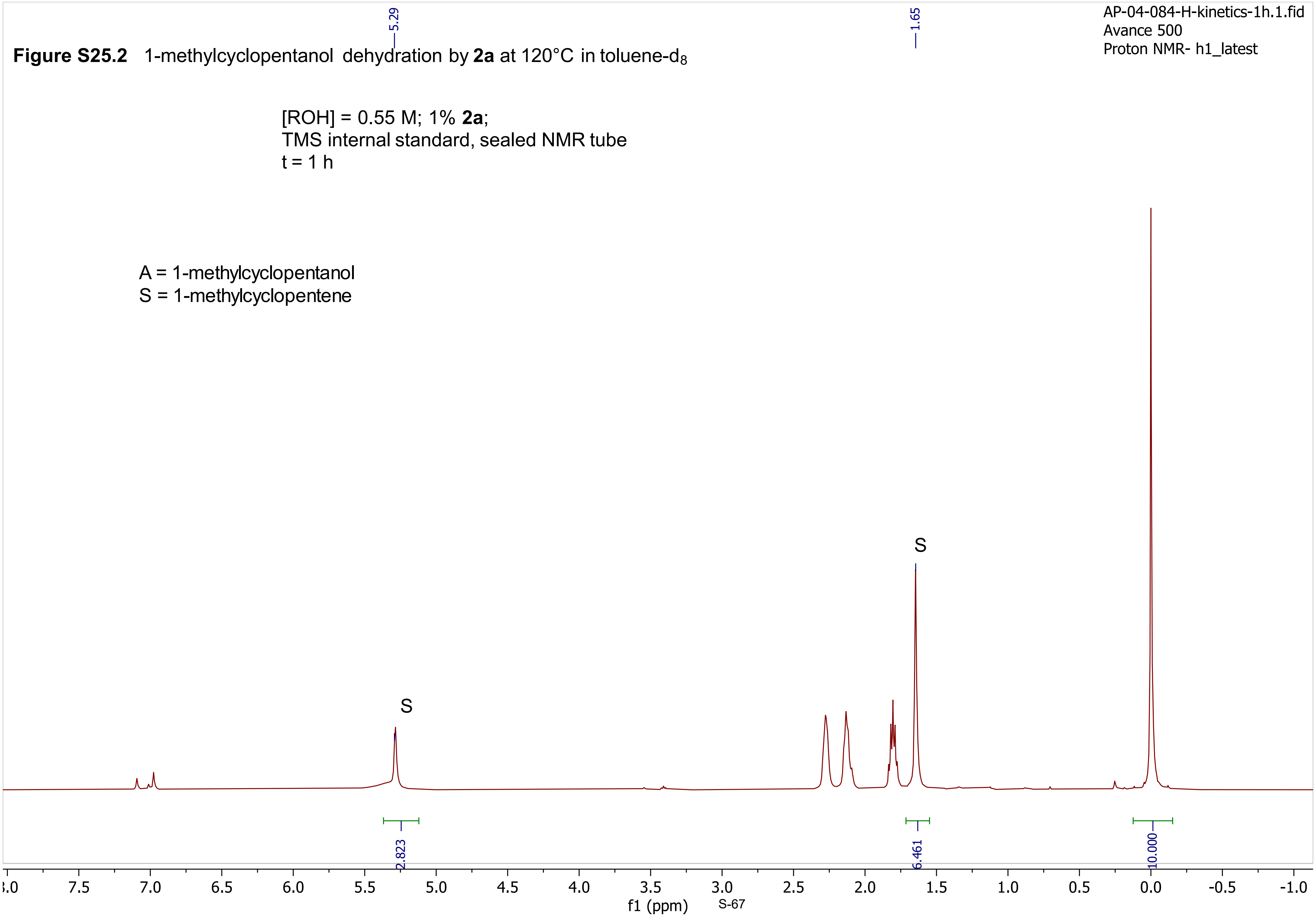

Figure S25.3 1-Methylcyclopentanol Dehydration

AP-04-084

Reaction conditions: [1-Mecyclopentanol] = 0.55 M; toluene-d8; 1% catalyst **2a**; 120 °C.

Sealed NMR tube

time (h)	area 1-MeCypOH(3H)	area 1-Mecyclopentene	area ether1(2H)	area ether2(2H)	area naphth-dimer(1H)	total weighted area
0	6.397	0.000	0.000	0.000	0.000	2.132
1	0.000	2.823	0.000	0.000	0.000	2.823
2	0.000	3.316	0.000	0.000	0.000	3.316
3	0.000	4.231	0.000	0.000	0.000	4.231
	δ 1.65	δ 5.29				

time (h)	mol% 1-MeCypOH(3H)	mol% 1-Mecyclopentene	mol% ether1(2H)	mol% ether2(2H)	mol% naphth-dimer(1H)	mol% total
0	100.000	0.000	0.000	0.000	0.000	100.000
1	0.000	100.000	0.000	0.000	0.000	100.000
2	0.000	100.000	0.000	0.000	0.000	100.000
3	0.000	100.000	0.000	0.000	0.000	100.000

Area TMS set to area 10.00.

mol% = area/#H/total area* 100%

no ethers or dimer seen;

1-MeCypOH area 3H; Mecyclopentene area 1H

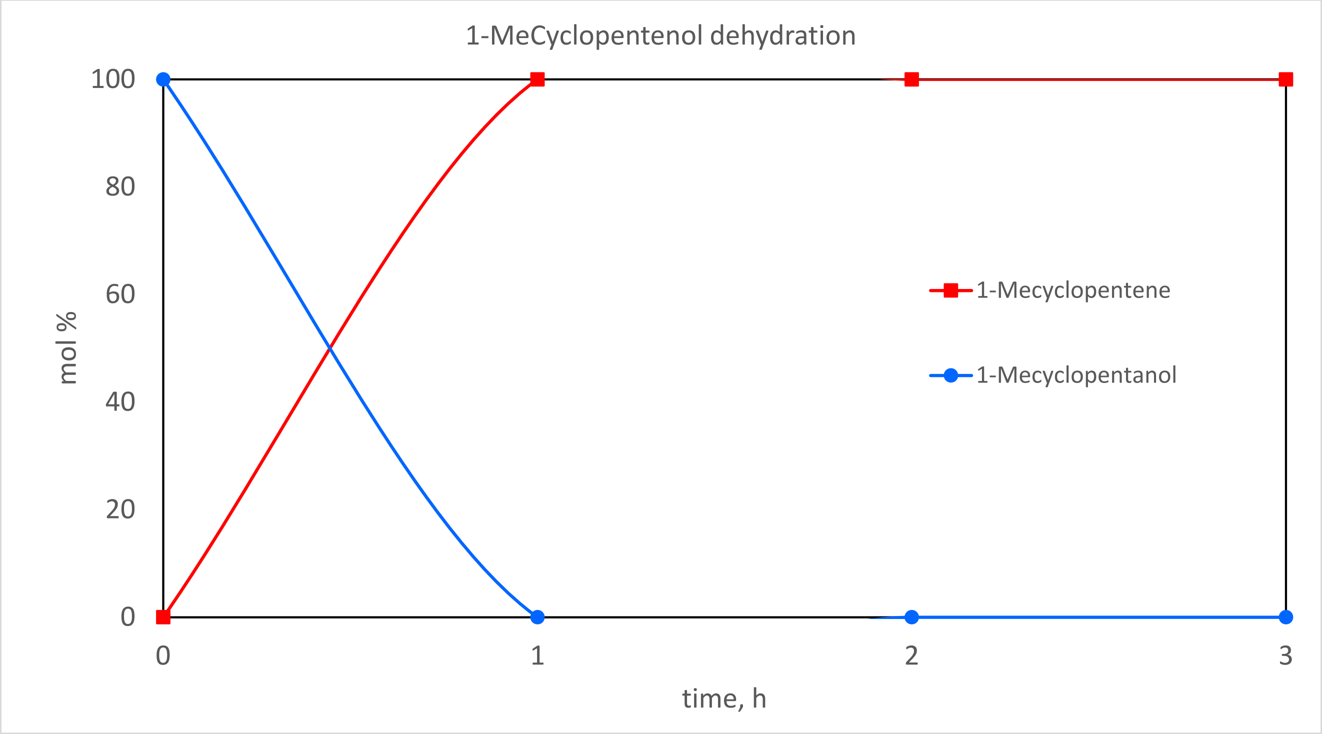

Figure S-26. Dehydration of 1-phenylethanol (0.55 M) by **2a** in toluene at 120 °C - recycle 2nd time
J-Young NMR tube

time, h	%alcohol	%styrene	%ether1	%ether2	%styrene-dimer
0	100.00	0.00	0.00	0.00	0.00
2	60.50	12.61	13.45	13.45	0.00
4	39.33	25.10	17.15	18.41	0.00
18	14.55	54.55	12.36	18.55	0.00
37	7.53	73.97	6.16	12.33	0.00

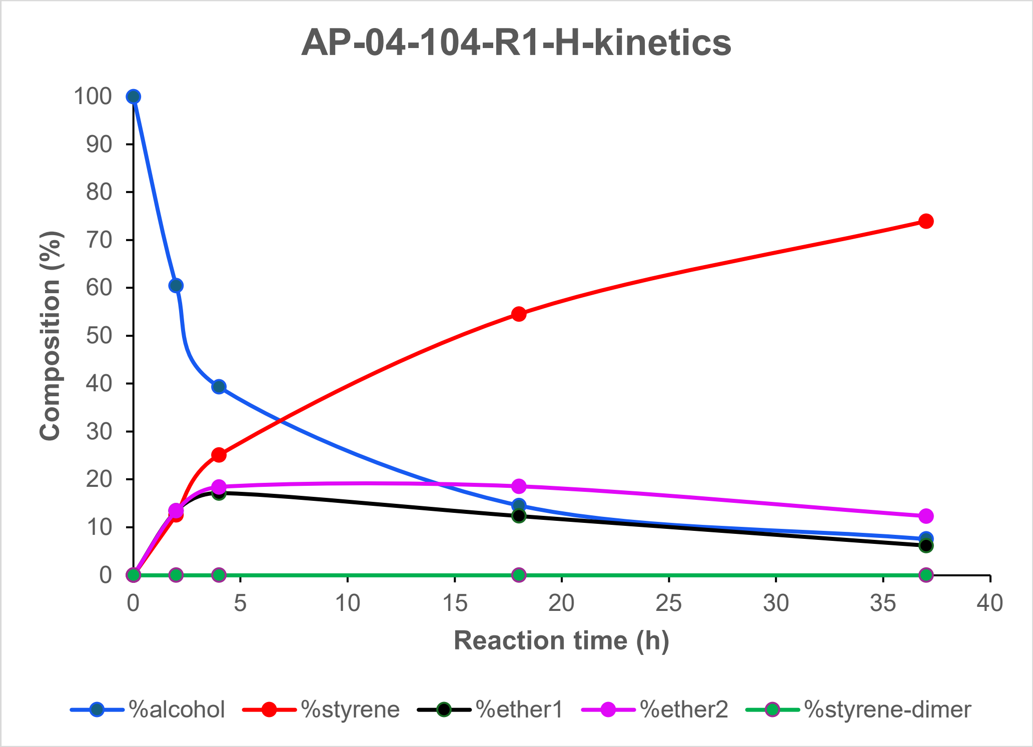

Figure S27. Dehydration of 1-Phenylethanol by $Zn(FOX\text{-}L2)(OTf)_2$

CW-01-047

Time (hrs)	Styrene (%)	1-phenylethanol (%)	Ether 1 (%)	Ether 2 (%)	Styrene Dimer (%)
0	0.000	100.000	0.000	0.000	0.000
2.5	0.087	95.863	2.457	1.593	0.000
3.5	0.706	92.239	3.893	3.162	0.000
4.5	0.422	84.434	9.490	5.654	0.000
25	15.373	24.534	28.416	31.677	0.000
28	13.452	21.447	31.726	33.376	0.000
48.5	25.289	11.297	28.241	35.173	0.000
52	25.677	8.258	27.871	38.194	0.000
120	95.651	0.109	0.178	3.858	0.204

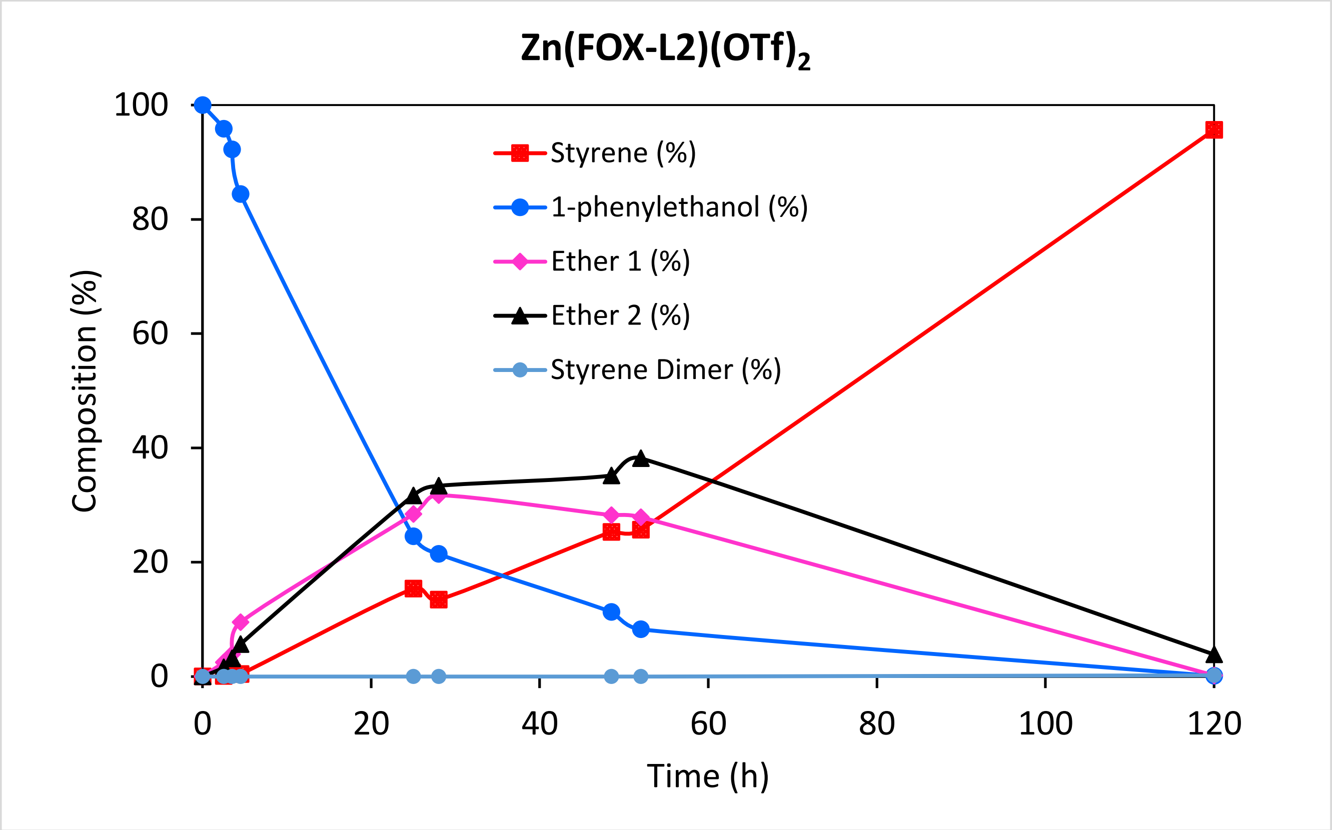

J-Young NMR tube

Figure S28. Dehydration of 1-Phenylethanol by Zn(FOX-L3)$(OTf)_2$ CW-01-055

Time (hours)	Styrene (%)	1-phenylethanol (%)	Ether 1 (%)	Ether 2 (%)	Styrene Dimer (%)
0	0.000	100.000	0.000	0.000	0.000
1	0.107	94.996	3.919	0.978	0.000
2	5.223	90.849	0.225	3.703	0.000
4.5	0.213	95.945	2.194	1.647	0.000
6	0.090	92.898	4.912	2.099	0.000
20	2.584	74.614	11.661	11.141	0.000
26	3.714	62.516	18.482	15.288	0.000
27	4.106	59.740	18.056	18.098	0.000
36	15.731	17.075	30.830	36.364	0.000
48.5	20.288	15.448	28.836	35.427	0.000
51	21.739	13.696	28.913	35.652	0.000
71	35.507	2.174	23.913	38.406	0.000

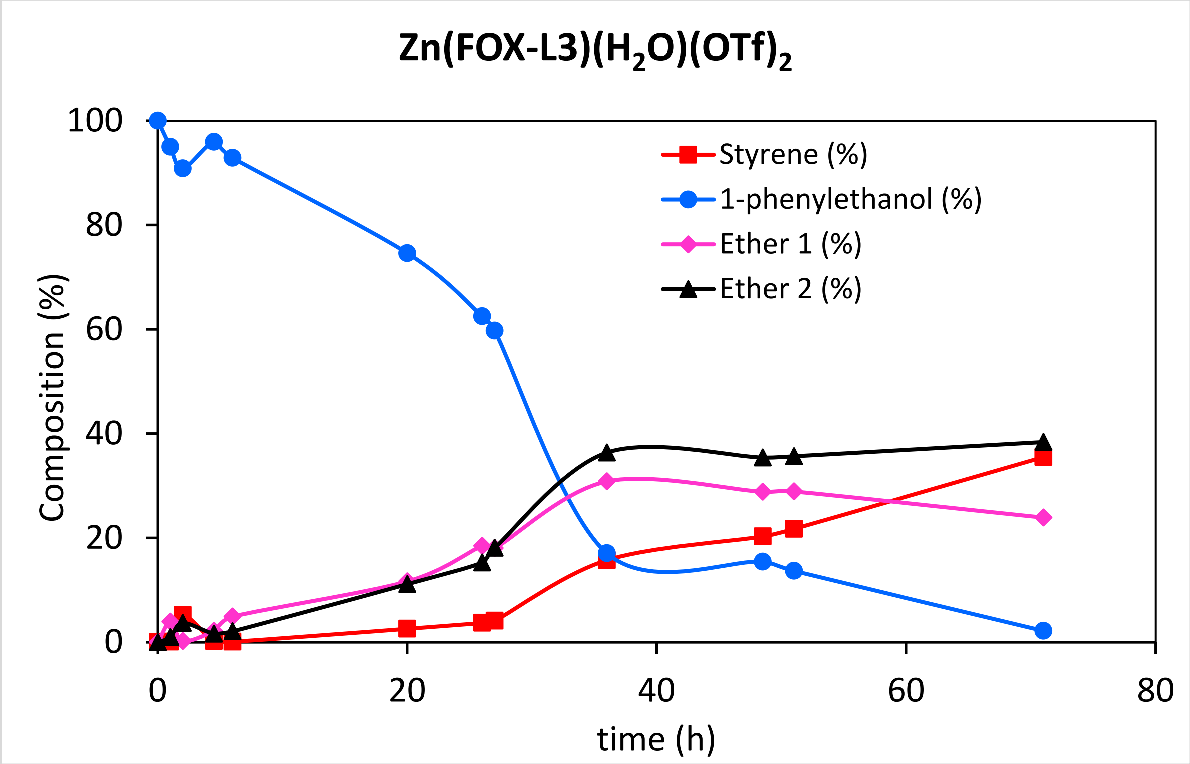

J-Young NMR tube

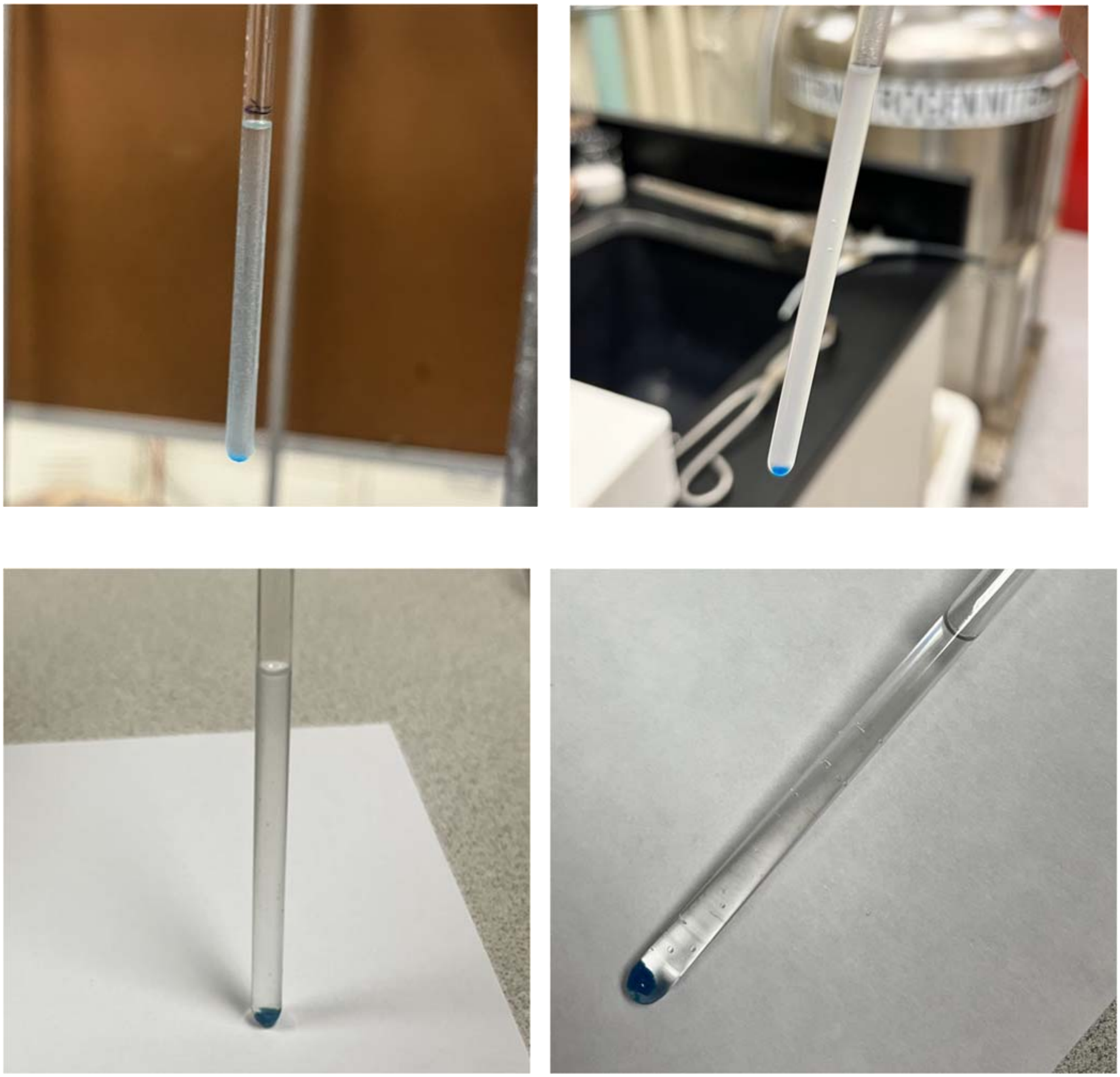

Figure S29. Photos of dehydration of 1-phenylethanol by **2a**. Sample before heating (upper left). Sample after heating for 18 h upon removal from heating block (upper right). Sample after heating for 18 h and cooling to RT (lower left). Close up os sample after heating for 18 h and cooling to RT (lower right). Note the droplets of water, immicible with the toluene.

Figure S30. Evans method 19F NMR spectrum of **2a**

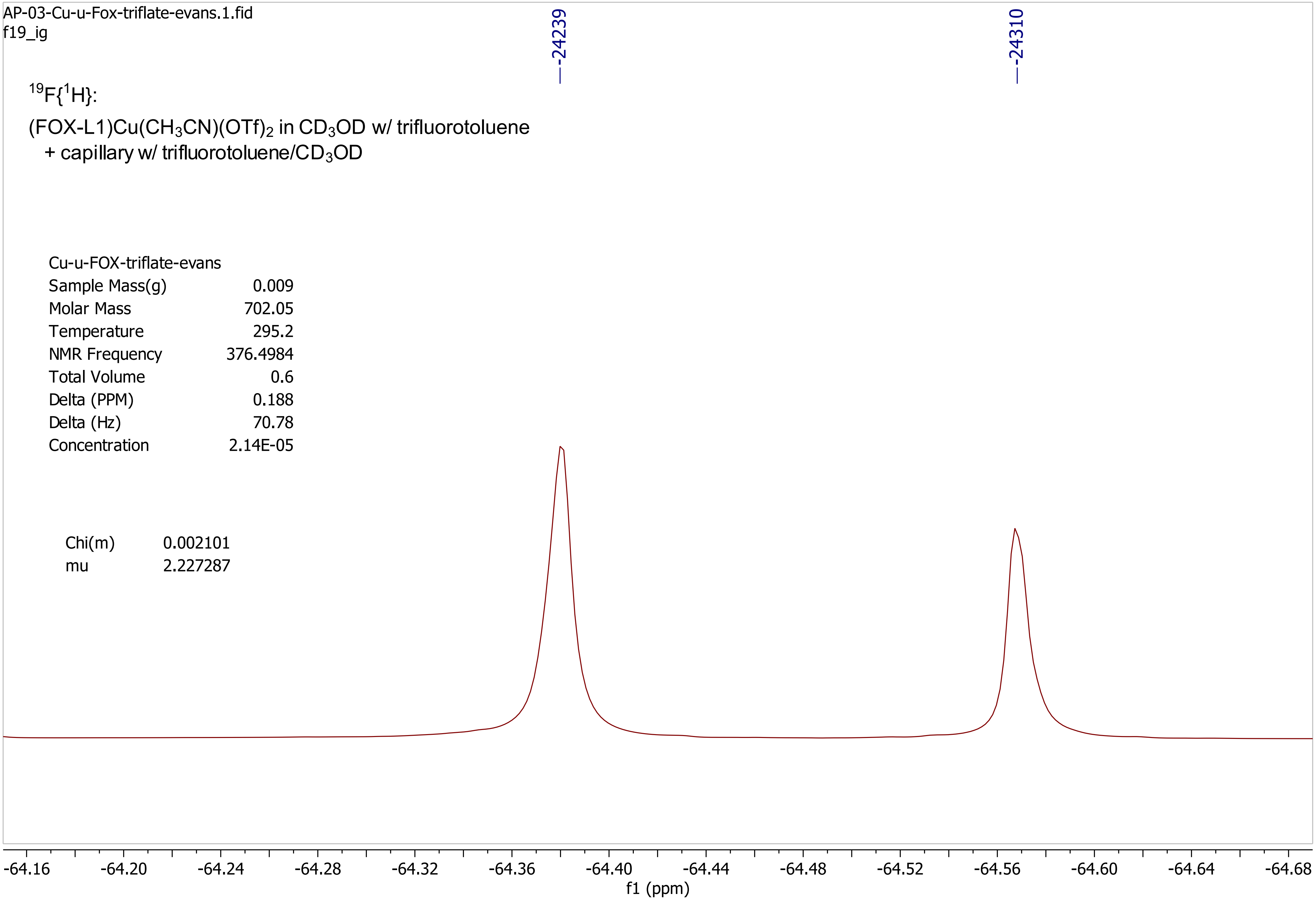

Figure S-31. ^{1}H NMR of (^{meso}FOX-L1)$ZnBr_2$ in DMSO-d_6

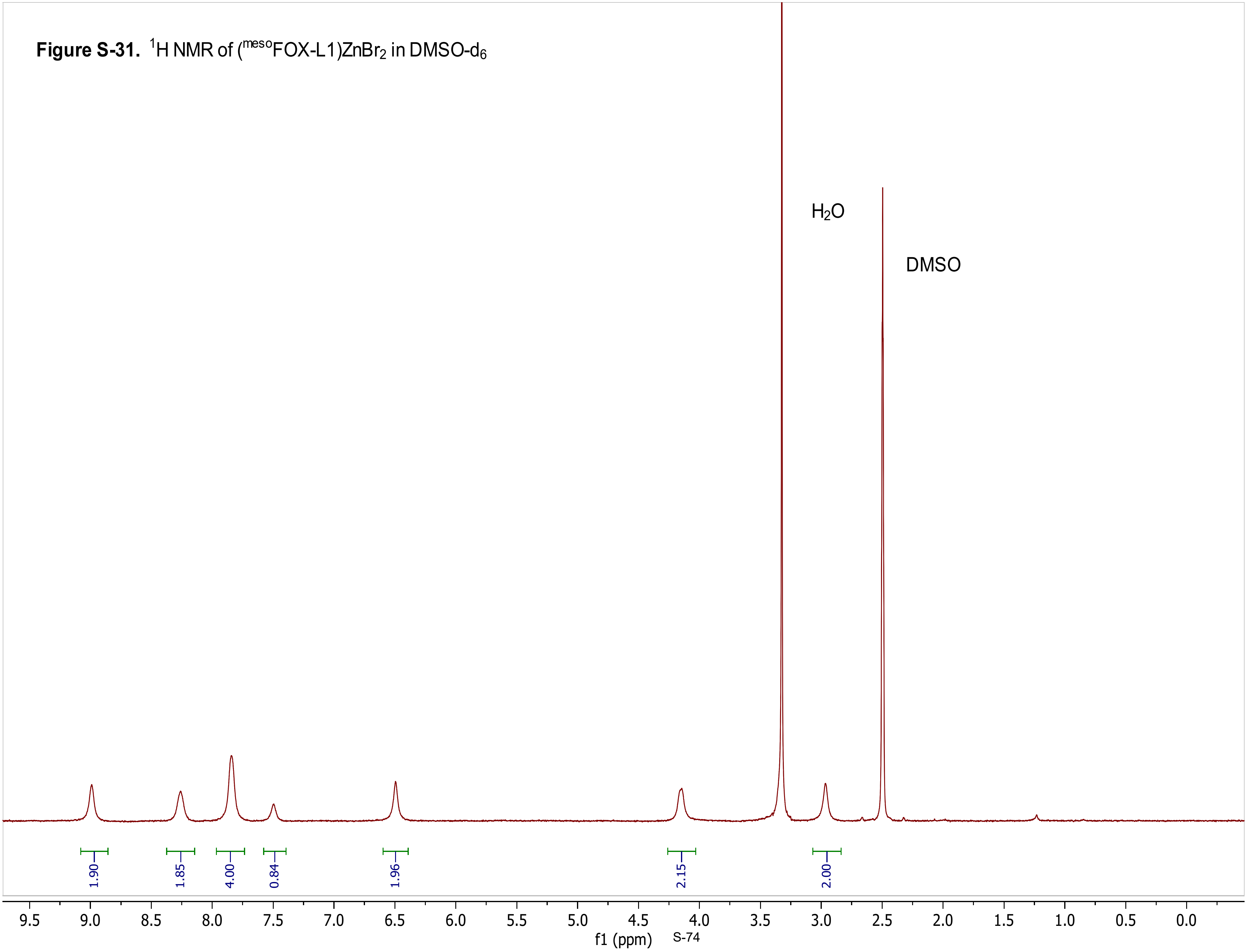

Figure S-32. ^{1}H NMR of (^{meso}FOX-L1)Zn(OTf)$_2$ in DMSO-d$_6$

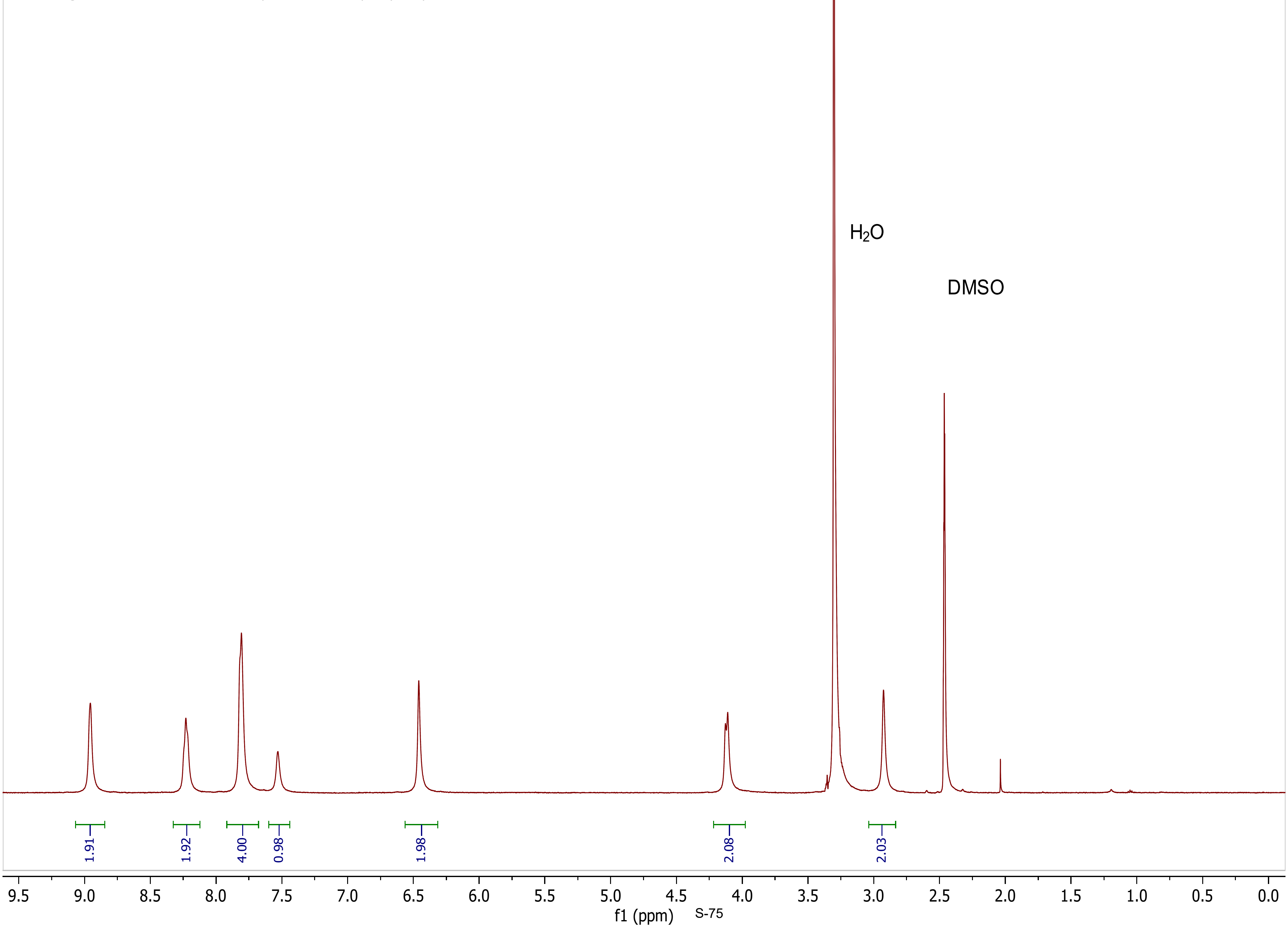

Figure S-33. ^{1}H NMR of (^{meso}FOX-L2)$ZnBr_2$ in DMSO-d$_6$

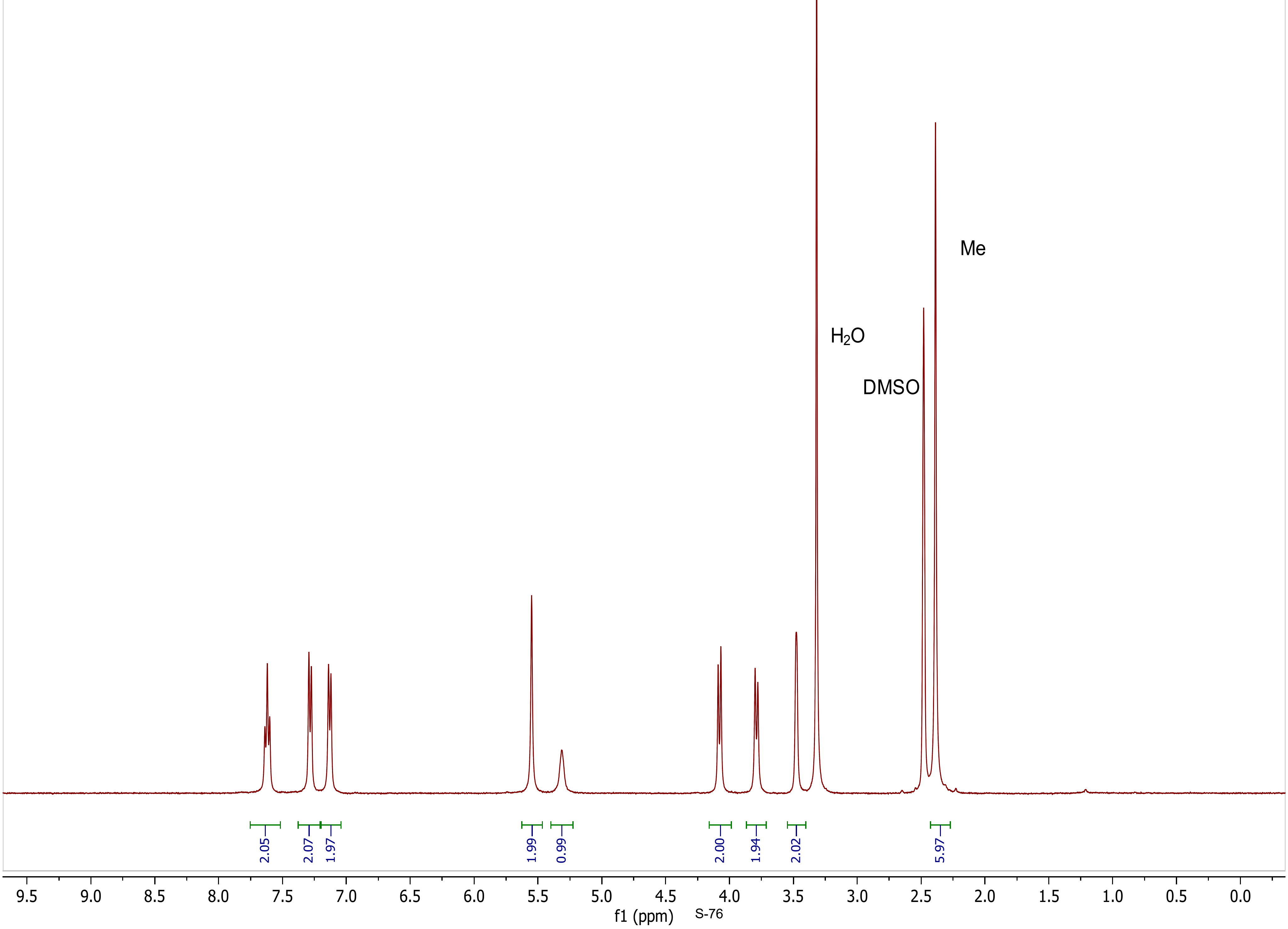

Figure S-34. ${}^{1}H$ NMR of (^{meso}FOX-L2)$Zn(OTf)_2$ in DMSO-d_6

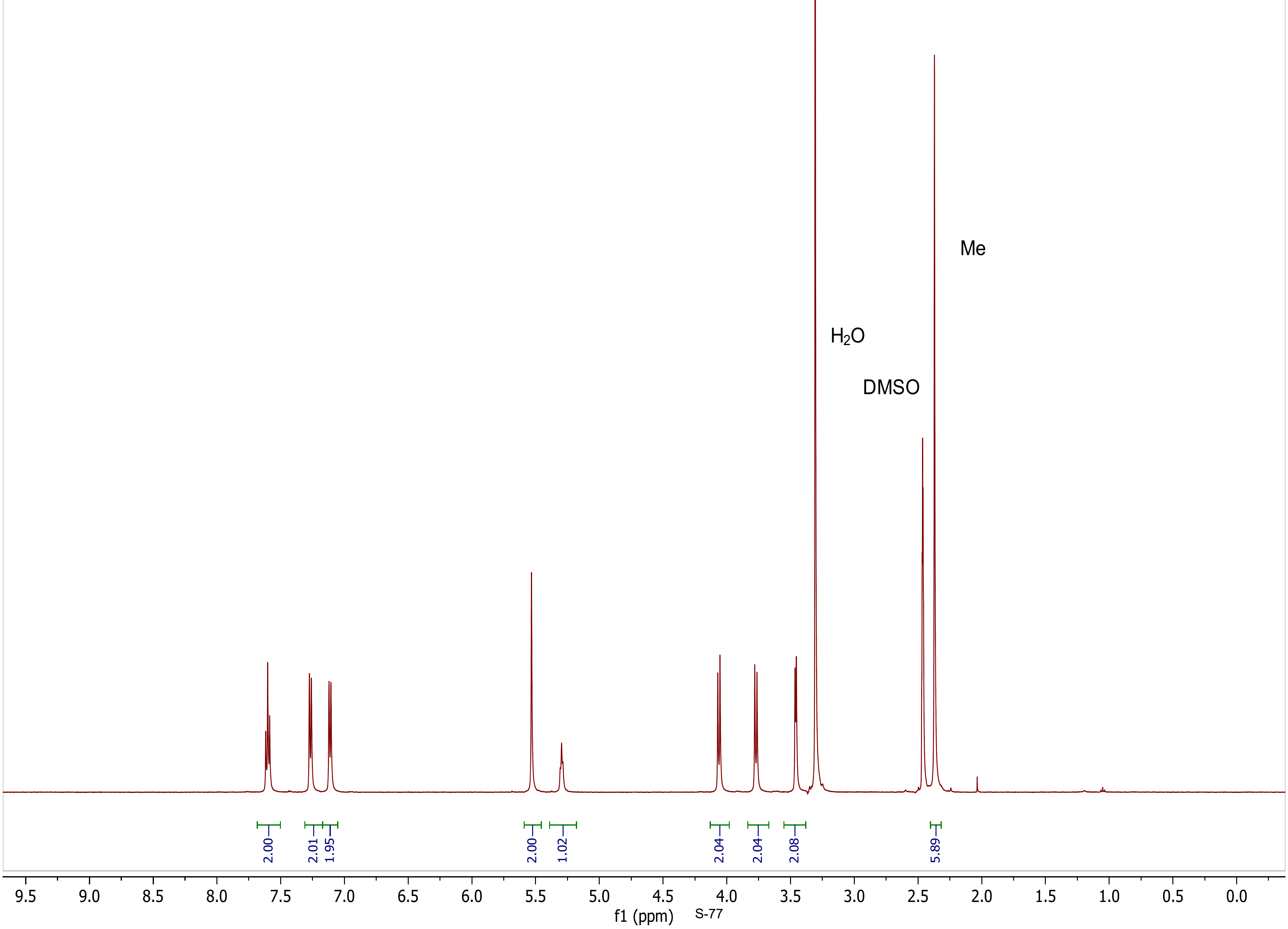

Figure S-35. ^{1}H NMR of (^{meso}FOX-L3)$ZnBr_2$ in DMSO-d_6

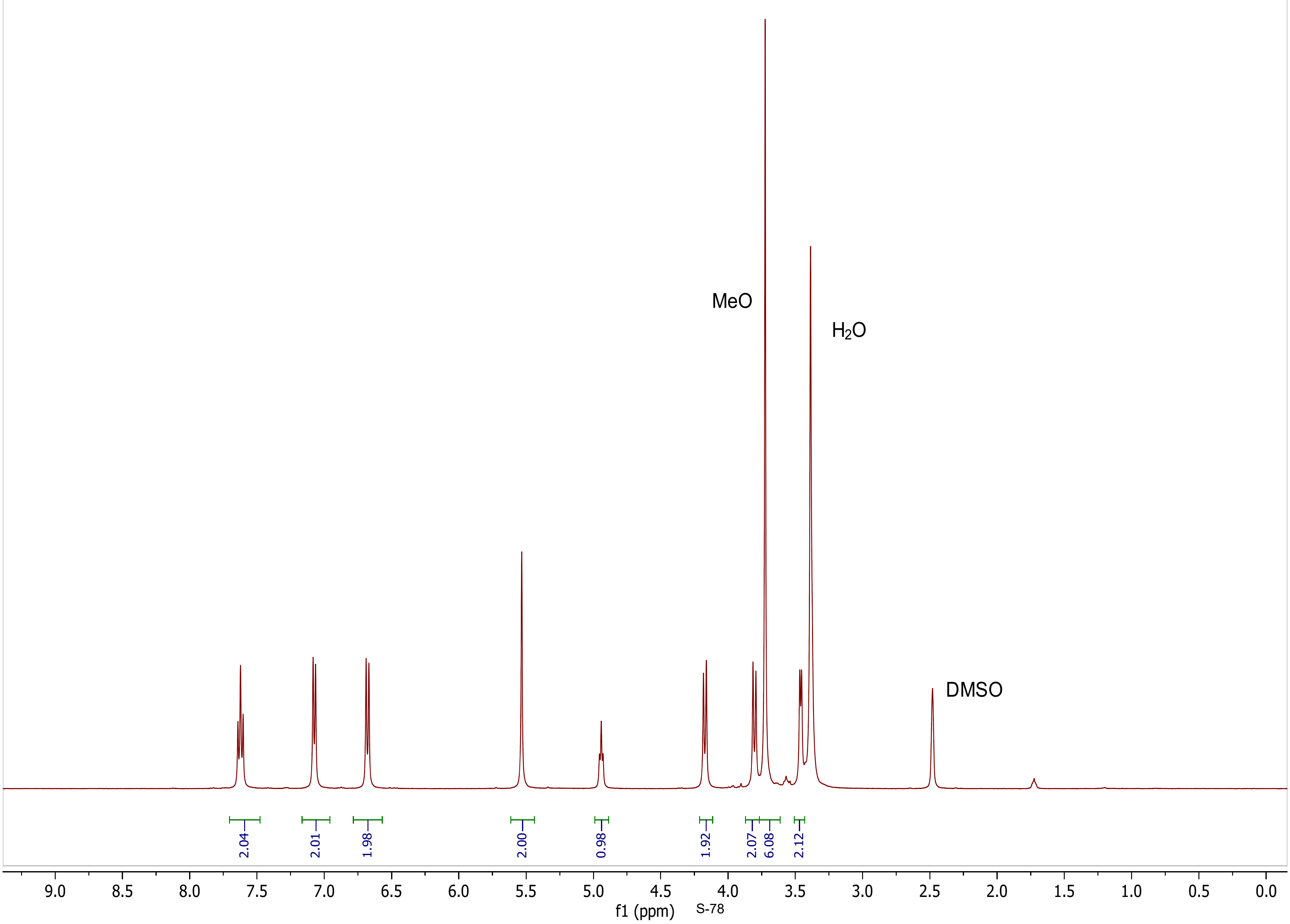

Figure S-36. ^{1}H NMR of (^{meso}FOX-L3)Zn(OTf)$_2$ in DMSO-d$_6$

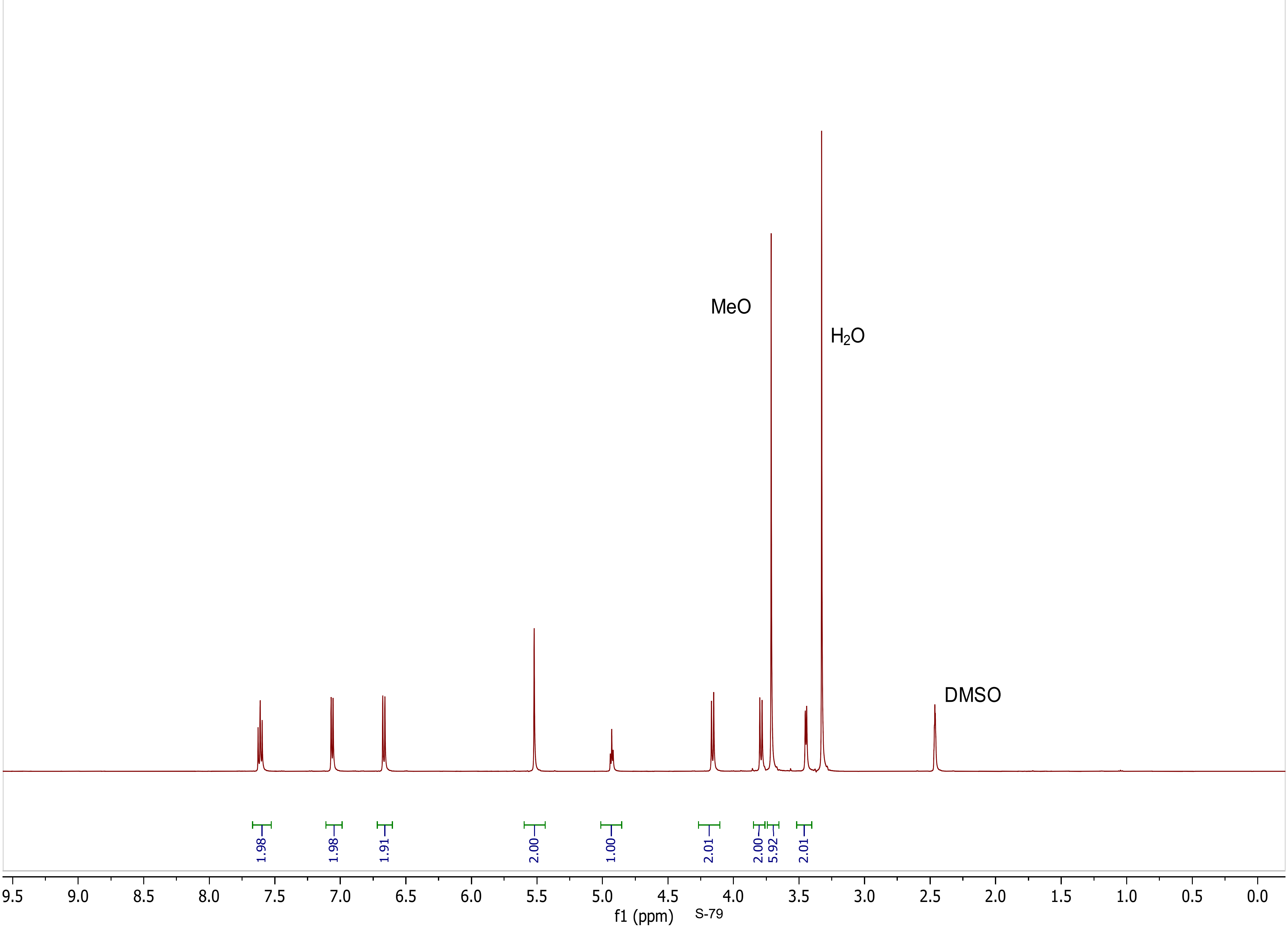

REFERENCE NUMBER: jonap15

1a

CRYSTAL STRUCTURE REPORT

$C_{16} H_{19} Br_2 Cu N_3 O_4$

or

$[(\kappa^4\text{-L})Cu(Br)][Br] \cdot H_2O$

Report prepared for:

A. Panda, Prof. W. Jones

April 29, 2023

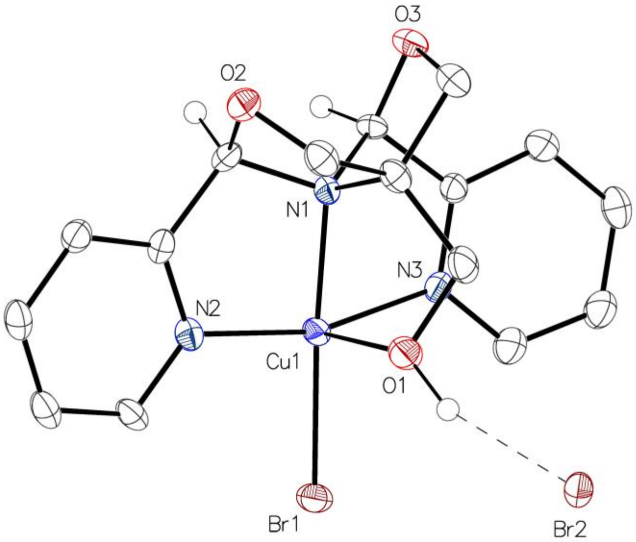

William W. Brennessel

X-ray Crystallographic Facility

Department of Chemistry, University of Rochester

120 Trustee Road

Rochester, NY 14627

Data collection

A crystal (0.138 x 0.101 x 0.033 mm^3) was placed onto a thin glass optical fiber or a nylon loop and mounted on a Rigaku XtaLAB Synergy-S Dualflex diffractometer equipped with a HyPix-6000HE HPC area detector for data collection at 100.00(10) K. A preliminary set of cell constants and an orientation matrix were calculated from a small sampling of reflections.[1] A short pre-experiment was run, from which an optimal data collection strategy was determined. The full data collection was carried out using a PhotonJet (Cu) X-ray source with frame times of 0.05 and 0.14 seconds and a detector distance of 34.0 mm. Series of frames were collected in 0.50º steps in ω at different 2θ, κ, and ϕ settings. After the intensity data were corrected for absorption, the final cell constants were calculated from the xyz centroids of 23362 strong reflections from the actual data collection after integration.[1] See Table S 1 for additional crystal and refinement information.

Structure solution and refinement

The structure was solved using SHELXT[2] and refined using SHELXL.[3] The space group *Cc* was determined based on systematic absences and intensity statistics. Most or all non-hydrogen atoms were assigned from the solution. Full-matrix least squares / difference Fourier cycles were performed which located any remaining non-hydrogen atoms. All non-hydrogen atoms were refined with anisotropic displacement parameters. The hydrogen atom on O1 was found from the difference Fourier map and refined freely. The hydrogen atoms on water molecule O4 were found from the difference Fourier map, given an idealized length (0.85 Å), and then refined with a riding model. All other hydrogen atoms were placed in ideal positions and refined as riding atoms with relative isotropic displacement parameters. The final full matrix least squares refinement converged to $R1 = 0.0284$ (F^2, $I > 2\sigma(I)$) and $wR2 = 0.0768$ (F^2, all data).

Structure description

The structure is the one suggested. The asymmetric unit contains one monocationic copper complex, one bromide anion, and one water solvent molecule of crystallization, all in general positions. The molecules are packed in sheets via O-H…Br hydrogen bonding (see figure and Table S 7). The distance between the metal center and the symmetry equivalent of atom Br2, which is found in the vicinity of the open coordination site, is 3.2527(10) Å.

Structure manipulation and figure generation were performed using Olex2.[4] Unless noted otherwise all structural diagrams containing anisotropic displacement ellipsoids are drawn at the 50 % probability level.

Data collection, structure solution, and structure refinement were conducted at the X-ray Crystallographic Facility, B04 Hutchison Hall, Department of Chemistry, University of Rochester. The instrument was purchased with funding from NSF MRI program grant CHE-1725028. All publications arising from this report MUST either 1) include William W. Brennessel as a coauthor or 2) acknowledge William W. Brennessel and the X-ray Crystallographic Facility of the Department of Chemistry at the University of Rochester.

[1] *CrysAlisPro*, version 171.42.85a; Rigaku Corporation: Oxford, UK, 2023.

[2] Sheldrick, G. M. *SHELXT*, version 2018/2; *Acta. Crystallogr.* **2015**, *A71*, 3-8.

[3] Sheldrick, G. M. *SHELXL*, version 2019/2; *Acta. Crystallogr.* **2015**, *C71*, 3-8.

[4] Dolomanov, O. V.; Bourhis, L. J.; Gildea, R. J.; Howard, J. A. K.; Puschmann, H. *Olex2*, version 1.5; *J. Appl. Cryst.* **2009**, *42*, 339-341.

Some equations of interest:

$$R_{int} = \Sigma |F_o^2 - \langle F_o^2 \rangle| / \Sigma|F_o^2|$$

$$R1 = \Sigma||F_o|-|F_c|| / \Sigma|F_o|$$

$$wR2 = [\Sigma[w(F_o^2-F_c^2)^2] / \Sigma [w(F_o^2)^2]]^{1/2}$$

$$\text{where } w = 1 / [\sigma^2 (F_o^2) + (aP)^2 + bP] \text{ and}$$

$$P = 1/3 \max (0, F_o^2) + 2/3 F_c^2$$

$$\text{GOF} = S = [\Sigma[w(F_o^2-F_c^2)^2] / (m-n)]^{1/2}$$

where m = number of reflections and n = number of parameters

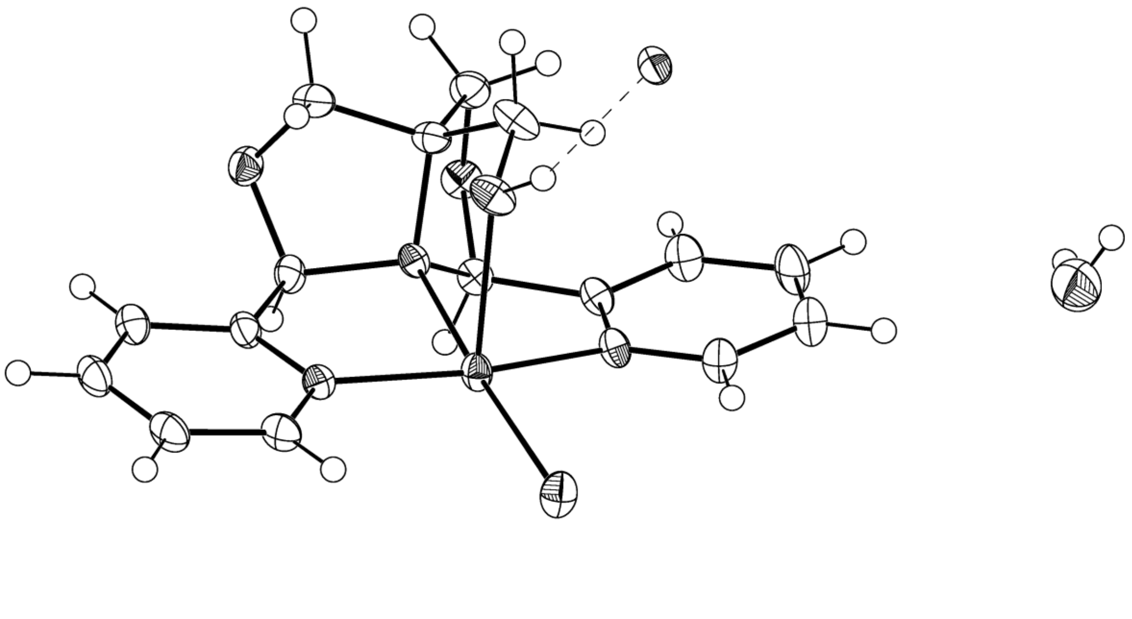

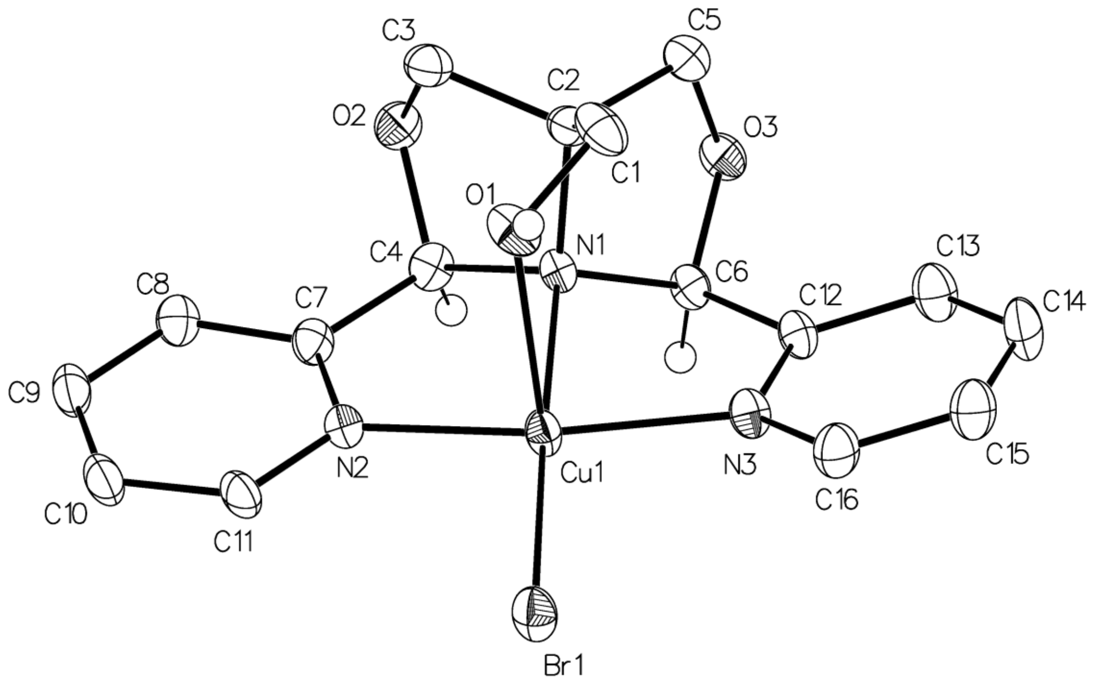
C3
C5
C2
O2
O3
C1
O1
N1
C4
C6
C13
C8
C7
C12
C14
C9
N2
Cu1
N3
C15
C16
C10
C11
Br1

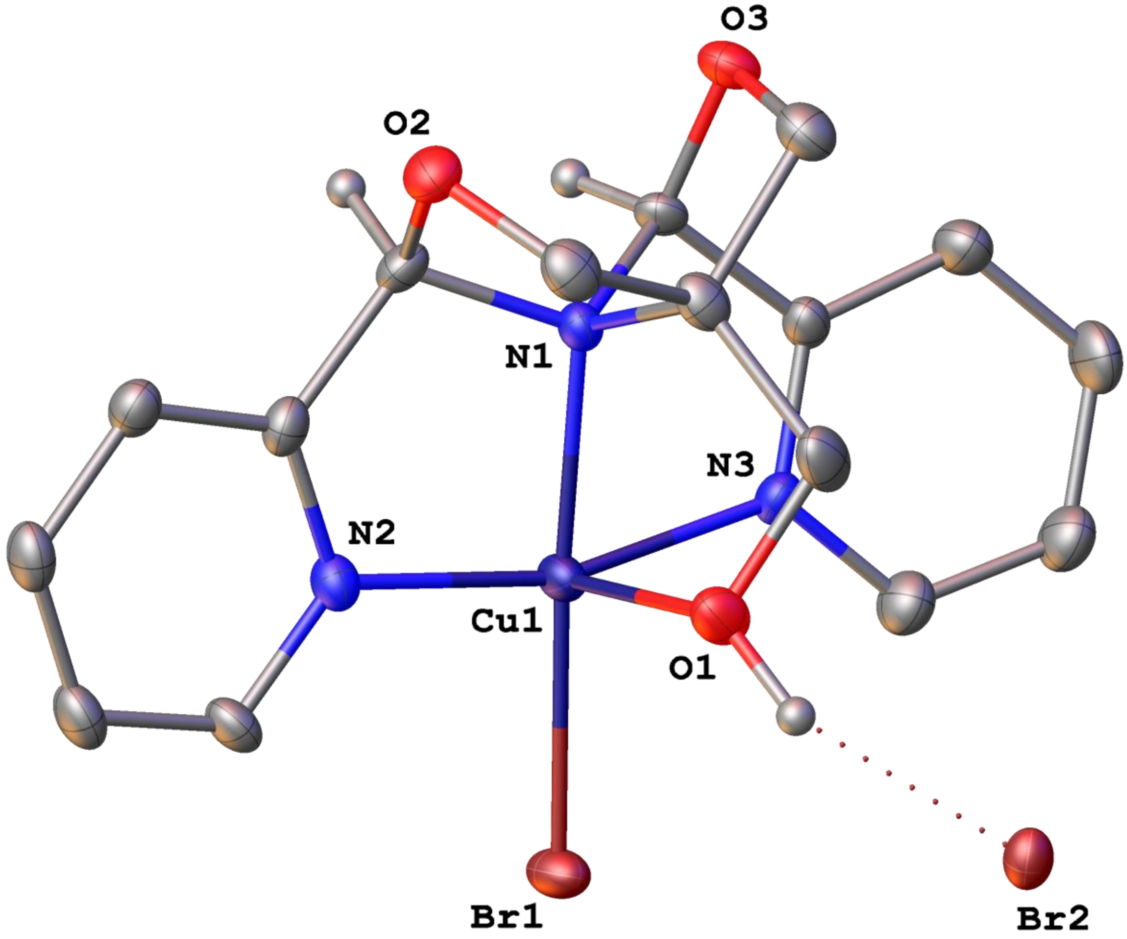
O3
O2
N1
N3
N2
Cu1
O1
Br1
Br2

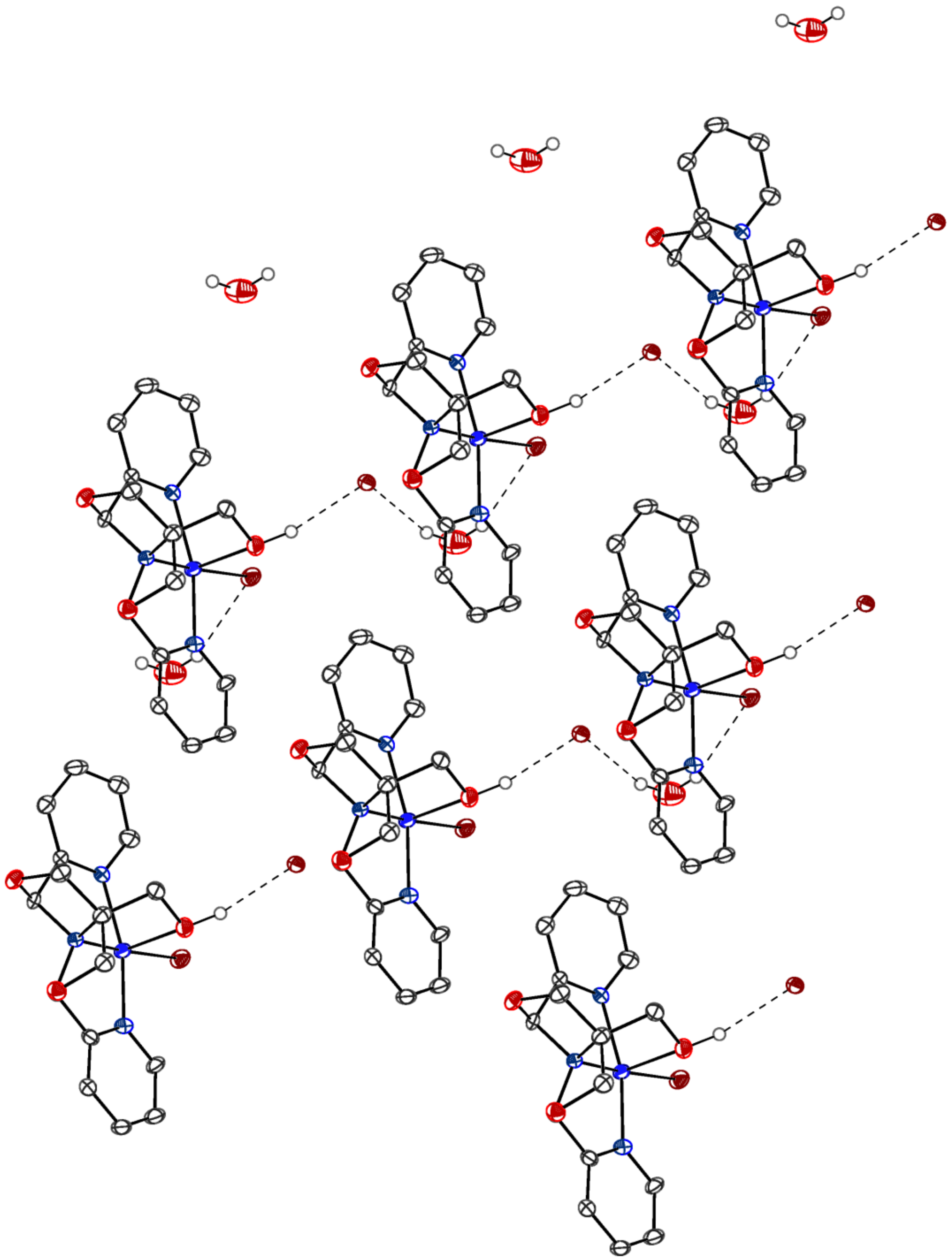

Table S1. Crystal data and structure refinement for jonap15.

Identification code	jonap15	
Empirical formula	C16 H19 Br2 Cu N3 O4	
Formula weight	540.70	
Temperature	100.00(10) K	
Wavelength	1.54184 Å	
Crystal system	monoclinic	
Space group	*Cc*	
Unit cell dimensions	a = 14.00250(10) Å	α = 90°
	b = 9.82590(10) Å	β = 112.3660(10)°
	c = 14.59860(10) Å	γ = 90°
Volume	1857.48(3) $Å^3$	
Z	4	
Density (calculated)	1.933 Mg/m^3	
Absorption coefficient	6.946 mm^{-1}	
F(000)	1068	
Crystal color, morphology	blue, plate	
Crystal size	0.138 x 0.101 x 0.033 mm^3	
Theta range for data collection	5.652 to 80.317°	
Index ranges	$-17 \leq h \leq 17$, $-12 \leq k \leq 12$, $-17 \leq l \leq 18$	
Reflections collected	30360	
Independent reflections	3879 [*R*(int) = 0.0426]	
Observed reflections	3864	
Completeness to theta = 74.504°	100.0%	
Absorption correction	Multi-scan	
Max. and min. transmission	1.00000 and 0.69849	
Refinement method	Full-matrix least-squares on F^2	
Data / restraints / parameters	3879 / 2 / 239	
Goodness-of-fit on F^2	1.123	
Final *R* indices [I>2sigma(I)]	$R1$ = 0.0284, $wR2$ = 0.0767	
R indices (all data)	$R1$ = 0.0285, $wR2$ = 0.0768	
Absolute structure parameter	-0.032(14)	
Largest diff. peak and hole	0.500 and -0.662 e.$Å^{-3}$	

Table S2. Atomic coordinates (x 10^4) and equivalent isotropic displacement parameters ($Å^2$x 10^3) for jonap15. U_{eq} is defined as one third of the trace of the orthogonalized U_{ij} tensor.

	x	y	z	U_{eq}
Br1	5992(1)	1980(1)	7245(1)	25(1)
Cu1	5731(1)	2160(1)	5525(1)	18(1)
O1	4073(3)	3247(4)	4849(4)	25(1)
O2	4470(3)	1315(4)	2599(3)	22(1)
O3	6050(3)	3762(4)	3057(3)	22(1)
N1	5474(4)	2430(5)	4050(4)	16(1)
N2	5061(4)	371(5)	5062(4)	18(1)
N3	6491(4)	3920(5)	5637(4)	18(1)
C1	4155(5)	4135(6)	4108(5)	26(1)
C2	4563(4)	3326(6)	3451(4)	19(1)
C3	3813(5)	2284(6)	2793(5)	22(1)
C4	5259(4)	1082(6)	3531(4)	18(1)
C5	5017(5)	4248(6)	2855(5)	22(1)
C6	6373(4)	3173(6)	4003(4)	16(1)
C7	4895(4)	68(6)	4113(4)	19(1)
C8	4393(5)	-1113(6)	3660(5)	21(1)
C9	4062(5)	-2017(6)	4213(5)	23(1)
C10	4258(5)	-1722(6)	5195(5)	24(1)
C11	4749(5)	-513(6)	5595(5)	21(1)
C12	6672(4)	4247(6)	4819(4)	18(1)
C13	7045(5)	5517(6)	4704(5)	25(1)
C14	7203(5)	6485(6)	5439(5)	30(1)
C15	7010(5)	6144(7)	6278(5)	27(1)
C16	6665(5)	4851(6)	6351(5)	25(1)
Br2	2673(1)	5387(1)	5478(1)	20(1)
O4	7335(5)	9055(6)	7410(4)	47(1)

Table S3. Bond lengths [Å] and angles [°] for jonap15.

Br(1)-Cu(1)	2.4023(10)	C(9)-H(9)	0.9500
Cu(1)-O(1)	2.399(4)	C(9)-C(10)	1.384(10)
Cu(1)-N(1)	2.060(5)	C(10)-H(10)	0.9500
Cu(1)-N(2)	1.986(5)	C(10)-C(11)	1.385(9)
Cu(1)-N(3)	2.004(5)	C(11)-H(11)	0.9500
O(1)-H(1)	0.98(9)	C(12)-C(13)	1.388(8)
O(1)-C(1)	1.428(8)	C(13)-H(13)	0.9500
O(2)-C(3)	1.426(8)	C(13)-C(14)	1.387(9)
O(2)-C(4)	1.407(7)	C(14)-H(14)	0.9500
O(3)-C(5)	1.443(7)	C(14)-C(15)	1.392(10)
O(3)-C(6)	1.404(7)	C(15)-H(15)	0.9500
N(1)-C(2)	1.523(7)	C(15)-C(16)	1.378(9)
N(1)-C(4)	1.500(7)	C(16)-H(16)	0.9500
N(1)-C(6)	1.479(7)	O(4)-H(4A)	0.8500
N(2)-C(7)	1.347(8)	O(4)-H(4B)	0.8499
N(2)-C(11)	1.346(8)	O(1)-Cu(1)-Br(1)	101.63(12)
N(3)-C(12)	1.350(8)	N(1)-Cu(1)-Br(1)	176.60(14)
N(3)-C(16)	1.338(8)	N(1)-Cu(1)-O(1)	75.89(18)
C(1)-H(1A)	0.9900	N(2)-Cu(1)-Br(1)	98.90(15)
C(1)-H(1B)	0.9900	N(2)-Cu(1)-O(1)	89.80(18)
C(1)-C(2)	1.516(9)	N(2)-Cu(1)-N(1)	83.5(2)
C(2)-C(3)	1.519(8)	N(2)-Cu(1)-N(3)	163.5(2)
C(2)-C(5)	1.550(8)	N(3)-Cu(1)-Br(1)	96.50(15)
C(3)-H(3A)	0.9900	N(3)-Cu(1)-O(1)	92.87(18)
C(3)-H(3B)	0.9900	N(3)-Cu(1)-N(1)	81.38(19)
C(4)-H(4)	1.0000	Cu(1)-O(1)-H(1)	126(5)
C(4)-C(7)	1.519(8)	C(1)-O(1)-Cu(1)	103.9(3)
C(5)-H(5A)	0.9900	C(1)-O(1)-H(1)	108(5)
C(5)-H(5B)	0.9900	C(4)-O(2)-C(3)	104.2(4)
C(6)-H(6)	1.0000	C(6)-O(3)-C(5)	105.1(4)
C(6)-C(12)	1.526(8)	C(2)-N(1)-Cu(1)	116.7(4)
C(7)-C(8)	1.387(8)	C(4)-N(1)-Cu(1)	109.6(3)
C(8)-H(8)	0.9500	C(4)-N(1)-C(2)	104.8(4)
C(8)-C(9)	1.393(8)	C(6)-N(1)-Cu(1)	107.4(3)

C(6)-N(1)-C(2)	103.3(4)	C(2)-C(5)-H(5B)	110.4
C(6)-N(1)-C(4)	115.1(4)	H(5A)-C(5)-H(5B)	108.6
C(7)-N(2)-Cu(1)	114.8(4)	O(3)-C(6)-N(1)	106.7(4)
C(11)-N(2)-Cu(1)	126.2(4)	O(3)-C(6)-H(6)	110.4
C(11)-N(2)-C(7)	119.0(5)	O(3)-C(6)-C(12)	111.9(5)
C(12)-N(3)-Cu(1)	113.9(4)	N(1)-C(6)-H(6)	110.4
C(16)-N(3)-Cu(1)	125.9(4)	N(1)-C(6)-C(12)	106.9(4)
C(16)-N(3)-C(12)	119.1(5)	C(12)-C(6)-H(6)	110.4
O(1)-C(1)-H(1A)	110.0	N(2)-C(7)-C(4)	117.9(5)
O(1)-C(1)-H(1B)	110.0	N(2)-C(7)-C(8)	122.2(6)
O(1)-C(1)-C(2)	108.5(5)	C(8)-C(7)-C(4)	119.9(5)
H(1A)-C(1)-H(1B)	108.4	C(7)-C(8)-H(8)	120.8
C(2)-C(1)-H(1A)	110.0	C(7)-C(8)-C(9)	118.4(6)
C(2)-C(1)-H(1B)	110.0	C(9)-C(8)-H(8)	120.8
N(1)-C(2)-C(5)	103.1(4)	C(8)-C(9)-H(9)	120.4
C(1)-C(2)-N(1)	112.0(5)	C(10)-C(9)-C(8)	119.2(5)
C(1)-C(2)-C(3)	114.6(5)	C(10)-C(9)-H(9)	120.4
C(1)-C(2)-C(5)	112.5(5)	C(9)-C(10)-H(10)	120.4
C(3)-C(2)-N(1)	101.6(4)	C(9)-C(10)-C(11)	119.1(6)
C(3)-C(2)-C(5)	111.9(5)	C(11)-C(10)-H(10)	120.4
O(2)-C(3)-C(2)	103.4(5)	N(2)-C(11)-C(10)	121.9(6)
O(2)-C(3)-H(3A)	111.1	N(2)-C(11)-H(11)	119.0
O(2)-C(3)-H(3B)	111.1	C(10)-C(11)-H(11)	119.0
C(2)-C(3)-H(3A)	111.1	N(3)-C(12)-C(6)	116.0(5)
C(2)-C(3)-H(3B)	111.1	N(3)-C(12)-C(13)	121.8(5)
H(3A)-C(3)-H(3B)	109.0	C(13)-C(12)-C(6)	122.1(5)
O(2)-C(4)-N(1)	106.1(4)	C(12)-C(13)-H(13)	120.7
O(2)-C(4)-H(4)	110.2	C(14)-C(13)-C(12)	118.7(6)
O(2)-C(4)-C(7)	110.0(5)	C(14)-C(13)-H(13)	120.7
N(1)-C(4)-H(4)	110.2	C(13)-C(14)-H(14)	120.4
N(1)-C(4)-C(7)	110.0(4)	C(13)-C(14)-C(15)	119.3(6)
C(7)-C(4)-H(4)	110.2	C(15)-C(14)-H(14)	120.4
O(3)-C(5)-C(2)	106.6(5)	C(14)-C(15)-H(15)	120.6
O(3)-C(5)-H(5A)	110.4	C(16)-C(15)-C(14)	118.7(6)
O(3)-C(5)-H(5B)	110.4	C(16)-C(15)-H(15)	120.6
C(2)-C(5)-H(5A)	110.4	N(3)-C(16)-C(15)	122.4(6)

N(3)-C(16)-H(16)	118.8	H(4A)-O(4)-H(4B)	111.5
C(15)-C(16)-H(16)	118.8		

Table S4. Anisotropic displacement parameters ($Å^2 x 10^3$) for jonap15. The anisotropic displacement factor exponent takes the form: $-2\pi^2[h^2 a^{*2}U_{11} + ... + 2 h k a^* b^* U_{12}]$

	U_{11}	U_{22}	U_{33}	U_{23}	U_{13}	U_{12}
Br1	34(1)	24(1)	19(1)	-2(1)	14(1)	-8(1)
Cu1	23(1)	15(1)	18(1)	-1(1)	10(1)	-4(1)
O1	27(2)	22(2)	31(2)	6(2)	18(2)	6(2)
O2	25(2)	21(2)	19(2)	-3(2)	8(2)	-1(2)
O3	25(2)	24(2)	19(2)	4(2)	12(2)	-2(2)
N1	18(2)	13(2)	18(2)	1(2)	9(2)	-1(2)
N2	19(2)	15(2)	20(2)	1(2)	6(2)	-1(2)
N3	22(2)	15(2)	22(2)	-3(2)	12(2)	0(2)
C1	28(3)	18(3)	37(4)	8(3)	19(3)	7(2)
C2	17(2)	19(3)	21(3)	5(2)	6(2)	1(2)
C3	19(3)	23(3)	24(3)	5(2)	6(2)	2(2)
C4	20(3)	18(3)	18(3)	-5(2)	10(2)	1(2)
C5	24(3)	22(3)	22(3)	4(2)	9(2)	-1(2)
C6	17(2)	18(2)	18(3)	2(2)	11(2)	-1(2)
C7	19(3)	14(2)	23(3)	-3(2)	8(2)	1(2)
C8	21(3)	18(3)	25(3)	-3(2)	11(2)	-1(2)
C9	22(3)	14(3)	33(3)	0(2)	11(2)	-3(2)
C10	26(3)	16(3)	31(3)	5(2)	12(3)	-2(2)
C11	23(3)	19(3)	24(3)	6(2)	12(2)	-2(2)
C12	19(2)	14(2)	23(3)	0(2)	12(2)	0(2)
C13	31(3)	22(3)	27(3)	-1(2)	17(3)	-5(2)
C14	37(3)	17(3)	37(4)	-2(3)	16(3)	-9(3)
C15	32(3)	21(3)	30(3)	-7(2)	14(3)	-5(2)
C16	29(3)	21(3)	25(3)	-5(2)	11(3)	-3(2)
Br2	19(1)	16(1)	26(1)	-2(1)	9(1)	0(1)
O4	62(4)	35(3)	38(3)	3(2)	12(3)	-3(3)

Table S5. Hydrogen coordinates (x 10^4) and isotropic displacement parameters ($Å^2$ x 10^3) for jonap15.

	x	y	z	U(eq)
H1	3740(70)	3750(90)	5230(60)	30(20)
H1A	4630	4896	4423	31
H1B	3469	4521	3707	31
H3A	3404	1854	3140	27
H3B	3335	2706	2172	27
H4	5893	734	3449	22
H5A	4592	4187	2137	27
H5B	5033	5208	3066	27
H6	6962	2534	4109	20
H8	4276	-1300	2987	25
H9	3707	-2828	3920	28
H10	4057	-2340	5590	29
H11	4868	-303	6265	25
H13	7189	5720	4134	30
H14	7440	7370	5371	36
H15	7113	6791	6791	33
H16	6546	4612	6929	30
H4A	7528	9214	6934	71
H4B	6893	9636	7430	71

Table S6. Torsion angles [°] for jonap15.

Cu1-O1-C1-C2	50.3(5)	C2-N1-C6-C12	84.0(5)
Cu1-N1-C2-C1	13.7(6)	C3-O2-C4-N1	-37.7(5)
Cu1-N1-C2-C3	-109.1(4)	C3-O2-C4-C7	81.3(5)
Cu1-N1-C2-C5	134.9(4)	C3-C2-C5-O3	-102.9(5)
Cu1-N1-C4-O2	140.6(4)	C4-O2-C3-C2	45.8(6)
Cu1-N1-C4-C7	21.6(5)	C4-N1-C2-C1	135.2(5)
Cu1-N1-C6-O3	-159.8(3)	C4-N1-C2-C3	12.4(5)
Cu1-N1-C6-C12	-39.9(5)	C4-N1-C2-C5	-103.6(5)
Cu1-N2-C7-C4	1.9(6)	C4-N1-C6-O3	77.8(6)
Cu1-N2-C7-C8	-177.0(4)	C4-N1-C6-C12	-162.3(4)
Cu1-N2-C11-C10	177.9(4)	C4-C7-C8-C9	-179.6(5)
Cu1-N3-C12-C6	-6.0(6)	C5-O3-C6-N1	40.2(5)
Cu1-N3-C12-C13	170.0(5)	C5-O3-C6-C12	-76.4(5)
Cu1-N3-C16-C15	-166.9(5)	C5-C2-C3-O2	74.4(6)
O1-C1-C2-N1	-45.5(7)	C6-O3-C5-C2	-27.7(6)
O1-C1-C2-C3	69.6(7)	C6-N1-C2-C1	-103.9(5)
O1-C1-C2-C5	-161.1(5)	C6-N1-C2-C3	133.3(5)
O2-C4-C7-N2	-132.7(5)	C6-N1-C2-C5	17.3(5)
O2-C4-C7-C8	46.2(7)	C6-N1-C4-O2	-98.3(5)
O3-C6-C12-N3	147.8(5)	C6-N1-C4-C7	142.8(5)
O3-C6-C12-C13	-28.2(8)	C6-C12-C13-C14	173.7(6)
N1-C2-C3-O2	-35.0(6)	C7-N2-C11-C10	-0.3(9)
N1-C2-C5-O3	5.5(6)	C7-C8-C9-C10	-0.9(9)
N1-C4-C7-N2	-16.2(7)	C8-C9-C10-C11	2.0(9)
N1-C4-C7-C8	162.7(5)	C9-C10-C11-N2	-1.4(9)
N1-C6-C12-N3	31.3(6)	C11-N2-C7-C4	-179.8(5)
N1-C6-C12-C13	-144.7(6)	C11-N2-C7-C8	1.4(9)
N2-C7-C8-C9	-0.7(9)	C12-N3-C16-C15	0.9(9)
N3-C12-C13-C14	-2.1(10)	C12-C13-C14-C15	1.7(10)
C1-C2-C3-O2	-156.0(5)	C13-C14-C15-C16	-0.1(10)
C1-C2-C5-O3	126.4(5)	C14-C15-C16-N3	-1.3(10)
C2-N1-C4-O2	14.5(5)	C16-N3-C12-C6	-175.3(5)
C2-N1-C4-C7	-104.5(5)	C16-N3-C12-C13	0.8(9)
C2-N1-C6-O3	-35.8(5)		

Table S7. Hydrogen bonds and close contacts for jonap15 [Å and °].

D-H...A	d(D-H)	d(H...A)	d(D...A)	<(DHA)
O1-H1...Br2	0.98(9)	2.32(9)	3.237(4)	156(7)
O4-H4A...Br2#1	0.85	2.49	3.303(6)	159.4
O4-H4B...Br1#2	0.85	2.59	3.392(6)	157.7

Symmetry transformations used to generate equivalent atoms:

#1 x+1/2,y+1/2,z #2 x,y+1,z

REFERENCE NUMBER: jonap08

2a

CRYSTAL STRUCTURE REPORT

C_{20} H_{20} Cu F_6 N_4 O_9 S_2

or

[(κ^4-L)Cu(NCMe)][OTf]$_2$

Report prepared for:

A. Panda, Prof. W. Jones

September 23, 2022

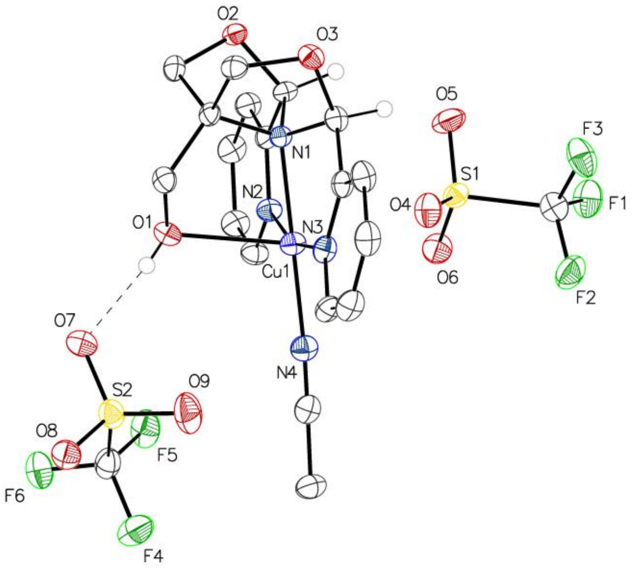

William W. Brennessel

X-ray Crystallographic Facility

Department of Chemistry, University of Rochester

120 Trustee Road

Rochester, NY 14627

Data collection

A crystal (0.34 x 0.034 x 0.016 mm^3) was placed onto a thin glass optical fiber or a nylon loop and mounted on a Rigaku XtaLAB Synergy-S Dualflex diffractometer equipped with a HyPix-6000HE HPC area detector for data collection at 99.99(10) K. A preliminary set of cell constants and an orientation matrix were calculated from a small sampling of reflections.[1] A short pre-experiment was run, from which an optimal data collection strategy was determined. The full data collection was carried out using a PhotonJet (Cu) X-ray source with frame times of 1.07 and 4.29 seconds and a detector distance of 34.0 mm. Series of frames were collected in 0.50º steps in ω at different 2θ, κ, and ϕ settings. After the intensity data were corrected for absorption, the final cell constants were calculated from the xyz centroids of 14895 strong reflections from the actual data collection after integration.[1] See Table S8 for additional crystal and refinement information.

Structure solution and refinement

The structure was solved using SHELXT[2] and refined using SHELXL.[3] The space group *P*-1 was determined based on intensity statistics. Most or all non-hydrogen atoms were assigned from the solution. Full-matrix least squares / difference Fourier cycles were performed which located any remaining non-hydrogen atoms. All non-hydrogen atoms were refined with anisotropic displacement parameters. The O-H hydrogen atom was found from the difference Fourier map and refined freely. All other hydrogen atoms were placed in ideal positions and refined as riding atoms with relative isotropic displacement parameters. The final full matrix least squares refinement converged to $R1 = 0.0430$ (F^2, $I > 2\sigma(I)$) and $wR2 = 0.1151$ (F^2, all data).

Structure description

The structure is the one suggested. The asymmetric unit contains one dicationic copper complex and two triflate anions in general positions. Because the distance between the metal center and atom O4 of one triflate anion is only 2.578(2) Å, the formula unit could be written as [(κ^4-L)Cu(OTf)(MeCN)][OTf] (see figure). The copper complex and the triflate anion containing atom S2 are linked via O-H…O hydrogen bonding (see figure and Table S14).

Structure manipulation and figure generation were performed using Olex2.[4] Unless noted otherwise all structural diagrams containing anisotropic displacement ellipsoids are drawn at the 50 % probability level.

Data collection, structure solution, and structure refinement were conducted at the X-ray Crystallographic Facility, B04 Hutchison Hall, Department of Chemistry, University of Rochester. The instrument was purchased with funding from NSF MRI program grant CHE-1725028. All publications arising from this report MUST either 1) include William W. Brennessel as a coauthor or 2) acknowledge William W. Brennessel and the X-ray Crystallographic Facility of the Department of Chemistry at the University of Rochester.

1 *CrysAlisPro*, version 171.42.64a; Rigaku Corporation: Oxford, UK, 2022.

2 Sheldrick, G. M. *SHELXT*, version 2018/2; *Acta. Crystallogr.* **2015**, *A71*, 3-8.

3 Sheldrick, G. M. *SHELXL*, version 2019/2; *Acta. Crystallogr.* **2015**, *C71*, 3-8.

4 Dolomanov, O. V.; Bourhis, L. J.; Gildea, R. J.; Howard, J. A. K.; Puschmann, H. *Olex2*, version 1.5; *J. Appl. Cryst.* **2009**, *42*, 339-341.

Some equations of interest:

$$R_{int} = \Sigma\, |F_o^2 - \langle F_o^2 \rangle| \,/\, \Sigma |F_o^2|$$

$$R1 = \Sigma ||F_o| - |F_c|| \,/\, \Sigma |F_o|$$

$$wR2 = [\Sigma [w(F_o^2 - F_c^2)^2] \,/\, \Sigma\, [w(F_o^2)^2]]^{1/2}$$

$$\text{where } w = 1 \,/\, [\sigma^2 (F_o^2) + (aP)^2 + bP] \text{ and}$$

$$P = 1/3 \max (0, F_o^2) + 2/3\, F_c^2$$

$$\text{GOF} = S = [\Sigma [w(F_o^2 - F_c^2)^2] \,/\, (m-n)]^{1/2}$$

where m = number of reflections and n = number of parameters

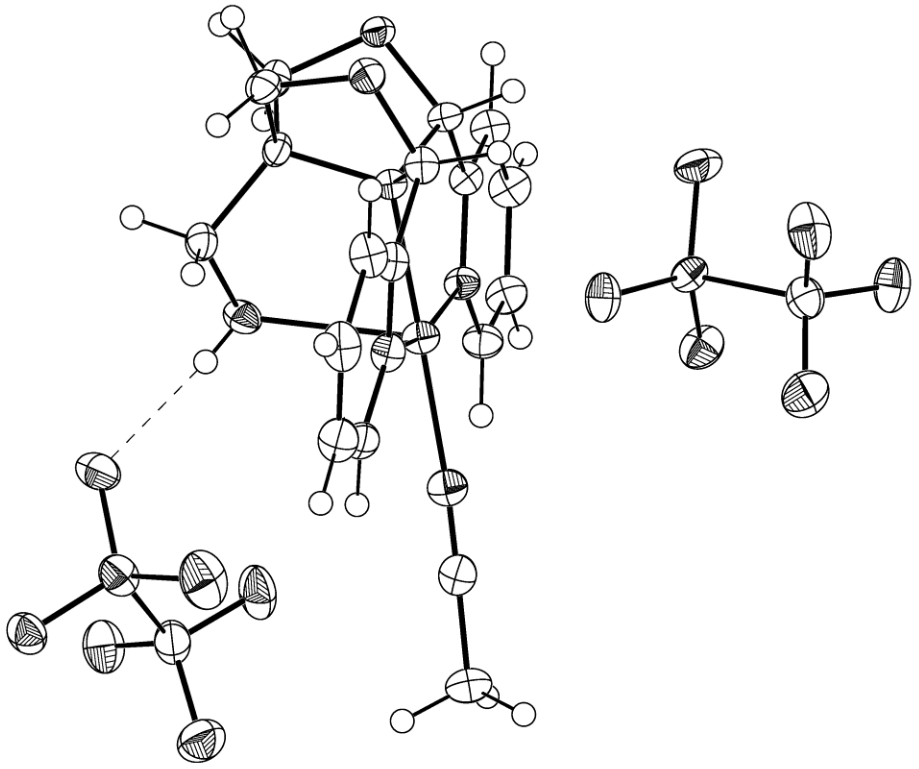

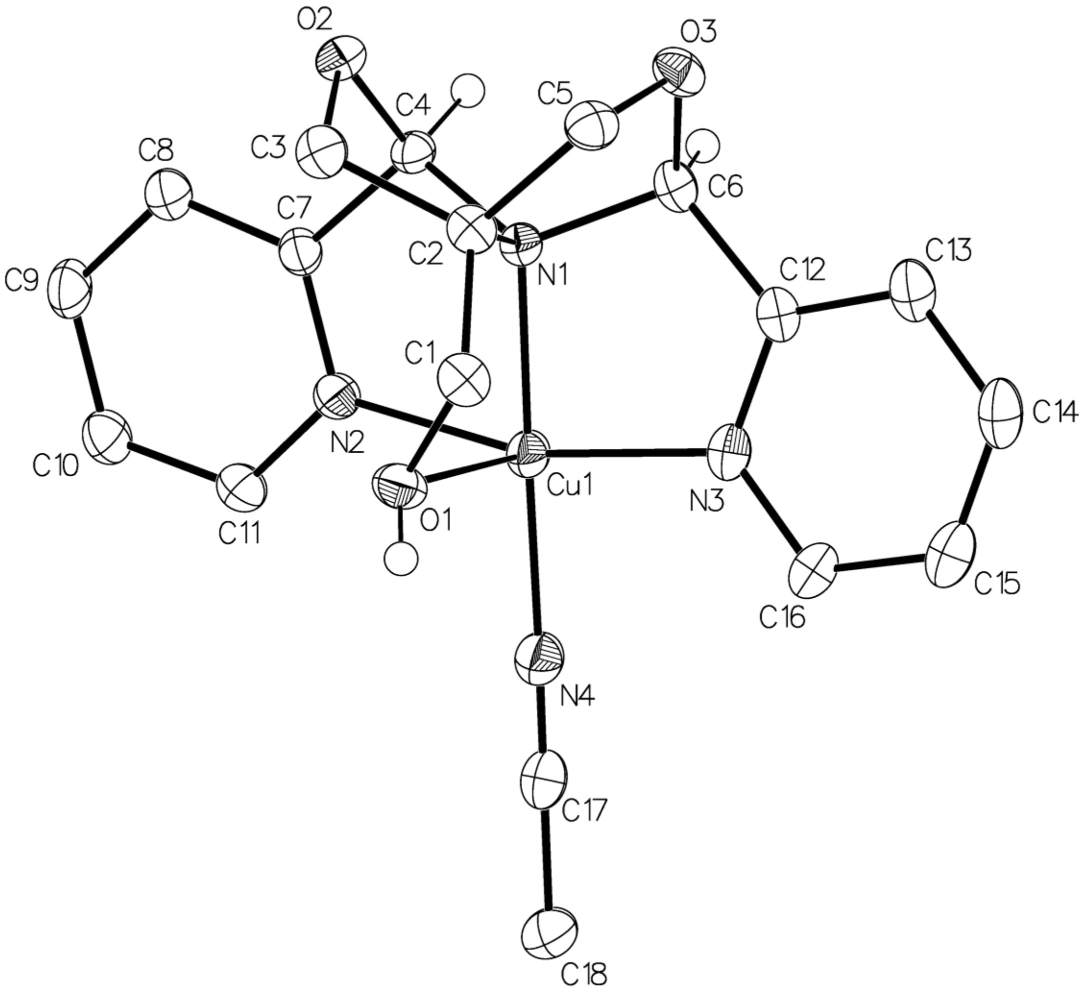
O2
O3
C4
C5
C3
C8
C7
C6
C2
N1
C9
C12
C13
C1
C14
N2
C10
Cu1
N3
O1
C11
C15
C16
N4
C17
C18

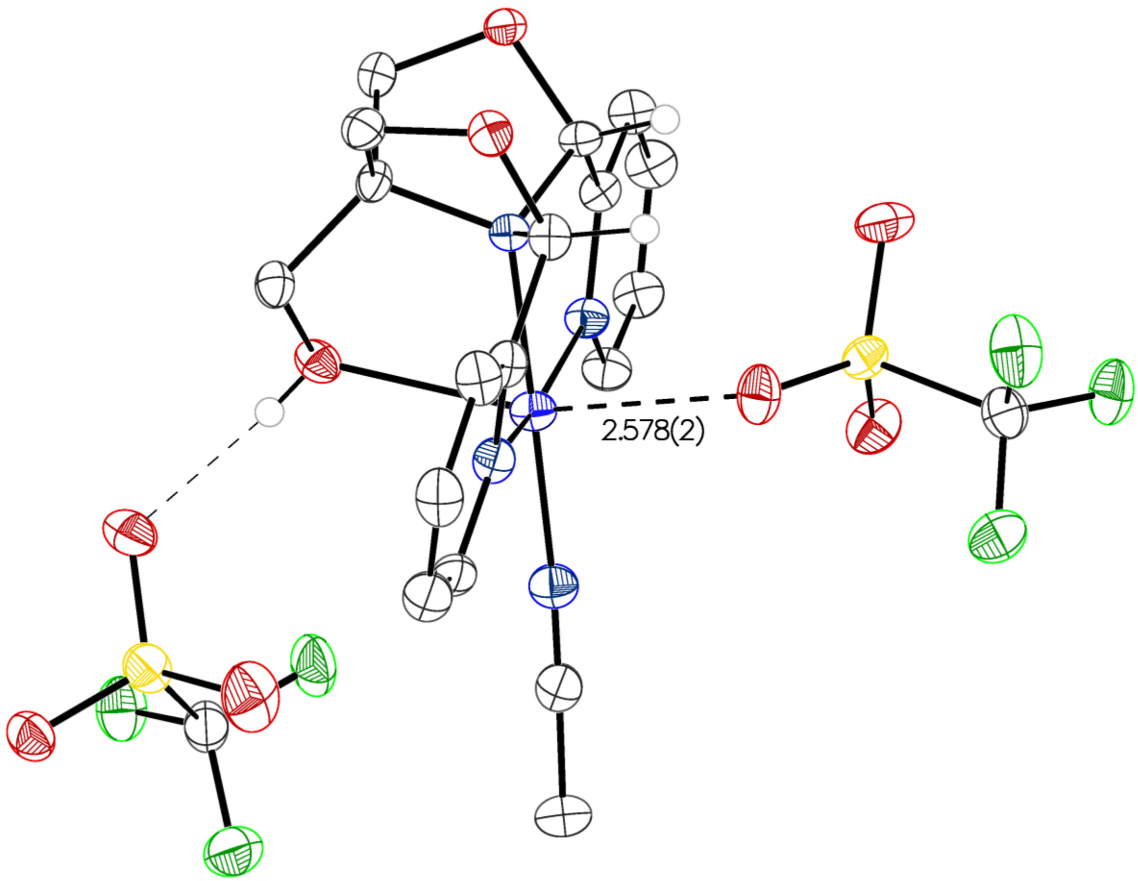
2.578(2)

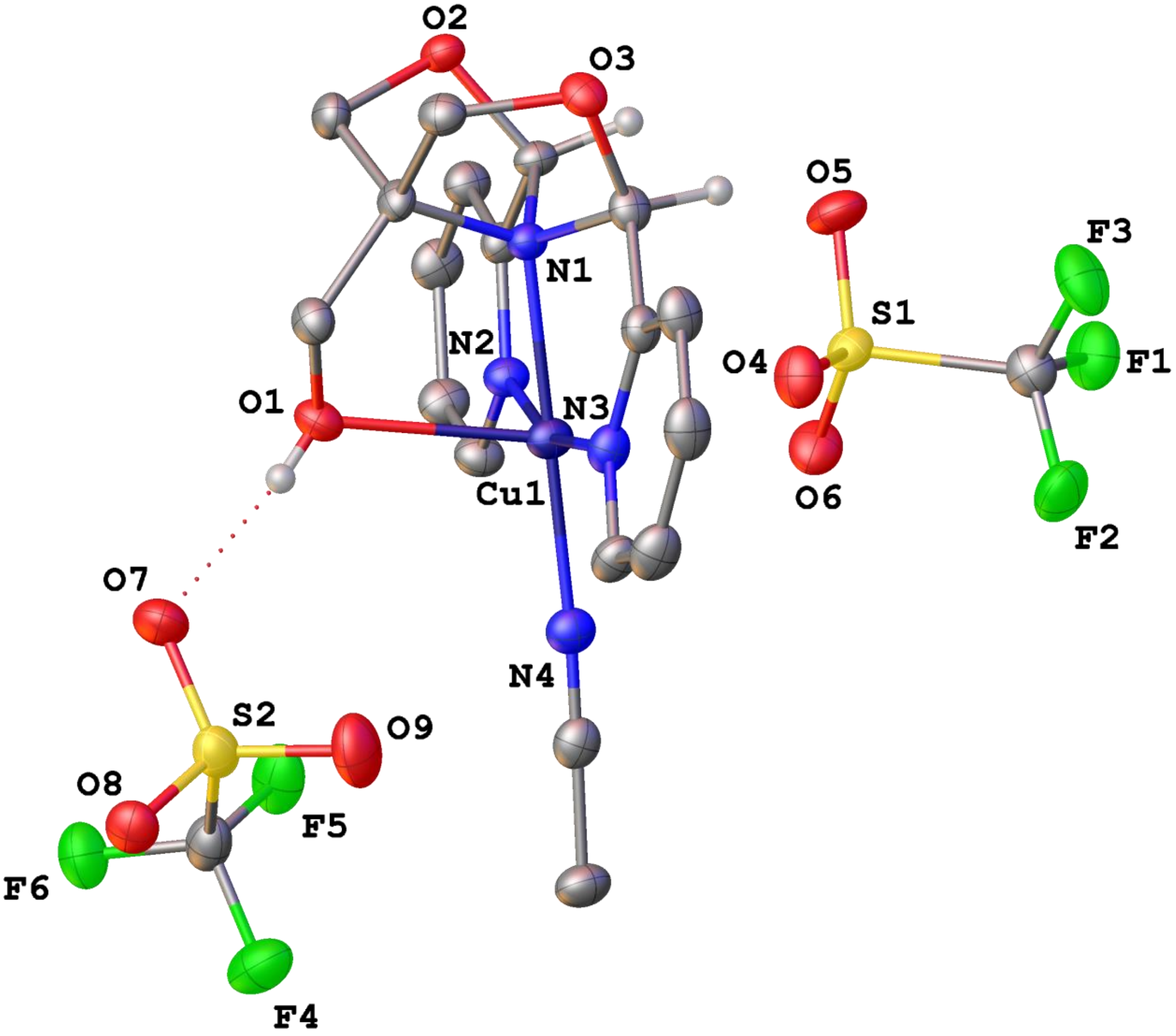
O2
O3
O5
F3
N1
S1
N2
O4
F1
O1
N3
Cu1
O6
F2
O7
N4
S2
O9
O8
F5
F6
F4

Table S8. Crystal data and structure refinement for jonap08.

Identification code	jonap08	
Empirical formula	C20 H20 Cu F6 N4 O9 S2	
Formula weight	702.06	
Temperature	99.99(10) K	
Wavelength	1.54184 Å	
Crystal system	triclinic	
Space group	*P*-1	
Unit cell dimensions	*a* = 8.7846(2) Å	α = 101.605(3)°
	b = 12.8459(4) Å	β = 107.706(3)°
	c = 13.2400(4) Å	γ = 104.284(3)°
Volume	1316.13(7) $Å^3$	
Z	2	
Density (calculated)	1.772 Mg/m^3	
Absorption coefficient	3.592 mm^{-1}	
F(000)	710	
Crystal color, morphology	blue, needle	
Crystal size	0.34 x 0.034 x 0.016 mm^3	
Theta range for data collection	3.673 to 80.153°	
Index ranges	$-10 \le h \le 11$, $-16 \le k \le 15$, $-16 \le l \le 16$	
Reflections collected	27882	
Independent reflections	5567 [*R*(int) = 0.0505]	
Observed reflections	4783	
Completeness to theta = 74.504°	99.3%	
Absorption correction	Multi-scan	
Max. and min. transmission	1.00000 and 0.60750	
Refinement method	Full-matrix least-squares on F^2	
Data / restraints / parameters	5567 / 0 / 384	
Goodness-of-fit on F^2	1.113	
Final *R* indices [*I*>2sigma(*I*)]	*R*1 = 0.0430, *wR*2 = 0.1109	
R indices (all data)	*R*1 = 0.0513, *wR*2 = 0.1151	
Largest diff. peak and hole	0.448 and -0.888 e.$Å^{-3}$	

Table S9. Atomic coordinates (x 10^4) and equivalent isotropic displacement parameters ($Å^2$x 10^3) for jonap08. U_{eq} is defined as one third of the trace of the orthogonalized U_{ij} tensor.

	x	y	z	U_{eq}
Cu1	5102(1)	6895(1)	2633(1)	19(1)
O1	7470(3)	8452(2)	3921(2)	22(1)
O2	7113(2)	5914(2)	5302(2)	19(1)
O3	3982(2)	6660(2)	5366(2)	21(1)
N1	5344(3)	6610(2)	4122(2)	16(1)
N2	6640(3)	6003(2)	2592(2)	19(1)
N3	3409(3)	7560(2)	2949(2)	21(1)
N4	4846(3)	7205(2)	1198(2)	23(1)
C1	7116(3)	8673(2)	4907(2)	21(1)
C2	6540(3)	7565(2)	5159(2)	18(1)
C3	7960(3)	7105(2)	5606(2)	21(1)
C4	6003(3)	5650(2)	4188(2)	17(1)
C5	5481(3)	7633(2)	5907(2)	22(1)
C6	3671(3)	6435(2)	4227(2)	20(1)
C7	6945(3)	5514(2)	3406(2)	18(1)
C8	8033(3)	4898(2)	3523(2)	22(1)
C9	8851(4)	4784(2)	2782(2)	25(1)
C10	8534(4)	5277(2)	1938(2)	24(1)
C11	7419(3)	5881(2)	1861(2)	22(1)
C12	2906(3)	7245(2)	3737(2)	20(1)
C13	1783(3)	7646(2)	4100(3)	26(1)
C14	1136(4)	8387(3)	3615(3)	28(1)
C15	1644(4)	8705(3)	2801(3)	28(1)
C16	2800(3)	8288(2)	2492(2)	23(1)
C17	4750(3)	7438(2)	398(2)	24(1)
C18	4640(4)	7753(3)	-602(2)	28(1)
S2	7581(1)	10792(1)	2702(1)	27(1)
F4	7595(3)	10505(2)	678(2)	40(1)
F5	8175(2)	9229(2)	1418(2)	38(1)
F6	10045(2)	10873(2)	1946(2)	35(1)
O7	8569(3)	10482(2)	3623(2)	34(1)

O8	7980(3)	11978(2)	2884(2)	29(1)
O9	5819(3)	10126(2)	2206(2)	44(1)
C20	8387(4)	10332(2)	1635(3)	29(1)
S1	2636(1)	4030(1)	1357(1)	22(1)
F1	377(2)	2146(2)	-53(2)	36(1)
F2	-294(2)	3626(2)	-215(2)	41(1)
F3	-469(2)	2982(2)	1139(2)	39(1)
O4	2488(3)	5099(2)	1849(2)	30(1)
O5	3175(3)	3440(2)	2136(2)	34(1)
O6	3435(3)	4053(2)	561(2)	30(1)
C19	446(4)	3154(2)	507(3)	26(1)

Table S10. Bond lengths [Å] and angles [°] for jonap08.

Cu(1)-O(1)	2.377(2)	C(8)-C(9)	1.386(4)
Cu(1)-N(1)	2.036(2)	C(9)-H(9)	0.9500
Cu(1)-N(2)	1.982(2)	C(9)-C(10)	1.382(4)
Cu(1)-N(3)	1.994(2)	C(10)-H(10)	0.9500
Cu(1)-N(4)	1.977(2)	C(10)-C(11)	1.385(4)
O(1)-H(1)	0.75(4)	C(11)-H(11)	0.9500
O(1)-C(1)	1.422(3)	C(12)-C(13)	1.383(4)
O(2)-C(3)	1.436(3)	C(13)-H(13)	0.9500
O(2)-C(4)	1.415(3)	C(13)-C(14)	1.390(4)
O(3)-C(5)	1.437(3)	C(14)-H(14)	0.9500
O(3)-C(6)	1.402(3)	C(14)-C(15)	1.381(5)
N(1)-C(2)	1.517(3)	C(15)-H(15)	0.9500
N(1)-C(4)	1.493(3)	C(15)-C(16)	1.385(4)
N(1)-C(6)	1.484(3)	C(16)-H(16)	0.9500
N(2)-C(7)	1.344(3)	C(17)-C(18)	1.443(4)
N(2)-C(11)	1.350(3)	C(18)-H(18A)	0.9800
N(3)-C(12)	1.346(4)	C(18)-H(18B)	0.9800
N(3)-C(16)	1.344(4)	C(18)-H(18C)	0.9800
N(4)-C(17)	1.141(4)	S(2)-O(7)	1.456(2)
C(1)-H(1A)	0.9900	S(2)-O(8)	1.428(2)
C(1)-H(1B)	0.9900	S(2)-O(9)	1.436(2)
C(1)-C(2)	1.526(4)	S(2)-C(20)	1.824(3)
C(2)-C(3)	1.521(4)	F(4)-C(20)	1.340(4)
C(2)-C(5)	1.556(4)	F(5)-C(20)	1.340(3)
C(3)-H(3A)	0.9900	F(6)-C(20)	1.339(4)
C(3)-H(3B)	0.9900	S(1)-O(4)	1.452(2)
C(4)-H(4)	1.0000	S(1)-O(5)	1.433(2)
C(4)-C(7)	1.520(3)	S(1)-O(6)	1.435(2)
C(5)-H(5A)	0.9900	S(1)-C(19)	1.828(3)
C(5)-H(5B)	0.9900	F(1)-C(19)	1.333(3)
C(6)-H(6)	1.0000	F(2)-C(19)	1.324(4)
C(6)-C(12)	1.518(4)	F(3)-C(19)	1.337(3)
C(7)-C(8)	1.377(4)	N(1)-Cu(1)-O(1)	76.46(8)
C(8)-H(8)	0.9500	N(2)-Cu(1)-O(1)	88.83(8)

N(2)-Cu(1)-N(1)	83.95(9)	C(3)-C(2)-C(1)	114.1(2)
N(2)-Cu(1)-N(3)	164.44(10)	C(3)-C(2)-C(5)	112.0(2)
N(3)-Cu(1)-O(1)	94.00(8)	O(2)-C(3)-C(2)	103.8(2)
N(3)-Cu(1)-N(1)	81.86(9)	O(2)-C(3)-H(3A)	111.0
N(4)-Cu(1)-O(1)	103.04(9)	O(2)-C(3)-H(3B)	111.0
N(4)-Cu(1)-N(1)	178.48(9)	C(2)-C(3)-H(3A)	111.0
N(4)-Cu(1)-N(2)	97.49(9)	C(2)-C(3)-H(3B)	111.0
N(4)-Cu(1)-N(3)	96.77(10)	H(3A)-C(3)-H(3B)	109.0
Cu(1)-O(1)-H(1)	122(3)	O(2)-C(4)-N(1)	106.4(2)
C(1)-O(1)-Cu(1)	104.91(15)	O(2)-C(4)-H(4)	109.9
C(1)-O(1)-H(1)	106(3)	O(2)-C(4)-C(7)	110.3(2)
C(4)-O(2)-C(3)	103.49(19)	N(1)-C(4)-H(4)	109.9
C(6)-O(3)-C(5)	105.52(19)	N(1)-C(4)-C(7)	110.3(2)
C(2)-N(1)-Cu(1)	117.09(16)	C(7)-C(4)-H(4)	109.9
C(4)-N(1)-Cu(1)	109.34(15)	O(3)-C(5)-C(2)	105.6(2)
C(4)-N(1)-C(2)	105.13(19)	O(3)-C(5)-H(5A)	110.6
C(6)-N(1)-Cu(1)	107.24(16)	O(3)-C(5)-H(5B)	110.6
C(6)-N(1)-C(2)	103.61(19)	C(2)-C(5)-H(5A)	110.6
C(6)-N(1)-C(4)	114.6(2)	C(2)-C(5)-H(5B)	110.6
C(7)-N(2)-Cu(1)	114.65(17)	H(5A)-C(5)-H(5B)	108.7
C(7)-N(2)-C(11)	119.0(2)	O(3)-C(6)-N(1)	106.3(2)
C(11)-N(2)-Cu(1)	126.32(19)	O(3)-C(6)-H(6)	110.2
C(12)-N(3)-Cu(1)	113.63(18)	O(3)-C(6)-C(12)	112.1(2)
C(16)-N(3)-Cu(1)	127.12(19)	N(1)-C(6)-H(6)	110.2
C(16)-N(3)-C(12)	119.2(2)	N(1)-C(6)-C(12)	107.7(2)
C(17)-N(4)-Cu(1)	176.6(2)	C(12)-C(6)-H(6)	110.2
O(1)-C(1)-H(1A)	109.9	N(2)-C(7)-C(4)	116.9(2)
O(1)-C(1)-H(1B)	109.9	N(2)-C(7)-C(8)	122.1(2)
O(1)-C(1)-C(2)	108.8(2)	C(8)-C(7)-C(4)	120.9(2)
H(1A)-C(1)-H(1B)	108.3	C(7)-C(8)-H(8)	120.5
C(2)-C(1)-H(1A)	109.9	C(7)-C(8)-C(9)	119.0(3)
C(2)-C(1)-H(1B)	109.9	C(9)-C(8)-H(8)	120.5
N(1)-C(2)-C(1)	112.4(2)	C(8)-C(9)-H(9)	120.4
N(1)-C(2)-C(3)	102.0(2)	C(10)-C(9)-C(8)	119.1(3)
N(1)-C(2)-C(5)	103.5(2)	C(10)-C(9)-H(9)	120.4
C(1)-C(2)-C(5)	111.9(2)	C(9)-C(10)-H(10)	120.4

C(9)-C(10)-C(11)	119.1(2)
C(11)-C(10)-H(10)	120.4
N(2)-C(11)-C(10)	121.6(3)
N(2)-C(11)-H(11)	119.2
C(10)-C(11)-H(11)	119.2
N(3)-C(12)-C(6)	116.1(2)
N(3)-C(12)-C(13)	122.4(3)
C(13)-C(12)-C(6)	121.5(2)
C(12)-C(13)-H(13)	120.8
C(12)-C(13)-C(14)	118.3(3)
C(14)-C(13)-H(13)	120.8
C(13)-C(14)-H(14)	120.3
C(15)-C(14)-C(13)	119.3(3)
C(15)-C(14)-H(14)	120.3
C(14)-C(15)-H(15)	120.3
C(14)-C(15)-C(16)	119.4(3)
C(16)-C(15)-H(15)	120.3
N(3)-C(16)-C(15)	121.4(3)
N(3)-C(16)-H(16)	119.3
C(15)-C(16)-H(16)	119.3
N(4)-C(17)-C(18)	179.0(3)
C(17)-C(18)-H(18A)	109.5
C(17)-C(18)-H(18B)	109.5
C(17)-C(18)-H(18C)	109.5
H(18A)-C(18)-H(18B)	109.5
H(18A)-C(18)-H(18C)	109.5
H(18B)-C(18)-H(18C)	109.5
O(7)-S(2)-C(20)	101.57(14)
O(8)-S(2)-O(7)	114.08(14)
O(8)-S(2)-O(9)	116.73(15)
O(8)-S(2)-C(20)	104.43(14)
O(9)-S(2)-O(7)	114.52(15)
O(9)-S(2)-C(20)	102.88(15)
F(4)-C(20)-S(2)	111.9(2)
F(4)-C(20)-F(5)	107.3(2)
F(5)-C(20)-S(2)	111.0(2)
F(6)-C(20)-S(2)	111.9(2)
F(6)-C(20)-F(4)	107.3(3)
F(6)-C(20)-F(5)	107.0(2)
O(4)-S(1)-C(19)	103.48(13)
O(5)-S(1)-O(4)	114.24(14)
O(5)-S(1)-O(6)	115.46(14)
O(5)-S(1)-C(19)	103.67(14)
O(6)-S(1)-O(4)	114.62(14)
O(6)-S(1)-C(19)	103.16(13)
F(1)-C(19)-S(1)	110.7(2)
F(1)-C(19)-F(3)	107.2(2)
F(2)-C(19)-S(1)	111.6(2)
F(2)-C(19)-F(1)	108.4(3)
F(2)-C(19)-F(3)	107.7(2)
F(3)-C(19)-S(1)	111.1(2)

Table S11. Anisotropic displacement parameters ($Å^2$x 10^3) for jonap08. The anisotropic displacement factor exponent takes the form: $-2\pi^2[h^2 a^{*2}U_{11} + ... + 2 h k a^* b^* U_{12}]$

	U_{11}	U_{22}	U_{33}	U_{23}	U_{13}	U_{12}
Cu1	20(1)	23(1)	20(1)	8(1)	9(1)	11(1)
O1	26(1)	21(1)	25(1)	10(1)	13(1)	7(1)
O2	22(1)	20(1)	17(1)	7(1)	6(1)	8(1)
O3	24(1)	21(1)	21(1)	6(1)	13(1)	7(1)
N1	16(1)	18(1)	16(1)	6(1)	6(1)	6(1)
N2	21(1)	21(1)	18(1)	7(1)	10(1)	10(1)
N3	18(1)	21(1)	22(1)	4(1)	6(1)	9(1)
N4	23(1)	26(1)	24(1)	9(1)	10(1)	11(1)
C1	22(1)	19(1)	21(1)	2(1)	9(1)	5(1)
C2	19(1)	18(1)	14(1)	1(1)	5(1)	4(1)
C3	19(1)	18(1)	20(1)	3(1)	4(1)	3(1)
C4	19(1)	21(1)	15(1)	7(1)	7(1)	10(1)
C5	25(1)	22(1)	18(1)	5(1)	9(1)	8(1)
C6	18(1)	21(1)	22(1)	5(1)	10(1)	7(1)
C7	16(1)	20(1)	18(1)	6(1)	7(1)	6(1)
C8	21(1)	27(1)	23(1)	9(1)	10(1)	10(1)
C9	22(1)	29(2)	28(2)	8(1)	12(1)	14(1)
C10	24(1)	29(1)	24(1)	8(1)	13(1)	12(1)
C11	24(1)	26(1)	22(1)	10(1)	12(1)	10(1)
C12	16(1)	20(1)	22(1)	4(1)	7(1)	5(1)
C13	20(1)	24(1)	34(2)	6(1)	12(1)	9(1)
C14	21(1)	26(1)	38(2)	4(1)	11(1)	11(1)
C15	25(1)	27(2)	32(2)	6(1)	6(1)	14(1)
C16	22(1)	23(1)	22(1)	7(1)	4(1)	11(1)
C17	21(1)	24(1)	26(2)	6(1)	8(1)	11(1)
C18	29(2)	36(2)	24(2)	15(1)	10(1)	15(1)
S2	22(1)	26(1)	34(1)	11(1)	9(1)	7(1)
F4	48(1)	40(1)	30(1)	11(1)	6(1)	19(1)
F5	34(1)	21(1)	48(1)	2(1)	5(1)	11(1)
F6	27(1)	32(1)	43(1)	5(1)	14(1)	6(1)
O7	37(1)	30(1)	35(1)	18(1)	11(1)	9(1)

O8	31(1)	22(1)	34(1)	9(1)	10(1)	11(1)
O9	23(1)	41(1)	62(2)	13(1)	13(1)	5(1)
C20	26(1)	22(1)	32(2)	4(1)	4(1)	7(1)
S1	22(1)	25(1)	17(1)	5(1)	7(1)	9(1)
F1	37(1)	28(1)	35(1)	-4(1)	11(1)	8(1)
F2	30(1)	43(1)	39(1)	13(1)	-1(1)	12(1)
F3	34(1)	33(1)	49(1)	3(1)	27(1)	4(1)
O4	29(1)	23(1)	32(1)	-1(1)	11(1)	7(1)
O5	38(1)	39(1)	24(1)	15(1)	5(1)	16(1)
O6	29(1)	39(1)	25(1)	7(1)	15(1)	10(1)
C19	28(1)	23(1)	29(2)	5(1)	13(1)	10(1)

Table S12. Hydrogen coordinates (x 10^4) and isotropic displacement parameters ($Å^2$ x 10^3) for jonap08.

	x	y	z	U(eq)
H1	7710(50)	9010(40)	3810(30)	34(11)
H1A	6216	9023	4806	26
H1B	8144	9201	5534	26
H3A	8810	7269	5261	25
H3B	8529	7430	6423	25
H4	5044	4941	3992	21
H5A	6125	7623	6660	26
H5B	5184	8332	5973	26
H6	2916	5641	3819	24
H8	8220	4556	4102	27
H9	9620	4372	2854	30
H10	9073	5203	1418	29
H11	7197	6219	1280	27
H13	1461	7422	4667	31
H14	352	8672	3841	34
H15	1205	9205	2456	34
H16	3170	8522	1945	28
H18A	3471	7419	-1140	42
H18B	5390	7479	-921	42
H18C	4982	8575	-424	42

Table S13. Torsion angles [°] for jonap08.

Cu1-O1-C1-C2	47.5(2)	C2-N1-C6-C12	86.3(2)
Cu1-N1-C2-C1	11.0(3)	C3-O2-C4-N1	-38.2(2)
Cu1-N1-C2-C3	-111.58(19)	C3-O2-C4-C7	81.5(2)
Cu1-N1-C2-C5	131.95(17)	C3-C2-C5-O3	-100.0(2)
Cu1-N1-C4-O2	143.13(15)	C4-O2-C3-C2	44.6(2)
Cu1-N1-C4-C7	23.4(2)	C4-N1-C2-C1	132.6(2)
Cu1-N1-C6-O3	-158.44(16)	C4-N1-C2-C3	10.0(2)
Cu1-N1-C6-C12	-38.2(2)	C4-N1-C2-C5	-106.5(2)
Cu1-N2-C7-C4	3.6(3)	C4-N1-C6-O3	80.0(2)
Cu1-N2-C7-C8	-177.6(2)	C4-N1-C6-C12	-159.7(2)
Cu1-N2-C11-C10	177.0(2)	C4-C7-C8-C9	179.2(2)
Cu1-N3-C12-C6	-1.1(3)	C5-O3-C6-N1	41.0(2)
Cu1-N3-C12-C13	177.7(2)	C5-O3-C6-C12	-76.4(2)
Cu1-N3-C16-C15	-178.8(2)	C5-C2-C3-O2	77.1(2)
O1-C1-C2-N1	-41.6(3)	C6-O3-C5-C2	-30.6(3)
O1-C1-C2-C3	73.9(3)	C6-N1-C2-C1	-106.8(2)
O1-C1-C2-C5	-157.6(2)	C6-N1-C2-C3	130.6(2)
O2-C4-C7-N2	-135.8(2)	C6-N1-C2-C5	14.1(2)
O2-C4-C7-C8	45.4(3)	C6-N1-C4-O2	-96.4(2)
O3-C6-C12-N3	143.4(2)	C6-N1-C4-C7	143.8(2)
O3-C6-C12-C13	-35.4(3)	C6-C12-C13-C14	179.8(3)
N1-C2-C3-O2	-33.0(2)	C7-N2-C11-C10	-0.7(4)
N1-C2-C5-O3	9.1(3)	C7-C8-C9-C10	-1.0(4)
N1-C4-C7-N2	-18.5(3)	C8-C9-C10-C11	0.6(4)
N1-C4-C7-C8	162.8(2)	C9-C10-C11-N2	0.2(4)
N1-C6-C12-N3	26.8(3)	C11-N2-C7-C4	-178.4(2)
N1-C6-C12-C13	-152.0(2)	C11-N2-C7-C8	0.3(4)
N2-C7-C8-C9	0.5(4)	C12-N3-C16-C15	-1.1(4)
N3-C12-C13-C14	1.1(4)	C12-C13-C14-C15	-0.7(4)
C1-C2-C3-O2	-154.4(2)	C13-C14-C15-C16	-0.6(4)
C1-C2-C5-O3	130.4(2)	C14-C15-C16-N3	1.6(4)
C2-N1-C4-O2	16.6(2)	C16-N3-C12-C6	-179.0(2)
C2-N1-C4-C7	-103.1(2)	C16-N3-C12-C13	-0.2(4)
C2-N1-C6-O3	-34.0(2)	O7-S2-C20-F4	177.9(2)

O7-S2-C20-F5	58.0(2)	O4-S1-C19-F2	55.9(2)
O7-S2-C20-F6	-61.6(2)	O4-S1-C19-F3	-64.3(2)
O8-S2-C20-F4	-63.3(2)	O5-S1-C19-F1	-63.8(2)
O8-S2-C20-F5	176.8(2)	O5-S1-C19-F2	175.4(2)
O8-S2-C20-F6	57.3(2)	O5-S1-C19-F3	55.2(2)
O9-S2-C20-F4	59.1(2)	O6-S1-C19-F1	57.0(2)
O9-S2-C20-F5	-60.8(2)	O6-S1-C19-F2	-63.8(2)
O9-S2-C20-F6	179.6(2)	O6-S1-C19-F3	175.9(2)
O4-S1-C19-F1	176.7(2)		

Table S14. Hydrogen bonds and close contacts for jonap08 [Å and °].

D-H...A	d(D-H)	d(H...A)	d(D...A)	<(DHA)
O1-H1...O7	0.75(4)	1.95(4)	2.701(3)	174(4)

REFERENCE NUMBER: jonap11

3a

CRYSTAL STRUCTURE REPORT

$C_{18.5}$ H_{20} Cl Cu F_6 N_3 O_{10} S_2

or

[(κ^4-L)Cu(OTf)(OH_2)][OTf] · ½ CH_2Cl_2

Report prepared for:

A. Panda, Prof. W. Jones

December 07, 2022

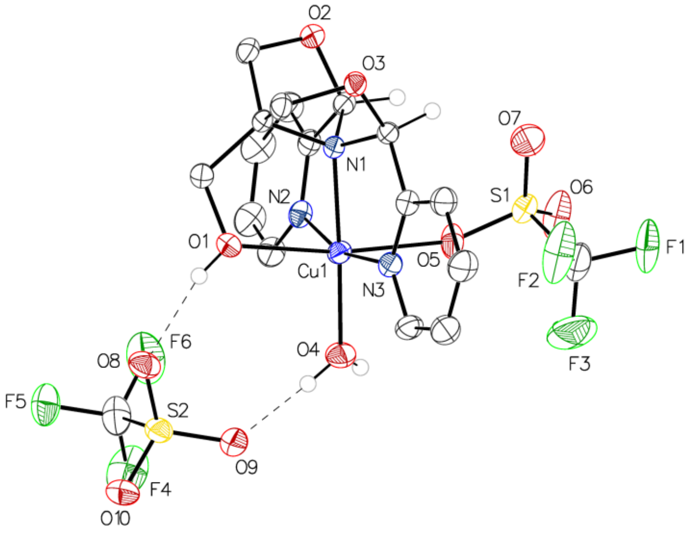

William W. Brennessel

X-ray Crystallographic Facility

Department of Chemistry, University of Rochester

120 Trustee Road

Rochester, NY 14627

Data collection

A crystal (0.108 x 0.084 x 0.026 mm^3) was placed onto a thin glass optical fiber or a nylon loop and mounted on a Rigaku XtaLAB Synergy-S Dualflex diffractometer equipped with a HyPix-6000HE HPC area detector for data collection at 99.95(13) K. A preliminary set of cell constants and an orientation matrix were calculated from a small sampling of reflections.[1] A short pre-experiment was run, from which an optimal data collection strategy was determined. The full data collection was carried out using a PhotonJet (Cu) X-ray source with frame times of 1.16 and 4.65 seconds and a detector distance of 34.0 mm. Series of frames were collected in 0.50º steps in ω at different 2θ, κ, and ϕ settings. After the intensity data were corrected for absorption, the final cell constants were calculated from the xyz centroids of 13204 strong reflections from the actual data collection after integration.[1] See Table S15 for additional crystal and refinement information.

Structure solution and refinement

The structure was solved using SHELXT[2] and refined using SHELXL.[3] The space group *C*2/*c* was determined based on systematic absences and intensity statistics. Most or all non-hydrogen atoms were assigned from the solution. Full-matrix least squares / difference Fourier cycles were performed which located any remaining non-hydrogen atoms. All non-hydrogen atoms were refined with anisotropic displacement parameters. The O-H hydrogen atoms were found from the difference Fourier map and refined freely. All other hydrogen atoms were placed in ideal positions and refined as riding atoms with relative isotropic displacement parameters. The final full matrix least squares refinement converged to $R1 = 0.0481$ (F^2, $I > 2\sigma(I)$) and $wR2 = 0.1264$ (F^2, all data).

Structure description

The structure is the one suggested. The asymmetric unit contains one monocationic copper complex and one triflate anion in general positions and one-half of a dichloromethane solvent molecule of crystallization on a crystallographic two-fold axis. The trilato ligand is modeled as disordered over two positions (0.81:0.19). The triflate anion is modeled as disordered over two positions (0.82:0.18). The dichloromethane solvent molecule is modeled as disordered over the two-fold axis (0.50:0.50) and additionally over two general positions (0.61(4):0.39(4)). Cations and anions are linked along [010] via O-H…O hydrogen bonding (see figure and Table S21).

Structure manipulation and figure generation were performed using Olex2.[4] Unless noted otherwise all structural diagrams containing anisotropic displacement ellipsoids are drawn at the 50 % probability level.

Data collection, structure solution, and structure refinement were conducted at the X-ray Crystallographic Facility, B04 Hutchison Hall, Department of Chemistry, University of Rochester. The instrument was purchased with funding from NSF MRI program grant CHE-1725028. All publications arising from this report MUST either 1) include William W. Brennessel as a coauthor or 2) acknowledge William W. Brennessel and the X-ray

Crystallographic Facility of the Department of Chemistry at the University of Rochester.

---

[1] *CrysAlisPro*, version 171.42.72a; Rigaku Corporation: Oxford, UK, 2022.

[2] Sheldrick, G. M. *SHELXT*, version 2018/2; *Acta. Crystallogr.* **2015**, *A71*, 3-8.

[3] Sheldrick, G. M. *SHELXL*, version 2019/2; *Acta. Crystallogr.* **2015**, *C71*, 3-8.

[4] Dolomanov, O. V.; Bourhis, L. J.; Gildea, R. J.; Howard, J. A. K.; Puschmann, H. *Olex2*, version 1.5; *J. Appl. Cryst.* **2009**, *42*, 339-341.

Some equations of interest:

$$R_{int} = \Sigma\, |F_o^2 - \langle F_o^2 \rangle| \,/\, \Sigma|F_o^2|$$

$$R1 = \Sigma||F_o|-|F_c|| \,/\, \Sigma|F_o|$$

$$wR2 = [\Sigma[w(F_o^2-F_c^2)^2] \,/\, \Sigma\,[w(F_o^2)^2]]^{1/2}$$

where $w = 1 / [\sigma^2 (F_o^2) + (aP)^2 + bP]$ and

$$P = 1/3 \max (0, F_o^2) + 2/3\, F_c^2$$

$$\text{GOF} = S = [\Sigma[w(F_o^2-F_c^2)^2] \,/\, (m-n)]^{1/2}$$

where m = number of reflections and n = number of parameters

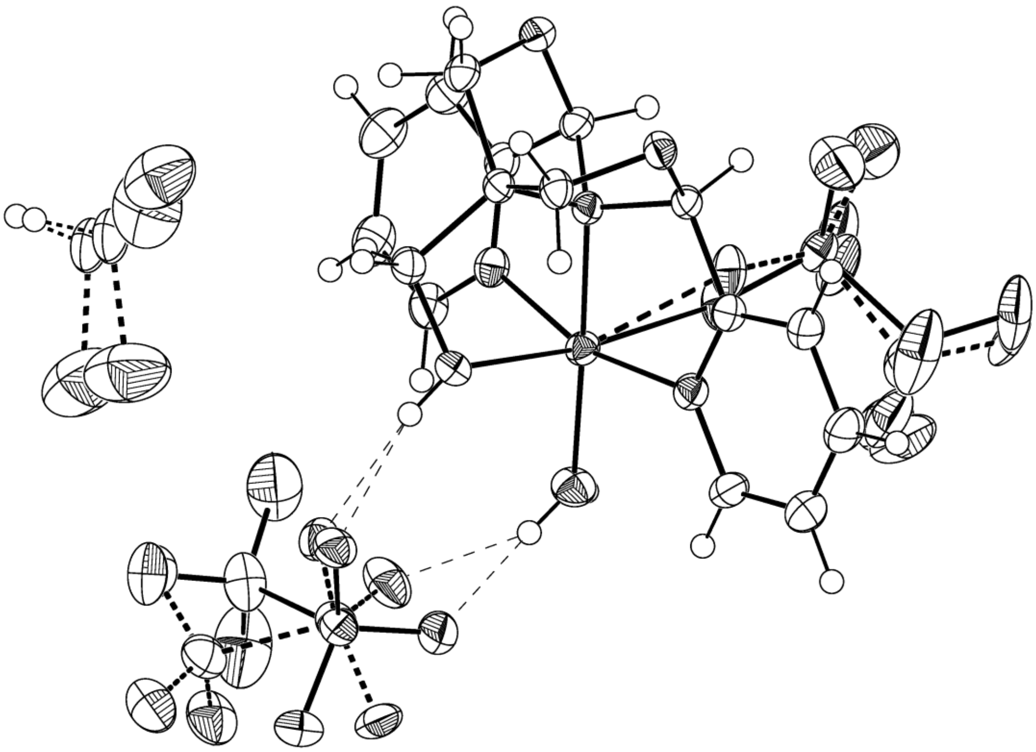

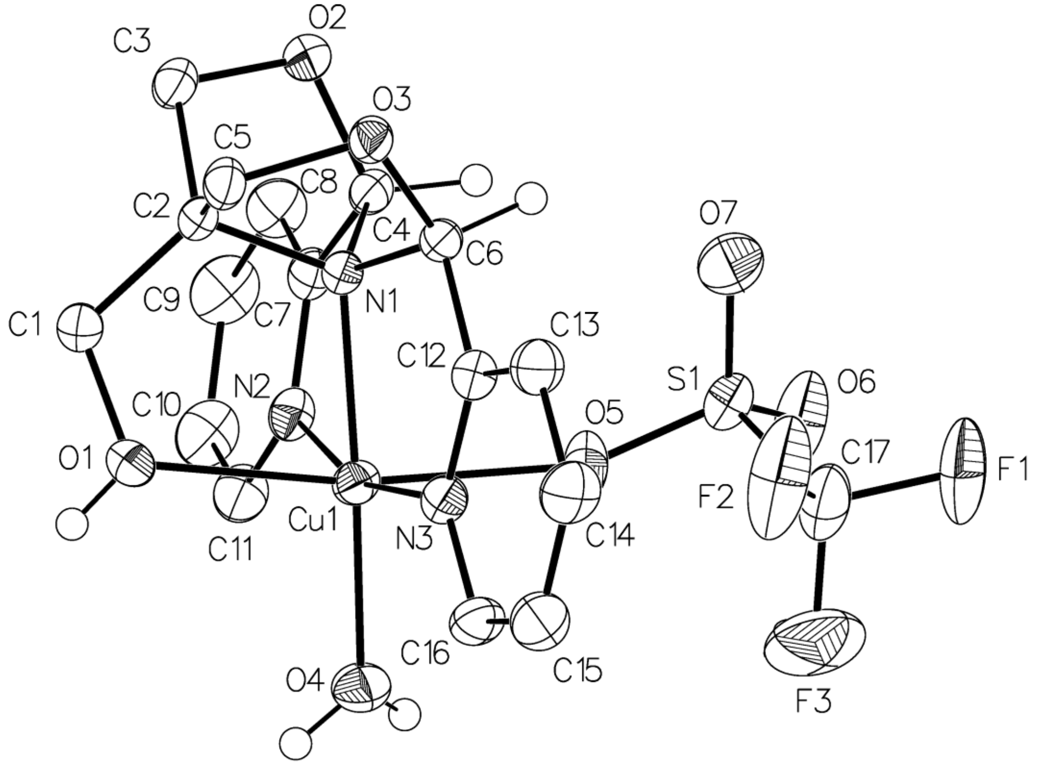
O2
C3
O3
C5
C8
C2
C4
C6
O7
C9
C7
N1
C1
C12
C13
S1
O6
N2
C10
O5
O1
C17
F1
F2
Cu1
N3
C14
C11
C16
C15
O4
F3

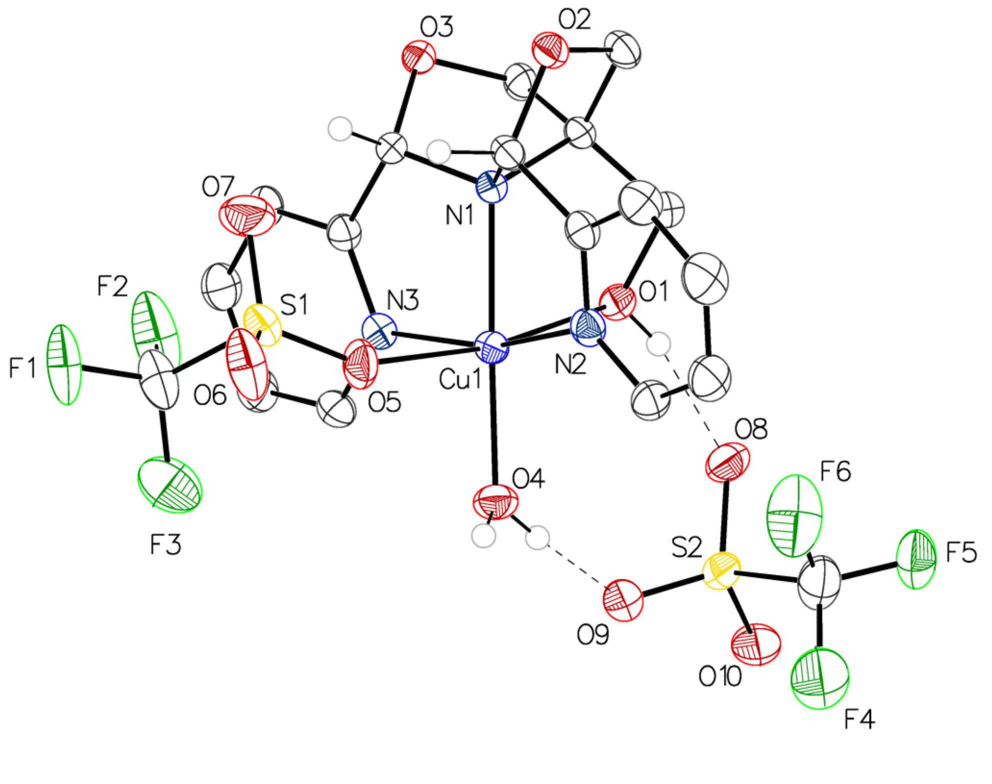
O3
O2
O7
N1
F2
S1
N3
O1
F1
Cu1
N2
O6
O5
O8
F3
O4
F6
S2
F5
O9
O10
F4

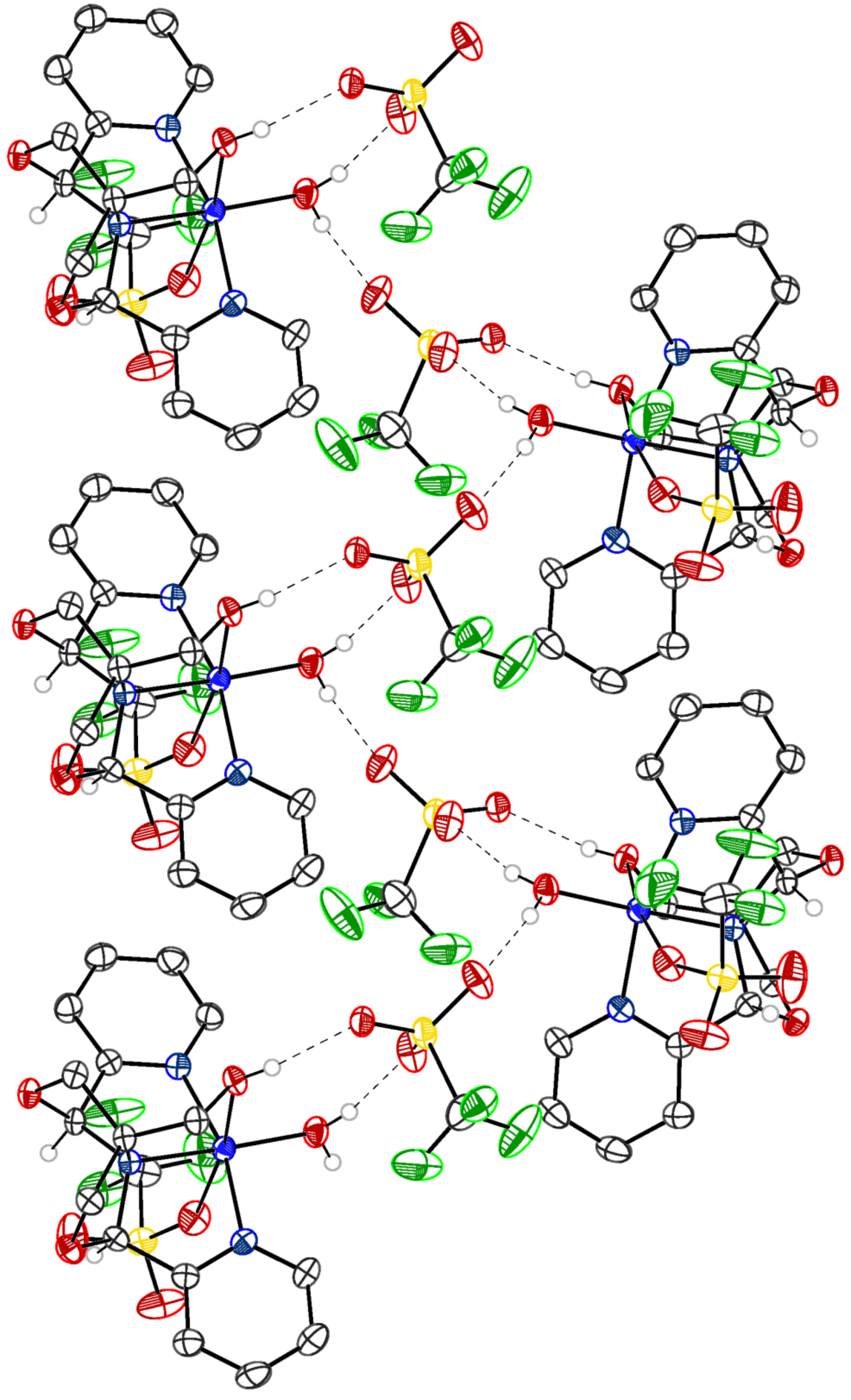

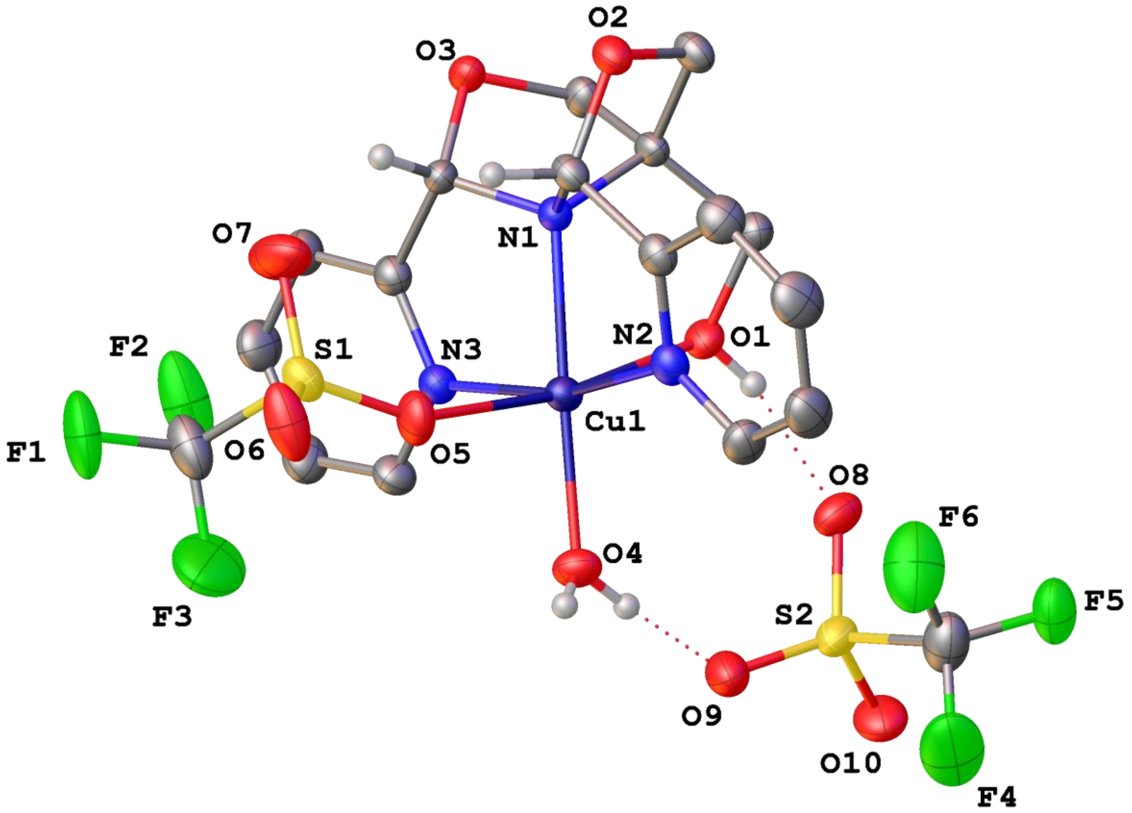
O2
O3
O7
N1
N2
O1
F2
S1
N3
F1
O6
Cu1
O5
O8
O4
F6
F5
S2
F3
O9
O10
F4

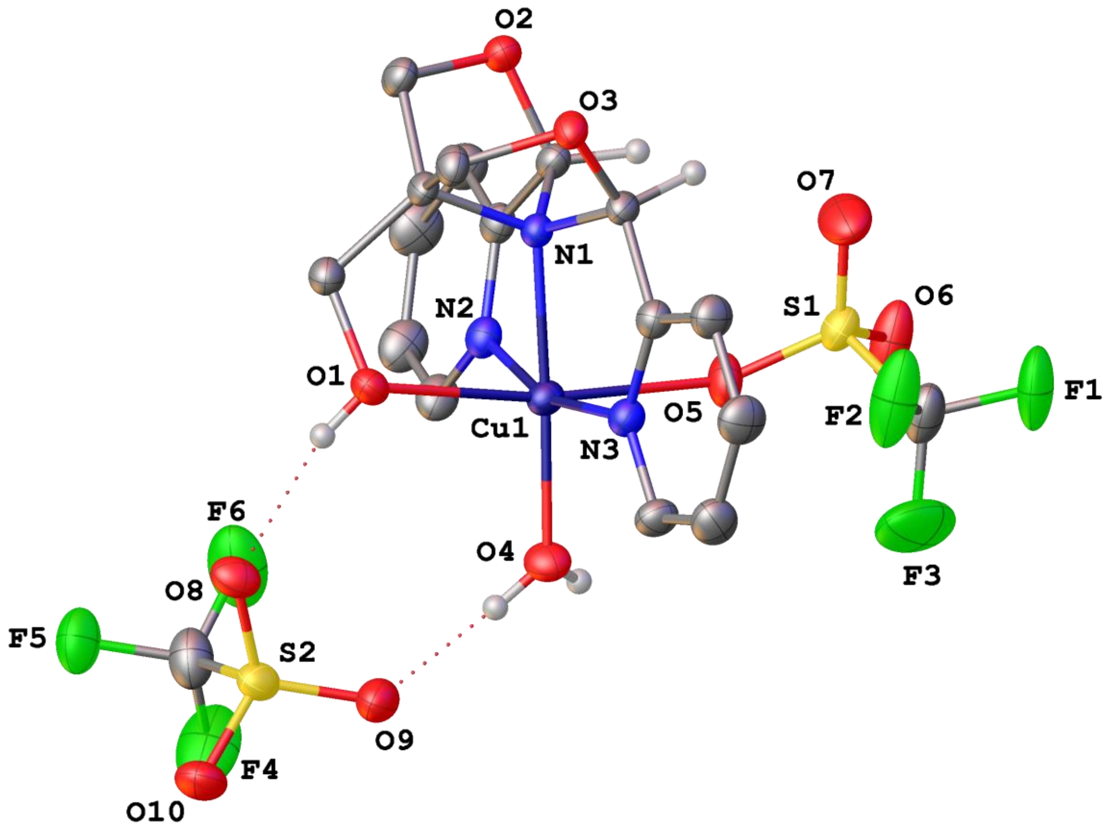
O2
O3
O7
N1
N2
S1
O6
O1
F1
Cu1
O5
F2
N3
F6
O4
F3
O8
F5
S2
O9
F4
O10

Table S15. Crystal data and structure refinement for jonap11.

Identification code	jonap11	
Empirical formula	C18.50 H20 Cl Cu F6 N3 O10 S2	
Formula weight	721.48	
Temperature	99.95(13) K	
Wavelength	1.54184 Å	
Crystal system	monoclinic	
Space group	*C*2/*c*	
Unit cell dimensions	*a* = 18.4521(2) Å	α = 90°
	b = 8.93525(11) Å	β = 100.7028(13)°
	c = 33.1858(4) Å	γ = 90°
Volume	5376.30(12) $Å^3$	
Z	8	
Density (calculated)	1.783 Mg/m^3	
Absorption coefficient	4.445 mm^{-1}	
F(000)	2912	
Crystal color, morphology	blue-green, plate	
Crystal size	0.108 x 0.084 x 0.026 mm^3	
Theta range for data collection	4.878 to 80.213°	
Index ranges	$-23 \le h \le 23$, $-10 \le k \le 7$, $-42 \le l \le 42$	
Reflections collected	29203	
Independent reflections	5723 [*R*(int) = 0.0522]	
Observed reflections	4946	
Completeness to theta = 67.684°	99.9%	
Absorption correction	Multi-scan	
Max. and min. transmission	1.00000 and 0.85475	
Refinement method	Full-matrix least-squares on F^2	
Data / restraints / parameters	5723 / 285 / 565	
Goodness-of-fit on F^2	1.052	
Final *R* indices [*I*>2sigma(*I*)]	*R*1 = 0.0481, *wR*2 = 0.1224	
R indices (all data)	*R*1 = 0.0552, *wR*2 = 0.1264	
Largest diff. peak and hole	0.736 and -0.813 e.$Å^{-3}$	

Table S16. Atomic coordinates (x 10^4) and equivalent isotropic displacement parameters ($Å^2$x 10^3) for jonap11. U_{eq} is defined as one third of the trace of the orthogonalized U_{ij} tensor.

	x	y	z	U_{eq}
Cu1	2585(1)	6685(1)	3706(1)	20(1)
O1	1424(1)	5410(2)	3558(1)	25(1)
O2	1638(1)	8947(2)	4538(1)	24(1)
O3	2030(1)	5563(2)	4781(1)	22(1)
O4	2964(1)	6283(3)	3201(1)	29(1)
N1	2153(1)	7023(3)	4218(1)	18(1)
N2	2189(1)	8728(3)	3570(1)	23(1)
N3	3011(1)	4860(3)	3992(1)	22(1)
C1	955(2)	6327(3)	3749(1)	23(1)
C2	1345(1)	6627(3)	4190(1)	20(1)
C3	1020(2)	8017(3)	4371(1)	24(1)
C4	2184(2)	8648(3)	4303(1)	21(1)
C5	1386(2)	5269(3)	4474(1)	23(1)
C6	2561(2)	6084(3)	4557(1)	19(1)
C7	2046(2)	9481(3)	3897(1)	23(1)
C8	1793(2)	10934(4)	3866(1)	31(1)
C9	1698(2)	11647(4)	3488(1)	38(1)
C10	1852(2)	10876(4)	3155(1)	36(1)
C11	2093(2)	9417(4)	3204(1)	29(1)
C12	2936(2)	4772(3)	4385(1)	22(1)
C13	3205(2)	3574(4)	4632(1)	29(1)
C14	3565(2)	2433(4)	4466(1)	33(1)
C15	3648(2)	2522(4)	4062(1)	33(1)
C16	3365(2)	3750(4)	3832(1)	26(1)
S1	4294(1)	8390(1)	4300(1)	27(1)
O5	3703(2)	7993(5)	3954(1)	34(1)
O6	4704(5)	9672(8)	4223(3)	47(2)
O7	4060(2)	8348(7)	4682(1)	55(1)
C17	4913(3)	6803(5)	4346(2)	40(1)
F1	5534(3)	7087(8)	4609(2)	55(2)
F2	4623(2)	5590(3)	4477(2)	67(1)

F3	5104(3)	6481(6)	3986(1)	85(2)
O5'	3639(10)	7970(30)	4100(6)	33(2)
O6'	4750(20)	9390(40)	4139(13)	38(3)
O7'	4355(13)	8780(20)	4731(6)	55(4)
F1'	5580(12)	6960(30)	4506(6)	40(4)
F2'	4712(6)	5693(13)	4153(6)	60(4)
F3'	5206(7)	7480(19)	3872(4)	60(4)
C17'	4981(10)	7073(17)	4215(5)	47(3)
O8	998(4)	4229(7)	2794(2)	31(1)
S2	1396(2)	4427(4)	2464(1)	29(1)
O9	2177(2)	4698(4)	2592(1)	40(1)
O10	1222(2)	3320(4)	2144(1)	42(1)
C18	1041(2)	6175(6)	2222(1)	42(1)
F4	1378(2)	6536(5)	1918(1)	74(1)
F5	317(4)	6101(7)	2080(3)	44(2)
F6	1167(2)	7271(3)	2499(1)	68(1)
O8'	928(19)	4510(40)	2760(9)	32(3)
S2'	1395(9)	4577(17)	2458(5)	31(4)
O9'	1921(8)	5779(17)	2542(4)	42(3)
O10'	1704(8)	3179(15)	2342(4)	40(3)
C18'	772(8)	5143(17)	1996(4)	34(4)
F4'	1177(7)	5533(17)	1718(3)	47(3)
F5'	308(7)	4059(14)	1853(4)	51(3)
F6'	410(20)	6380(30)	2063(13)	47(6)
Cl1	202(13)	10130(40)	2151(11)	100(6)
Cl2	-90(13)	9980(30)	2981(10)	82(4)
C19	-30(20)	11130(20)	2562(9)	52(8)
Cl1'	350(15)	9680(30)	2336(11)	96(6)
Cl2'	-121(17)	9990(30)	3122(10)	73(4)
C19'	20(50)	10880(40)	2674(15)	51(8)

Table S17. Bond lengths [Å] and angles [°] for jonap11.

Cu(1)-O(1)	2.395(2)	C(6)-C(12)	1.526(4)
Cu(1)-O(4)	1.965(2)	C(7)-C(8)	1.377(4)
Cu(1)-N(1)	2.028(2)	C(8)-H(8)	0.9500
Cu(1)-N(2)	1.987(3)	C(8)-C(9)	1.390(5)
Cu(1)-N(3)	1.976(2)	C(9)-H(9)	0.9500
Cu(1)-O(5)	2.382(4)	C(9)-C(10)	1.375(5)
Cu(1)-O(5')	2.423(19)	C(10)-H(10)	0.9500
O(1)-H(1)	0.80(4)	C(10)-C(11)	1.377(5)
O(1)-C(1)	1.425(4)	C(11)-H(11)	0.9500
O(2)-C(3)	1.436(3)	C(12)-C(13)	1.385(4)
O(2)-C(4)	1.409(3)	C(13)-H(13)	0.9500
O(3)-C(5)	1.440(3)	C(13)-C(14)	1.386(5)
O(3)-C(6)	1.414(3)	C(14)-H(14)	0.9500
O(4)-H(4A)	0.81(5)	C(14)-C(15)	1.382(5)
O(4)-H(4B)	0.82(6)	C(15)-H(15)	0.9500
N(1)-C(2)	1.518(3)	C(15)-C(16)	1.381(4)
N(1)-C(4)	1.478(4)	C(16)-H(16)	0.9500
N(1)-C(6)	1.492(3)	S(1)-O(5)	1.473(4)
N(2)-C(7)	1.344(4)	S(1)-O(6)	1.422(8)
N(2)-C(11)	1.344(4)	S(1)-O(7)	1.412(4)
N(3)-C(12)	1.337(4)	S(1)-C(17)	1.809(5)
N(3)-C(16)	1.350(4)	S(1)-O(5')	1.321(18)
C(1)-H(1A)	0.9900	S(1)-O(6')	1.40(3)
C(1)-H(1B)	0.9900	S(1)-O(7')	1.453(19)
C(1)-C(2)	1.530(4)	S(1)-C(17')	1.789(19)
C(2)-C(3)	1.547(4)	C(17)-F(1)	1.330(5)
C(2)-C(5)	1.529(4)	C(17)-F(2)	1.319(5)
C(3)-H(3A)	0.9900	C(17)-F(3)	1.336(5)
C(3)-H(3B)	0.9900	F(1')-C(17')	1.331(8)
C(4)-H(4)	1.0000	F(2')-C(17')	1.331(8)
C(4)-C(7)	1.517(4)	F(3')-C(17')	1.332(8)
C(5)-H(5A)	0.9900	O(8)-S(2)	1.439(4)
C(5)-H(5B)	0.9900	S(2)-O(9)	1.445(5)
C(6)-H(6)	1.0000	S(2)-O(10)	1.443(5)

S(2)-C(18)	1.820(6)	N(3)-Cu(1)-O(5)	89.80(13)
C(18)-F(4)	1.320(6)	N(3)-Cu(1)-O(5')	85.7(5)
C(18)-F(5)	1.334(9)	O(5)-Cu(1)-O(1)	171.46(12)
C(18)-F(6)	1.333(6)	Cu(1)-O(1)-H(1)	122(3)
O(8')-S(2')	1.440(15)	C(1)-O(1)-Cu(1)	103.23(16)
S(2')-O(9')	1.441(15)	C(1)-O(1)-H(1)	113(3)
S(2')-O(10')	1.453(15)	C(4)-O(2)-C(3)	105.9(2)
S(2')-C(18')	1.807(15)	C(6)-O(3)-C(5)	104.38(19)
C(18')-F(4')	1.338(15)	Cu(1)-O(4)-H(4A)	119(3)
C(18')-F(5')	1.322(15)	Cu(1)-O(4)-H(4B)	127(4)
C(18')-F(6')	1.326(19)	H(4A)-O(4)-H(4B)	106(5)
Cl(1)-C(19)	1.748(10)	C(2)-N(1)-Cu(1)	116.71(16)
Cl(2)-C(19)	1.747(10)	C(4)-N(1)-Cu(1)	107.38(16)
C(19)-H(19A)	0.9900	C(4)-N(1)-C(2)	104.1(2)
C(19)-H(19B)	0.9900	C(4)-N(1)-C(6)	114.3(2)
Cl(1')-C(19')	1.747(11)	C(6)-N(1)-Cu(1)	109.08(16)
Cl(2')-C(19')	1.747(11)	C(6)-N(1)-C(2)	105.4(2)
C(19')-H(19C)	0.9900	C(7)-N(2)-Cu(1)	113.32(19)
C(19')-H(19D)	0.9900	C(7)-N(2)-C(11)	119.2(3)
O(1)-Cu(1)-O(5')	159.1(4)	C(11)-N(2)-Cu(1)	127.4(2)
O(4)-Cu(1)-O(1)	100.61(9)	C(12)-N(3)-Cu(1)	114.37(19)
O(4)-Cu(1)-N(1)	177.11(10)	C(12)-N(3)-C(16)	119.1(3)
O(4)-Cu(1)-N(2)	98.53(10)	C(16)-N(3)-Cu(1)	126.6(2)
O(4)-Cu(1)-N(3)	94.97(10)	O(1)-C(1)-H(1A)	110.1
O(4)-Cu(1)-O(5)	87.82(13)	O(1)-C(1)-H(1B)	110.1
O(4)-Cu(1)-O(5')	99.8(4)	O(1)-C(1)-C(2)	108.1(2)
N(1)-Cu(1)-O(1)	76.55(8)	H(1A)-C(1)-H(1B)	108.4
N(1)-Cu(1)-O(5)	95.01(12)	C(2)-C(1)-H(1A)	110.1
N(1)-Cu(1)-O(5')	83.0(4)	C(2)-C(1)-H(1B)	110.1
N(2)-Cu(1)-O(1)	96.51(9)	N(1)-C(2)-C(1)	112.4(2)
N(2)-Cu(1)-N(1)	82.41(10)	N(1)-C(2)-C(3)	103.6(2)
N(2)-Cu(1)-O(5)	83.46(13)	N(1)-C(2)-C(5)	102.3(2)
N(2)-Cu(1)-O(5')	84.9(5)	C(1)-C(2)-C(3)	111.3(2)
N(3)-Cu(1)-O(1)	88.12(9)	C(5)-C(2)-C(1)	114.2(2)
N(3)-Cu(1)-N(1)	84.46(10)	C(5)-C(2)-C(3)	112.2(2)
N(3)-Cu(1)-N(2)	164.65(10)	O(2)-C(3)-C(2)	106.1(2)

O(2)-C(3)-H(3A)	110.5	N(2)-C(11)-H(11)	119.1
O(2)-C(3)-H(3B)	110.5	C(10)-C(11)-H(11)	119.1
C(2)-C(3)-H(3A)	110.5	N(3)-C(12)-C(6)	117.2(2)
C(2)-C(3)-H(3B)	110.5	N(3)-C(12)-C(13)	122.2(3)
H(3A)-C(3)-H(3B)	108.7	C(13)-C(12)-C(6)	120.6(3)
O(2)-C(4)-N(1)	106.6(2)	C(12)-C(13)-H(13)	120.7
O(2)-C(4)-H(4)	109.8	C(12)-C(13)-C(14)	118.7(3)
O(2)-C(4)-C(7)	112.2(2)	C(14)-C(13)-H(13)	120.7
N(1)-C(4)-H(4)	109.8	C(13)-C(14)-H(14)	120.3
N(1)-C(4)-C(7)	108.6(2)	C(15)-C(14)-C(13)	119.4(3)
C(7)-C(4)-H(4)	109.8	C(15)-C(14)-H(14)	120.3
O(3)-C(5)-C(2)	103.4(2)	C(14)-C(15)-H(15)	120.5
O(3)-C(5)-H(5A)	111.1	C(16)-C(15)-C(14)	118.9(3)
O(3)-C(5)-H(5B)	111.1	C(16)-C(15)-H(15)	120.5
C(2)-C(5)-H(5A)	111.1	N(3)-C(16)-C(15)	121.8(3)
C(2)-C(5)-H(5B)	111.1	N(3)-C(16)-H(16)	119.1
H(5A)-C(5)-H(5B)	109.0	C(15)-C(16)-H(16)	119.1
O(3)-C(6)-N(1)	106.3(2)	O(5)-S(1)-C(17)	103.9(2)
O(3)-C(6)-H(6)	109.9	O(6)-S(1)-O(5)	113.2(4)
O(3)-C(6)-C(12)	110.5(2)	O(6)-S(1)-C(17)	107.4(4)
N(1)-C(6)-H(6)	109.9	O(7)-S(1)-O(5)	112.8(2)
N(1)-C(6)-C(12)	110.4(2)	O(7)-S(1)-O(6)	116.4(4)
C(12)-C(6)-H(6)	109.9	O(7)-S(1)-C(17)	101.5(3)
N(2)-C(7)-C(4)	116.4(3)	O(5')-S(1)-O(6')	123(2)
N(2)-C(7)-C(8)	121.8(3)	O(5')-S(1)-O(7')	117.3(11)
C(8)-C(7)-C(4)	121.8(3)	O(5')-S(1)-C(17')	110.1(10)
C(7)-C(8)-H(8)	120.5	O(6')-S(1)-O(7')	106.1(18)
C(7)-C(8)-C(9)	118.9(3)	O(6')-S(1)-C(17')	82.3(15)
C(9)-C(8)-H(8)	120.5	O(7')-S(1)-C(17')	112.7(10)
C(8)-C(9)-H(9)	120.5	S(1)-O(5)-Cu(1)	148.9(3)
C(10)-C(9)-C(8)	119.1(3)	F(1)-C(17)-S(1)	111.0(4)
C(10)-C(9)-H(9)	120.5	F(1)-C(17)-F(3)	106.8(4)
C(9)-C(10)-H(10)	120.3	F(2)-C(17)-S(1)	112.5(3)
C(9)-C(10)-C(11)	119.3(3)	F(2)-C(17)-F(1)	107.0(4)
C(11)-C(10)-H(10)	120.3	F(2)-C(17)-F(3)	108.1(4)
N(2)-C(11)-C(10)	121.7(3)	F(3)-C(17)-S(1)	111.2(3)

S(1)-O(5')-Cu(1)	166.8(15)	O(9')-S(2')-O(10')	114.1(14)
F(1')-C(17')-S(1)	116.8(15)	O(9')-S(2')-C(18')	104.8(11)
F(1')-C(17')-F(2')	106.2(12)	O(10')-S(2')-C(18')	103.7(11)
F(1')-C(17')-F(3')	107.0(12)	F(4')-C(18')-S(2')	108.1(11)
F(2')-C(17')-S(1)	112.3(12)	F(5')-C(18')-S(2')	111.6(11)
F(2')-C(17')-F(3')	106.7(12)	F(5')-C(18')-F(4')	110.3(13)
F(3')-C(17')-S(1)	107.4(12)	F(5')-C(18')-F(6')	111(2)
O(8)-S(2)-O(9)	114.8(4)	F(6')-C(18')-S(2')	111(2)
O(8)-S(2)-O(10)	113.6(4)	F(6')-C(18')-F(4')	105(2)
O(8)-S(2)-C(18)	104.3(3)	Cl(1)-C(19)-H(19A)	109.1
O(9)-S(2)-C(18)	104.1(3)	Cl(1)-C(19)-H(19B)	109.1
O(10)-S(2)-O(9)	113.9(3)	Cl(2)-C(19)-Cl(1)	112.4(10)
O(10)-S(2)-C(18)	104.5(3)	Cl(2)-C(19)-H(19A)	109.1
F(4)-C(18)-S(2)	111.4(4)	Cl(2)-C(19)-H(19B)	109.1
F(4)-C(18)-F(5)	108.9(5)	H(19A)-C(19)-H(19B)	107.9
F(4)-C(18)-F(6)	107.5(4)	Cl(1')-C(19')-H(19C)	109.0
F(5)-C(18)-S(2)	111.6(4)	Cl(1')-C(19')-H(19D)	109.0
F(6)-C(18)-S(2)	109.0(3)	Cl(2')-C(19')-Cl(1')	112.8(11)
F(6)-C(18)-F(5)	108.4(5)	Cl(2')-C(19')-H(19C)	109.0
O(8')-S(2')-O(9')	111.5(19)	Cl(2')-C(19')-H(19D)	109.0
O(8')-S(2')-O(10')	117.5(17)	H(19C)-C(19')-H(19D)	107.8
O(8')-S(2')-C(18')	103.5(16)		

Table S18. Anisotropic displacement parameters ($Å^2$x 10^3) for jonap11. The anisotropic displacement factor exponent takes the form: $-2\pi^2[h^2 a^{*2}U_{11} + ... + 2 h k a^* b^* U_{12}]$

	U_{11}	U_{22}	U_{33}	U_{23}	U_{13}	U_{12}
Cu1	20(1)	22(1)	20(1)	1(1)	5(1)	2(1)
O1	24(1)	30(1)	20(1)	-5(1)	2(1)	2(1)
O2	21(1)	27(1)	24(1)	-5(1)	4(1)	2(1)
O3	18(1)	32(1)	18(1)	2(1)	4(1)	-1(1)
O4	31(1)	34(1)	24(1)	1(1)	10(1)	-2(1)
N1	16(1)	21(1)	17(1)	0(1)	3(1)	0(1)
N2	18(1)	24(1)	25(1)	3(1)	4(1)	-1(1)
N3	20(1)	25(1)	22(1)	0(1)	4(1)	1(1)
C1	20(1)	29(2)	21(1)	0(1)	3(1)	1(1)
C2	14(1)	27(1)	19(1)	-1(1)	4(1)	-1(1)
C3	17(1)	27(2)	28(1)	-3(1)	6(1)	-1(1)
C4	17(1)	22(1)	24(1)	-3(1)	4(1)	1(1)
C5	16(1)	28(2)	23(1)	2(1)	3(1)	-2(1)
C6	17(1)	25(1)	16(1)	1(1)	3(1)	2(1)
C7	18(1)	23(1)	27(1)	-1(1)	4(1)	-1(1)
C8	33(2)	26(2)	35(2)	0(1)	7(1)	1(1)
C9	42(2)	25(2)	47(2)	9(1)	10(2)	8(1)
C10	35(2)	36(2)	37(2)	13(1)	8(1)	6(1)
C11	27(2)	33(2)	26(1)	9(1)	6(1)	1(1)
C12	19(1)	24(1)	23(1)	0(1)	3(1)	0(1)
C13	28(2)	31(2)	27(1)	3(1)	2(1)	7(1)
C14	34(2)	28(2)	36(2)	5(1)	4(1)	10(1)
C15	33(2)	29(2)	36(2)	-2(1)	4(1)	8(1)
C16	26(1)	26(2)	27(1)	-1(1)	7(1)	6(1)
S1	20(1)	27(1)	34(1)	2(1)	6(1)	1(1)
O5	20(1)	44(2)	35(2)	4(2)	-2(2)	-3(1)
O6	24(2)	32(3)	80(5)	14(2)	-1(2)	-1(2)
O7	36(2)	95(4)	38(2)	-7(2)	16(2)	-14(2)
C17	24(2)	35(2)	59(3)	-4(2)	-1(2)	2(2)
F1	26(2)	46(3)	83(3)	12(3)	-17(2)	1(2)
F2	34(2)	30(2)	129(4)	22(2)	-6(2)	-3(1)

F3	88(3)	94(4)	74(2)	-13(2)	21(2)	52(3)
O5'	17(3)	41(3)	39(4)	5(4)	1(3)	-4(3)
O6'	18(4)	32(6)	65(7)	13(5)	13(4)	-4(5)
O7'	57(10)	55(9)	53(7)	-1(5)	13(5)	2(7)
F1'	12(5)	37(7)	70(7)	-5(6)	11(4)	3(4)
F2'	22(5)	35(5)	119(10)	-17(5)	3(6)	4(4)
F3'	44(6)	66(8)	73(6)	-12(5)	18(5)	5(6)
C17'	29(5)	43(5)	67(6)	-10(4)	2(4)	0(4)
O8	39(2)	33(3)	22(2)	2(2)	6(2)	6(2)
S2	31(1)	34(1)	21(1)	-1(1)	3(1)	7(1)
O9	28(2)	60(2)	30(1)	-6(1)	3(1)	10(1)
O10	42(2)	52(2)	33(2)	-17(1)	6(1)	8(2)
C18	33(2)	48(3)	41(2)	10(2)	0(2)	-4(2)
F4	54(2)	102(3)	69(2)	49(2)	19(2)	-3(2)
F5	33(2)	46(3)	47(2)	14(2)	-3(2)	1(2)
F6	67(2)	34(2)	90(3)	-4(2)	-16(2)	0(1)
O8'	36(5)	38(7)	22(5)	5(5)	7(5)	6(5)
S2'	22(5)	54(6)	19(5)	-2(4)	6(4)	-3(4)
O9'	43(6)	58(7)	24(6)	1(5)	3(5)	-9(6)
O10'	47(7)	40(6)	35(6)	-7(5)	12(6)	24(5)
C18'	34(7)	44(9)	27(7)	-8(6)	11(6)	5(6)
F4'	51(7)	63(8)	28(5)	16(5)	11(5)	-6(6)
F5'	49(7)	62(8)	38(6)	-6(6)	-2(5)	-2(6)
F6'	39(10)	40(10)	58(10)	1(9)	-1(8)	3(9)
Cl1	100(11)	70(9)	133(17)	-14(8)	31(10)	17(7)
Cl2	64(6)	56(6)	123(14)	5(9)	10(9)	-29(5)
C19	46(6)	50(8)	50(30)	-3(11)	-10(20)	22(15)
Cl1'	99(10)	80(10)	117(14)	-39(8)	41(10)	-13(8)
Cl2'	57(5)	61(6)	108(12)	-14(8)	30(8)	-10(5)
C19'	47(9)	49(11)	50(30)	-2(12)	-20(20)	20(15)

Table S19. Hydrogen coordinates (x 10^4) and isotropic displacement parameters (Å^2 x 10^3) for jonap11.

	x	y	z	U(eq)
H1	1240(20)	5170(50)	3331(14)	38(11)
H4A	3240(30)	6900(60)	3125(14)	45(13)
H4B	2740(30)	5900(70)	2991(18)	71(17)
H1A	479	5814	3748	28
H1B	854	7284	3599	28
H3A	679	8558	4154	29
H3B	747	7718	4588	29
H4	2680	8919	4463	25
H5A	939	5193	4598	27
H5B	1444	4331	4324	27
H6	2938	6706	4739	23
H8	1685	11441	4100	37
H9	1529	12652	3460	45
H10	1792	11345	2894	43
H11	2195	8885	2973	34
H13	3144	3535	4910	35
H14	3754	1597	4630	40
H15	3895	1752	3943	39
H16	3422	3815	3554	31
H19A	348	11911	2649	62
H19B	-509	11637	2471	62
H19C	369	11717	2748	61
H19D	-458	11318	2533	61

Table S20. Torsion angles [°] for jonap11.

Cu1-O1-C1-C2	-50.0(2)	C2-N1-C6-C12	103.7(2)
Cu1-N1-C2-C1	-11.5(3)	C3-O2-C4-N1	-38.6(3)
Cu1-N1-C2-C3	-131.75(19)	C3-O2-C4-C7	80.2(3)
Cu1-N1-C2-C5	111.4(2)	C3-C2-C5-O3	-78.4(3)
Cu1-N1-C4-O2	156.51(16)	C4-O2-C3-C2	28.7(3)
Cu1-N1-C4-C7	35.4(2)	C4-N1-C2-C1	106.6(3)
Cu1-N1-C6-O3	-142.29(17)	C4-N1-C2-C3	-13.7(3)
Cu1-N1-C6-C12	-22.4(3)	C4-N1-C2-C5	-130.5(2)
Cu1-N2-C7-C4	-1.9(3)	C4-N1-C6-O3	97.5(3)
Cu1-N2-C7-C8	177.7(2)	C4-N1-C6-C12	-142.6(2)
Cu1-N2-C11-C10	-176.2(2)	C4-C7-C8-C9	178.2(3)
Cu1-N3-C12-C6	-1.8(3)	C5-O3-C6-N1	37.5(3)
Cu1-N3-C12-C13	-179.7(2)	C5-O3-C6-C12	-82.3(3)
Cu1-N3-C16-C15	179.5(2)	C5-C2-C3-O2	101.2(3)
O1-C1-C2-N1	44.4(3)	C6-O3-C5-C2	-43.6(3)
O1-C1-C2-C3	160.1(2)	C6-N1-C2-C1	-132.7(2)
O1-C1-C2-C5	-71.5(3)	C6-N1-C2-C3	107.0(2)
O2-C4-C7-N2	-140.7(2)	C6-N1-C2-C5	-9.8(3)
O2-C4-C7-C8	39.8(4)	C6-N1-C4-O2	-82.3(3)
O3-C6-C12-N3	134.0(2)	C6-N1-C4-C7	156.6(2)
O3-C6-C12-C13	-48.1(3)	C6-C12-C13-C14	-177.6(3)
N1-C2-C3-O2	-8.4(3)	C7-N2-C11-C10	-0.1(5)
N1-C2-C5-O3	32.0(3)	C7-C8-C9-C10	0.6(5)
N1-C4-C7-N2	-23.1(3)	C8-C9-C10-C11	0.3(5)
N1-C4-C7-C8	157.4(3)	C9-C10-C11-N2	-0.6(5)
N1-C6-C12-N3	16.7(3)	C11-N2-C7-C4	-178.5(3)
N1-C6-C12-C13	-165.5(3)	C11-N2-C7-C8	1.0(4)
N2-C7-C8-C9	-1.3(5)	C12-N3-C16-C15	0.4(5)
N3-C12-C13-C14	0.1(5)	C12-C13-C14-C15	0.2(5)
C1-C2-C3-O2	-129.4(2)	C13-C14-C15-C16	-0.2(5)
C1-C2-C5-O3	153.7(2)	C14-C15-C16-N3	-0.1(5)
C2-N1-C4-O2	32.2(3)	C16-N3-C12-C6	177.4(3)
C2-N1-C4-C7	-88.9(2)	C16-N3-C12-C13	-0.4(4)
C2-N1-C6-O3	-16.2(3)	O5-S1-C17-F1	170.9(4)

O5-S1-C17-F2	-69.2(4)	O7'-S1-C17'-F3'	-140.5(14)
O5-S1-C17-F3	52.2(4)	C17'-S1-O5'-Cu1	10(6)
O6-S1-O5-Cu1	-161.7(6)	O8-S2-C18-F4	177.8(5)
O6-S1-C17-F1	50.7(6)	O8-S2-C18-F5	-60.3(6)
O6-S1-C17-F2	170.6(5)	O8-S2-C18-F6	59.4(5)
O6-S1-C17-F3	-68.0(6)	O9-S2-C18-F4	57.1(5)
O7-S1-O5-Cu1	-26.9(7)	O9-S2-C18-F5	179.1(5)
O7-S1-C17-F1	-71.9(5)	O9-S2-C18-F6	-61.3(4)
O7-S1-C17-F2	48.0(4)	O10-S2-C18-F4	-62.7(5)
O7-S1-C17-F3	169.4(4)	O10-S2-C18-F5	59.3(6)
C17-S1-O5-Cu1	82.1(5)	O10-S2-C18-F6	179.0(4)
O5'-S1-C17'-F1'	-153.4(14)	O8'-S2'-C18'-F4'	167.7(19)
O5'-S1-C17'-F2'	-30.5(14)	O8'-S2'-C18'-F5'	-71(2)
O5'-S1-C17'-F3'	86.5(13)	O8'-S2'-C18'-F6'	53(3)
O6'-S1-O5'-Cu1	104(6)	O9'-S2'-C18'-F4'	50.8(16)
O6'-S1-C17'-F1'	84(2)	O9'-S2'-C18'-F5'	172.3(13)
O6'-S1-C17'-F2'	-153(2)	O9'-S2'-C18'-F6'	-64(2)
O6'-S1-C17'-F3'	-36(2)	O10'-S2'-C18'-F4'	-69.2(15)
O7'-S1-O5'-Cu1	-121(5)	O10'-S2'-C18'-F5'	52.3(15)
O7'-S1-C17'-F1'	-20.3(16)	O10'-S2'-C18'-F6'	177(2)
O7'-S1-C17'-F2'	102.6(15)		

Table S21. Hydrogen bonds and close contacts for jonap11 [Å and °].

D-H...A	d(D-H)	d(H...A)	d(D...A)	<(DHA)
O1-H1...O8	0.80(4)	1.94(5)	2.723(8)	166(4)
O1-H1...O8'	0.80(4)	1.97(6)	2.76(4)	172(4)
O4-H4A...O10#1	0.81(5)	1.94(5)	2.742(4)	171(5)
O4-H4A...O10'#1	0.81(5)	1.95(5)	2.628(12)	141(4)
O4-H4B...O9	0.82(6)	1.86(6)	2.666(4)	168(6)
O4-H4B...O9'	0.82(6)	1.91(6)	2.668(13)	153(6)

Symmetry transformations used to generate equivalent atoms:

#1 -x+1/2,y+1/2,-z+1/2

REFERENCE NUMBER: jonap19

4a

CRYSTAL STRUCTURE REPORT

C_{16} H_{17} Br_2 N_3 O_3 Zn

or

(κ^3-L)$ZnBr_2$

Report prepared for:

C. Wood, A. Panda, Prof. W. Jones

June 12, 2023

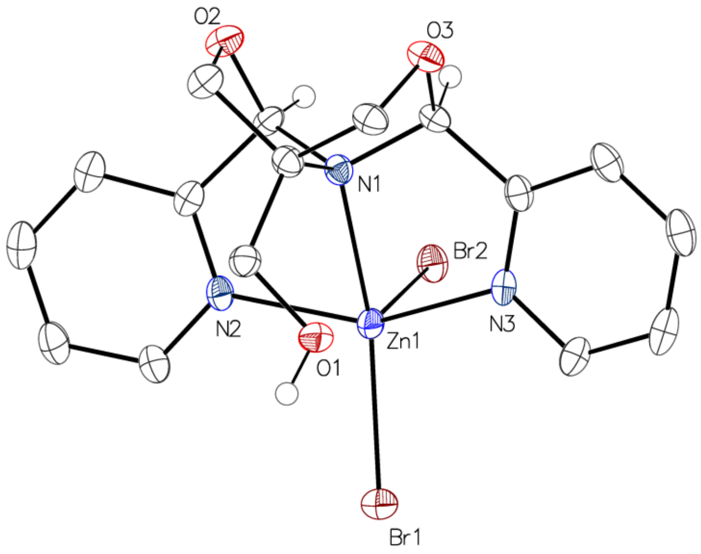

William W. Brennessel

X-ray Crystallographic Facility

Department of Chemistry, University of Rochester

120 Trustee Road

Rochester, NY 14627

Data collection

A crystal (0.226 x 0.027 x 0.018 mm^3) was placed onto a nylon loop and mounted on a Rigaku XtaLAB Synergy-S Dualflex diffractometer equipped with a HyPix-6000HE HPC area detector for data collection at 100.00(10) K. A preliminary set of cell constants and an orientation matrix were calculated from a small sampling of reflections.[1] A short pre-experiment was run, from which an optimal data collection strategy was determined. The full data collection was carried out using a PhotonJet (Cu) X-ray source with frame times of 0.22 and 0.89 seconds and a detector distance of 34.0 mm. Series of frames were collected in 0.50º steps in ω at different 2θ, κ, and ϕ settings. After the intensity data were corrected for absorption, the final cell constants were calculated from the xyz centroids of 18652 strong reflections from the actual data collection after integration.[1] See Table S22 for additional crystal and refinement information.

Structure solution and refinement

The structure was solved using SHELXT[2] and refined using SHELXL.[3] The space group $P2_1/n$ was determined based on systematic absences. Most or all non-hydrogen atoms were assigned from the solution. Full-matrix least squares / difference Fourier cycles were performed which located any remaining non-hydrogen atoms. All non-hydrogen atoms were refined with anisotropic displacement parameters. The O-H hydrogen atom was found from the difference Fourier map and refined freely. All other hydrogen atoms were placed in ideal positions and refined as riding atoms with relative isotropic displacement parameters. The final full matrix least squares refinement converged to $R1 = 0.0324$ (F^2, $I > 2\sigma(I)$) and $wR2 = 0.0876$ (F^2, all data).

Structure description

The structure is the one suggested. The asymmetric unit contains one molecule in a general position. Molecules are linked in one dimension along [010] via O-H…O hydrogen bonding (see figure and Table S28). Additional metrics related to the pendant hydroxide group:

Zn1…O1: 2.557(3) Å

O1…Zn1-Br1: 88.90(6) degrees
O1…Zn1-Br2: 165.25(6) degrees
O1…Zn1-N1: 67.72(9) degrees
O1…Zn1-N2: 87.31(9) degrees
O1…Zn1-N3: 78.82(9) degrees

Structure manipulation and figure generation were performed using Olex2.[4] Unless noted otherwise all structural diagrams containing anisotropic displacement ellipsoids are drawn at the 50 % probability level.

Data collection, structure solution, and structure refinement were conducted at the X-ray Crystallographic Facility, B04 Hutchison Hall, Department of Chemistry, University of Rochester. The instrument was purchased with

funding from NSF MRI program grant CHE-1725028. All publications arising from this report MUST either 1) include William W. Brennessel as a coauthor or 2) acknowledge William W. Brennessel and the X-ray Crystallographic Facility of the Department of Chemistry at the University of Rochester.

---

[1] *CrysAlisPro*, version 171.42.90a; Rigaku Corporation: Oxford, UK, 2023.

[2] Sheldrick, G. M. *SHELXT*, version 2018/2; *Acta. Crystallogr.* **2015**, *A71*, 3-8.

[3] Sheldrick, G. M. *SHELXL*, version 2019/2; *Acta. Crystallogr.* **2015**, *C71*, 3-8.

[4] Dolomanov, O. V.; Bourhis, L. J.; Gildea, R. J.; Howard, J. A. K.; Puschmann, H. *Olex2*, version 1.5; *J. Appl. Cryst.* **2009**, *42*, 339-341.

Some equations of interest:

$$R_{int} = \Sigma\, |F_o^2 - \langle F_o^2 \rangle| \,/\, \Sigma |F_o^2|$$

$$R1 = \Sigma||F_o|-|F_c|| \,/\, \Sigma|F_o|$$

$$wR2 = [\Sigma[w(F_o^2-F_c^2)^2] \,/\, \Sigma\, [w(F_o^2)^2]]^{1/2}$$

$$\text{where } w = 1 / [\sigma^2 (F_o^2) + (aP)^2 + bP] \text{ and}$$

$$P = 1/3 \max(0, F_o^2) + 2/3\, F_c^2$$

$$\text{GOF} = S = [\Sigma[w(F_o^2-F_c^2)^2] \,/\, (m-n)]^{1/2}$$

where m = number of reflections and n = number of parameters

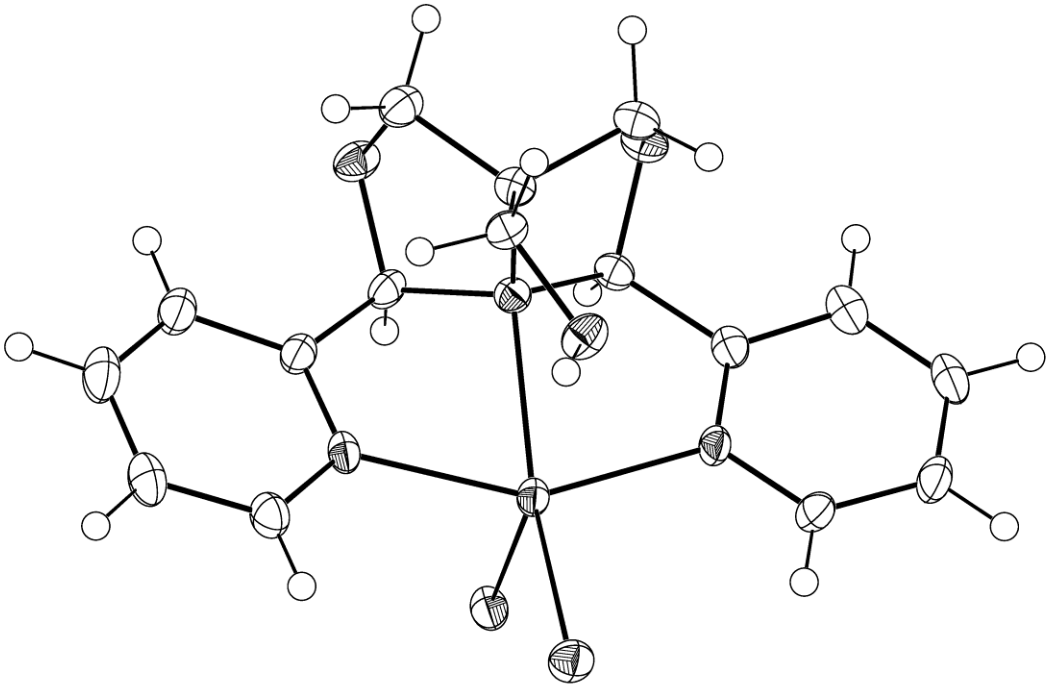

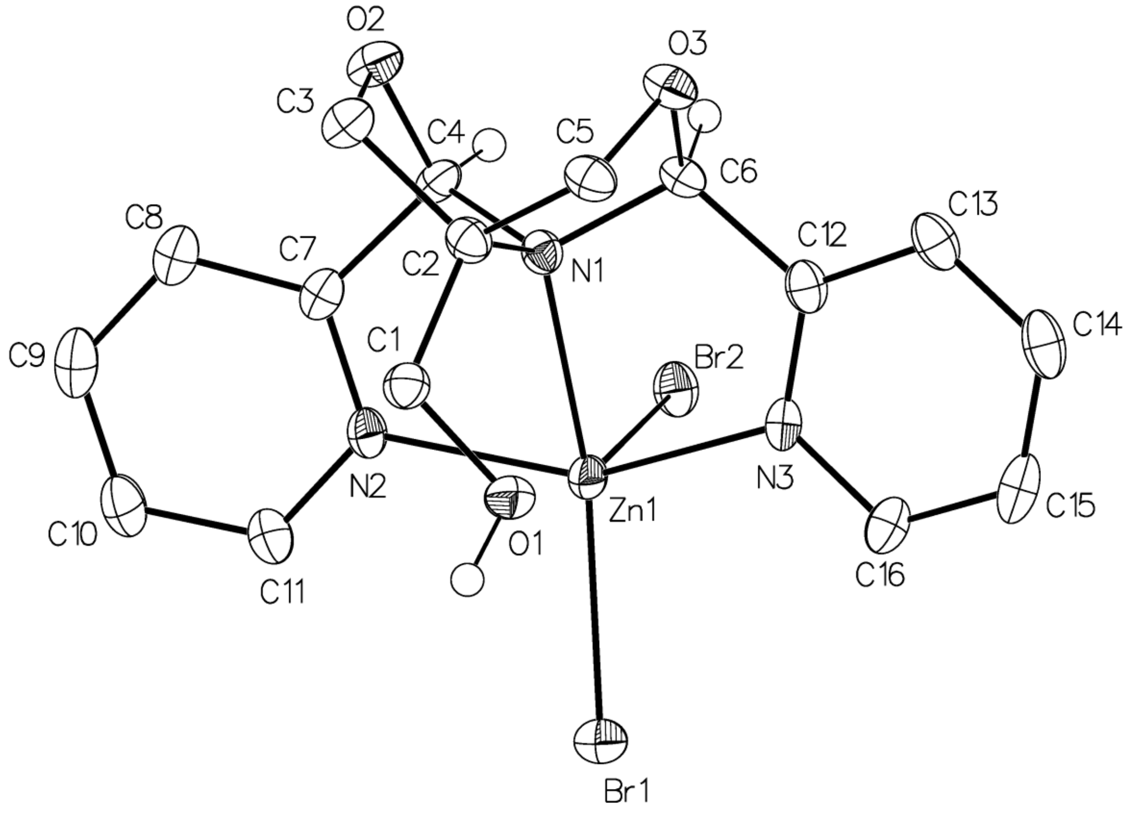
O2
O3
C3
C4
C5
C6
C8
C7
C2
N1
C12
C13
C1
C14
C9
Br2
N2
N3
Zn1
C15
C10
O1
C16
C11
Br1

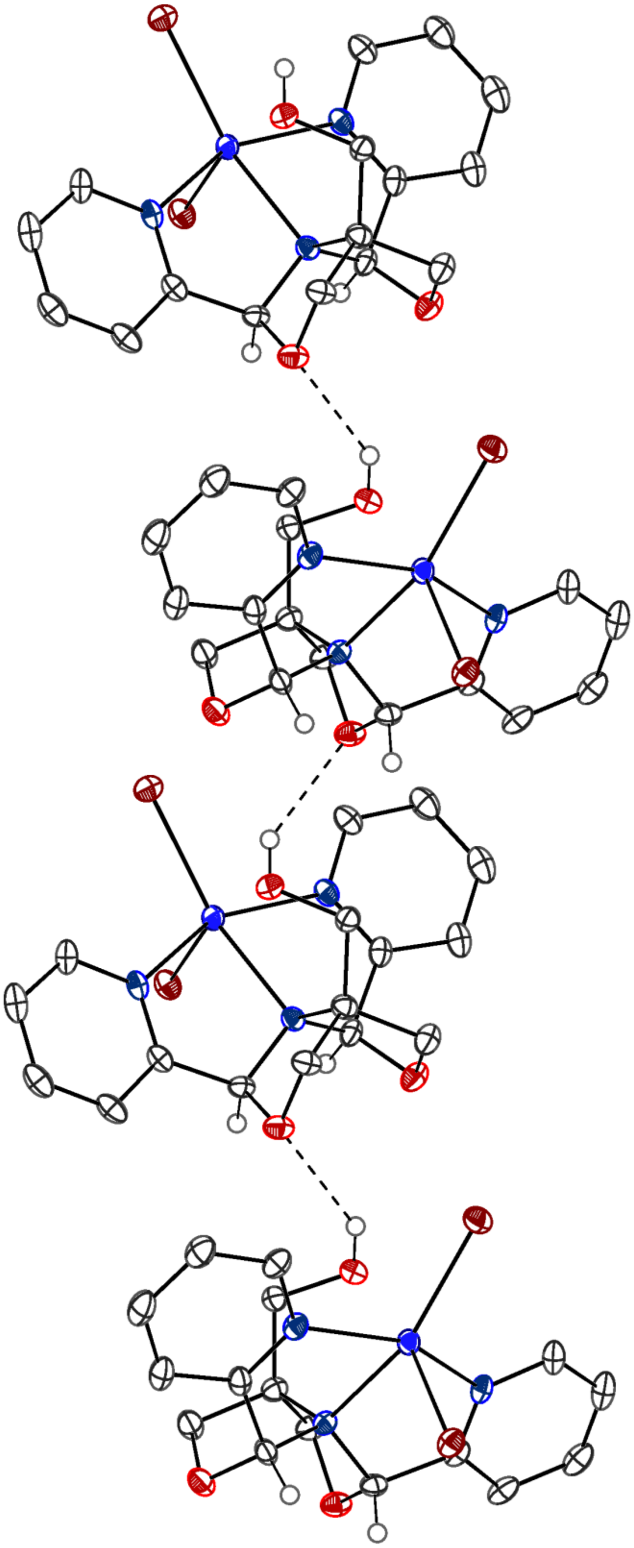

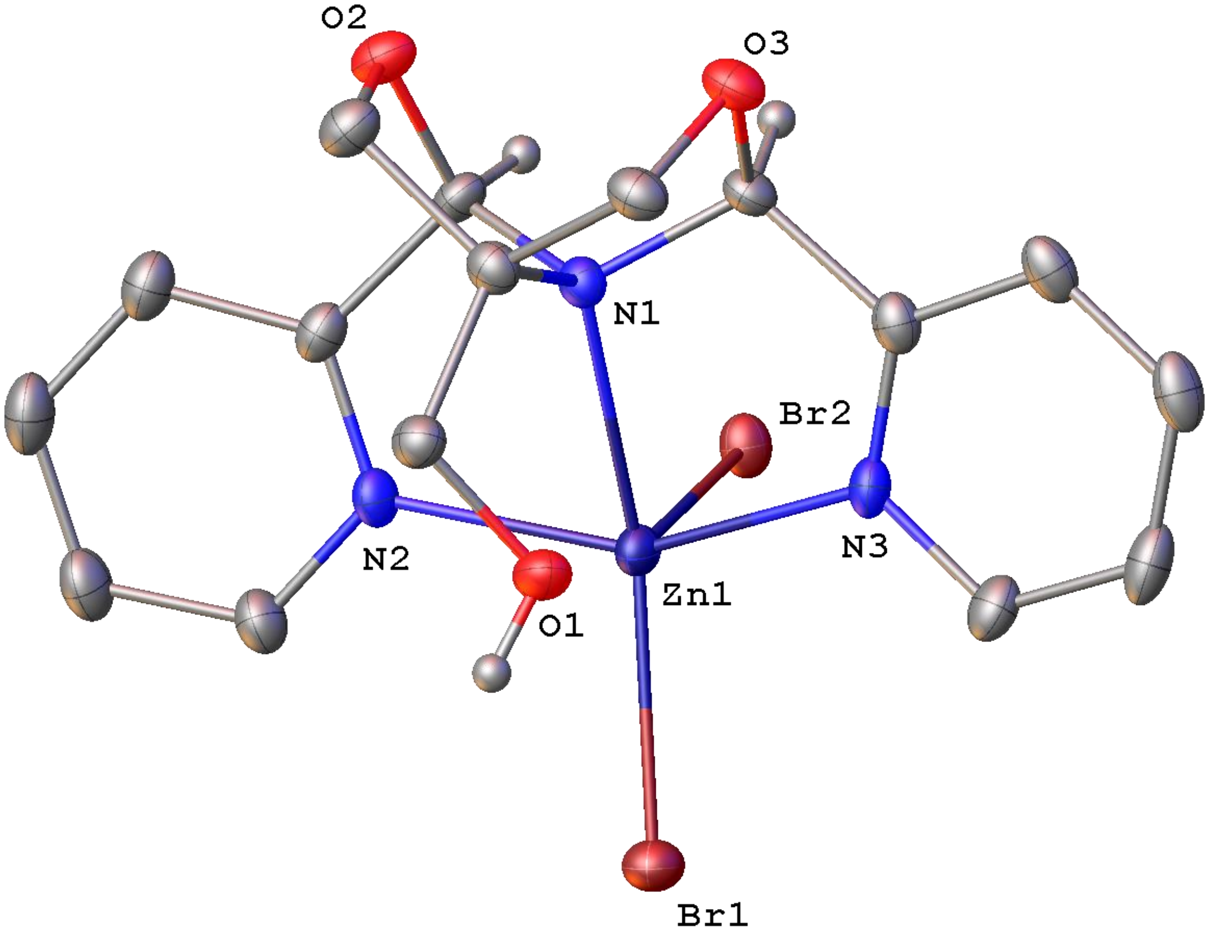
O2
O3
N1
Br2
N2
N3
Zn1
O1
Br1

Table S22. Crystal data and structure refinement for jonap19.

Identification code	jonap19	
Empirical formula	C16 H17 Br2 N3 O3 Zn	
Formula weight	524.52	
Temperature	100.00(10) K	
Wavelength	1.54184 Å	
Crystal system	monoclinic	
Space group	$P2_1/n$	
Unit cell dimensions	*a* = 8.76440(10) Å	α = 90°
	b = 13.05600(10) Å	β = 93.5050(10)°
	c = 15.1824(2) Å	γ = 90°
Volume	1734.04(3) $Å^3$	
Z	4	
Density (calculated)	2.009 Mg/m^3	
Absorption coefficient	7.559 mm^{-1}	
F(000)	1032	
Crystal color, morphology	colourless, needle	
Crystal size	0.226 x 0.027 x 0.018 mm^3	
Theta range for data collection	4.470 to 80.067°	
Index ranges	$-10 \le h \le 11$, $-14 \le k \le 16$, $-19 \le l \le 18$	
Reflections collected	29959	
Independent reflections	3755 [*R*(int) = 0.0484]	
Observed reflections	3498	
Completeness to theta = 74.504°	100.0%	
Absorption correction	Multi-scan	
Max. and min. transmission	1.00000 and 0.70024	
Refinement method	Full-matrix least-squares on F^2	
Data / restraints / parameters	3755 / 0 / 230	
Goodness-of-fit on F^2	1.151	
Final *R* indices [*I*>2sigma(*I*)]	*R*1 = 0.0324, *wR*2 = 0.0863	
R indices (all data)	*R*1 = 0.0349, *wR*2 = 0.0876	
Largest diff. peak and hole	0.596 and -0.812 e.$Å^{-3}$	

Table S23. Atomic coordinates (x 10^4) and equivalent isotropic displacement parameters ($Å^2$x 10^3) for jonap19. U_{eq} is defined as one third of the trace of the orthogonalized U_{ij} tensor.

	x	y	z	U_{eq}
Br1	7271(1)	884(1)	5055(1)	24(1)
Br2	5488(1)	3602(1)	4536(1)	21(1)
Zn1	6761(1)	2513(1)	5745(1)	17(1)
O1	8134(3)	1855(2)	7168(2)	23(1)
O2	5002(3)	4455(2)	7754(2)	23(1)
O3	8408(3)	4927(2)	7478(2)	22(1)
N1	6742(3)	3694(2)	6874(2)	16(1)
N2	4690(3)	2248(2)	6345(2)	18(1)
N3	8878(3)	3258(2)	5709(2)	18(1)
C1	7389(4)	2244(3)	7906(2)	19(1)
C2	7226(4)	3397(2)	7809(2)	18(1)
C3	5934(4)	3839(3)	8358(2)	22(1)
C4	5130(4)	3974(3)	6925(2)	18(1)
C5	8704(4)	4010(2)	7987(2)	20(1)
C6	7761(4)	4539(2)	6657(2)	17(1)
C7	4189(4)	2987(3)	6869(2)	19(1)
C8	2892(4)	2859(3)	7343(2)	24(1)
C9	2085(4)	1948(3)	7257(3)	29(1)
C10	2572(4)	1194(3)	6693(3)	27(1)
C11	3890(4)	1371(3)	6250(2)	23(1)
C12	9069(4)	4149(3)	6124(2)	19(1)
C13	10416(4)	4711(3)	6102(2)	22(1)
C14	11588(4)	4327(3)	5627(2)	25(1)
C15	11402(4)	3386(3)	5213(2)	26(1)
C16	10029(4)	2870(3)	5259(2)	22(1)

Table S24. Bond lengths [Å] and angles [°] for jonap19.

Br(1)-Zn(1)	2.4246(5)	C(9)-H(9)	0.9500
Br(2)-Zn(1)	2.5270(5)	C(9)-C(10)	1.390(6)
Zn(1)-N(1)	2.307(3)	C(10)-H(10)	0.9500
Zn(1)-N(2)	2.108(3)	C(10)-C(11)	1.390(5)
Zn(1)-N(3)	2.099(3)	C(11)-H(11)	0.9500
O(1)-C(1)	1.424(4)	C(12)-C(13)	1.392(4)
O(1)-H(1)	0.85(6)	C(13)-H(13)	0.9500
O(2)-C(3)	1.437(4)	C(13)-C(14)	1.385(5)
O(2)-C(4)	1.417(4)	C(14)-H(14)	0.9500
O(3)-C(5)	1.440(4)	C(14)-C(15)	1.384(6)
O(3)-C(6)	1.430(4)	C(15)-H(15)	0.9500
N(1)-C(2)	1.507(4)	C(15)-C(16)	1.385(5)
N(1)-C(4)	1.466(4)	C(16)-H(16)	0.9500
N(1)-C(6)	1.470(4)	Br(1)-Zn(1)-Br(2)	105.340(19)
N(2)-C(7)	1.341(4)	N(1)-Zn(1)-Br(1)	156.56(7)
N(2)-C(11)	1.346(4)	N(1)-Zn(1)-Br(2)	98.10(7)
N(3)-C(12)	1.328(4)	N(2)-Zn(1)-Br(1)	103.26(8)
N(3)-C(16)	1.352(4)	N(2)-Zn(1)-Br(2)	93.02(8)
C(1)-H(1A)	0.9900	N(2)-Zn(1)-N(1)	75.10(10)
C(1)-H(1B)	0.9900	N(3)-Zn(1)-Br(1)	102.04(8)
C(1)-C(2)	1.518(4)	N(3)-Zn(1)-Br(2)	94.14(8)
C(2)-C(3)	1.558(5)	N(3)-Zn(1)-N(1)	75.87(10)
C(2)-C(5)	1.532(4)	N(3)-Zn(1)-N(2)	150.80(11)
C(3)-H(3A)	0.9900	C(1)-O(1)-H(1)	109(4)
C(3)-H(3B)	0.9900	C(4)-O(2)-C(3)	104.3(2)
C(4)-H(4)	1.0000	C(6)-O(3)-C(5)	102.8(2)
C(4)-C(7)	1.529(5)	C(2)-N(1)-Zn(1)	120.89(19)
C(5)-H(5A)	0.9900	C(4)-N(1)-Zn(1)	104.87(18)
C(5)-H(5B)	0.9900	C(4)-N(1)-C(2)	103.4(2)
C(6)-H(6)	1.0000	C(4)-N(1)-C(6)	115.1(3)
C(6)-C(12)	1.530(4)	C(6)-N(1)-Zn(1)	107.61(18)
C(7)-C(8)	1.392(4)	C(6)-N(1)-C(2)	105.4(2)
C(8)-H(8)	0.9500	C(7)-N(2)-Zn(1)	117.4(2)
C(8)-C(9)	1.386(5)	C(7)-N(2)-C(11)	119.2(3)

C(11)-N(2)-Zn(1)	123.4(2)	O(3)-C(6)-H(6)	110.5
C(12)-N(3)-Zn(1)	118.7(2)	O(3)-C(6)-C(12)	107.9(3)
C(12)-N(3)-C(16)	119.5(3)	N(1)-C(6)-H(6)	110.5
C(16)-N(3)-Zn(1)	121.9(2)	N(1)-C(6)-C(12)	110.7(3)
O(1)-C(1)-H(1A)	109.9	C(12)-C(6)-H(6)	110.5
O(1)-C(1)-H(1B)	109.9	N(2)-C(7)-C(4)	116.4(3)
O(1)-C(1)-C(2)	108.8(3)	N(2)-C(7)-C(8)	121.8(3)
H(1A)-C(1)-H(1B)	108.3	C(8)-C(7)-C(4)	121.8(3)
C(2)-C(1)-H(1A)	109.9	C(7)-C(8)-H(8)	120.6
C(2)-C(1)-H(1B)	109.9	C(9)-C(8)-C(7)	118.9(3)
N(1)-C(2)-C(1)	111.4(3)	C(9)-C(8)-H(8)	120.6
N(1)-C(2)-C(3)	103.7(3)	C(8)-C(9)-H(9)	120.3
N(1)-C(2)-C(5)	102.6(2)	C(8)-C(9)-C(10)	119.5(3)
C(1)-C(2)-C(3)	112.5(3)	C(10)-C(9)-H(9)	120.3
C(1)-C(2)-C(5)	115.3(3)	C(9)-C(10)-H(10)	120.8
C(5)-C(2)-C(3)	110.2(3)	C(9)-C(10)-C(11)	118.4(3)
O(2)-C(3)-C(2)	105.6(3)	C(11)-C(10)-H(10)	120.8
O(2)-C(3)-H(3A)	110.6	N(2)-C(11)-C(10)	122.2(3)
O(2)-C(3)-H(3B)	110.6	N(2)-C(11)-H(11)	118.9
C(2)-C(3)-H(3A)	110.6	C(10)-C(11)-H(11)	118.9
C(2)-C(3)-H(3B)	110.6	N(3)-C(12)-C(6)	117.7(3)
H(3A)-C(3)-H(3B)	108.8	N(3)-C(12)-C(13)	122.2(3)
O(2)-C(4)-N(1)	106.6(3)	C(13)-C(12)-C(6)	120.1(3)
O(2)-C(4)-H(4)	110.6	C(12)-C(13)-H(13)	120.7
O(2)-C(4)-C(7)	110.5(3)	C(14)-C(13)-C(12)	118.6(3)
N(1)-C(4)-H(4)	110.6	C(14)-C(13)-H(13)	120.7
N(1)-C(4)-C(7)	107.7(3)	C(13)-C(14)-H(14)	120.4
C(7)-C(4)-H(4)	110.6	C(15)-C(14)-C(13)	119.2(3)
O(3)-C(5)-C(2)	102.6(3)	C(15)-C(14)-H(14)	120.4
O(3)-C(5)-H(5A)	111.3	C(14)-C(15)-H(15)	120.4
O(3)-C(5)-H(5B)	111.3	C(14)-C(15)-C(16)	119.2(3)
C(2)-C(5)-H(5A)	111.3	C(16)-C(15)-H(15)	120.4
C(2)-C(5)-H(5B)	111.3	N(3)-C(16)-C(15)	121.4(3)
H(5A)-C(5)-H(5B)	109.2	N(3)-C(16)-H(16)	119.3
O(3)-C(6)-N(1)	106.5(2)	C(15)-C(16)-H(16)	119.3

Table S25. Anisotropic displacement parameters ($Å^2$x 10^3) for jonap19. The anisotropic displacement factor exponent takes the form: $-2\pi^2$[h^2 $a^{*2}U_{11}$ + ... + 2 h k a* b* U_{12}]

	U_{11}	U_{22}	U_{33}	U_{23}	U_{13}	U_{12}
Br1	26(1)	19(1)	26(1)	-4(1)	4(1)	0(1)
Br2	17(1)	24(1)	21(1)	4(1)	0(1)	-2(1)
Zn1	14(1)	18(1)	19(1)	-1(1)	3(1)	-2(1)
O1	28(1)	17(1)	26(1)	0(1)	7(1)	3(1)
O2	25(1)	23(1)	20(1)	-2(1)	2(1)	7(1)
O3	27(1)	16(1)	22(1)	-1(1)	-2(1)	-2(1)
N1	17(1)	16(1)	14(1)	1(1)	1(1)	-1(1)
N2	13(1)	21(1)	21(1)	2(1)	1(1)	-3(1)
N3	14(1)	23(1)	16(1)	3(1)	3(1)	-2(1)
C1	22(2)	19(2)	17(1)	1(1)	2(1)	1(1)
C2	21(2)	17(2)	17(2)	2(1)	0(1)	-1(1)
C3	23(2)	25(2)	20(2)	-1(1)	3(1)	4(1)
C4	17(2)	20(2)	18(2)	-1(1)	4(1)	5(1)
C5	24(2)	17(2)	19(2)	0(1)	-2(1)	-1(1)
C6	19(2)	13(1)	19(2)	-2(1)	0(1)	-2(1)
C7	16(1)	25(2)	17(1)	2(1)	3(1)	3(1)
C8	19(2)	32(2)	22(2)	3(1)	5(1)	1(1)
C9	20(2)	39(2)	30(2)	9(2)	6(1)	-3(2)
C10	20(2)	28(2)	32(2)	5(2)	2(1)	-6(1)
C11	21(2)	23(2)	25(2)	2(1)	2(1)	-5(1)
C12	17(1)	21(2)	18(2)	5(1)	0(1)	-2(1)
C13	20(2)	21(2)	25(2)	8(1)	-4(1)	-3(1)
C14	17(2)	32(2)	24(2)	11(1)	-4(1)	-8(1)
C15	17(2)	45(2)	18(2)	6(2)	5(1)	1(1)
C16	20(2)	31(2)	16(2)	0(1)	4(1)	0(1)

Table S26. Hydrogen coordinates (x 10^4) and isotropic displacement parameters (Å^2 x 10^3) for jonap19.

	x	y	z	U(eq)
H1A	7996	2078	8459	23
H1B	6368	1925	7932	23
H3A	5324	3279	8601	27
H3B	6372	4261	8853	27
H4	4800	4451	6434	22
H5A	8882	4168	8623	24
H5B	9598	3635	7782	24
H6	7174	5088	6324	21
H8	2566	3387	7719	29
H9	1206	1840	7582	35
H10	2018	572	6611	32
H11	4237	856	5868	27
H13	10528	5345	6406	26
H14	12508	4704	5586	30
H15	12207	3099	4902	32
H16	9893	2230	4967	27
H1	8020(60)	1210(40)	7150(30)	45(15)

Table S27. Torsion angles [°] for jonap19.

Zn1-N1-C2-C1	-11.2(3)	C2-N1-C6-C12	99.0(3)
Zn1-N1-C2-C3	-132.5(2)	C3-O2-C4-N1	-42.1(3)
Zn1-N1-C2-C5	112.8(2)	C3-O2-C4-C7	74.7(3)
Zn1-N1-C4-O2	163.35(19)	C3-C2-C5-O3	-76.8(3)
Zn1-N1-C4-C7	44.7(3)	C4-O2-C3-C2	30.3(3)
Zn1-N1-C6-O3	-148.35(19)	C4-N1-C2-C1	105.6(3)
Zn1-N1-C6-C12	-31.2(3)	C4-N1-C2-C3	-15.7(3)
Zn1-N2-C7-C4	3.8(4)	C4-N1-C2-C5	-130.5(3)
Zn1-N2-C7-C8	-175.5(2)	C4-N1-C6-O3	95.1(3)
Zn1-N2-C11-C10	176.3(3)	C4-N1-C6-C12	-147.7(3)
Zn1-N3-C12-C6	4.1(4)	C4-C7-C8-C9	179.5(3)
Zn1-N3-C12-C13	-177.4(2)	C5-O3-C6-N1	40.0(3)
Zn1-N3-C16-C15	177.6(3)	C5-O3-C6-C12	-79.0(3)
O1-C1-C2-N1	42.4(3)	C5-C2-C3-O2	100.8(3)
O1-C1-C2-C3	158.4(3)	C6-O3-C5-C2	-45.0(3)
O1-C1-C2-C5	-74.0(3)	C6-N1-C2-C1	-133.2(3)
O2-C4-C7-N2	-151.3(3)	C6-N1-C2-C3	105.5(3)
O2-C4-C7-C8	28.1(4)	C6-N1-C2-C5	-9.3(3)
O3-C6-C12-N3	136.6(3)	C6-N1-C4-O2	-78.6(3)
O3-C6-C12-C13	-42.0(4)	C6-N1-C4-C7	162.8(2)
N1-C2-C3-O2	-8.4(3)	C6-C12-C13-C14	178.8(3)
N1-C2-C5-O3	33.1(3)	C7-N2-C11-C10	-1.4(5)
N1-C4-C7-N2	-35.2(4)	C7-C8-C9-C10	-1.0(5)
N1-C4-C7-C8	144.2(3)	C8-C9-C10-C11	1.9(5)
N1-C6-C12-N3	20.3(4)	C9-C10-C11-N2	-0.7(5)
N1-C6-C12-C13	-158.2(3)	C11-N2-C7-C4	-178.3(3)
N2-C7-C8-C9	-1.2(5)	C11-N2-C7-C8	2.4(5)
N3-C12-C13-C14	0.3(5)	C12-N3-C16-C15	-0.6(5)
C1-C2-C3-O2	-129.0(3)	C12-C13-C14-C15	-1.9(5)
C1-C2-C5-O3	154.4(3)	C13-C14-C15-C16	2.2(5)
C2-N1-C4-O2	35.8(3)	C14-C15-C16-N3	-1.0(5)
C2-N1-C4-C7	-82.9(3)	C16-N3-C12-C6	-177.6(3)
C2-N1-C6-O3	-18.1(3)	C16-N3-C12-C13	0.9(5)

Table S28. Hydrogen bonds and close contacts for jonap19 [Å and °].

D-H...A	d(D-H)	d(H...A)	d(D...A)	<(DHA)
O1-H1...O3#1	0.85(6)	2.18(6)	2.922(3)	145(5)

Symmetry transformations used to generate equivalent atoms:

#1 -x+3/2,y-1/2,-z+3/2

REFERENCE NUMBER: jonap21

4b

CRYSTAL STRUCTURE REPORT

$C_{18} H_{19} F_6 N_3 O_{10} S_2 Zn$

or

$[(\kappa^4\text{-L})Zn(OTf)(OH_2)][OTf]$

Report prepared for:

C. Wood, A. Panda, Prof. W. Jones

June 20, 2023

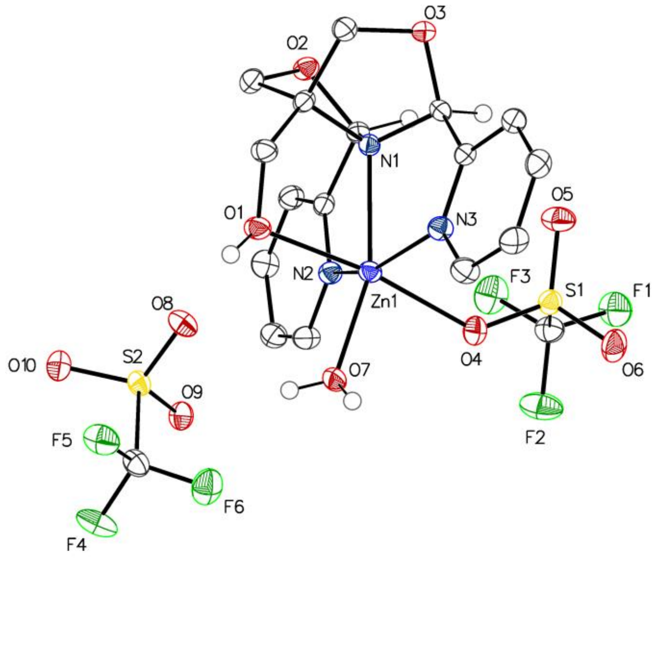

William W. Brennessel

X-ray Crystallographic Facility

Department of Chemistry, University of Rochester

120 Trustee Road

Rochester, NY 14627

Data collection

A crystal (0.108 x 0.062 x 0.038 mm^3) was placed onto a nylon loop and mounted on a Rigaku XtaLAB Synergy-S Dualflex diffractometer equipped with a HyPix-6000HE HPC area detector for data collection at 99.99(10) K. A preliminary set of cell constants and an orientation matrix were calculated from a small sampling of reflections.[1] A short pre-experiment was run, from which an optimal data collection strategy was determined. The full data collection was carried out using a PhotonJet (Cu) X-ray source with frame times of 0.13 and 0.52 seconds and a detector distance of 34.0 mm. Series of frames were collected in 0.50º steps in ω at different 2θ, κ, and ϕ settings. After the intensity data were corrected for absorption, the final cell constants were calculated from the xyz centroids of 27299 strong reflections from the actual data collection after integration.[1] See Table S29 for additional crystal and refinement information.

Structure solution and refinement

The structure was solved using SHELXT[2] and refined using SHELXL.[3] The space group *P*-1 was determined based on intensity statistics. Most or all non-hydrogen atoms were assigned from the solution. Full-matrix least squares / difference Fourier cycles were performed which located any remaining non-hydrogen atoms. All non-hydrogen atoms were refined with anisotropic displacement parameters. The O-H hydrogen atoms were found from the difference Fourier map and refined freely. All other hydrogen atoms were placed in ideal positions and refined as riding atoms with relative isotropic displacement parameters. The final full matrix least squares refinement converged to $R1 = 0.0309$ (F^2, $I > 2\sigma(I)$) and $wR2 = 0.0796$ (F^2, all data).

Structure description

The structure is the one suggested. The asymmetric unit contains one monocationic Zn complex and one OTf (triflate) anion in general positions. Each cation-anion pairing and its inverted symmetry equivalent are linked via O-H…O hydrogen bonding (see figure and Table S35).

Structure manipulation and figure generation were performed using Olex2.[4] Unless noted otherwise all structural diagrams containing anisotropic displacement ellipsoids are drawn at the 50 % probability level.

Data collection, structure solution, and structure refinement were conducted at the X-ray Crystallographic Facility, B04 Hutchison Hall, Department of Chemistry, University of Rochester. The instrument was purchased with funding from NSF MRI program grant CHE-1725028. All publications arising from this report MUST either 1) include William W. Brennessel as a coauthor or 2) acknowledge William W. Brennessel and the X-ray Crystallographic Facility of the Department of Chemistry at the University of Rochester.

[1] *CrysAlisPro*, version 171.42.90a; Rigaku Corporation: Oxford, UK, 2023.

[2] Sheldrick, G. M. *SHELXT*, version 2018/2; *Acta. Crystallogr.* **2015**, *A71*, 3-8.

[3] Sheldrick, G. M. *SHELXL*, version 2019/2; *Acta. Crystallogr.* **2015**, *C71*, 3-8.

[4] Dolomanov, O. V.; Bourhis, L. J.; Gildea, R. J.; Howard, J. A. K.; Puschmann, H. *Olex2*, version 1.5; *J. Appl. Cryst.* **2009**, *42*, 339-341.

Some equations of interest:

$$R_{int} = \Sigma |F_o^2 - \langle F_o^2 \rangle| / \Sigma |F_o^2|$$

$$R1 = \Sigma||F_o|-|F_c|| / \Sigma|F_o|$$

$$wR2 = [\Sigma[w(F_o^2-F_c^2)^2] / \Sigma [w(F_o^2)^2]]^{1/2}$$

where $w = 1 / [\sigma^2 (F_o^2) + (aP)^2 + bP]$ and

$$P = 1/3 \max (0, F_o^2) + 2/3 F_c^2$$

$$GOF = S = [\Sigma[w(F_o^2-F_c^2)^2] / (m-n)]^{1/2}$$

where m = number of reflections and n = number of parameters

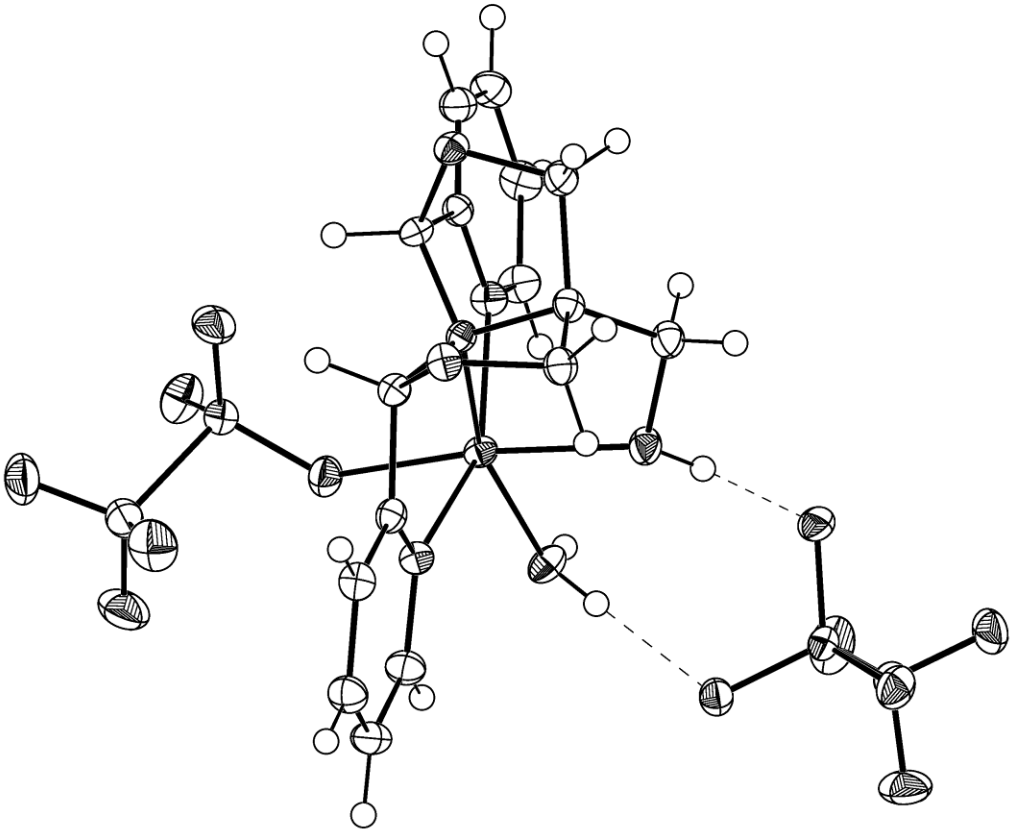

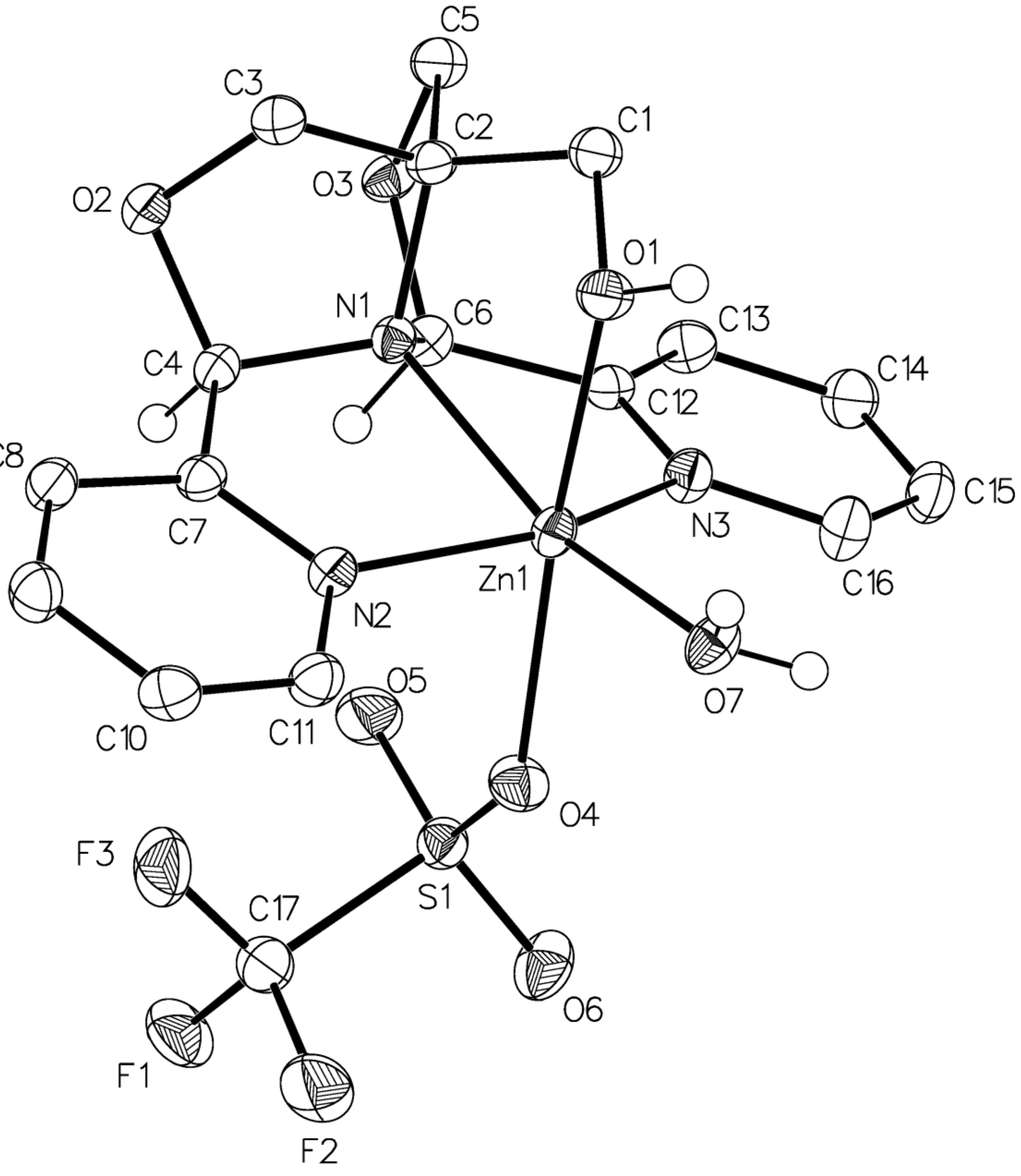
C5
C3
C2
C1
O2
O3
O1
N1
C6
C13
C4
C14
C12
C8
C7
N3
C15
Zn1
C16
C9
N2
O7
O5
C10
C11
O4
F3
C17
S1
O6
F1
F2

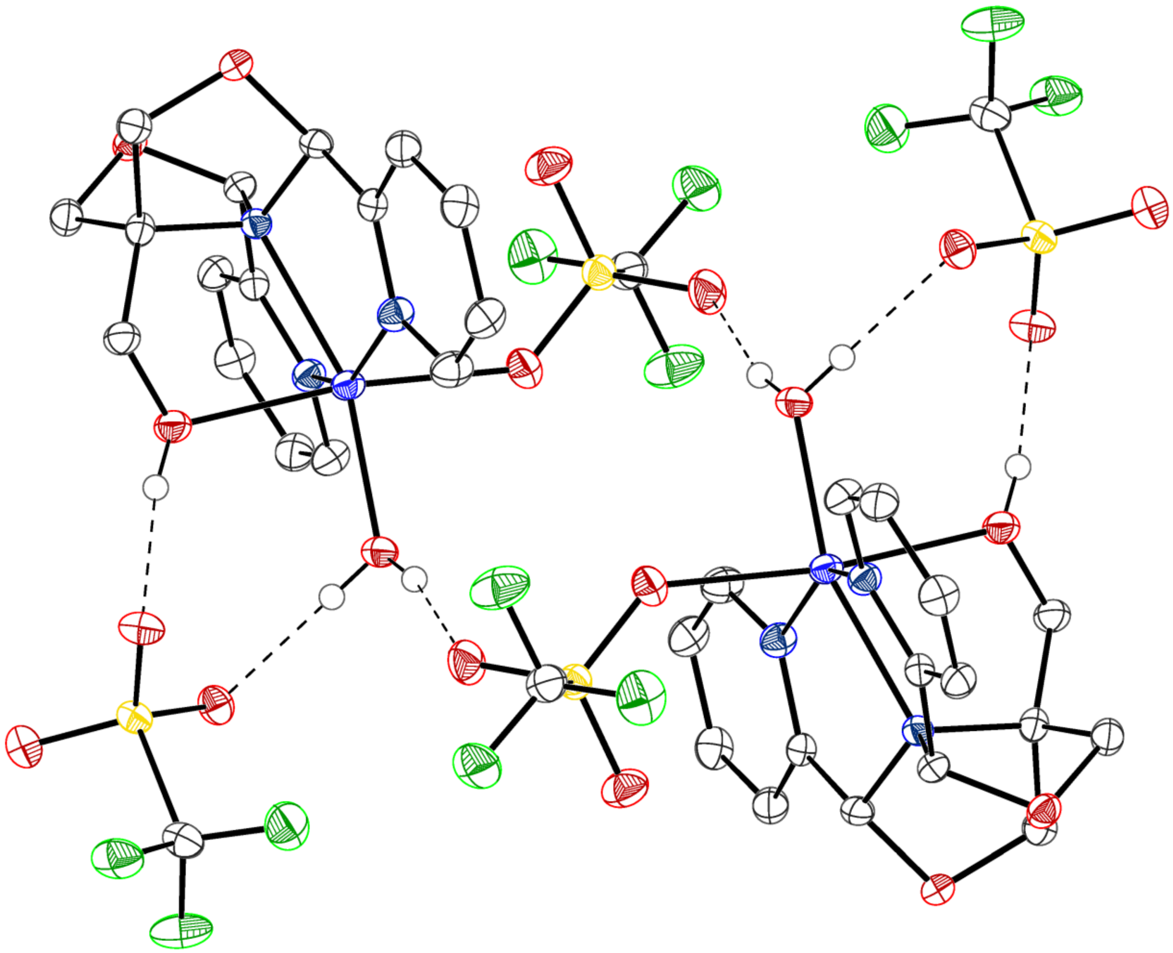

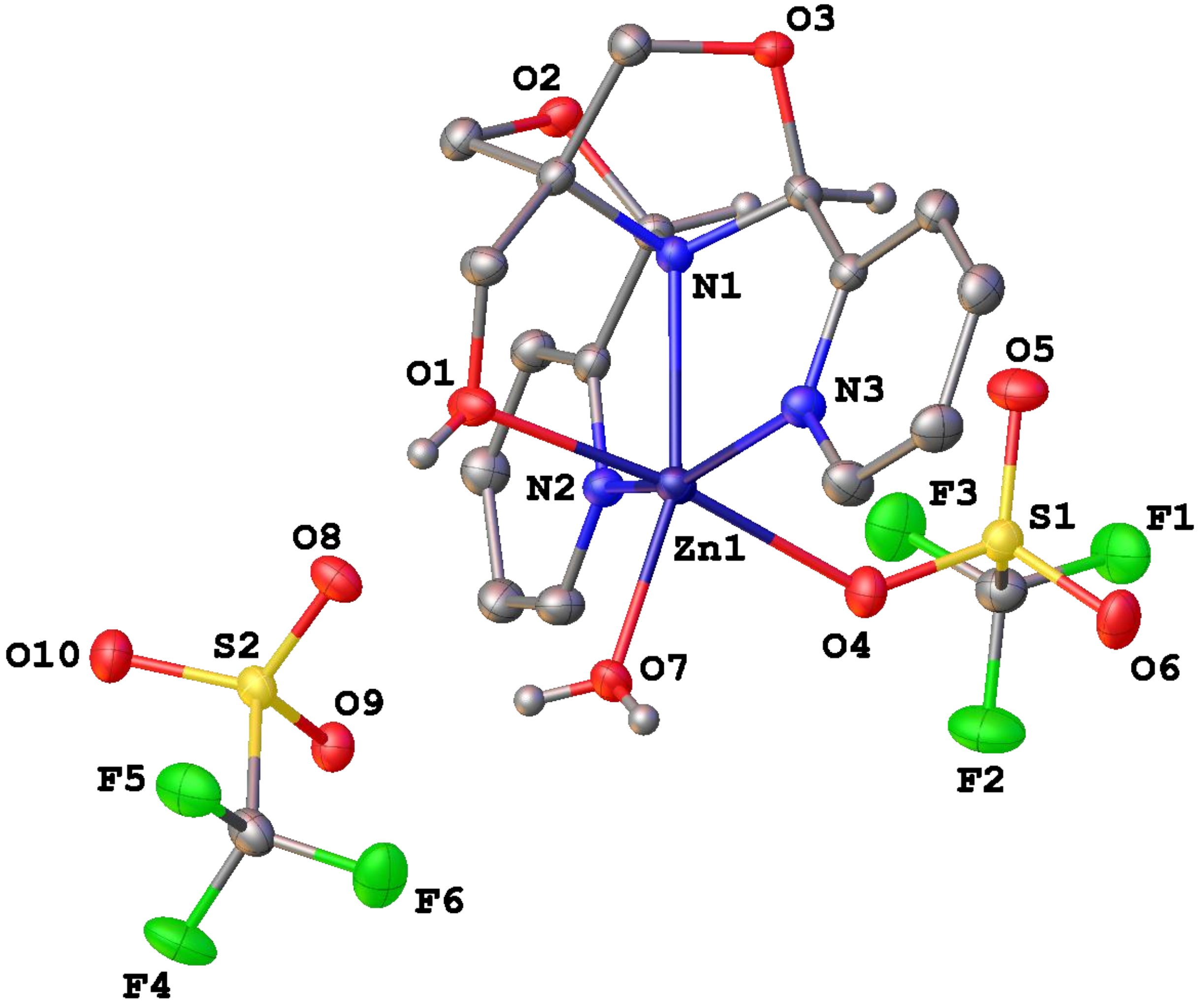
O3
O2
N1
O1
N3
O5
N2
F3
S1
F1
O8
Zn1
O10
S2
O4
O6
O7
O9
F5
F2
F6
F4

Table S29. Crystal data and structure refinement for jonap21.

Identification code	jonap21	
Empirical formula	C18 H19 F6 N3 O10 S2 Zn	
Formula weight	680.85	
Temperature	99.99(10) K	
Wavelength	1.54184 Å	
Crystal system	triclinic	
Space group	*P*-1	
Unit cell dimensions	*a* = 9.5046(4) Å	α = 71.422(3)°
	b = 11.2321(4) Å	β = 86.836(3)°
	c = 12.1135(4) Å	γ = 83.235(3)°
Volume	1217.06(8) $Å^3$	
Z	2	
Density (calculated)	1.858 Mg/m^3	
Absorption coefficient	4.011 mm^{-1}	
F(000)	688	
Crystal color, morphology	colourless, plate	
Crystal size	0.108 x 0.062 x 0.038 mm^3	
Theta range for data collection	3.850 to 80.096°	
Index ranges	$-11 \leq h \leq 12$, $-14 \leq k \leq 14$, $-15 \leq l \leq 15$	
Reflections collected	41165	
Independent reflections	5220 [*R*(int) = 0.0455]	
Observed reflections	4873	
Completeness to theta = 74.504°	99.7%	
Absorption correction	Multi-scan	
Max. and min. transmission	1.00000 and 0.88939	
Refinement method	Full-matrix least-squares on F^2	
Data / restraints / parameters	5220 / 0 / 373	
Goodness-of-fit on F^2	1.108	
Final *R* indices [*I*>2sigma(*I*)]	*R*1 = 0.0309, *wR*2 = 0.0785	
R indices (all data)	*R*1 = 0.0328, *wR*2 = 0.0796	
Largest diff. peak and hole	0.471 and -0.471 e.$Å^{-3}$	

Table S30. Atomic coordinates (x 10^4) and equivalent isotropic displacement parameters ($Å^2$x 10^3) for jonap21. U_{eq} is defined as one third of the trace of the orthogonalized U_{ij} tensor.

	x	y	z	U_{eq}
Zn1	3325(1)	2609(1)	6417(1)	15(1)
S1	4685(1)	4550(1)	7608(1)	18(1)
F1	3901(2)	5639(1)	9197(1)	34(1)
F2	2746(2)	6405(1)	7606(1)	38(1)
F3	2341(2)	4591(1)	8815(1)	35(1)
O1	2628(2)	1037(1)	5909(1)	21(1)
O2	2206(2)	-257(1)	9386(1)	18(1)
O3	5554(1)	-532(1)	8887(1)	17(1)
O4	3866(2)	4335(1)	6721(1)	22(1)
O5	5225(2)	3432(1)	8502(1)	28(1)
O6	5660(2)	5480(2)	7124(1)	28(1)
O7	2681(2)	3766(1)	4824(1)	20(1)
N1	3694(2)	899(1)	7964(1)	14(1)
N2	1492(2)	2799(2)	7359(1)	16(1)
N3	5485(2)	2128(2)	6298(1)	18(1)
C1	3464(2)	-132(2)	6434(2)	20(1)
C2	3529(2)	-322(2)	7735(2)	16(1)
C3	2157(2)	-697(2)	8401(2)	19(1)
C4	2635(2)	975(2)	8886(2)	15(1)
C5	4848(2)	-1212(2)	8297(2)	19(1)
C6	5174(2)	768(2)	8312(2)	15(1)
C7	1349(2)	1921(2)	8396(2)	16(1)
C8	112(2)	1894(2)	9054(2)	20(1)
C9	-1013(2)	2806(2)	8633(2)	22(1)
C10	-854(2)	3737(2)	7575(2)	22(1)
C11	408(2)	3703(2)	6962(2)	21(1)
C12	6114(2)	1274(2)	7238(2)	15(1)
C13	7554(2)	910(2)	7256(2)	20(1)
C14	8383(2)	1440(2)	6285(2)	22(1)
C15	7742(2)	2336(2)	5325(2)	23(1)
C16	6291(2)	2655(2)	5363(2)	22(1)

C17	3344(2)	5340(2)	8350(2)	23(1)
S2	960(1)	2200(1)	3200(1)	17(1)
F4	172(2)	3729(1)	1153(1)	35(1)
F5	1750(2)	2209(1)	1098(1)	31(1)
F6	2335(2)	3782(1)	1550(1)	35(1)
O8	2314(2)	1489(1)	3603(1)	22(1)
O9	573(2)	3209(1)	3705(1)	20(1)
O10	-160(2)	1461(1)	3172(1)	26(1)
C18	1322(2)	3035(2)	1665(2)	22(1)

Table S31. Bond lengths [Å] and angles [°] for jonap21.

Zn(1)-O(1)	2.2247(14)	C(3)-H(3B)	0.9900
Zn(1)-O(4)	2.2064(14)	C(4)-H(4)	1.0000
Zn(1)-O(7)	2.0327(14)	C(4)-C(7)	1.532(3)
Zn(1)-N(1)	2.2233(15)	C(5)-H(5A)	0.9900
Zn(1)-N(2)	2.0575(16)	C(5)-H(5B)	0.9900
Zn(1)-N(3)	2.0695(17)	C(6)-H(6)	1.0000
S(1)-O(4)	1.4571(15)	C(6)-C(12)	1.528(3)
S(1)-O(5)	1.4334(15)	C(7)-C(8)	1.381(3)
S(1)-O(6)	1.4393(15)	C(8)-H(8)	0.9500
S(1)-C(17)	1.827(2)	C(8)-C(9)	1.386(3)
F(1)-C(17)	1.329(3)	C(9)-H(9)	0.9500
F(2)-C(17)	1.332(2)	C(9)-C(10)	1.385(3)
F(3)-C(17)	1.326(3)	C(10)-H(10)	0.9500
O(1)-H(1)	0.83(3)	C(10)-C(11)	1.379(3)
O(1)-C(1)	1.427(2)	C(11)-H(11)	0.9500
O(2)-C(3)	1.434(2)	C(12)-C(13)	1.380(3)
O(2)-C(4)	1.421(2)	C(13)-H(13)	0.9500
O(3)-C(5)	1.437(2)	C(13)-C(14)	1.387(3)
O(3)-C(6)	1.416(2)	C(14)-H(14)	0.9500
O(7)-H(7A)	0.84(4)	C(14)-C(15)	1.387(3)
O(7)-H(7B)	0.80(3)	C(15)-H(15)	0.9500
N(1)-C(2)	1.510(2)	C(15)-C(16)	1.386(3)
N(1)-C(4)	1.479(2)	C(16)-H(16)	0.9500
N(1)-C(6)	1.468(2)	S(2)-O(8)	1.4531(15)
N(2)-C(7)	1.339(2)	S(2)-O(9)	1.4543(14)
N(2)-C(11)	1.349(3)	S(2)-O(10)	1.4327(15)
N(3)-C(12)	1.348(2)	S(2)-C(18)	1.830(2)
N(3)-C(16)	1.343(3)	F(4)-C(18)	1.324(2)
C(1)-H(1A)	0.9900	F(5)-C(18)	1.336(2)
C(1)-H(1B)	0.9900	F(6)-C(18)	1.323(3)
C(1)-C(2)	1.525(3)	O(4)-Zn(1)-O(1)	172.52(5)
C(2)-C(3)	1.525(3)	O(4)-Zn(1)-N(1)	112.75(6)
C(2)-C(5)	1.550(3)	O(7)-Zn(1)-O(1)	86.87(6)
C(3)-H(3A)	0.9900	O(7)-Zn(1)-O(4)	85.83(6)

O(7)-Zn(1)-N(1)	161.18(6)	C(16)-N(3)-Zn(1)	123.72(14)
O(7)-Zn(1)-N(2)	98.97(6)	C(16)-N(3)-C(12)	118.81(17)
O(7)-Zn(1)-N(3)	107.57(7)	O(1)-C(1)-H(1A)	110.1
N(1)-Zn(1)-O(1)	74.46(5)	O(1)-C(1)-H(1B)	110.1
N(2)-Zn(1)-O(1)	93.07(6)	O(1)-C(1)-C(2)	107.98(16)
N(2)-Zn(1)-O(4)	86.46(6)	H(1A)-C(1)-H(1B)	108.4
N(2)-Zn(1)-N(1)	80.04(6)	C(2)-C(1)-H(1A)	110.1
N(2)-Zn(1)-N(3)	151.93(6)	C(2)-C(1)-H(1B)	110.1
N(3)-Zn(1)-O(1)	97.25(6)	N(1)-C(2)-C(1)	111.49(15)
N(3)-Zn(1)-O(4)	86.53(6)	N(1)-C(2)-C(3)	101.91(15)
N(3)-Zn(1)-N(1)	77.74(6)	N(1)-C(2)-C(5)	104.01(14)
O(4)-S(1)-C(17)	102.32(9)	C(1)-C(2)-C(3)	113.69(16)
O(5)-S(1)-O(4)	115.21(9)	C(1)-C(2)-C(5)	112.08(16)
O(5)-S(1)-O(6)	116.21(10)	C(3)-C(2)-C(5)	112.77(16)
O(5)-S(1)-C(17)	104.56(10)	O(2)-C(3)-C(2)	103.19(15)
O(6)-S(1)-O(4)	112.67(9)	O(2)-C(3)-H(3A)	111.1
O(6)-S(1)-C(17)	103.67(10)	O(2)-C(3)-H(3B)	111.1
Zn(1)-O(1)-H(1)	122(2)	C(2)-C(3)-H(3A)	111.1
C(1)-O(1)-Zn(1)	111.93(11)	C(2)-C(3)-H(3B)	111.1
C(1)-O(1)-H(1)	108(2)	H(3A)-C(3)-H(3B)	109.1
C(4)-O(2)-C(3)	103.25(14)	O(2)-C(4)-N(1)	106.42(14)
C(6)-O(3)-C(5)	106.59(14)	O(2)-C(4)-H(4)	109.5
S(1)-O(4)-Zn(1)	133.07(9)	O(2)-C(4)-C(7)	110.02(15)
Zn(1)-O(7)-H(7A)	121(2)	N(1)-C(4)-H(4)	109.5
Zn(1)-O(7)-H(7B)	121(2)	N(1)-C(4)-C(7)	111.76(15)
H(7A)-O(7)-H(7B)	103(3)	C(7)-C(4)-H(4)	109.5
C(2)-N(1)-Zn(1)	114.04(11)	O(3)-C(5)-C(2)	105.93(15)
C(4)-N(1)-Zn(1)	109.41(11)	O(3)-C(5)-H(5A)	110.6
C(4)-N(1)-C(2)	105.51(14)	O(3)-C(5)-H(5B)	110.6
C(6)-N(1)-Zn(1)	108.77(11)	C(2)-C(5)-H(5A)	110.6
C(6)-N(1)-C(2)	104.56(14)	C(2)-C(5)-H(5B)	110.6
C(6)-N(1)-C(4)	114.60(14)	H(5A)-C(5)-H(5B)	108.7
C(7)-N(2)-Zn(1)	117.15(13)	O(3)-C(6)-N(1)	106.83(14)
C(7)-N(2)-C(11)	118.89(17)	O(3)-C(6)-H(6)	109.4
C(11)-N(2)-Zn(1)	123.83(13)	O(3)-C(6)-C(12)	111.81(15)
C(12)-N(3)-Zn(1)	117.41(13)	N(1)-C(6)-H(6)	109.4

N(1)-C(6)-C(12)	109.81(14)	C(15)-C(14)-H(14)	120.5
C(12)-C(6)-H(6)	109.4	C(14)-C(15)-H(15)	120.6
N(2)-C(7)-C(4)	118.24(17)	C(16)-C(15)-C(14)	118.81(19)
N(2)-C(7)-C(8)	121.92(18)	C(16)-C(15)-H(15)	120.6
C(8)-C(7)-C(4)	119.74(17)	N(3)-C(16)-C(15)	122.26(19)
C(7)-C(8)-H(8)	120.4	N(3)-C(16)-H(16)	118.9
C(7)-C(8)-C(9)	119.15(19)	C(15)-C(16)-H(16)	118.9
C(9)-C(8)-H(8)	120.4	F(1)-C(17)-S(1)	111.48(15)
C(8)-C(9)-H(9)	120.5	F(1)-C(17)-F(2)	107.61(17)
C(10)-C(9)-C(8)	119.01(19)	F(2)-C(17)-S(1)	110.71(15)
C(10)-C(9)-H(9)	120.5	F(3)-C(17)-S(1)	110.11(15)
C(9)-C(10)-H(10)	120.6	F(3)-C(17)-F(1)	108.20(18)
C(11)-C(10)-C(9)	118.76(19)	F(3)-C(17)-F(2)	108.63(18)
C(11)-C(10)-H(10)	120.6	O(8)-S(2)-O(9)	112.77(9)
N(2)-C(11)-C(10)	122.22(19)	O(8)-S(2)-C(18)	103.24(9)
N(2)-C(11)-H(11)	118.9	O(9)-S(2)-C(18)	103.78(9)
C(10)-C(11)-H(11)	118.9	O(10)-S(2)-O(8)	115.68(9)
N(3)-C(12)-C(6)	117.01(16)	O(10)-S(2)-O(9)	115.15(9)
N(3)-C(12)-C(13)	121.92(18)	O(10)-S(2)-C(18)	104.21(9)
C(13)-C(12)-C(6)	121.00(17)	F(4)-C(18)-S(2)	111.13(15)
C(12)-C(13)-H(13)	120.4	F(4)-C(18)-F(5)	107.93(17)
C(12)-C(13)-C(14)	119.28(19)	F(5)-C(18)-S(2)	110.16(14)
C(14)-C(13)-H(13)	120.4	F(6)-C(18)-S(2)	111.27(14)
C(13)-C(14)-H(14)	120.5	F(6)-C(18)-F(4)	108.52(18)
C(15)-C(14)-C(13)	118.91(19)	F(6)-C(18)-F(5)	107.70(18)

Table S32. Anisotropic displacement parameters ($Å^2$x 10^3) for jonap21. The anisotropic displacement factor exponent takes the form: $-2\pi^2[h^2 a^{*2}U_{11} + ... + 2 h k a^* b^* U_{12}]$

	U_{11}	U_{22}	U_{33}	U_{23}	U_{13}	U_{12}
Zn1	16(1)	16(1)	13(1)	-2(1)	-1(1)	-2(1)
S1	18(1)	18(1)	17(1)	-5(1)	0(1)	-3(1)
F1	47(1)	34(1)	26(1)	-18(1)	-4(1)	-1(1)
F2	45(1)	30(1)	32(1)	-7(1)	-4(1)	15(1)
F3	29(1)	44(1)	34(1)	-15(1)	13(1)	-10(1)
O1	30(1)	17(1)	16(1)	-5(1)	-5(1)	0(1)
O2	22(1)	16(1)	15(1)	-3(1)	2(1)	-4(1)
O3	20(1)	14(1)	16(1)	-2(1)	-3(1)	-2(1)
O4	24(1)	23(1)	24(1)	-10(1)	-2(1)	-5(1)
O5	34(1)	21(1)	25(1)	-5(1)	-4(1)	5(1)
O6	27(1)	34(1)	24(1)	-8(1)	3(1)	-16(1)
O7	22(1)	22(1)	15(1)	-2(1)	-4(1)	-6(1)
N1	15(1)	15(1)	12(1)	-4(1)	0(1)	-2(1)
N2	16(1)	16(1)	15(1)	-3(1)	0(1)	-2(1)
N3	17(1)	19(1)	16(1)	-4(1)	2(1)	-2(1)
C1	26(1)	17(1)	16(1)	-5(1)	-2(1)	-1(1)
C2	18(1)	14(1)	17(1)	-5(1)	-1(1)	-3(1)
C3	21(1)	16(1)	20(1)	-6(1)	1(1)	-4(1)
C4	16(1)	14(1)	14(1)	-2(1)	0(1)	-3(1)
C5	21(1)	16(1)	19(1)	-5(1)	-2(1)	-2(1)
C6	14(1)	16(1)	15(1)	-3(1)	-3(1)	-1(1)
C7	18(1)	15(1)	15(1)	-5(1)	-1(1)	-4(1)
C8	21(1)	19(1)	18(1)	-4(1)	1(1)	-4(1)
C9	19(1)	24(1)	23(1)	-7(1)	3(1)	-2(1)
C10	18(1)	20(1)	27(1)	-6(1)	-3(1)	1(1)
C11	21(1)	18(1)	20(1)	-3(1)	-1(1)	0(1)
C12	17(1)	13(1)	16(1)	-5(1)	-1(1)	-2(1)
C13	19(1)	18(1)	22(1)	-5(1)	-2(1)	-2(1)
C14	17(1)	24(1)	26(1)	-9(1)	3(1)	-3(1)
C15	21(1)	25(1)	20(1)	-5(1)	6(1)	-4(1)
C16	21(1)	26(1)	18(1)	-3(1)	2(1)	-3(1)

C17	27(1)	21(1)	21(1)	-6(1)	-1(1)	-1(1)
S2	18(1)	20(1)	16(1)	-7(1)	-2(1)	-2(1)
F4	32(1)	40(1)	24(1)	-3(1)	-7(1)	14(1)
F5	42(1)	32(1)	20(1)	-13(1)	-3(1)	6(1)
F6	40(1)	42(1)	24(1)	-10(1)	6(1)	-19(1)
O8	22(1)	27(1)	17(1)	-7(1)	-4(1)	3(1)
O9	19(1)	22(1)	21(1)	-10(1)	1(1)	-3(1)
O10	26(1)	25(1)	29(1)	-9(1)	-4(1)	-8(1)
C18	22(1)	24(1)	21(1)	-9(1)	-4(1)	1(1)

Table S33. Hydrogen coordinates (x 10^4) and isotropic displacement parameters (Å^2 x 10^3) for jonap21.

	x	y	z	U(eq)
H1	2510(30)	1100(30)	5220(30)	40(8)
H7A	3270(40)	4040(30)	4280(30)	55(10)
H7B	2060(30)	3600(30)	4500(30)	31(8)
H1A	4431	-111	6085	24
H1B	3032	-836	6306	24
H3A	1320	-281	7921	23
H3B	2131	-1624	8650	23
H4	3083	1227	9494	18
H5A	4562	-2006	8856	23
H5B	5481	-1421	7693	23
H6	5250	1256	8863	18
H8	33	1259	9785	23
H9	-1881	2793	9063	26
H10	-1599	4386	7278	26
H11	518	4338	6234	25
H13	7971	302	7927	24
H14	9374	1194	6277	27
H15	8289	2725	4653	27
H16	5851	3267	4705	27

Table S34. Torsion angles [°] for jonap21.

Zn1-O1-C1-C2	49.98(18)	N1-C4-C7-N2	-19.7(2)
Zn1-N1-C2-C1	12.51(19)	N1-C4-C7-C8	163.84(17)
Zn1-N1-C2-C3	-109.11(13)	N1-C6-C12-N3	23.8(2)
Zn1-N1-C2-C5	133.48(12)	N1-C6-C12-C13	-159.07(17)
Zn1-N1-C4-O2	139.12(11)	N2-C7-C8-C9	-0.4(3)
Zn1-N1-C4-C7	19.00(17)	N3-C12-C13-C14	0.4(3)
Zn1-N1-C6-O3	-153.60(11)	C1-C2-C3-O2	-154.11(15)
Zn1-N1-C6-C12	-32.16(17)	C1-C2-C5-O3	126.55(16)
Zn1-N2-C7-C4	9.5(2)	C2-N1-C4-O2	16.02(18)
Zn1-N2-C7-C8	-174.19(14)	C2-N1-C4-C7	-104.10(16)
Zn1-N2-C11-C10	174.27(15)	C2-N1-C6-O3	-31.40(18)
Zn1-N3-C12-C6	-1.5(2)	C2-N1-C6-C12	90.04(16)
Zn1-N3-C12-C13	-178.60(14)	C3-O2-C4-N1	-38.37(18)
Zn1-N3-C16-C15	178.16(16)	C3-O2-C4-C7	82.88(17)
O1-C1-C2-N1	-40.1(2)	C3-C2-C5-O3	-103.62(17)
O1-C1-C2-C3	74.4(2)	C4-O2-C3-C2	45.09(17)
O1-C1-C2-C5	-156.26(16)	C4-N1-C2-C1	132.61(16)
O2-C4-C7-N2	-137.71(17)	C4-N1-C2-C3	10.98(17)
O2-C4-C7-C8	45.9(2)	C4-N1-C2-C5	-106.42(16)
O3-C6-C12-N3	142.23(16)	C4-N1-C6-O3	83.61(18)
O3-C6-C12-C13	-40.7(2)	C4-N1-C6-C12	-154.96(15)
O4-S1-C17-F1	-179.00(14)	C4-C7-C8-C9	175.88(18)
O4-S1-C17-F2	-59.24(17)	C5-O3-C6-N1	36.19(18)
O4-S1-C17-F3	60.90(16)	C5-O3-C6-C12	-83.96(18)
O5-S1-O4-Zn1	-2.53(16)	C5-C2-C3-O2	76.88(18)
O5-S1-C17-F1	60.53(17)	C6-O3-C5-C2	-25.57(19)
O5-S1-C17-F2	-179.72(15)	C6-N1-C2-C1	-106.17(17)
O5-S1-C17-F3	-59.57(17)	C6-N1-C2-C3	132.21(15)
O6-S1-O4-Zn1	133.99(12)	C6-N1-C2-C5	14.80(18)
O6-S1-C17-F1	-61.67(16)	C6-N1-C4-O2	-98.43(17)
O6-S1-C17-F2	58.09(17)	C6-N1-C4-C7	141.44(15)
O6-S1-C17-F3	178.23(14)	C6-C12-C13-C14	-176.59(18)
N1-C2-C3-O2	-34.02(17)	C7-N2-C11-C10	-1.5(3)
N1-C2-C5-O3	5.98(19)	C7-C8-C9-C10	-1.4(3)

C8-C9-C10-C11	1.8(3)	C17-S1-O4-Zn1	-115.32(13)
C9-C10-C11-N2	-0.3(3)	O8-S2-C18-F4	179.24(14)
C11-N2-C7-C4	-174.46(17)	O8-S2-C18-F5	59.67(16)
C11-N2-C7-C8	1.9(3)	O8-S2-C18-F6	-59.70(16)
C12-N3-C16-C15	1.0(3)	O9-S2-C18-F4	-62.91(16)
C12-C13-C14-C15	0.8(3)	O9-S2-C18-F5	177.52(14)
C13-C14-C15-C16	-1.0(3)	O9-S2-C18-F6	58.15(16)
C14-C15-C16-N3	0.1(3)	O10-S2-C18-F4	57.99(17)
C16-N3-C12-C6	175.82(17)	O10-S2-C18-F5	-61.58(16)
C16-N3-C12-C13	-1.2(3)	O10-S2-C18-F6	179.05(14)

Table S35. Hydrogen bonds and close contacts for jonap21 [Å and °].

D-H...A	d(D-H)	d(H...A)	d(D...A)	<(DHA)
O1-H1...O8	0.83(3)	1.88(3)	2.702(2)	172(3)
O7-H7A...O6#1	0.84(4)	1.89(4)	2.720(2)	168(4)
O7-H7B...O9	0.80(3)	1.92(3)	2.716(2)	179(3)

Symmetry transformations used to generate equivalent atoms:

#1 -x+1,-y+1,-z+1

REFERENCE NUMBER: jonap13

5a

CRYSTAL STRUCTURE REPORT

$C_{18} H_{21} Br_2 N_3 O_3 Zn$

or

$(\kappa^3\text{-}L^{Me})ZnBr_2$

Report prepared for:

A. Panda, Prof. W. Jones

April 05, 2023

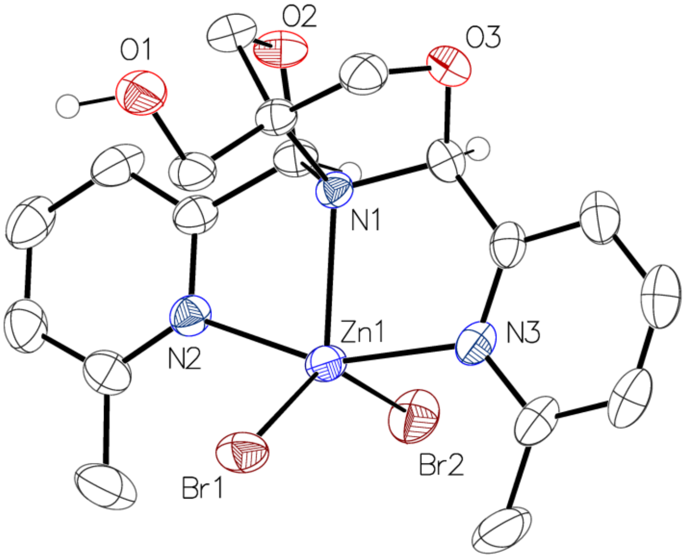

William W. Brennessel

X-ray Crystallographic Facility

Department of Chemistry, University of Rochester

120 Trustee Road

Rochester, NY 14627

Data collection

A crystal (0.284 x 0.273 x 0.16 mm^3) was placed onto a thin glass optical fiber or a nylon loop and mounted on a Rigaku XtaLAB Synergy-S Dualflex diffractometer equipped with a HyPix-6000HE HPC area detector for data collection at 173.00(10) K. A preliminary set of cell constants and an orientation matrix were calculated from a small sampling of reflections.[1] A short pre-experiment was run, from which an optimal data collection strategy was determined. The full data collection was carried out using a PhotonJet (Cu) X-ray source with a frame time of 0.05 seconds and a detector distance of 34.0 mm. Series of frames were collected in 0.50º steps in ω at different 2θ, κ, and ϕ settings. After the intensity data were corrected for absorption, the final cell constants were calculated from the xyz centroids of 44621 strong reflections from the actual data collection after integration.[1] See Table S36 for additional crystal and refinement information.

Structure solution and refinement

The structure was solved using SHELXT[2] and refined using SHELXL.[3] The space group *P*-1 was determined based on intensity statistics. Most or all non-hydrogen atoms were assigned from the solution. Full-matrix least squares / difference Fourier cycles were performed which located any remaining non-hydrogen atoms. All non-hydrogen atoms were refined with anisotropic displacement parameters. Hydroxyl group hydrogen atoms O1-H1 and O4-H4 were found from the difference Fourier map and refined freely. All other hydrogen atoms were placed in ideal positions and refined as riding atoms with relative isotropic displacement parameters. The final full matrix least squares refinement converged to $R1 = 0.0414$ (F^2, $I > 2\sigma(I)$) and $wR2 = 0.1044$ (F^2, all data).

Structure description

The structure is the one suggested. The asymmetric unit contains two molecules in general positions. Hydroxyl group O4-H4 is modeled as disordered over two positions (0.61:0.39). Intra- and intermolecular O-H…Br hydrogen bonding is present (see Table S42).

Structure manipulation and figure generation were performed using Olex2.[4] Unless noted otherwise all structural diagrams containing anisotropic displacement ellipsoids are drawn at the 50 % probability level.

Data collection, structure solution, and structure refinement were conducted at the X-ray Crystallographic Facility, B04 Hutchison Hall, Department of Chemistry, University of Rochester. The instrument was purchased with funding from NSF MRI program grant CHE-1725028. All publications arising from this report MUST either 1) include William W. Brennessel as a coauthor or 2) acknowledge William W. Brennessel and the X-ray Crystallographic Facility of the Department of Chemistry at the University of Rochester.

[1] *CrysAlisPro*, version 171.42.85a; Rigaku Corporation: Oxford, UK, 2023.

[2] Sheldrick, G. M. *SHELXT*, version 2018/2; *Acta. Crystallogr.* **2015**, *A71*, 3-8.

[3] Sheldrick, G. M. *SHELXL*, version 2019/2; *Acta. Crystallogr.* **2015**, *C71*, 3-8.

[4] Dolomanov, O. V.; Bourhis, L. J.; Gildea, R. J.; Howard, J. A. K.; Puschmann, H. *Olex2*, version 1.5; *J. Appl. Cryst.* **2009**, *42*, 339-341.

Some equations of interest:

$$R_{int} = \Sigma\,|F_o^2 - \langle F_o^2 \rangle| \,/\, \Sigma|F_o^2|$$

$$R1 = \Sigma||F_o|-|F_c|| \,/\, \Sigma|F_o|$$

$$wR2 = [\Sigma[w(F_o^2-F_c^2)^2] \,/\, \Sigma\,[w(F_o^2)^2]]^{1/2}$$

where $w = 1 / [\sigma^2 (F_o^2) + (aP)^2 + bP]$ and

$$P = 1/3 \max(0, F_o^2) + 2/3\, F_c^2$$

$$\mathrm{GOF} = S = [\Sigma[w(F_o^2-F_c^2)^2] \,/\, (m-n)]^{1/2}$$

where m = number of reflections and n = number of parameters

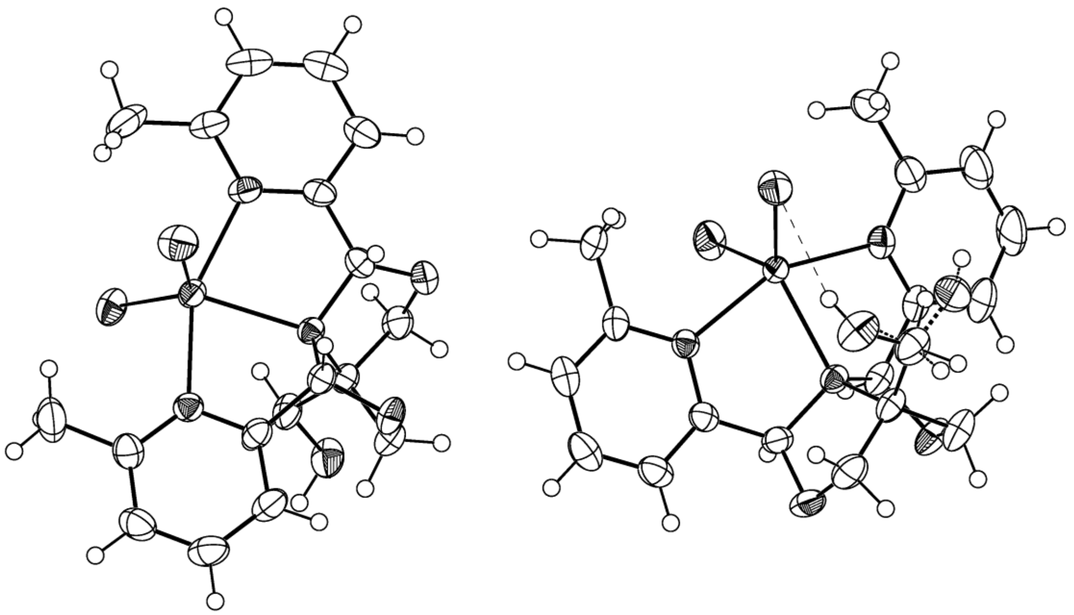

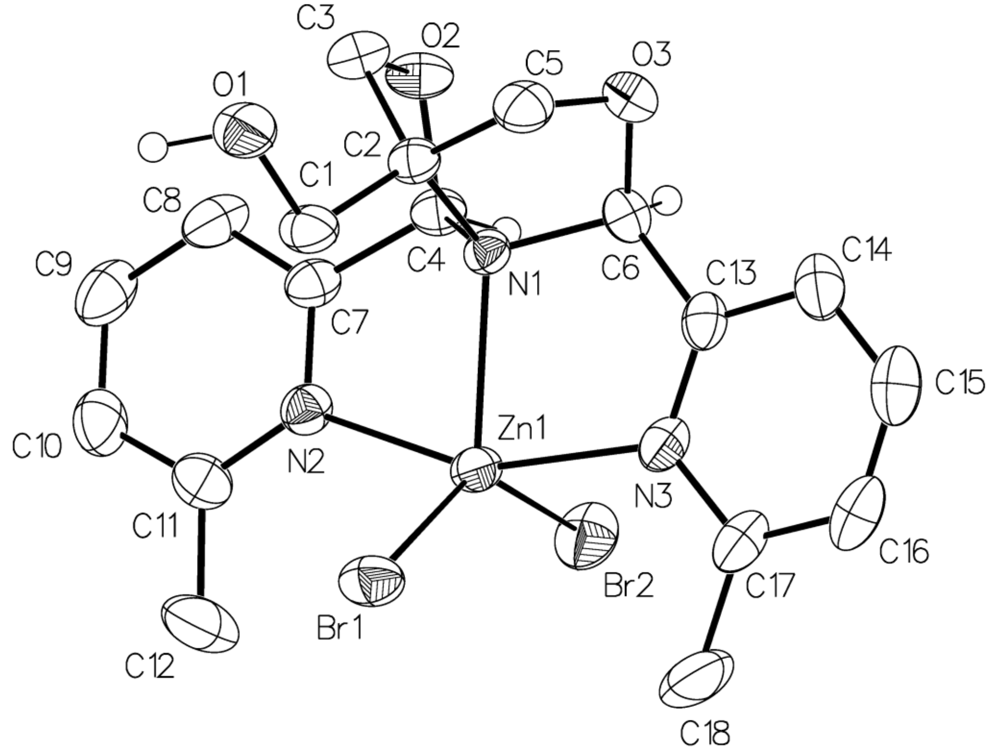
C3
O2
C5
O3
O1
C2
C1
C8
C9
C4
N1
C6
C13
C14
C7
C15
Zn1
C10
N2
N3
C11
C16
Br2
C17
Br1
C12
C18

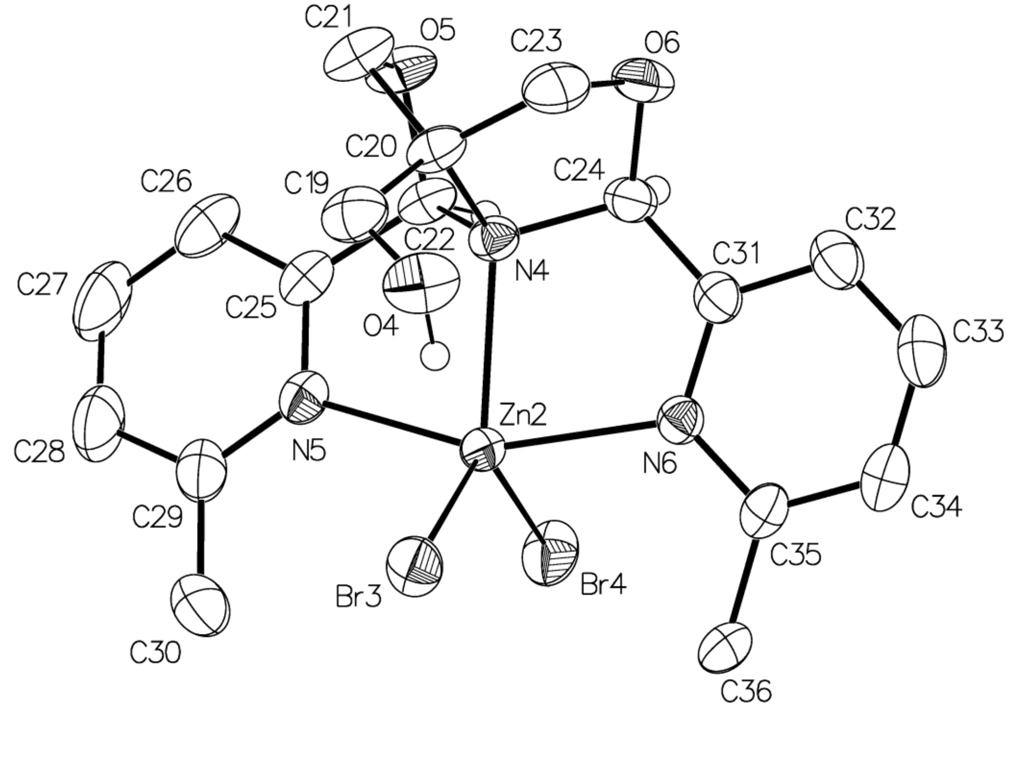
C21
O5
C23
O6
C20
C26
C19
C24
C22
C32
C27
C25
N4
C31
O4
C33
Zn2
C28
N5
N6
C29
C34
C35
Br3
Br4
C30
C36

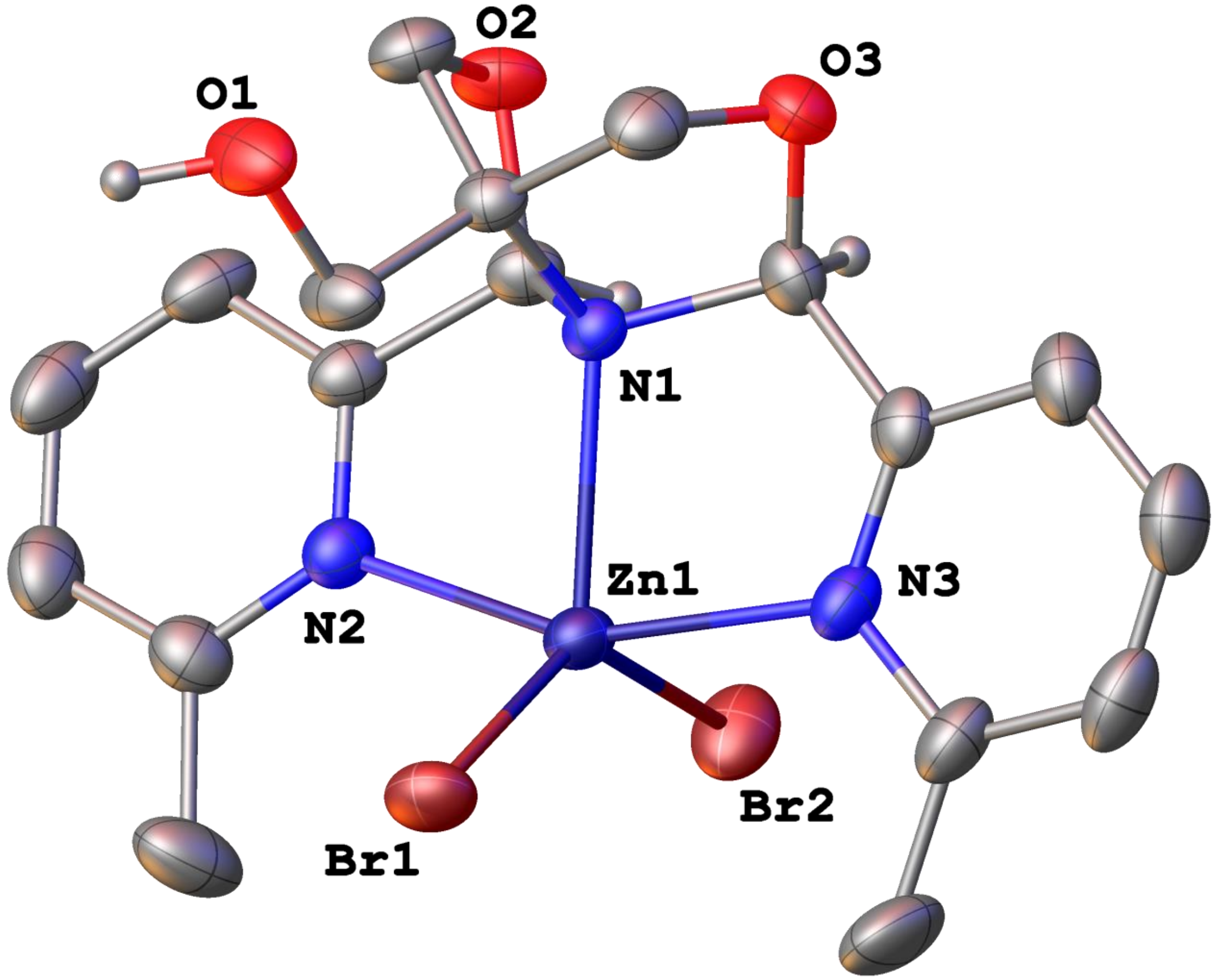
O2
O3
O1
N1
Zn1
N3
N2
Br2
Br1

Table S36. Crystal data and structure refinement for jonap13.

Identification code	jonap13	
Empirical formula	C18 H21 Br2 N3 O3 Zn	
Formula weight	552.57	
Temperature	173.00(10) K	
Wavelength	1.54184 Å	
Crystal system	triclinic	
Space group	*P*-1	
Unit cell dimensions	*a* = 9.49240(10) Å	α = 76.7440(10)°
	b = 14.09470(10) Å	β = 88.0030(10)°
	c = 16.3066(2) Å	γ = 70.5770(10)°
Volume	2000.68(4) $Å^3$	
Z	4	
Density (calculated)	1.834 Mg/m^3	
Absorption coefficient	6.588 mm^{-1}	
F(000)	1096	
Crystal color, morphology	colourless, block	
Crystal size	0.284 x 0.273 x 0.16 mm^3	
Theta range for data collection	3.417 to 80.009°	
Index ranges	$-11 \le h \le 12$, $-17 \le k \le 18$, $-20 \le l \le 19$	
Reflections collected	65999	
Independent reflections	8557 [*R*(int) = 0.0685]	
Observed reflections	8189	
Completeness to theta = 74.504°	99.7%	
Absorption correction	Multi-scan	
Max. and min. transmission	1.00000 and 0.65969	
Refinement method	Full-matrix least-squares on F^2	
Data / restraints / parameters	8557 / 0 / 510	
Goodness-of-fit on F^2	1.052	
Final *R* indices [*I*>2sigma(*I*)]	*R*1 = 0.0414, *wR*2 = 0.1031	
R indices (all data)	*R*1 = 0.0429, *wR*2 = 0.1044	
Extinction coefficient	0.00240(9)	
Largest diff. peak and hole	2.344 and -1.456 e.$Å^{-3}$	

Table S37. Atomic coordinates (x 10^4) and equivalent isotropic displacement parameters ($Å^2$x 10^3) for jonap13. U_{eq} is defined as one third of the trace of the orthogonalized U_{ij} tensor.

	x	y	z	U_{eq}
Br1	40(1)	5156(1)	7900(1)	38(1)
Br2	1400(1)	7564(1)	8741(1)	47(1)
Zn1	1442(1)	6296(1)	7924(1)	28(1)
O1	4157(4)	3924(2)	6118(2)	45(1)
O2	5738(3)	5995(2)	6984(2)	35(1)
O3	3302(3)	7183(2)	5571(2)	39(1)
N1	3186(3)	6300(2)	6967(2)	23(1)
N2	3450(3)	5209(2)	8641(2)	29(1)
N3	184(3)	7542(2)	6905(2)	31(1)
C1	3375(4)	4587(3)	6649(2)	37(1)
C2	3849(3)	5534(2)	6416(2)	26(1)
C3	5554(4)	5270(3)	6545(2)	34(1)
C4	4494(4)	6182(3)	7498(2)	28(1)
C5	3289(4)	6188(3)	5534(2)	36(1)
C6	2682(4)	7325(2)	6352(2)	30(1)
C7	4708(4)	5304(3)	8276(2)	30(1)
C8	6122(4)	4680(3)	8593(2)	40(1)
C9	6243(5)	3928(3)	9322(3)	47(1)
C10	4973(5)	3845(3)	9718(2)	44(1)
C11	3568(4)	4500(3)	9371(2)	37(1)
C12	2169(5)	4450(4)	9807(3)	57(1)
C13	982(4)	7800(2)	6256(2)	30(1)
C14	339(5)	8549(3)	5531(2)	39(1)
C15	-1188(5)	9054(3)	5492(3)	47(1)
C16	-2001(4)	8818(3)	6174(3)	46(1)
C17	-1307(4)	8055(3)	6880(3)	40(1)
C18	-2174(5)	7781(4)	7619(3)	61(1)
Br3	6506(1)	10479(1)	3189(1)	39(1)
Br4	5043(1)	8257(1)	2355(1)	43(1)
Zn2	7017(1)	8897(1)	2716(1)	24(1)
O4	10038(6)	9545(4)	3423(3)	48(2)

O4'	9916(9)	9934(6)	1962(6)	59(3)
O5	10837(3)	7288(2)	1572(2)	41(1)
O6	11203(3)	6527(2)	3491(2)	42(1)
N4	9343(3)	7895(2)	2618(2)	26(1)
N5	7348(3)	9367(2)	1379(2)	31(1)
N6	7439(3)	7838(2)	3957(2)	26(1)
C19	10493(4)	9307(3)	2671(3)	42(1)
C20	10761(4)	8186(3)	2648(2)	32(1)
C21	11544(4)	7918(4)	1847(3)	44(1)
C22	9327(4)	7687(3)	1768(2)	32(1)
C23	11585(4)	7432(3)	3451(3)	42(1)
C24	9652(4)	6917(2)	3279(2)	30(1)
C25	8482(4)	8654(3)	1117(2)	34(1)
C26	8844(5)	8737(4)	276(3)	50(1)
C27	7992(6)	9592(4)	-305(3)	62(1)
C28	6822(6)	10322(4)	-52(3)	54(1)
C29	6510(4)	10195(3)	800(2)	39(1)
C30	5224(5)	10983(3)	1096(3)	51(1)
C31	8765(4)	7074(2)	4060(2)	28(1)
C32	9269(4)	6376(3)	4823(2)	38(1)
C33	8377(5)	6448(3)	5508(2)	43(1)
C34	6993(5)	7214(3)	5400(2)	39(1)
C35	6541(4)	7903(3)	4624(2)	31(1)
C36	5038(4)	8726(3)	4507(3)	43(1)

Table S38. Bond lengths [Å] and angles [°] for jonap13.

Br(1)-Zn(1)	2.4111(6)	C(9)-H(9)	0.9500
Br(2)-Zn(1)	2.4520(6)	C(9)-C(10)	1.374(6)
Zn(1)-N(1)	2.236(3)	C(10)-H(10)	0.9500
Zn(1)-N(2)	2.177(3)	C(10)-C(11)	1.395(6)
Zn(1)-N(3)	2.163(3)	C(11)-C(12)	1.499(5)
O(1)-H(1)	0.86(5)	C(12)-H(12A)	0.9800
O(1)-C(1)	1.429(4)	C(12)-H(12B)	0.9800
O(2)-C(3)	1.432(4)	C(12)-H(12C)	0.9800
O(2)-C(4)	1.411(4)	C(13)-C(14)	1.391(5)
O(3)-C(5)	1.422(5)	C(14)-H(14)	0.9500
O(3)-C(6)	1.413(4)	C(14)-C(15)	1.382(6)
N(1)-C(2)	1.524(4)	C(15)-H(15)	0.9500
N(1)-C(4)	1.480(4)	C(15)-C(16)	1.372(7)
N(1)-C(6)	1.493(4)	C(16)-H(16)	0.9500
N(2)-C(7)	1.349(4)	C(16)-C(17)	1.390(6)
N(2)-C(11)	1.348(5)	C(17)-C(18)	1.486(6)
N(3)-C(13)	1.332(5)	C(18)-H(18A)	0.9800
N(3)-C(17)	1.355(4)	C(18)-H(18B)	0.9800
C(1)-H(1A)	0.9900	C(18)-H(18C)	0.9800
C(1)-H(1B)	0.9900	Br(3)-Zn(2)	2.4172(5)
C(1)-C(2)	1.510(5)	Br(4)-Zn(2)	2.4732(5)
C(2)-C(3)	1.546(4)	Zn(2)-N(4)	2.214(3)
C(2)-C(5)	1.524(5)	Zn(2)-N(5)	2.172(3)
C(3)-H(3A)	0.9900	Zn(2)-N(6)	2.181(3)
C(3)-H(3B)	0.9900	O(4)-H(4)	0.94(11)
C(4)-H(4A)	1.0000	O(4)-C(19)	1.364(6)
C(4)-C(7)	1.521(5)	O(4')-H(4')	0.8501
C(5)-H(5A)	0.9900	O(4')-C(19)	1.292(10)
C(5)-H(5B)	0.9900	O(5)-C(21)	1.427(5)
C(6)-H(6)	1.0000	O(5)-C(22)	1.409(4)
C(6)-C(13)	1.526(5)	O(6)-C(23)	1.425(5)
C(7)-C(8)	1.377(5)	O(6)-C(24)	1.414(4)
C(8)-H(8)	0.9500	N(4)-C(20)	1.537(4)
C(8)-C(9)	1.379(6)	N(4)-C(22)	1.483(4)

N(4)-C(24)	1.490(4)	C(34)-H(34)	0.9500
N(5)-C(25)	1.340(5)	C(34)-C(35)	1.388(5)
N(5)-C(29)	1.348(5)	C(35)-C(36)	1.494(5)
N(6)-C(31)	1.343(4)	C(36)-H(36A)	0.9800
N(6)-C(35)	1.359(4)	C(36)-H(36B)	0.9800
C(19)-H(19A)	0.9900	C(36)-H(36C)	0.9800
C(19)-H(19B)	0.9900	Br(1)-Zn(1)-Br(2)	135.44(2)
C(19)-H(19C)	0.9900	N(1)-Zn(1)-Br(1)	116.34(7)
C(19)-H(19D)	0.9900	N(1)-Zn(1)-Br(2)	108.18(7)
C(19)-C(20)	1.524(5)	N(2)-Zn(1)-Br(1)	100.26(8)
C(20)-C(21)	1.539(5)	N(2)-Zn(1)-Br(2)	90.04(7)
C(20)-C(23)	1.529(5)	N(2)-Zn(1)-N(1)	78.21(10)
C(21)-H(21A)	0.9900	N(3)-Zn(1)-Br(1)	96.96(8)
C(21)-H(21B)	0.9900	N(3)-Zn(1)-Br(2)	89.57(8)
C(22)-H(22)	1.0000	N(3)-Zn(1)-N(1)	78.89(10)
C(22)-C(25)	1.515(5)	N(3)-Zn(1)-N(2)	155.75(11)
C(23)-H(23A)	0.9900	C(1)-O(1)-H(1)	106(3)
C(23)-H(23B)	0.9900	C(4)-O(2)-C(3)	104.1(2)
C(24)-H(24)	1.0000	C(6)-O(3)-C(5)	104.1(2)
C(24)-C(31)	1.520(5)	C(2)-N(1)-Zn(1)	128.96(19)
C(25)-C(26)	1.390(5)	C(4)-N(1)-Zn(1)	101.45(18)
C(26)-H(26)	0.9500	C(4)-N(1)-C(2)	102.7(2)
C(26)-C(27)	1.373(7)	C(4)-N(1)-C(6)	111.6(2)
C(27)-H(27)	0.9500	C(6)-N(1)-Zn(1)	107.88(18)
C(27)-C(28)	1.365(7)	C(6)-N(1)-C(2)	103.8(2)
C(28)-H(28)	0.9500	C(7)-N(2)-Zn(1)	112.1(2)
C(28)-C(29)	1.395(6)	C(11)-N(2)-Zn(1)	128.9(2)
C(29)-C(30)	1.504(6)	C(11)-N(2)-C(7)	119.0(3)
C(30)-H(30A)	0.9800	C(13)-N(3)-Zn(1)	115.2(2)
C(30)-H(30B)	0.9800	C(13)-N(3)-C(17)	119.3(3)
C(30)-H(30C)	0.9800	C(17)-N(3)-Zn(1)	125.6(3)
C(31)-C(32)	1.383(5)	O(1)-C(1)-H(1A)	110.5
C(32)-H(32)	0.9500	O(1)-C(1)-H(1B)	110.5
C(32)-C(33)	1.379(6)	O(1)-C(1)-C(2)	106.2(3)
C(33)-H(33)	0.9500	H(1A)-C(1)-H(1B)	108.7
C(33)-C(34)	1.381(6)	C(2)-C(1)-H(1A)	110.5

C(2)-C(1)-H(1B)	110.5	C(9)-C(8)-H(8)	121.1
N(1)-C(2)-C(3)	103.5(2)	C(8)-C(9)-H(9)	120.2
C(1)-C(2)-N(1)	113.1(3)	C(10)-C(9)-C(8)	119.6(4)
C(1)-C(2)-C(3)	112.5(3)	C(10)-C(9)-H(9)	120.2
C(1)-C(2)-C(5)	113.1(3)	C(9)-C(10)-H(10)	119.9
C(5)-C(2)-N(1)	102.4(2)	C(9)-C(10)-C(11)	120.2(4)
C(5)-C(2)-C(3)	111.4(3)	C(11)-C(10)-H(10)	119.9
O(2)-C(3)-C(2)	106.1(3)	N(2)-C(11)-C(10)	120.1(3)
O(2)-C(3)-H(3A)	110.5	N(2)-C(11)-C(12)	118.7(3)
O(2)-C(3)-H(3B)	110.5	C(10)-C(11)-C(12)	121.1(4)
C(2)-C(3)-H(3A)	110.5	C(11)-C(12)-H(12A)	109.5
C(2)-C(3)-H(3B)	110.5	C(11)-C(12)-H(12B)	109.5
H(3A)-C(3)-H(3B)	108.7	C(11)-C(12)-H(12C)	109.5
O(2)-C(4)-N(1)	106.4(2)	H(12A)-C(12)-H(12B)	109.5
O(2)-C(4)-H(4A)	109.3	H(12A)-C(12)-H(12C)	109.5
O(2)-C(4)-C(7)	111.7(3)	H(12B)-C(12)-H(12C)	109.5
N(1)-C(4)-H(4A)	109.3	N(3)-C(13)-C(6)	118.2(3)
N(1)-C(4)-C(7)	110.8(3)	N(3)-C(13)-C(14)	122.7(3)
C(7)-C(4)-H(4A)	109.3	C(14)-C(13)-C(6)	118.9(3)
O(3)-C(5)-C(2)	103.4(3)	C(13)-C(14)-H(14)	120.9
O(3)-C(5)-H(5A)	111.1	C(15)-C(14)-C(13)	118.2(4)
O(3)-C(5)-H(5B)	111.1	C(15)-C(14)-H(14)	120.9
C(2)-C(5)-H(5A)	111.1	C(14)-C(15)-H(15)	120.5
C(2)-C(5)-H(5B)	111.1	C(16)-C(15)-C(14)	119.1(4)
H(5A)-C(5)-H(5B)	109.0	C(16)-C(15)-H(15)	120.5
O(3)-C(6)-N(1)	107.8(3)	C(15)-C(16)-H(16)	119.8
O(3)-C(6)-H(6)	108.6	C(15)-C(16)-C(17)	120.4(4)
O(3)-C(6)-C(13)	110.8(3)	C(17)-C(16)-H(16)	119.8
N(1)-C(6)-H(6)	108.6	N(3)-C(17)-C(16)	120.3(4)
N(1)-C(6)-C(13)	112.4(3)	N(3)-C(17)-C(18)	118.7(4)
C(13)-C(6)-H(6)	108.6	C(16)-C(17)-C(18)	121.1(4)
N(2)-C(7)-C(4)	116.2(3)	C(17)-C(18)-H(18A)	109.5
N(2)-C(7)-C(8)	123.2(3)	C(17)-C(18)-H(18B)	109.5
C(8)-C(7)-C(4)	120.5(3)	C(17)-C(18)-H(18C)	109.5
C(7)-C(8)-H(8)	121.1	H(18A)-C(18)-H(18B)	109.5
C(7)-C(8)-C(9)	117.8(4)	H(18A)-C(18)-H(18C)	109.5

H(18B)-C(18)-H(18C)	109.5	C(20)-C(19)-H(19B)	108.6
Br(3)-Zn(2)-Br(4)	123.53(2)	C(20)-C(19)-H(19C)	109.3
N(4)-Zn(2)-Br(3)	120.89(7)	C(20)-C(19)-H(19D)	109.3
N(4)-Zn(2)-Br(4)	115.56(7)	N(4)-C(20)-C(21)	102.9(3)
N(5)-Zn(2)-Br(3)	103.02(8)	C(19)-C(20)-N(4)	115.3(3)
N(5)-Zn(2)-Br(4)	89.08(8)	C(19)-C(20)-C(21)	111.9(3)
N(5)-Zn(2)-N(4)	78.48(11)	C(19)-C(20)-C(23)	112.0(3)
N(5)-Zn(2)-N(6)	155.06(10)	C(23)-C(20)-N(4)	101.9(3)
N(6)-Zn(2)-Br(3)	97.39(7)	C(23)-C(20)-C(21)	112.1(3)
N(6)-Zn(2)-Br(4)	91.51(7)	O(5)-C(21)-C(20)	106.1(3)
N(6)-Zn(2)-N(4)	78.80(10)	O(5)-C(21)-H(21A)	110.5
C(19)-O(4)-H(4)	107(6)	O(5)-C(21)-H(21B)	110.5
C(19)-O(4')-H(4')	112.7	C(20)-C(21)-H(21A)	110.5
C(22)-O(5)-C(21)	104.7(3)	C(20)-C(21)-H(21B)	110.5
C(24)-O(6)-C(23)	103.7(3)	H(21A)-C(21)-H(21B)	108.7
C(20)-N(4)-Zn(2)	126.4(2)	O(5)-C(22)-N(4)	106.1(3)
C(22)-N(4)-Zn(2)	103.16(19)	O(5)-C(22)-H(22)	109.1
C(22)-N(4)-C(20)	103.7(2)	O(5)-C(22)-C(25)	111.6(3)
C(22)-N(4)-C(24)	110.2(3)	N(4)-C(22)-H(22)	109.1
C(24)-N(4)-Zn(2)	108.67(18)	N(4)-C(22)-C(25)	111.7(3)
C(24)-N(4)-C(20)	104.1(2)	C(25)-C(22)-H(22)	109.1
C(25)-N(5)-Zn(2)	111.9(2)	O(6)-C(23)-C(20)	104.4(3)
C(25)-N(5)-C(29)	118.6(3)	O(6)-C(23)-H(23A)	110.9
C(29)-N(5)-Zn(2)	129.2(2)	O(6)-C(23)-H(23B)	110.9
C(31)-N(6)-Zn(2)	114.5(2)	C(20)-C(23)-H(23A)	110.9
C(31)-N(6)-C(35)	118.4(3)	C(20)-C(23)-H(23B)	110.9
C(35)-N(6)-Zn(2)	127.0(2)	H(23A)-C(23)-H(23B)	108.9
O(4)-C(19)-H(19A)	108.6	O(6)-C(24)-N(4)	107.9(3)
O(4)-C(19)-H(19B)	108.6	O(6)-C(24)-H(24)	108.7
O(4)-C(19)-C(20)	114.7(4)	O(6)-C(24)-C(31)	110.3(3)
O(4')-C(19)-H(19C)	109.3	N(4)-C(24)-H(24)	108.7
O(4')-C(19)-H(19D)	109.3	N(4)-C(24)-C(31)	112.3(3)
O(4')-C(19)-C(20)	111.7(5)	C(31)-C(24)-H(24)	108.7
H(19A)-C(19)-H(19B)	107.6	N(5)-C(25)-C(22)	117.6(3)
H(19C)-C(19)-H(19D)	107.9	N(5)-C(25)-C(26)	122.9(4)
C(20)-C(19)-H(19A)	108.6	C(26)-C(25)-C(22)	119.3(3)

C(25)-C(26)-H(26)	121.1	C(32)-C(31)-C(24)	119.5(3)
C(27)-C(26)-C(25)	117.9(4)	C(31)-C(32)-H(32)	120.3
C(27)-C(26)-H(26)	121.1	C(33)-C(32)-C(31)	119.4(4)
C(26)-C(27)-H(27)	119.9	C(33)-C(32)-H(32)	120.3
C(28)-C(27)-C(26)	120.2(4)	C(32)-C(33)-H(33)	120.8
C(28)-C(27)-H(27)	119.9	C(32)-C(33)-C(34)	118.3(3)
C(27)-C(28)-H(28)	120.3	C(34)-C(33)-H(33)	120.8
C(27)-C(28)-C(29)	119.4(4)	C(33)-C(34)-H(34)	119.9
C(29)-C(28)-H(28)	120.3	C(33)-C(34)-C(35)	120.2(3)
N(5)-C(29)-C(28)	121.1(4)	C(35)-C(34)-H(34)	119.9
N(5)-C(29)-C(30)	118.3(3)	N(6)-C(35)-C(34)	121.0(3)
C(28)-C(29)-C(30)	120.6(4)	N(6)-C(35)-C(36)	118.8(3)
C(29)-C(30)-H(30A)	109.5	C(34)-C(35)-C(36)	120.1(3)
C(29)-C(30)-H(30B)	109.5	C(35)-C(36)-H(36A)	109.5
C(29)-C(30)-H(30C)	109.5	C(35)-C(36)-H(36B)	109.5
H(30A)-C(30)-H(30B)	109.5	C(35)-C(36)-H(36C)	109.5
H(30A)-C(30)-H(30C)	109.5	H(36A)-C(36)-H(36B)	109.5
H(30B)-C(30)-H(30C)	109.5	H(36A)-C(36)-H(36C)	109.5
N(6)-C(31)-C(24)	117.6(3)	H(36B)-C(36)-H(36C)	109.5
N(6)-C(31)-C(32)	122.6(3)		

Table S39. Anisotropic displacement parameters ($Å^2$x 10^3) for jonap13. The anisotropic displacement factor exponent takes the form: $-2\pi^2[h^2 a^{*2}U_{11} + ... + 2 h k a^* b^* U_{12}]$

	U_{11}	U_{22}	U_{33}	U_{23}	U_{13}	U_{12}
Br1	32(1)	45(1)	44(1)	-15(1)	9(1)	-19(1)
Br2	57(1)	45(1)	33(1)	-18(1)	1(1)	-4(1)
Zn1	24(1)	31(1)	27(1)	-7(1)	4(1)	-7(1)
O1	61(2)	36(1)	48(2)	-22(1)	24(1)	-21(1)
O2	27(1)	50(2)	36(1)	-14(1)	7(1)	-19(1)
O3	40(1)	36(1)	34(1)	-1(1)	11(1)	-11(1)
N1	23(1)	23(1)	25(1)	-8(1)	3(1)	-7(1)
N2	27(1)	31(1)	27(1)	-7(1)	0(1)	-7(1)
N3	28(1)	30(1)	31(1)	-10(1)	-1(1)	-1(1)
C1	42(2)	33(2)	44(2)	-19(2)	19(2)	-18(2)
C2	26(2)	28(2)	26(2)	-11(1)	4(1)	-10(1)
C3	27(2)	42(2)	35(2)	-15(2)	7(1)	-10(1)
C4	25(2)	34(2)	29(2)	-10(1)	3(1)	-12(1)
C5	37(2)	42(2)	25(2)	-10(1)	4(1)	-9(2)
C6	34(2)	25(2)	31(2)	-3(1)	1(1)	-12(1)
C7	27(2)	36(2)	27(2)	-10(1)	2(1)	-8(1)
C8	27(2)	55(2)	34(2)	-16(2)	2(1)	-3(2)
C9	40(2)	52(2)	35(2)	-12(2)	-6(2)	6(2)
C10	53(2)	38(2)	30(2)	-2(2)	-3(2)	-5(2)
C11	41(2)	41(2)	28(2)	-6(1)	2(1)	-16(2)
C12	50(2)	78(3)	38(2)	6(2)	7(2)	-30(2)
C13	35(2)	21(1)	32(2)	-8(1)	-1(1)	-6(1)
C14	50(2)	28(2)	34(2)	-3(1)	-3(2)	-8(2)
C15	52(2)	31(2)	47(2)	-2(2)	-16(2)	-4(2)
C16	34(2)	35(2)	61(3)	-15(2)	-12(2)	3(2)
C17	30(2)	36(2)	47(2)	-14(2)	-1(2)	2(1)
C18	30(2)	72(3)	64(3)	-14(2)	11(2)	5(2)
Br3	50(1)	29(1)	41(1)	-14(1)	9(1)	-14(1)
Br4	37(1)	55(1)	40(1)	-1(1)	-5(1)	-28(1)
Zn2	23(1)	24(1)	26(1)	-6(1)	4(1)	-7(1)
O4	43(3)	57(3)	51(3)	-28(2)	2(2)	-15(2)

O4'	61(5)	30(4)	80(6)	-6(4)	22(4)	-13(3)
O5	30(1)	50(2)	50(2)	-28(1)	15(1)	-13(1)
O6	23(1)	35(1)	57(2)	-5(1)	2(1)	-1(1)
N4	24(1)	25(1)	31(1)	-9(1)	4(1)	-9(1)
N5	32(1)	36(2)	26(1)	-7(1)	5(1)	-14(1)
N6	26(1)	25(1)	27(1)	-5(1)	1(1)	-10(1)
C19	34(2)	42(2)	58(2)	-20(2)	9(2)	-20(2)
C20	24(2)	40(2)	40(2)	-16(2)	9(1)	-15(1)
C21	32(2)	60(2)	50(2)	-26(2)	18(2)	-23(2)
C22	27(2)	37(2)	36(2)	-17(1)	10(1)	-12(1)
C23	25(2)	52(2)	49(2)	-14(2)	0(2)	-13(2)
C24	24(2)	25(2)	39(2)	-7(1)	1(1)	-6(1)
C25	34(2)	42(2)	30(2)	-12(1)	10(1)	-18(2)
C26	52(2)	68(3)	35(2)	-20(2)	21(2)	-23(2)
C27	74(3)	83(4)	27(2)	-7(2)	17(2)	-32(3)
C28	65(3)	63(3)	30(2)	3(2)	3(2)	-25(2)
C29	44(2)	40(2)	33(2)	-1(2)	2(2)	-18(2)
C30	51(2)	45(2)	41(2)	1(2)	-1(2)	-2(2)
C31	28(2)	25(2)	32(2)	-4(1)	-1(1)	-12(1)
C32	35(2)	34(2)	40(2)	1(2)	-8(2)	-12(2)
C33	53(2)	44(2)	31(2)	4(2)	-7(2)	-24(2)
C34	51(2)	43(2)	29(2)	-6(2)	8(2)	-25(2)
C35	35(2)	33(2)	30(2)	-8(1)	8(1)	-17(1)
C36	40(2)	42(2)	39(2)	-6(2)	20(2)	-8(2)

Table S40. Hydrogen coordinates (x 10^4) and isotropic displacement parameters (Å^2 x 10^3) for jonap13.

	x	y	z	U(eq)
H1	4340(60)	3300(40)	6420(30)	53(14)
H1A	3642	4238	7250	44
H1B	2281	4781	6554	44
H3A	6013	4554	6881	41
H3B	6030	5338	5994	41
H4A	4339	6841	7678	34
H5A	3963	5916	5102	43
H5B	2267	6209	5404	43
H6	3083	7807	6555	36
H8	6986	4765	8318	48
H9	7199	3471	9551	56
H10	5053	3340	10228	53
H12A	1495	5152	9794	85
H12B	2424	4063	10394	85
H12C	1674	4102	9518	85
H14	933	8709	5074	47
H15	-1667	9558	5001	56
H16	-3044	9179	6163	55
H18A	-2216	7086	7660	92
H18B	-3191	8281	7549	92
H18C	-1687	7794	8134	92
H4	9020(120)	9950(80)	3350(60)	70(30)
H4'	9686	10565	1981	89
H19A	11430	9461	2534	51
H19B	9726	9761	2226	51
H19C	9812	9487	3127	51
H19D	11454	9386	2799	51
H21A	11427	8555	1406	52
H21B	12624	7537	1974	52
H22	8844	7151	1790	38

H23A	12678	7278	3417	50
H23B	11248	7718	3952	50
H24	9382	6405	3039	36
H26	9654	8219	109	60
H27	8217	9675	-883	74
H28	6226	10911	-454	65
H30A	4645	10630	1483	77
H30B	4579	11446	610	77
H30C	5608	11387	1390	77
H32	10221	5851	4875	45
H33	8706	5983	6040	52
H34	6349	7269	5858	47
H36A	5135	9370	4585	64
H36B	4368	8509	4922	64
H36C	4625	8837	3936	64

Table S41. Torsion angles [°] for jonap13.

Zn1-N1-C2-C1	-8.5(4)	C2-N1-C6-O3	-12.3(3)
Zn1-N1-C2-C3	-130.5(2)	C2-N1-C6-C13	110.1(3)
Zn1-N1-C2-C5	113.5(3)	C3-O2-C4-N1	-43.2(3)
Zn1-N1-C4-O2	170.1(2)	C3-O2-C4-C7	77.8(3)
Zn1-N1-C4-C7	48.5(3)	C3-C2-C5-O3	-74.9(3)
Zn1-N1-C6-O3	-151.7(2)	C4-O2-C3-C2	32.6(3)
Zn1-N1-C6-C13	-29.3(3)	C4-N1-C2-C1	107.9(3)
Zn1-N2-C7-C4	4.9(4)	C4-N1-C2-C3	-14.1(3)
Zn1-N2-C7-C8	-177.8(3)	C4-N1-C2-C5	-130.1(3)
Zn1-N2-C11-C10	177.7(3)	C4-N1-C6-O3	97.7(3)
Zn1-N2-C11-C12	-3.7(5)	C4-N1-C6-C13	-139.9(3)
Zn1-N3-C13-C6	-9.2(4)	C4-C7-C8-C9	176.9(3)
Zn1-N3-C13-C14	176.7(3)	C5-O3-C6-N1	35.5(3)
Zn1-N3-C17-C16	-177.6(3)	C5-O3-C6-C13	-87.9(3)
Zn1-N3-C17-C18	2.3(5)	C5-C2-C3-O2	98.8(3)
O1-C1-C2-N1	-176.4(3)	C6-O3-C5-C2	-43.9(3)
O1-C1-C2-C3	-59.6(4)	C6-N1-C2-C1	-135.8(3)
O1-C1-C2-C5	67.8(4)	C6-N1-C2-C3	102.2(3)
O2-C4-C7-N2	-157.2(3)	C6-N1-C2-C5	-13.7(3)
O2-C4-C7-C8	25.4(4)	C6-N1-C4-O2	-75.3(3)
O3-C6-C13-N3	147.8(3)	C6-N1-C4-C7	163.1(3)
O3-C6-C13-C14	-37.9(4)	C6-C13-C14-C15	-173.0(3)
N1-C2-C3-O2	-10.6(3)	C7-N2-C11-C10	-2.9(5)
N1-C2-C5-O3	35.1(3)	C7-N2-C11-C12	175.7(4)
N1-C4-C7-N2	-38.8(4)	C7-C8-C9-C10	-1.8(6)
N1-C4-C7-C8	143.8(3)	C8-C9-C10-C11	1.6(6)
N1-C6-C13-N3	27.1(4)	C9-C10-C11-N2	0.8(6)
N1-C6-C13-C14	-158.6(3)	C9-C10-C11-C12	-177.8(4)
N2-C7-C8-C9	-0.3(6)	C11-N2-C7-C4	-174.6(3)
N3-C13-C14-C15	1.1(5)	C11-N2-C7-C8	2.7(5)
C1-C2-C3-O2	-133.0(3)	C13-N3-C17-C16	2.0(6)
C1-C2-C5-O3	157.2(3)	C13-N3-C17-C18	-178.1(4)
C2-N1-C4-O2	35.4(3)	C13-C14-C15-C16	1.6(6)
C2-N1-C4-C7	-86.2(3)	C14-C15-C16-C17	-2.4(6)

C15-C16-C17-N3	0.7(6)	N6-C31-C32-C33	1.0(5)
C15-C16-C17-C18	-179.2(4)	C19-C20-C21-O5	-139.2(3)
C17-N3-C13-C6	171.2(3)	C19-C20-C23-O6	156.6(3)
C17-N3-C13-C14	-2.9(5)	C20-N4-C22-O5	31.7(3)
Zn2-N4-C20-C19	-5.6(4)	C20-N4-C22-C25	-90.1(3)
Zn2-N4-C20-C21	-127.8(3)	C20-N4-C24-O6	-15.2(3)
Zn2-N4-C20-C23	116.0(3)	C20-N4-C24-C31	106.6(3)
Zn2-N4-C22-O5	164.9(2)	C21-O5-C22-N4	-42.3(4)
Zn2-N4-C22-C25	43.1(3)	C21-O5-C22-C25	79.6(4)
Zn2-N4-C24-O6	-152.1(2)	C21-C20-C23-O6	-76.6(3)
Zn2-N4-C24-C31	-30.3(3)	C22-O5-C21-C20	35.1(4)
Zn2-N5-C25-C22	-0.9(4)	C22-N4-C20-C19	112.5(3)
Zn2-N5-C25-C26	175.0(3)	C22-N4-C20-C21	-9.7(3)
Zn2-N5-C29-C28	-173.9(3)	C22-N4-C20-C23	-125.9(3)
Zn2-N5-C29-C30	6.0(5)	C22-N4-C24-O6	95.5(3)
Zn2-N6-C31-C24	-11.4(4)	C22-N4-C24-C31	-142.7(3)
Zn2-N6-C31-C32	175.2(3)	C22-C25-C26-C27	175.8(4)
Zn2-N6-C35-C34	-175.5(3)	C23-O6-C24-N4	36.8(4)
Zn2-N6-C35-C36	5.4(5)	C23-O6-C24-C31	-86.3(3)
O4-C19-C20-N4	73.5(5)	C23-C20-C21-O5	93.9(4)
O4-C19-C20-C21	-169.4(4)	C24-O6-C23-C20	-43.3(3)
O4-C19-C20-C23	-42.5(5)	C24-N4-C20-C19	-132.2(3)
O4'-C19-C20-N4	-69.7(5)	C24-N4-C20-C21	105.7(3)
O4'-C19-C20-C21	47.4(6)	C24-N4-C20-C23	-10.6(3)
O4'-C19-C20-C23	174.3(5)	C24-N4-C22-O5	-79.2(3)
O5-C22-C25-N5	-149.1(3)	C24-N4-C22-C25	159.0(3)
O5-C22-C25-C26	34.8(5)	C24-C31-C32-C33	-172.3(3)
O6-C24-C31-N6	149.4(3)	C25-N5-C29-C28	-1.0(6)
O6-C24-C31-C32	-36.9(4)	C25-N5-C29-C30	178.9(4)
N4-C20-C21-O5	-14.8(4)	C25-C26-C27-C28	-0.7(8)
N4-C20-C23-O6	32.8(3)	C26-C27-C28-C29	0.5(8)
N4-C22-C25-N5	-30.6(4)	C27-C28-C29-N5	0.3(7)
N4-C22-C25-C26	153.4(3)	C27-C28-C29-C30	-179.6(5)
N4-C24-C31-N6	29.0(4)	C29-N5-C25-C22	-175.0(3)
N4-C24-C31-C32	-157.4(3)	C29-N5-C25-C26	0.8(5)
N5-C25-C26-C27	0.0(7)	C31-N6-C35-C34	1.5(5)

C31-N6-C35-C36	-177.7(3)	C33-C34-C35-C36	179.5(4)
C31-C32-C33-C34	0.9(6)	C35-N6-C31-C24	171.3(3)
C32-C33-C34-C35	-1.5(6)	C35-N6-C31-C32	-2.1(5)
C33-C34-C35-N6	0.4(6)		

Table S42. Hydrogen bonds and close contacts for jonap13 [Å and °].

D-H...A	d(D-H)	d(H...A)	d(D...A)	<(DHA)
O1-H1...Br4#1	0.86(5)	2.53(5)	3.361(3)	163(4)
O4-H4...Br3	0.94(11)	2.25(11)	3.169(5)	164(8)
O4'-H4'...Br2#2	0.85	2.50	3.260(7)	149.1

Symmetry transformations used to generate equivalent atoms:

#1 -x+1,-y+1,-z+1 #2 -x+1,-y+2,-z+1

REFERENCE NUMBER: jonap14

5b

CRYSTAL STRUCTURE REPORT

C_{20} H_{21} F_6 N_3 O_9 S_2 Zn

or

[(κ^4-L^{Me})Zn(OTf)][OTf]

Report prepared for:

A. Panda, Prof. W. Jones

April 06, 2023

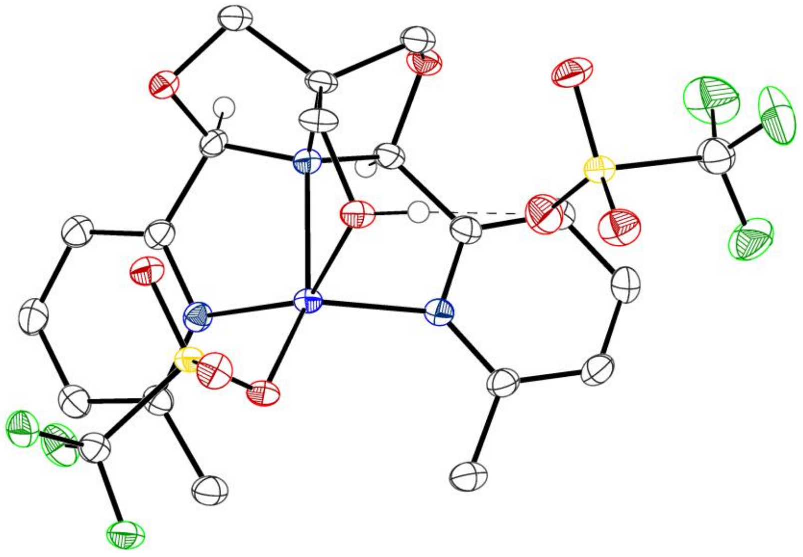

William W. Brennessel

X-ray Crystallographic Facility

Department of Chemistry, University of Rochester

120 Trustee Road

Rochester, NY 14627

Data collection

A crystal (0.203 x 0.031 x 0.015 mm^3) was placed onto a thin glass optical fiber or a nylon loop and mounted on a Rigaku XtaLAB Synergy-S Dualflex diffractometer equipped with a HyPix-6000HE HPC area detector for data collection at 100.00(10) K. A preliminary set of cell constants and an orientation matrix were calculated from a small sampling of reflections.[1] A short pre-experiment was run, from which an optimal data collection strategy was determined. The full data collection was carried out using a PhotonJet (Cu) X-ray source with frame times of 0.35 and 1.40 seconds and a detector distance of 34.0 mm. Series of frames were collected in 0.50º steps in ω at different 2θ, κ, and ϕ settings. After the intensity data were corrected for absorption, the final cell constants were calculated from the xyz centroids of 15203 strong reflections from the actual data collection after integration.[1] See Table S43 for additional crystal and refinement information.

Structure solution and refinement

The structure was solved using SHELXT[2] and refined using SHELXL.[3] The space group *P*-1 was determined based on intensity statistics. Most or all non-hydrogen atoms were assigned from the solution. Full-matrix least squares / difference Fourier cycles were performed which located any remaining non-hydrogen atoms. All non-hydrogen atoms were refined with anisotropic displacement parameters. The O-H hydrogen atom was found from the difference Fourier map and refined freely. All other hydrogen atoms were placed in ideal positions and refined as riding atoms with relative isotropic displacement parameters. The final full matrix least squares refinement converged to $R1 = 0.0394$ (F^2, $I > 2\sigma(I)$) and $wR2 = 0.1131$ (F^2, all data).

Structure description

The structure is the one suggested. The asymmetric unit contains one monocationic zinc complex and one triflate anion in general positions. The cation and anion are linked via hydrogen bonding (see figures and Table S49).

Structure manipulation and figure generation were performed using Olex2.[4] Unless noted otherwise all structural diagrams containing anisotropic displacement ellipsoids are drawn at the 50 % probability level.

Data collection, structure solution, and structure refinement were conducted at the X-ray Crystallographic Facility, B04 Hutchison Hall, Department of Chemistry, University of Rochester. The instrument was purchased with funding from NSF MRI program grant CHE-1725028. All publications arising from this report MUST either 1) include William W. Brennessel as a coauthor or 2) acknowledge William W. Brennessel and the X-ray Crystallographic Facility of the Department of Chemistry at the University of Rochester.

[1] *CrysAlisPro*, version 171.42.85a; Rigaku Corporation: Oxford, UK, 2023.

[2] Sheldrick, G. M. *SHELXT*, version 2018/2; *Acta. Crystallogr.* **2015**, *A71*, 3-8.

[3] Sheldrick, G. M. *SHELXL*, version 2019/2; *Acta. Crystallogr.* **2015**, *C71*, 3-8.

[4] Dolomanov, O. V.; Bourhis, L. J.; Gildea, R. J.; Howard, J. A. K.; Puschmann, H. *Olex2*, version 1.5; *J. Appl. Cryst.* **2009**, *42*, 339-341.

Some equations of interest:

$$R_{int} = \Sigma |F_o^2 - \langle F_o^2 \rangle| / \Sigma |F_o^2|$$

$$R1 = \Sigma ||F_o| - |F_c|| / \Sigma |F_o|$$

$$wR2 = [\Sigma[w(F_o^2 - F_c^2)^2] / \Sigma [w(F_o^2)^2]]^{1/2}$$

where $w = 1 / [\sigma^2 (F_o^2) + (aP)^2 + bP]$ and

$$P = 1/3 \max (0, F_o^2) + 2/3 F_c^2$$

$$GOF = S = [\Sigma[w(F_o^2 - F_c^2)^2] / (m-n)]^{1/2}$$

where m = number of reflections and n = number of parameters

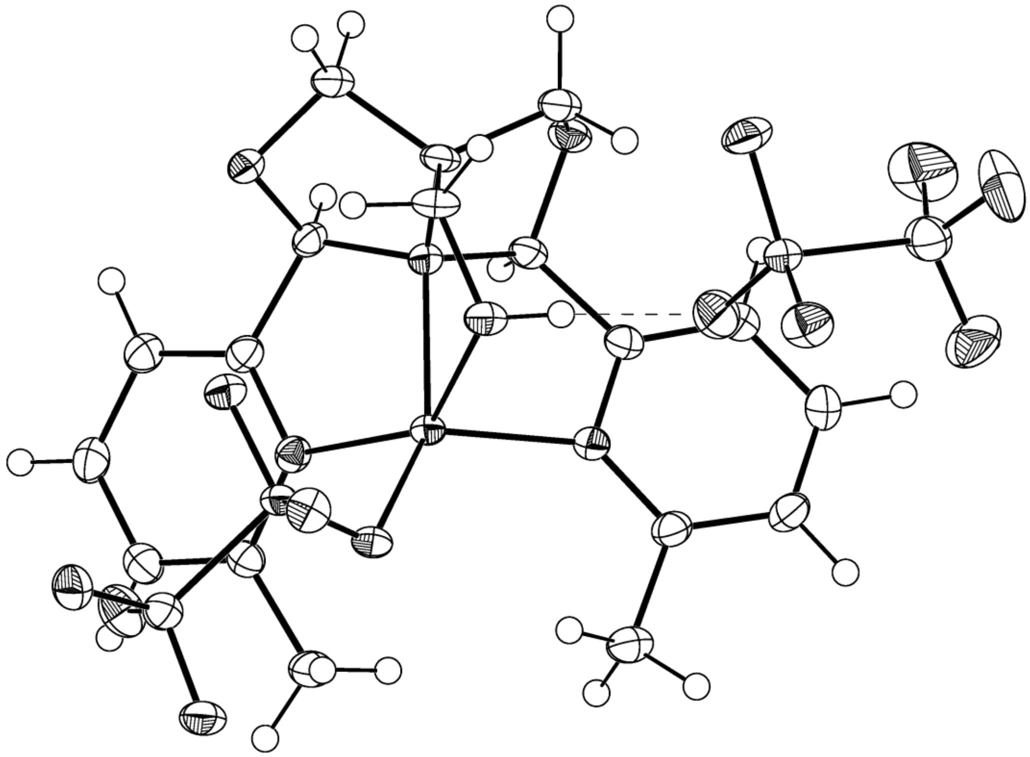

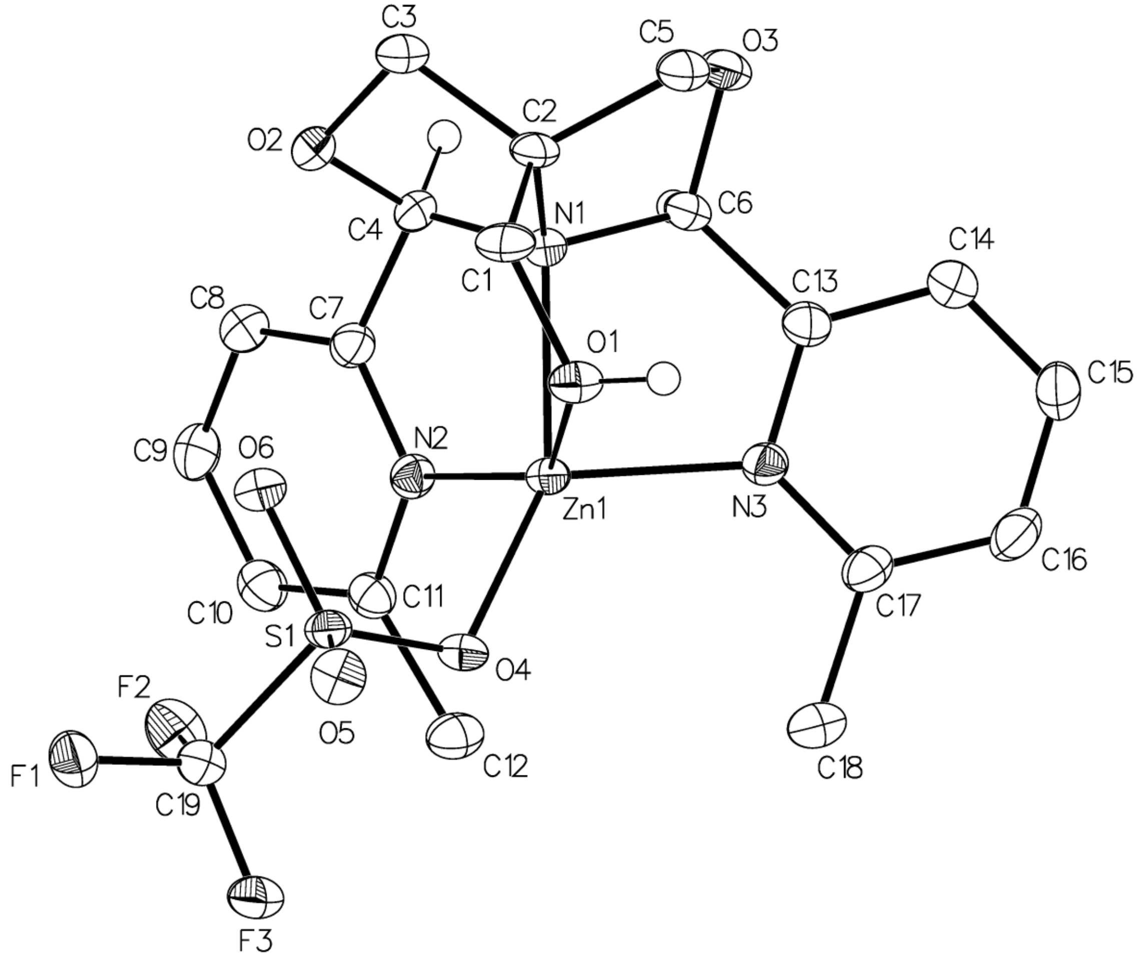
C3
C5
O3
C2
O2
C4
N1
C6
C1
C14
C13
C8
C7
O1
C15
N2
C9
O6
Zn1
N3
C16
C11
C17
C10
S1
O4
F2
O5
C18
C12
F1
C19
F3

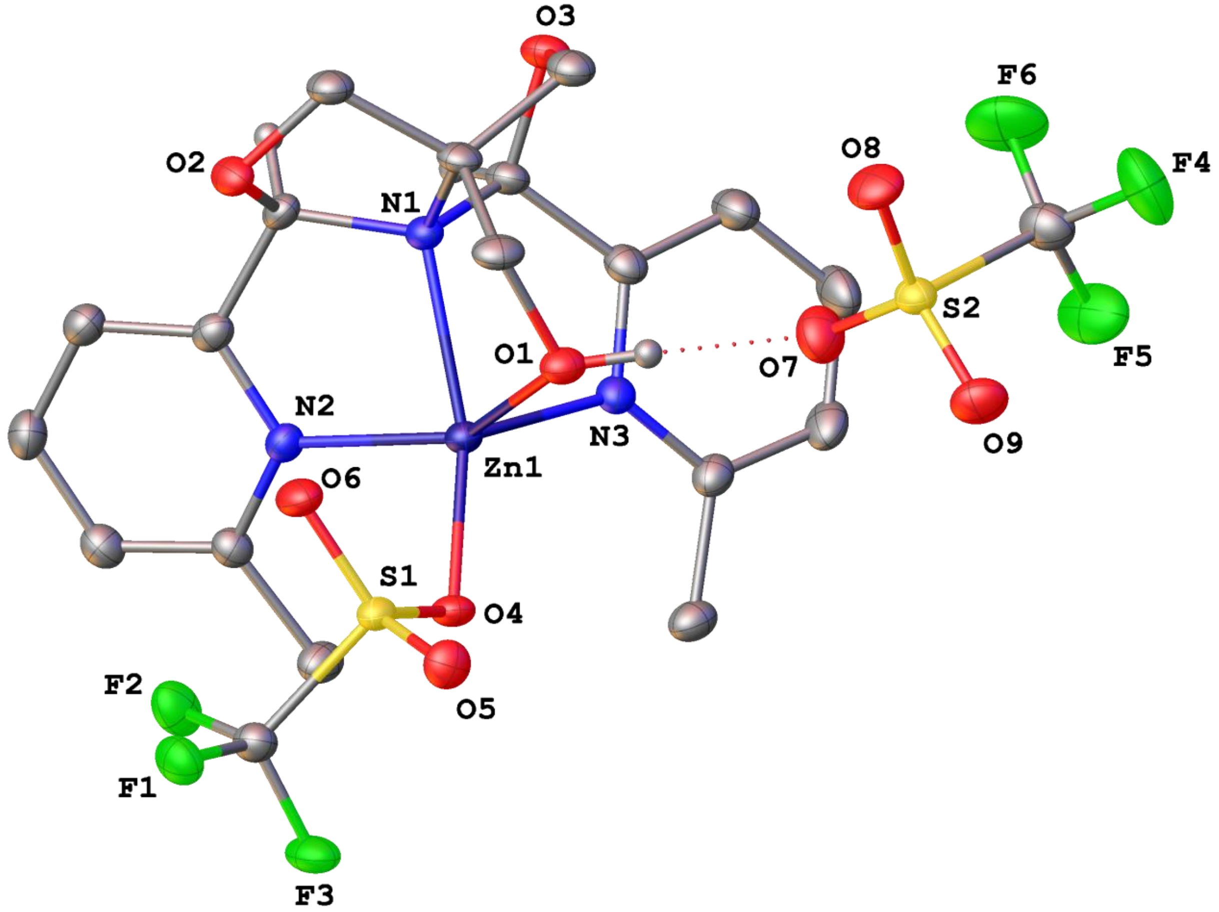
O3
F6
O2
O8
F4
N1
S2
O1
O7
F5
N2
N3
O9
Zn1
O6
S1
O4
F2
O5
F1
F3

Table S43. Crystal data and structure refinement for jonap14.

Identification code	jonap14	
Empirical formula	C20 H21 F6 N3 O9 S2 Zn	
Formula weight	690.89	
Temperature	100.00(10) K	
Wavelength	1.54184 Å	
Crystal system	triclinic	
Space group	*P*-1	
Unit cell dimensions	*a* = 9.2939(3) Å	α = 108.248(2)°
	b = 11.4913(3) Å	β = 90.750(3)°
	c = 12.9119(4) Å	γ = 99.190(2)°
Volume	1289.89(7) $Å^3$	
Z	2	
Density (calculated)	1.779 Mg/m^3	
Absorption coefficient	3.765 mm^{-1}	
F(000)	700	
Crystal color, morphology	colourless, plate	
Crystal size	0.203 x 0.031 x 0.015 mm^3	
Theta range for data collection	3.613 to 79.966°	
Index ranges	$-10 \leq h \leq 11$, $-14 \leq k \leq 14$, $-16 \leq l \leq 16$	
Reflections collected	23820	
Independent reflections	5441 [*R*(int) = 0.0505]	
Observed reflections	4811	
Completeness to theta = 74.504°	99.0%	
Absorption correction	Multi-scan	
Max. and min. transmission	1.00000 and 0.86265	
Refinement method	Full-matrix least-squares on F^2	
Data / restraints / parameters	5441 / 0 / 376	
Goodness-of-fit on F^2	1.114	
Final *R* indices [*I*>2sigma(*I*)]	*R*1 = 0.0394, *wR*2 = 0.1098	
R indices (all data)	*R*1 = 0.0443, *wR*2 = 0.1131	
Largest diff. peak and hole	0.642 and -0.838 e.$Å^{-3}$	

Table S44. Atomic coordinates (x 10^4) and equivalent isotropic displacement parameters (Å^{2}x 10^3) for jonap14. U_{eq} is defined as one third of the trace of the orthogonalized U_{ij} tensor.

	x	y	z	U_{eq}
Zn1	2566(1)	4093(1)	7508(1)	16(1)
S1	4259(1)	6369(1)	7135(1)	17(1)
F1	3872(2)	8375(1)	6706(1)	28(1)
F2	1969(2)	7482(2)	7268(2)	31(1)
F3	3784(2)	8525(1)	8413(1)	28(1)
O1	4604(2)	3566(2)	6850(2)	21(1)
O2	1320(2)	2671(2)	4594(1)	21(1)
O3	1717(2)	329(2)	6086(2)	22(1)
O4	3706(2)	5861(2)	7993(1)	19(1)
O5	5790(2)	6854(2)	7282(2)	24(1)
O6	3677(2)	5615(2)	6057(2)	26(1)
N1	1823(2)	2398(2)	6240(2)	16(1)
N2	572(2)	4447(2)	7087(2)	17(1)
N3	2439(2)	3022(2)	8505(2)	18(1)
C1	4374(3)	2864(2)	5716(2)	22(1)
C2	2960(3)	1904(2)	5484(2)	19(1)
C3	2219(3)	1722(2)	4363(2)	22(1)
C4	639(3)	2572(2)	5538(2)	18(1)
C5	3112(3)	698(2)	5718(2)	22(1)
C6	1345(3)	1447(2)	6777(2)	19(1)
C7	-25(3)	3713(2)	6097(2)	18(1)
C8	-1255(3)	3922(2)	5620(2)	22(1)
C9	-1895(3)	4924(3)	6189(2)	24(1)
C10	-1319(3)	5648(2)	7224(2)	22(1)
C11	-91(3)	5383(2)	7669(2)	18(1)
C12	496(3)	6077(2)	8823(2)	23(1)
C13	2044(3)	1801(2)	7934(2)	20(1)
C14	2170(3)	884(3)	8393(2)	28(1)
C15	2731(4)	1235(3)	9467(2)	33(1)
C16	3093(3)	2482(3)	10058(2)	28(1)
C17	2921(3)	3367(2)	9561(2)	21(1)

C18	3210(3)	4733(2)	10199(2)	24(1)
C19	3429(3)	7773(2)	7403(2)	21(1)
S2	7487(1)	2117(1)	7805(1)	19(1)
F4	7109(3)	1364(2)	9514(2)	49(1)
F5	8579(3)	450(2)	8393(2)	52(1)
F6	6290(3)	-19(2)	7998(2)	53(1)
O7	7697(2)	1452(2)	6684(2)	31(1)
O8	6084(2)	2515(2)	7977(2)	33(1)
O9	8718(2)	3008(2)	8392(2)	30(1)
C20	7351(3)	917(3)	8469(2)	30(1)

Table S45. Bond lengths [Å] and angles [°] for jonap14.

Zn(1)-O(1)	2.1816(18)	C(5)-H(5A)	0.9900
Zn(1)-O(4)	2.0348(17)	C(5)-H(5B)	0.9900
Zn(1)-N(1)	2.120(2)	C(6)-H(6)	1.0000
Zn(1)-N(2)	2.062(2)	C(6)-C(13)	1.527(3)
Zn(1)-N(3)	2.033(2)	C(7)-C(8)	1.380(4)
S(1)-O(4)	1.4675(19)	C(8)-H(8)	0.9500
S(1)-O(5)	1.4302(19)	C(8)-C(9)	1.385(4)
S(1)-O(6)	1.4346(19)	C(9)-H(9)	0.9500
S(1)-C(19)	1.837(3)	C(9)-C(10)	1.381(4)
F(1)-C(19)	1.329(3)	C(10)-H(10)	0.9500
F(2)-C(19)	1.340(3)	C(10)-C(11)	1.389(4)
F(3)-C(19)	1.321(3)	C(11)-C(12)	1.501(4)
O(1)-C(1)	1.428(3)	C(12)-H(12A)	0.9800
O(1)-H(1)	0.87(5)	C(12)-H(12B)	0.9800
O(2)-C(3)	1.439(3)	C(12)-H(12C)	0.9800
O(2)-C(4)	1.409(3)	C(13)-C(14)	1.380(4)
O(3)-C(5)	1.433(3)	C(14)-H(14)	0.9500
O(3)-C(6)	1.414(3)	C(14)-C(15)	1.385(4)
N(1)-C(2)	1.505(3)	C(15)-H(15)	0.9500
N(1)-C(4)	1.494(3)	C(15)-C(16)	1.380(4)
N(1)-C(6)	1.483(3)	C(16)-H(16)	0.9500
N(2)-C(7)	1.345(3)	C(16)-C(17)	1.388(4)
N(2)-C(11)	1.349(3)	C(17)-C(18)	1.505(4)
N(3)-C(13)	1.352(3)	C(18)-H(18A)	0.9800
N(3)-C(17)	1.345(3)	C(18)-H(18B)	0.9800
C(1)-H(1A)	0.9900	C(18)-H(18C)	0.9800
C(1)-H(1B)	0.9900	S(2)-O(7)	1.442(2)
C(1)-C(2)	1.534(3)	S(2)-O(8)	1.447(2)
C(2)-C(3)	1.533(4)	S(2)-O(9)	1.432(2)
C(2)-C(5)	1.535(3)	S(2)-C(20)	1.827(3)
C(3)-H(3A)	0.9900	F(4)-C(20)	1.321(4)
C(3)-H(3B)	0.9900	F(5)-C(20)	1.329(4)
C(4)-H(4)	1.0000	F(6)-C(20)	1.317(4)
C(4)-C(7)	1.516(3)	O(4)-Zn(1)-O(1)	84.99(7)

O(4)-Zn(1)-N(1)	149.28(8)	H(1A)-C(1)-H(1B)	108.0
O(4)-Zn(1)-N(2)	98.98(8)	C(2)-C(1)-H(1A)	109.4
N(1)-Zn(1)-O(1)	77.67(7)	C(2)-C(1)-H(1B)	109.4
N(2)-Zn(1)-O(1)	143.14(8)	N(1)-C(2)-C(1)	110.41(18)
N(2)-Zn(1)-N(1)	81.07(8)	N(1)-C(2)-C(3)	101.69(19)
N(3)-Zn(1)-O(1)	94.24(8)	N(1)-C(2)-C(5)	102.88(19)
N(3)-Zn(1)-O(4)	121.60(8)	C(1)-C(2)-C(5)	114.3(2)
N(3)-Zn(1)-N(1)	85.18(8)	C(3)-C(2)-C(1)	111.4(2)
N(3)-Zn(1)-N(2)	113.65(8)	C(3)-C(2)-C(5)	115.0(2)
O(4)-S(1)-C(19)	102.55(11)	O(2)-C(3)-C(2)	103.34(18)
O(5)-S(1)-O(4)	113.78(11)	O(2)-C(3)-H(3A)	111.1
O(5)-S(1)-O(6)	117.58(12)	O(2)-C(3)-H(3B)	111.1
O(5)-S(1)-C(19)	103.02(11)	C(2)-C(3)-H(3A)	111.1
O(6)-S(1)-O(4)	113.37(11)	C(2)-C(3)-H(3B)	111.1
O(6)-S(1)-C(19)	104.13(12)	H(3A)-C(3)-H(3B)	109.1
Zn(1)-O(1)-H(1)	119(3)	O(2)-C(4)-N(1)	104.57(19)
C(1)-O(1)-Zn(1)	110.52(14)	O(2)-C(4)-H(4)	109.4
C(1)-O(1)-H(1)	111(3)	O(2)-C(4)-C(7)	111.56(19)
C(4)-O(2)-C(3)	103.49(18)	N(1)-C(4)-H(4)	109.4
C(6)-O(3)-C(5)	104.72(18)	N(1)-C(4)-C(7)	112.51(19)
S(1)-O(4)-Zn(1)	117.37(10)	C(7)-C(4)-H(4)	109.4
C(2)-N(1)-Zn(1)	115.40(14)	O(3)-C(5)-C(2)	103.7(2)
C(4)-N(1)-Zn(1)	109.42(14)	O(3)-C(5)-H(5A)	111.0
C(4)-N(1)-C(2)	106.17(18)	O(3)-C(5)-H(5B)	111.0
C(6)-N(1)-Zn(1)	106.68(14)	C(2)-C(5)-H(5A)	111.0
C(6)-N(1)-C(2)	105.81(18)	C(2)-C(5)-H(5B)	111.0
C(6)-N(1)-C(4)	113.51(19)	H(5A)-C(5)-H(5B)	109.0
C(7)-N(2)-Zn(1)	114.53(16)	O(3)-C(6)-N(1)	105.69(19)
C(7)-N(2)-C(11)	119.4(2)	O(3)-C(6)-H(6)	108.9
C(11)-N(2)-Zn(1)	125.91(17)	O(3)-C(6)-C(13)	111.9(2)
C(13)-N(3)-Zn(1)	111.57(17)	N(1)-C(6)-H(6)	108.9
C(17)-N(3)-Zn(1)	127.66(17)	N(1)-C(6)-C(13)	112.61(19)
C(17)-N(3)-C(13)	119.7(2)	C(13)-C(6)-H(6)	108.9
O(1)-C(1)-H(1A)	109.4	N(2)-C(7)-C(4)	117.5(2)
O(1)-C(1)-H(1B)	109.4	N(2)-C(7)-C(8)	122.4(2)
O(1)-C(1)-C(2)	111.3(2)	C(8)-C(7)-C(4)	119.9(2)

C(7)-C(8)-H(8)	120.8	C(17)-C(16)-H(16)	120.2
C(7)-C(8)-C(9)	118.4(2)	N(3)-C(17)-C(16)	120.7(2)
C(9)-C(8)-H(8)	120.8	N(3)-C(17)-C(18)	118.2(2)
C(8)-C(9)-H(9)	120.4	C(16)-C(17)-C(18)	121.1(2)
C(10)-C(9)-C(8)	119.2(2)	C(17)-C(18)-H(18A)	109.5
C(10)-C(9)-H(9)	120.4	C(17)-C(18)-H(18B)	109.5
C(9)-C(10)-H(10)	120.1	C(17)-C(18)-H(18C)	109.5
C(9)-C(10)-C(11)	119.8(2)	H(18A)-C(18)-H(18B)	109.5
C(11)-C(10)-H(10)	120.1	H(18A)-C(18)-H(18C)	109.5
N(2)-C(11)-C(10)	120.6(2)	H(18B)-C(18)-H(18C)	109.5
N(2)-C(11)-C(12)	117.8(2)	F(1)-C(19)-S(1)	110.02(17)
C(10)-C(11)-C(12)	121.5(2)	F(1)-C(19)-F(2)	107.4(2)
C(11)-C(12)-H(12A)	109.5	F(2)-C(19)-S(1)	111.02(17)
C(11)-C(12)-H(12B)	109.5	F(3)-C(19)-S(1)	111.03(18)
C(11)-C(12)-H(12C)	109.5	F(3)-C(19)-F(1)	109.1(2)
H(12A)-C(12)-H(12B)	109.5	F(3)-C(19)-F(2)	108.1(2)
H(12A)-C(12)-H(12C)	109.5	O(7)-S(2)-O(8)	114.46(13)
H(12B)-C(12)-H(12C)	109.5	O(7)-S(2)-C(20)	103.38(13)
N(3)-C(13)-C(6)	118.2(2)	O(8)-S(2)-C(20)	102.34(14)
N(3)-C(13)-C(14)	121.9(2)	O(9)-S(2)-O(7)	115.33(13)
C(14)-C(13)-C(6)	119.8(2)	O(9)-S(2)-O(8)	115.63(13)
C(13)-C(14)-H(14)	120.7	O(9)-S(2)-C(20)	103.09(14)
C(13)-C(14)-C(15)	118.6(3)	F(4)-C(20)-S(2)	111.5(2)
C(15)-C(14)-H(14)	120.7	F(4)-C(20)-F(5)	108.5(3)
C(14)-C(15)-H(15)	120.3	F(5)-C(20)-S(2)	110.3(2)
C(16)-C(15)-C(14)	119.5(3)	F(6)-C(20)-S(2)	111.5(2)
C(16)-C(15)-H(15)	120.3	F(6)-C(20)-F(4)	108.1(3)
C(15)-C(16)-H(16)	120.2	F(6)-C(20)-F(5)	106.7(3)
C(15)-C(16)-C(17)	119.6(3)		

Table S46. Anisotropic displacement parameters ($Å^2$x 10^3) for jonap14. The anisotropic displacement factor exponent takes the form: $-2\pi^2$[h^2 $a^{*2}U_{11}$ + ... + 2 h k a* b* U_{12}]

	U_{11}	U_{22}	U_{33}	U_{23}	U_{13}	U_{12}
Zn1	18(1)	13(1)	14(1)	2(1)	-1(1)	3(1)
S1	19(1)	15(1)	15(1)	1(1)	0(1)	4(1)
F1	36(1)	24(1)	27(1)	12(1)	5(1)	8(1)
F2	22(1)	30(1)	43(1)	14(1)	3(1)	8(1)
F3	43(1)	18(1)	20(1)	2(1)	4(1)	6(1)
O1	22(1)	19(1)	19(1)	1(1)	2(1)	4(1)
O2	31(1)	19(1)	14(1)	5(1)	2(1)	6(1)
O3	29(1)	14(1)	21(1)	2(1)	3(1)	2(1)
O4	23(1)	13(1)	18(1)	0(1)	-1(1)	2(1)
O5	19(1)	27(1)	26(1)	9(1)	3(1)	3(1)
O6	39(1)	20(1)	18(1)	2(1)	-4(1)	5(1)
N1	20(1)	14(1)	14(1)	3(1)	0(1)	3(1)
N2	17(1)	18(1)	17(1)	5(1)	-1(1)	2(1)
N3	20(1)	18(1)	16(1)	4(1)	2(1)	6(1)
C1	24(1)	19(1)	19(1)	-2(1)	6(1)	5(1)
C2	25(1)	14(1)	16(1)	0(1)	4(1)	6(1)
C3	28(1)	17(1)	17(1)	0(1)	2(1)	4(1)
C4	22(1)	17(1)	13(1)	4(1)	-3(1)	1(1)
C5	29(1)	17(1)	19(1)	2(1)	4(1)	6(1)
C6	26(1)	13(1)	17(1)	3(1)	2(1)	3(1)
C7	21(1)	18(1)	16(1)	6(1)	0(1)	0(1)
C8	25(1)	22(1)	19(1)	5(1)	-2(1)	3(1)
C9	24(1)	28(1)	23(1)	11(1)	-1(1)	6(1)
C10	23(1)	21(1)	24(1)	8(1)	4(1)	6(1)
C11	18(1)	18(1)	20(1)	6(1)	3(1)	4(1)
C12	22(1)	24(1)	21(1)	2(1)	1(1)	7(1)
C13	24(1)	19(1)	19(1)	5(1)	5(1)	8(1)
C14	41(2)	20(1)	22(1)	6(1)	2(1)	9(1)
C15	52(2)	30(1)	24(1)	13(1)	4(1)	18(1)
C16	35(1)	35(2)	15(1)	6(1)	1(1)	14(1)
C17	21(1)	25(1)	16(1)	4(1)	2(1)	7(1)

C18	24(1)	24(1)	20(1)	1(1)	0(1)	4(1)
C19	25(1)	18(1)	20(1)	5(1)	1(1)	3(1)
S2	24(1)	17(1)	16(1)	3(1)	2(1)	5(1)
F4	81(2)	43(1)	26(1)	16(1)	12(1)	7(1)
F5	55(1)	49(1)	72(2)	37(1)	10(1)	24(1)
F6	61(1)	28(1)	59(1)	12(1)	7(1)	-14(1)
O7	42(1)	31(1)	15(1)	2(1)	5(1)	7(1)
O8	31(1)	39(1)	33(1)	11(1)	4(1)	16(1)
O9	30(1)	24(1)	30(1)	6(1)	1(1)	-4(1)
C20	38(2)	24(1)	28(1)	9(1)	6(1)	4(1)

Table S47. Hydrogen coordinates (x 10^4) and isotropic displacement parameters (Å^2 x 10^3) for jonap14.

	x	y	z	U(eq)
H1A	5211	2431	5483	27
H1B	4320	3435	5285	27
H3A	2950	1840	3838	26
H3B	1619	882	4063	26
H4	-143	1818	5333	22
H5A	3320	55	5048	27
H5B	3902	849	6290	27
H6	259	1339	6809	23
H8	-1654	3391	4919	27
H9	-2720	5111	5871	29
H10	-1761	6326	7630	27
H12A	377	5510	9258	35
H12B	-41	6761	9136	35
H12C	1535	6414	8831	35
H14	1879	30	7982	33
H15	2865	623	9794	39
H16	3457	2734	10799	34
H18A	3649	5204	9734	36
H18B	3880	4882	10837	36
H18C	2288	5004	10442	36
H1	5130(50)	3210(40)	7190(40)	57(13)

Table S48. Torsion angles [°] for jonap14.

Zn1-O1-C1-C2	-42.8(2)	N1-C2-C3-O2	30.3(2)
Zn1-N1-C2-C1	-8.3(2)	N1-C2-C5-O3	26.9(2)
Zn1-N1-C2-C3	-126.62(16)	N1-C4-C7-N2	4.2(3)
Zn1-N1-C2-C5	114.03(17)	N1-C4-C7-C8	-171.7(2)
Zn1-N1-C4-O2	103.01(16)	N1-C6-C13-N3	28.0(3)
Zn1-N1-C4-C7	-18.2(2)	N1-C6-C13-C14	-156.1(2)
Zn1-N1-C6-O3	-144.62(15)	N2-C7-C8-C9	-0.2(4)
Zn1-N1-C6-C13	-22.2(2)	N3-C13-C14-C15	-0.6(4)
Zn1-N2-C7-C4	12.9(3)	C1-C2-C3-O2	-87.4(2)
Zn1-N2-C7-C8	-171.24(19)	C1-C2-C5-O3	146.6(2)
Zn1-N2-C11-C10	169.78(18)	C2-N1-C4-O2	-22.1(2)
Zn1-N2-C11-C12	-13.3(3)	C2-N1-C4-C7	-143.38(19)
Zn1-N3-C13-C6	-17.5(3)	C2-N1-C6-O3	-21.2(2)
Zn1-N3-C13-C14	166.7(2)	C2-N1-C6-C13	101.2(2)
Zn1-N3-C17-C16	-163.7(2)	C3-O2-C4-N1	42.2(2)
Zn1-N3-C17-C18	18.8(3)	C3-O2-C4-C7	164.12(19)
O1-C1-C2-N1	33.6(3)	C3-C2-C5-O3	-82.8(2)
O1-C1-C2-C3	145.8(2)	C4-O2-C3-C2	-45.7(2)
O1-C1-C2-C5	-81.8(3)	C4-N1-C2-C1	113.1(2)
O2-C4-C7-N2	-112.9(2)	C4-N1-C2-C3	-5.2(2)
O2-C4-C7-C8	71.1(3)	C4-N1-C2-C5	-124.6(2)
O3-C6-C13-N3	146.9(2)	C4-N1-C6-O3	94.8(2)
O3-C6-C13-C14	-37.2(3)	C4-N1-C6-C13	-142.8(2)
O4-S1-C19-F1	177.84(17)	C4-C7-C8-C9	175.5(2)
O4-S1-C19-F2	-63.4(2)	C5-O3-C6-N1	39.4(2)
O4-S1-C19-F3	56.91(19)	C5-O3-C6-C13	-83.5(2)
O5-S1-O4-Zn1	-126.86(12)	C5-C2-C3-O2	140.6(2)
O5-S1-C19-F1	59.5(2)	C6-O3-C5-C2	-41.3(2)
O5-S1-C19-F2	178.19(18)	C6-N1-C2-C1	-126.0(2)
O5-S1-C19-F3	-61.5(2)	C6-N1-C2-C3	115.7(2)
O6-S1-O4-Zn1	11.01(15)	C6-N1-C2-C5	-3.7(2)
O6-S1-C19-F1	-63.8(2)	C6-N1-C4-O2	-137.96(19)
O6-S1-C19-F2	54.9(2)	C6-N1-C4-C7	100.8(2)
O6-S1-C19-F3	175.28(17)	C6-C13-C14-C15	-176.3(3)

C7-N2-C11-C10	-4.7(3)	C15-C16-C17-C18	175.9(3)
C7-N2-C11-C12	172.3(2)	C17-N3-C13-C6	173.4(2)
C7-C8-C9-C10	-2.4(4)	C17-N3-C13-C14	-2.4(4)
C8-C9-C10-C11	1.6(4)	C19-S1-O4-Zn1	122.64(12)
C9-C10-C11-N2	2.0(4)	O7-S2-C20-F4	-178.1(2)
C9-C10-C11-C12	-174.8(2)	O7-S2-C20-F5	61.3(2)
C11-N2-C7-C4	-172.0(2)	O7-S2-C20-F6	-57.1(2)
C11-N2-C7-C8	3.8(4)	O8-S2-C20-F4	-58.9(3)
C13-N3-C17-C16	3.5(4)	O8-S2-C20-F5	-179.5(2)
C13-N3-C17-C18	-174.1(2)	O8-S2-C20-F6	62.1(2)
C13-C14-C15-C16	2.5(5)	O9-S2-C20-F4	61.4(3)
C14-C15-C16-C17	-1.5(5)	O9-S2-C20-F5	-59.1(2)
C15-C16-C17-N3	-1.6(4)	O9-S2-C20-F6	-177.5(2)

Table S49. Hydrogen bonds and close contacts for jonap14 [Å and °].

D-H...A	d(D-H)	d(H...A)	d(D...A)	<(DHA)
O1-H1...O8	0.87(5)	1.78(5)	2.655(3)	175(5)

REFERENCE NUMBER: jonap23

5c

CRYSTAL STRUCTURE REPORT

$C_{20} H_{23.22} F_6 N_3 O_{12.11} S_2 Zn$

or

$[(\kappa^4\text{-}L^{OMe})Zn(OTf)(OH_2)][OTf] \cdot 0.11\ H_2O$

Report prepared for:

C. Wood, A. Panda, Prof. W. Jones

June 30, 2023

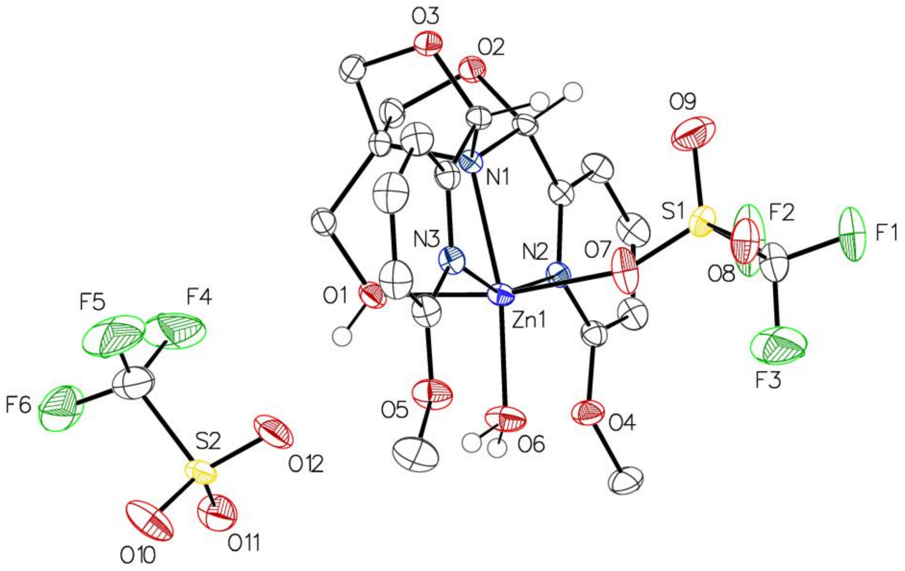

William W. Brennessel

X-ray Crystallographic Facility

Department of Chemistry, University of Rochester

120 Trustee Road

Rochester, NY 14627

Data collection

A crystal (0.324 x 0.038 x 0.015 mm^3) was placed onto a nylon loop and mounted on a Rigaku XtaLAB Synergy-S Dualflex diffractometer equipped with a HyPix-6000HE HPC area detector for data collection at 100.01(10) K. A preliminary set of cell constants and an orientation matrix were calculated from a small sampling of reflections.[1] A short pre-experiment was run, from which an optimal data collection strategy was determined. The full data collection was carried out using a PhotonJet (Cu) X-ray source with frame times of 0.27 and 1.08 seconds and a detector distance of 34.0 mm. Series of frames were collected in 0.50º steps in ω at different 2θ, κ, and ϕ settings. After the intensity data were corrected for absorption, the final cell constants were calculated from the xyz centroids of 31638 strong reflections from the actual data collection after integration.[1] See Table S50 for additional crystal and refinement information.

Structure solution and refinement

The structure was solved using SHELXT[2] and refined using SHELXL.[3] The space group *P*-1 was determined based on intensity statistics. Most or all non-hydrogen atoms were assigned from the solution. Full-matrix least squares / difference Fourier cycles were performed which located any remaining non-hydrogen atoms. All non-hydrogen atoms were refined with anisotropic displacement parameters. The O1-H and O6-H hydrogen atoms were found from the difference Fourier map and refined freely. The hydrogen atoms on partial-occupancy water molecule O13 (see below) were placed in positions reasonable for hydrogen bonding and then given riding models. All other hydrogen atoms were placed in ideal positions and refined as riding atoms with relative isotropic displacement parameters. The final full matrix least squares refinement converged to $R1 = 0.0346$ (F^2, $I > 2\sigma(I)$) and $wR2 = 0.0912$ (F^2, all data).

Structure description

The structure is the one suggested. The asymmetric unit contains one monocationic Zn complex, one triflate anion, and a water solvent molecule of crystallization that is of partial occupancy (0.11), all in general positions. Hydrogen bonding (O-H…O) links the asymmetric unit pairwise (see figure and Table S56).

Structure manipulation and figure generation were performed using Olex2.[4] Unless noted otherwise all structural diagrams containing anisotropic displacement ellipsoids are drawn at the 50 % probability level.

Data collection, structure solution, and structure refinement were conducted at the X-ray Crystallographic Facility, B04 Hutchison Hall, Department of Chemistry, University of Rochester. The instrument was purchased with funding from NSF MRI program grant CHE-1725028. All publications arising from this report MUST either 1) include William W. Brennessel as a coauthor or 2) acknowledge William W. Brennessel and the X-ray Crystallographic Facility of the Department of Chemistry at the University of Rochester.

1 *CrysAlisPro*, version 171.42.90a; Rigaku Corporation: Oxford, UK, 2023.

2 Sheldrick, G. M. *SHELXT*, version 2018/2; *Acta. Crystallogr.* **2015**, *A71*, 3-8.

3 Sheldrick, G. M. *SHELXL*, version 2019/2; *Acta. Crystallogr.* **2015**, *C71*, 3-8.

4 Dolomanov, O. V.; Bourhis, L. J.; Gildea, R. J.; Howard, J. A. K.; Puschmann, H. *Olex2*, version 1.5; *J. Appl. Cryst.* **2009**, *42*, 339-341.

Some equations of interest:

$$R_{int} = \Sigma |F_o^2 - \langle F_o^2 \rangle| / \Sigma |F_o^2|$$

$$R1 = \Sigma ||F_o| - |F_c|| / \Sigma |F_o|$$

$$wR2 = [\Sigma[w(F_o^2 - F_c^2)^2] / \Sigma [w(F_o^2)^2]]^{1/2}$$

$$\text{where } w = 1 / [\sigma^2 (F_o^2) + (aP)^2 + bP] \text{ and}$$

$$P = 1/3 \max (0, F_o^2) + 2/3 F_c^2$$

$$\text{GOF} = S = [\Sigma[w(F_o^2 - F_c^2)^2] / (m-n)]^{1/2}$$

where m = number of reflections and n = number of parameters

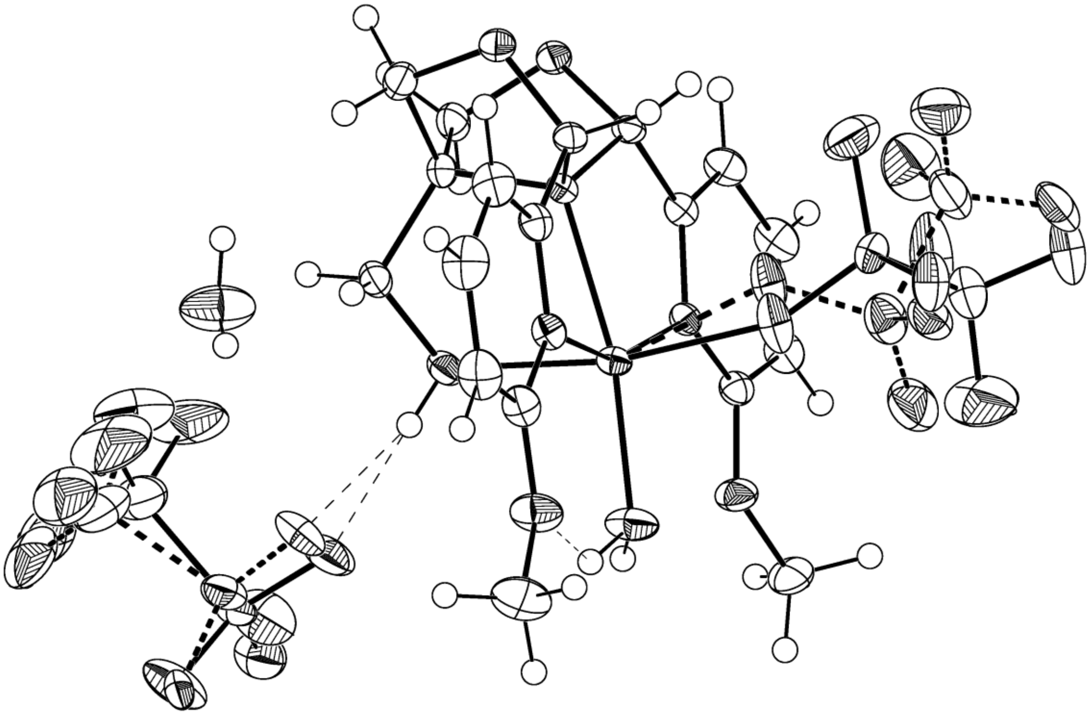

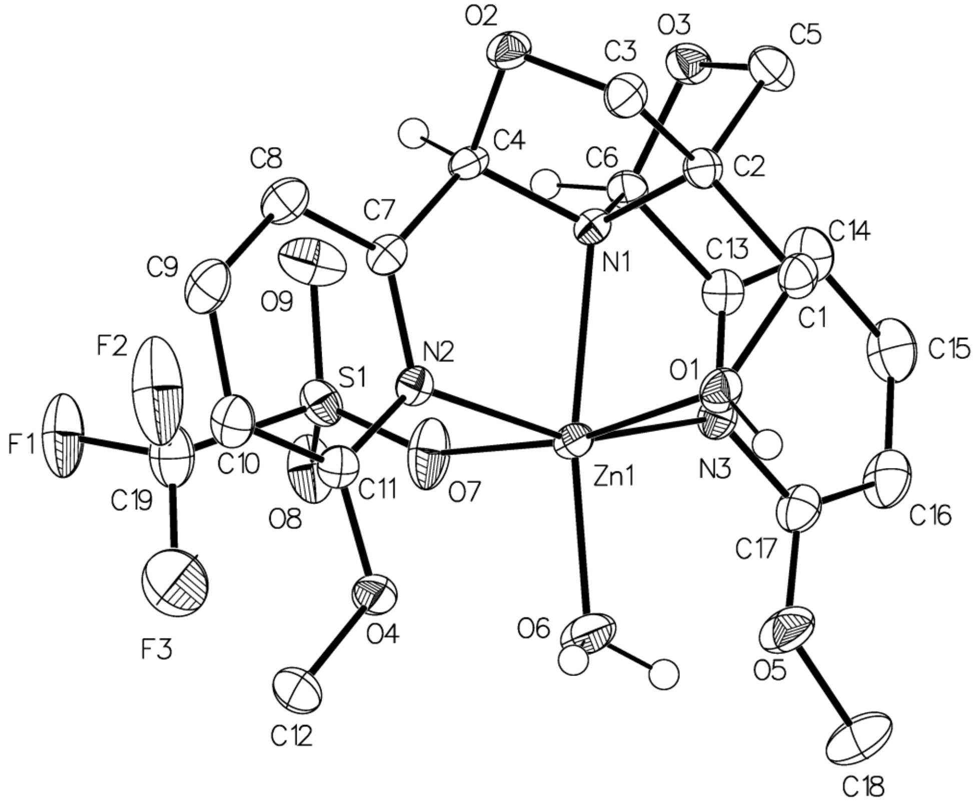
O2
O3
C5
C3
C4
C6
C2
C8
C7
N1
C13
C14
C9
O9
C1
F2
S1
N2
O1
C15
F1
C10
Zn1
N3
C19
C11
O7
C16
O8
C17
O4
O6
O5
F3
C12
C18

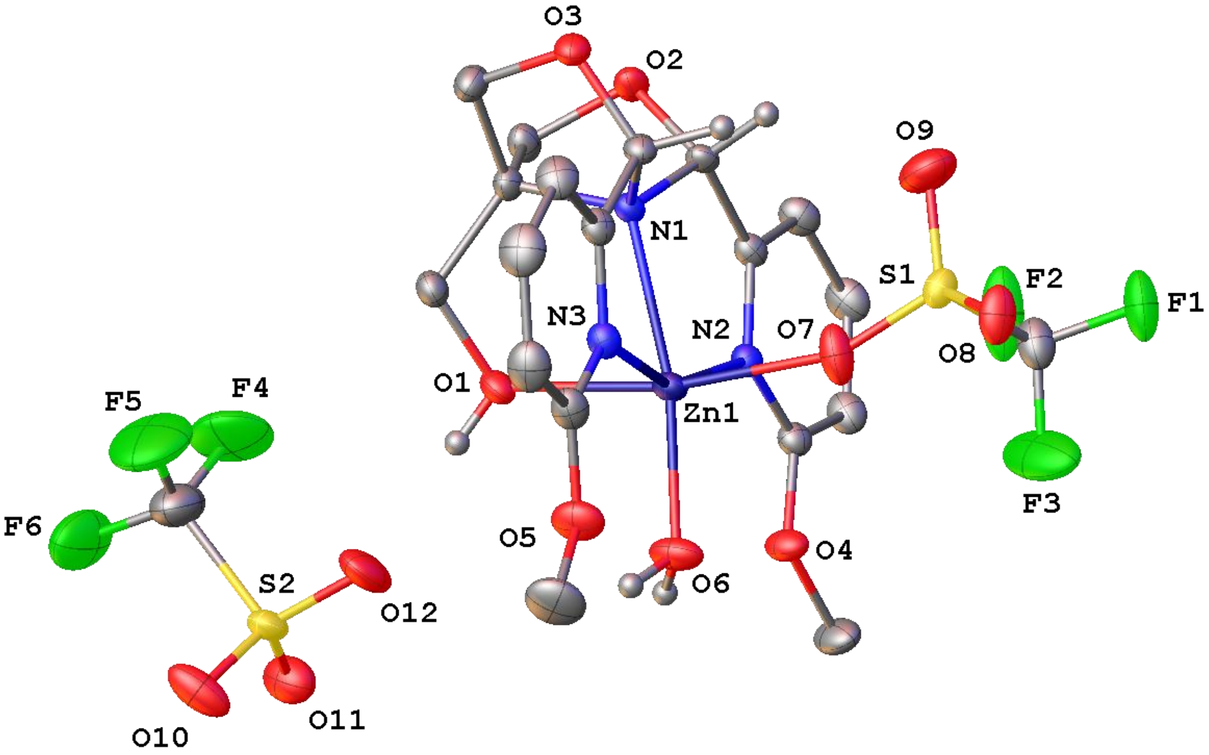
O3
O2
O9
N1
S1
F2
F1
N3
N2
O7
O8
F5
F4
O1
Zn1
F3
F6
O5
O4
S2
O6
O12
O10
O11

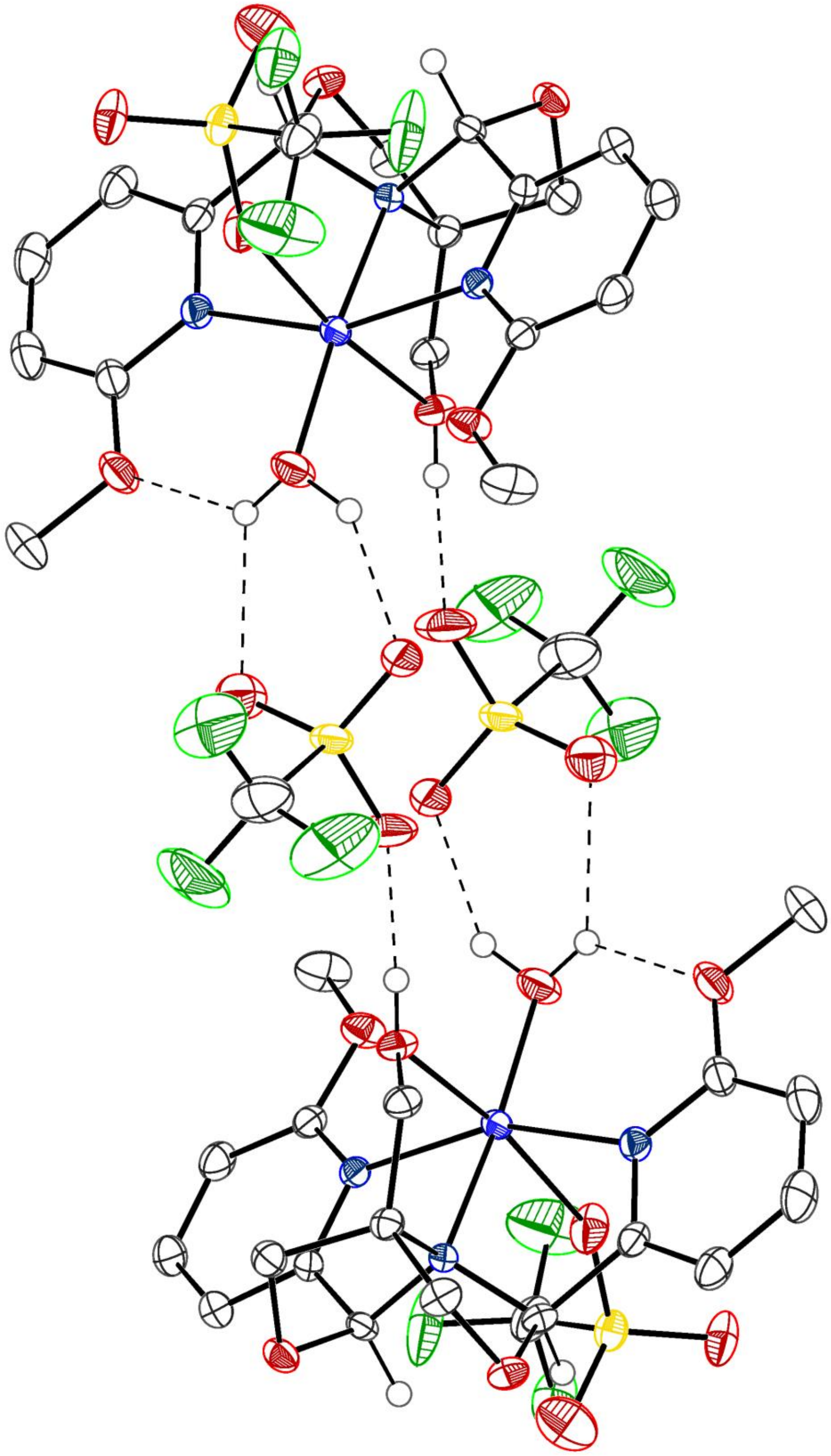

Table S50. Crystal data and structure refinement for jonap23.

Identification code	jonap23	
Empirical formula	C20 H23.22 F6 N3 O12.11 S2 Zn	
Formula weight	742.88	
Temperature	100.01(10) K	
Wavelength	1.54184 Å	
Crystal system	triclinic	
Space group	*P*-1	
Unit cell dimensions	*a* = 8.8083(3) Å	α = 89.822(2)°
	b = 10.1312(2) Å	β = 81.403(2)°
	c = 17.2325(2) Å	γ = 66.483(2)°
Volume	1391.52(6) $Å^3$	
Z	2	
Density (calculated)	1.773 Mg/m^3	
Absorption coefficient	3.626 mm^{-1}	
F(000)	754	
Crystal color, morphology	colourless, needle	
Crystal size	0.324 x 0.038 x 0.015 mm^3	
Theta range for data collection	4.770 to 80.346°	
Index ranges	$-9 \le h \le 11$, $-12 \le k \le 12$, $-21 \le l \le 21$	
Reflections collected	47441	
Independent reflections	5978 [*R*(int) = 0.0543]	
Observed reflections	5477	
Completeness to theta = 74.504°	99.9%	
Absorption correction	Multi-scan	
Max. and min. transmission	1.00000 and 0.49231	
Refinement method	Full-matrix least-squares on F^2	
Data / restraints / parameters	5978 / 329 / 565	
Goodness-of-fit on F^2	1.066	
Final *R* indices [*I*>2sigma(*I*)]	*R*1 = 0.0346, *wR*2 = 0.0892	
R indices (all data)	*R*1 = 0.0376, *wR*2 = 0.0912	
Largest diff. peak and hole	0.846 and -0.607 e.$Å^{-3}$	

Table S51. Atomic coordinates (x 10^4) and equivalent isotropic displacement parameters ($Å^2$x 10^3) for jonap23. U_{eq} is defined as one third of the trace of the orthogonalized U_{ij} tensor.

	x	y	z	U_{eq}
Zn1	7381(1)	3549(1)	7452(1)	15(1)
O1	7451(2)	5663(2)	7176(1)	21(1)
O2	6639(2)	5846(2)	9558(1)	20(1)
O3	3212(2)	6212(2)	9083(1)	21(1)
O4	11328(2)	1766(2)	7174(1)	24(1)
O5	5570(2)	3504(2)	5936(1)	30(1)
O6	8607(2)	2602(2)	6416(1)	29(1)
N1	5951(2)	4955(2)	8481(1)	14(1)
N2	9300(2)	3102(2)	8143(1)	15(1)
N3	4845(2)	4224(2)	7220(1)	18(1)
C1	5967(2)	6844(2)	7538(1)	19(1)
C2	5586(2)	6531(2)	8397(1)	17(1)
C3	6680(3)	6783(2)	8940(1)	20(1)
C4	6889(2)	4540(2)	9154(1)	16(1)
C5	3697(3)	7310(2)	8747(1)	22(1)
C6	4284(2)	4921(2)	8636(1)	17(1)
C7	8765(2)	3677(2)	8888(1)	16(1)
C8	9840(3)	3479(2)	9428(1)	22(1)
C9	11538(3)	2641(2)	9189(1)	25(1)
C10	12109(3)	2019(2)	8433(1)	23(1)
C11	10945(2)	2287(2)	7926(1)	18(1)
C12	13051(3)	851(3)	6885(1)	33(1)
C13	3675(2)	4835(2)	7861(1)	18(1)
C14	1972(3)	5359(2)	7845(1)	26(1)
C15	1454(3)	5233(3)	7139(2)	32(1)
C16	2618(3)	4596(3)	6479(1)	29(1)
C17	4309(3)	4110(2)	6542(1)	22(1)
C18	5141(4)	3187(3)	5202(1)	39(1)
S1	6790(1)	784(1)	8539(1)	20(1)
F1	8947(3)	-1421(2)	9146(2)	41(1)
F2	9657(3)	344(2)	8987(2)	59(1)

F3	9862(3)	-1019(3)	8009(1)	76(1)
O7	7042(3)	1666(2)	7911(1)	32(1)
O8	6121(3)	-200(3)	8306(2)	30(1)
O9	6052(3)	1567(2)	9279(1)	46(1)
C19	8923(3)	-386(3)	8677(2)	28(1)
S1'	7623(12)	354(9)	8180(5)	33(2)
F1'	8530(30)	-1110(20)	9395(16)	37(5)
F2'	6210(20)	590(20)	9620(12)	49(4)
F3'	8470(30)	950(20)	9487(14)	56(5)
O7'	6840(30)	1941(17)	8181(15)	31(2)
O8'	9320(20)	-240(30)	7811(16)	46(5)
O9'	6560(30)	-330(30)	8100(20)	33(6)
C19'	7720(20)	210(20)	9225(10)	36(4)
S2	9135(3)	7224(2)	5293(1)	23(1)
F4	8623(6)	8802(4)	6581(2)	82(1)
F5	6492(4)	9382(4)	6000(3)	89(2)
F6	8500(8)	9951(6)	5515(3)	74(2)
O10	8510(9)	7539(8)	4563(3)	41(2)
O11	10899(5)	6901(4)	5243(2)	37(1)
O12	8566(6)	6286(4)	5762(2)	45(1)
C20	8154(6)	8935(4)	5875(2)	46(1)
S2'	8712(12)	7433(11)	5355(6)	31(1)
F4'	7540(20)	9396(17)	6427(7)	65(4)
F5'	6068(13)	9638(13)	5483(9)	59(3)
F6'	8370(20)	10030(20)	5302(14)	57(5)
O10'	8560(40)	7460(30)	4532(10)	25(5)
O11'	10418(18)	6990(20)	5466(11)	49(4)
O12'	7818(19)	6667(18)	5777(9)	34(3)
C20'	7574(18)	9289(16)	5654(9)	44(3)
O13	2110(20)	8220(20)	6548(11)	51(7)

Table S52. Bond lengths [Å] and angles [°] for jonap23.

Zn(1)-O(1)	2.2142(15)	C(4)-C(7)	1.526(3)
Zn(1)-O(6)	1.9691(15)	C(5)-H(5A)	0.9900
Zn(1)-N(1)	2.1634(15)	C(5)-H(5B)	0.9900
Zn(1)-N(2)	2.1137(16)	C(6)-H(6)	1.0000
Zn(1)-N(3)	2.1591(16)	C(6)-C(13)	1.526(3)
Zn(1)-O(7)	2.1756(18)	C(7)-C(8)	1.383(3)
Zn(1)-O(7')	2.210(16)	C(8)-H(8)	0.9500
O(1)-H(1)	0.81(4)	C(8)-C(9)	1.390(3)
O(1)-C(1)	1.429(2)	C(9)-H(9)	0.9500
O(2)-C(3)	1.431(2)	C(9)-C(10)	1.379(3)
O(2)-C(4)	1.419(2)	C(10)-H(10)	0.9500
O(3)-C(5)	1.435(2)	C(10)-C(11)	1.391(3)
O(3)-C(6)	1.414(2)	C(12)-H(12A)	0.9800
O(4)-C(11)	1.343(2)	C(12)-H(12B)	0.9800
O(4)-C(12)	1.437(2)	C(12)-H(12C)	0.9800
O(5)-C(17)	1.348(3)	C(13)-C(14)	1.382(3)
O(5)-C(18)	1.445(3)	C(14)-H(14)	0.9500
O(6)-H(6A)	0.85(4)	C(14)-C(15)	1.384(3)
O(6)-H(6B)	0.90(3)	C(15)-H(15)	0.9500
N(1)-C(2)	1.509(2)	C(15)-C(16)	1.374(4)
N(1)-C(4)	1.487(2)	C(16)-H(16)	0.9500
N(1)-C(6)	1.467(2)	C(16)-C(17)	1.391(3)
N(2)-C(7)	1.347(2)	C(18)-H(18A)	0.9800
N(2)-C(11)	1.344(2)	C(18)-H(18B)	0.9800
N(3)-C(13)	1.350(3)	C(18)-H(18C)	0.9800
N(3)-C(17)	1.346(3)	S(1)-O(7)	1.4489(19)
C(1)-H(1A)	0.9900	S(1)-O(8)	1.430(2)
C(1)-H(1B)	0.9900	S(1)-O(9)	1.423(2)
C(1)-C(2)	1.529(3)	S(1)-C(19)	1.829(2)
C(2)-C(3)	1.528(3)	F(1)-C(19)	1.317(3)
C(2)-C(5)	1.552(3)	F(2)-C(19)	1.318(3)
C(3)-H(3A)	0.9900	F(3)-C(19)	1.310(3)
C(3)-H(3B)	0.9900	S(1')-O(7')	1.475(15)
C(4)-H(4)	1.0000	S(1')-O(8')	1.415(14)

S(1')-O(9')	1.391(15)	N(3)-Zn(1)-N(1)	78.24(6)
S(1')-C(19')	1.818(16)	N(3)-Zn(1)-O(7)	84.81(7)
F(1')-C(19')	1.305(15)	N(3)-Zn(1)-O(7')	85.4(7)
F(2')-C(19')	1.305(14)	O(7)-Zn(1)-O(1)	170.09(7)
F(3')-C(19')	1.297(14)	O(7')-Zn(1)-O(1)	157.0(6)
S(2)-O(10)	1.432(4)	Zn(1)-O(1)-H(1)	123(3)
S(2)-O(11)	1.443(3)	C(1)-O(1)-Zn(1)	112.27(11)
S(2)-O(12)	1.436(3)	C(1)-O(1)-H(1)	110(2)
S(2)-C(20)	1.821(4)	C(4)-O(2)-C(3)	103.60(14)
F(4)-C(20)	1.332(5)	C(6)-O(3)-C(5)	105.45(14)
F(5)-C(20)	1.331(5)	C(11)-O(4)-C(12)	117.38(17)
F(6)-C(20)	1.314(5)	C(17)-O(5)-C(18)	117.58(18)
S(2')-O(10')	1.445(13)	Zn(1)-O(6)-H(6A)	116(2)
S(2')-O(11')	1.430(11)	Zn(1)-O(6)-H(6B)	118(2)
S(2')-O(12')	1.436(11)	H(6A)-O(6)-H(6B)	108(3)
S(2')-C(20')	1.776(14)	C(2)-N(1)-Zn(1)	115.14(11)
F(4')-C(20')	1.331(13)	C(4)-N(1)-Zn(1)	110.04(11)
F(5')-C(20')	1.309(13)	C(4)-N(1)-C(2)	105.28(14)
F(6')-C(20')	1.308(13)	C(6)-N(1)-Zn(1)	108.71(11)
O(13)-H(13A)	0.8499	C(6)-N(1)-C(2)	103.93(14)
O(13)-H(13B)	0.8501	C(6)-N(1)-C(4)	113.72(14)
O(6)-Zn(1)-O(1)	94.55(7)	C(7)-N(2)-Zn(1)	114.65(12)
O(6)-Zn(1)-N(1)	169.44(7)	C(11)-N(2)-Zn(1)	127.28(13)
O(6)-Zn(1)-N(2)	103.44(7)	C(11)-N(2)-C(7)	118.03(16)
O(6)-Zn(1)-N(3)	99.18(7)	C(13)-N(3)-Zn(1)	113.18(13)
O(6)-Zn(1)-O(7)	95.32(8)	C(17)-N(3)-Zn(1)	129.09(14)
O(6)-Zn(1)-O(7')	108.4(6)	C(17)-N(3)-C(13)	117.73(17)
N(1)-Zn(1)-O(1)	75.45(6)	O(1)-C(1)-H(1A)	110.1
N(1)-Zn(1)-O(7)	94.64(7)	O(1)-C(1)-H(1B)	110.1
N(1)-Zn(1)-O(7')	81.7(6)	O(1)-C(1)-C(2)	107.94(15)
N(2)-Zn(1)-O(1)	91.24(6)	H(1A)-C(1)-H(1B)	108.4
N(2)-Zn(1)-N(1)	80.51(6)	C(2)-C(1)-H(1A)	110.1
N(2)-Zn(1)-N(3)	156.65(6)	C(2)-C(1)-H(1B)	110.1
N(2)-Zn(1)-O(7)	87.38(7)	N(1)-C(2)-C(1)	111.40(15)
N(2)-Zn(1)-O(7')	82.0(7)	N(1)-C(2)-C(3)	102.11(14)
N(3)-Zn(1)-O(1)	92.70(6)	N(1)-C(2)-C(5)	103.73(14)

C(1)-C(2)-C(5)	112.52(16)	C(9)-C(10)-H(10)	121.0
C(3)-C(2)-C(1)	115.07(16)	C(9)-C(10)-C(11)	117.91(19)
C(3)-C(2)-C(5)	110.95(16)	C(11)-C(10)-H(10)	121.0
O(2)-C(3)-C(2)	103.12(15)	O(4)-C(11)-N(2)	112.69(17)
O(2)-C(3)-H(3A)	111.1	O(4)-C(11)-C(10)	124.28(18)
O(2)-C(3)-H(3B)	111.1	N(2)-C(11)-C(10)	123.03(18)
C(2)-C(3)-H(3A)	111.1	O(4)-C(12)-H(12A)	109.5
C(2)-C(3)-H(3B)	111.1	O(4)-C(12)-H(12B)	109.5
H(3A)-C(3)-H(3B)	109.1	O(4)-C(12)-H(12C)	109.5
O(2)-C(4)-N(1)	106.38(14)	H(12A)-C(12)-H(12B)	109.5
O(2)-C(4)-H(4)	109.6	H(12A)-C(12)-H(12C)	109.5
O(2)-C(4)-C(7)	109.12(15)	H(12B)-C(12)-H(12C)	109.5
N(1)-C(4)-H(4)	109.6	N(3)-C(13)-C(6)	117.72(16)
N(1)-C(4)-C(7)	112.37(14)	N(3)-C(13)-C(14)	122.90(19)
C(7)-C(4)-H(4)	109.6	C(14)-C(13)-C(6)	119.38(18)
O(3)-C(5)-C(2)	105.71(15)	C(13)-C(14)-H(14)	120.9
O(3)-C(5)-H(5A)	110.6	C(13)-C(14)-C(15)	118.2(2)
O(3)-C(5)-H(5B)	110.6	C(15)-C(14)-H(14)	120.9
C(2)-C(5)-H(5A)	110.6	C(14)-C(15)-H(15)	119.9
C(2)-C(5)-H(5B)	110.6	C(16)-C(15)-C(14)	120.1(2)
H(5A)-C(5)-H(5B)	108.7	C(16)-C(15)-H(15)	119.9
O(3)-C(6)-N(1)	106.47(15)	C(15)-C(16)-H(16)	120.9
O(3)-C(6)-H(6)	109.9	C(15)-C(16)-C(17)	118.2(2)
O(3)-C(6)-C(13)	110.67(15)	C(17)-C(16)-H(16)	120.9
N(1)-C(6)-H(6)	109.9	O(5)-C(17)-C(16)	123.98(19)
N(1)-C(6)-C(13)	109.99(15)	N(3)-C(17)-O(5)	113.19(18)
C(13)-C(6)-H(6)	109.9	N(3)-C(17)-C(16)	122.8(2)
N(2)-C(7)-C(4)	118.59(16)	O(5)-C(18)-H(18A)	109.5
N(2)-C(7)-C(8)	122.69(18)	O(5)-C(18)-H(18B)	109.5
C(8)-C(7)-C(4)	118.69(17)	O(5)-C(18)-H(18C)	109.5
C(7)-C(8)-H(8)	120.9	H(18A)-C(18)-H(18B)	109.5
C(7)-C(8)-C(9)	118.27(19)	H(18A)-C(18)-H(18C)	109.5
C(9)-C(8)-H(8)	120.9	H(18B)-C(18)-H(18C)	109.5
C(8)-C(9)-H(9)	120.0	O(7)-S(1)-C(19)	103.67(13)
C(10)-C(9)-C(8)	120.04(19)	O(8)-S(1)-O(7)	112.73(14)
C(10)-C(9)-H(9)	120.0	O(8)-S(1)-C(19)	103.82(14)

O(9)-S(1)-O(7)	113.68(14)	O(10)-S(2)-O(12)	114.9(3)
O(9)-S(1)-O(8)	117.06(15)	O(10)-S(2)-C(20)	104.4(4)
O(9)-S(1)-C(19)	103.91(13)	O(11)-S(2)-C(20)	102.4(2)
S(1)-O(7)-Zn(1)	153.52(14)	O(12)-S(2)-O(11)	114.8(2)
F(1)-C(19)-S(1)	111.98(18)	O(12)-S(2)-C(20)	103.3(2)
F(1)-C(19)-F(2)	107.2(2)	F(4)-C(20)-S(2)	111.1(3)
F(2)-C(19)-S(1)	111.55(18)	F(5)-C(20)-S(2)	110.6(3)
F(3)-C(19)-S(1)	111.52(19)	F(5)-C(20)-F(4)	106.4(4)
F(3)-C(19)-F(1)	106.6(2)	F(6)-C(20)-S(2)	111.6(3)
F(3)-C(19)-F(2)	107.7(2)	F(6)-C(20)-F(4)	109.8(4)
O(7')-S(1')-C(19')	97.8(13)	F(6)-C(20)-F(5)	107.1(4)
O(8')-S(1')-O(7')	112.8(12)	O(10')-S(2')-C(20')	100.4(12)
O(8')-S(1')-C(19')	104.6(14)	O(11')-S(2')-O(10')	111.9(13)
O(9')-S(1')-O(7')	115.2(16)	O(11')-S(2')-O(12')	115.6(11)
O(9')-S(1')-O(8')	121.2(16)	O(11')-S(2')-C(20')	109.5(12)
O(9')-S(1')-C(19')	100.6(17)	O(12')-S(2')-O(10')	111.3(13)
S(1')-O(7')-Zn(1)	134.2(14)	O(12')-S(2')-C(20')	106.9(9)
F(1')-C(19')-S(1')	111.8(17)	F(4')-C(20')-S(2')	106.3(11)
F(2')-C(19')-S(1')	109.8(15)	F(5')-C(20')-S(2')	106.1(10)
F(2')-C(19')-F(1')	104.9(17)	F(5')-C(20')-F(4')	111.3(13)
F(3')-C(19')-S(1')	113.4(16)	F(6')-C(20')-S(2')	109.1(14)
F(3')-C(19')-F(1')	105.7(18)	F(6')-C(20')-F(4')	109.7(15)
F(3')-C(19')-F(2')	110.9(18)	F(6')-C(20')-F(5')	114.0(14)
O(10)-S(2)-O(11)	114.7(3)	H(13A)-O(13)-H(13B)	109.4

Table S53. Anisotropic displacement parameters ($Å^2$x 10^3) for jonap23. The anisotropic displacement factor exponent takes the form: $-2\pi^2[h^2 a^{*2}U_{11} + ... + 2 h k a^* b^* U_{12}]$

	U_{11}	U_{22}	U_{33}	U_{23}	U_{13}	U_{12}
Zn1	15(1)	19(1)	12(1)	0(1)	-2(1)	-7(1)
O1	19(1)	24(1)	17(1)	6(1)	0(1)	-9(1)
O2	29(1)	18(1)	15(1)	-1(1)	-4(1)	-11(1)
O3	18(1)	20(1)	20(1)	1(1)	3(1)	-6(1)
O4	17(1)	28(1)	18(1)	-1(1)	0(1)	-1(1)
O5	29(1)	42(1)	19(1)	-3(1)	-9(1)	-14(1)
O6	22(1)	44(1)	16(1)	-4(1)	-2(1)	-9(1)
N1	15(1)	15(1)	12(1)	2(1)	-2(1)	-8(1)
N2	15(1)	16(1)	15(1)	2(1)	-3(1)	-7(1)
N3	18(1)	19(1)	18(1)	3(1)	-5(1)	-9(1)
C1	20(1)	19(1)	19(1)	5(1)	-3(1)	-8(1)
C2	20(1)	14(1)	17(1)	2(1)	-2(1)	-8(1)
C3	27(1)	18(1)	20(1)	3(1)	-5(1)	-12(1)
C4	19(1)	17(1)	10(1)	0(1)	-2(1)	-8(1)
C5	21(1)	17(1)	26(1)	1(1)	0(1)	-6(1)
C6	14(1)	18(1)	17(1)	2(1)	1(1)	-6(1)
C7	18(1)	18(1)	15(1)	2(1)	-4(1)	-10(1)
C8	23(1)	28(1)	19(1)	2(1)	-6(1)	-13(1)
C9	21(1)	35(1)	24(1)	6(1)	-10(1)	-13(1)
C10	16(1)	28(1)	26(1)	6(1)	-6(1)	-10(1)
C11	16(1)	19(1)	18(1)	2(1)	-2(1)	-8(1)
C12	21(1)	33(1)	27(1)	0(1)	2(1)	4(1)
C13	17(1)	19(1)	21(1)	4(1)	-4(1)	-10(1)
C14	17(1)	30(1)	32(1)	4(1)	-5(1)	-11(1)
C15	20(1)	35(1)	42(1)	6(1)	-14(1)	-12(1)
C16	31(1)	31(1)	32(1)	5(1)	-17(1)	-16(1)
C17	26(1)	23(1)	22(1)	3(1)	-9(1)	-13(1)
C18	47(2)	55(2)	22(1)	-3(1)	-13(1)	-26(1)
S1	17(1)	16(1)	26(1)	1(1)	-5(1)	-6(1)
F1	42(1)	32(1)	60(2)	27(1)	-29(1)	-22(1)
F2	54(1)	45(1)	110(2)	40(1)	-58(1)	-37(1)

F3	33(1)	99(2)	49(1)	6(1)	3(1)	18(1)
O7	37(1)	29(1)	42(1)	19(1)	-22(1)	-21(1)
O8	29(1)	24(1)	48(2)	11(1)	-19(1)	-16(1)
O9	50(1)	37(1)	38(1)	-13(1)	0(1)	-5(1)
C19	23(1)	29(1)	38(1)	12(1)	-10(1)	-13(1)
S1'	41(5)	31(4)	34(4)	7(3)	-10(3)	-21(3)
F1'	59(11)	40(8)	24(10)	15(6)	-12(8)	-30(7)
F2'	63(8)	50(10)	37(8)	-6(7)	3(6)	-29(7)
F3'	70(11)	59(10)	59(10)	10(8)	-21(8)	-41(9)
O7'	37(2)	28(2)	42(3)	17(2)	-22(2)	-22(2)
O8'	43(8)	46(10)	46(10)	14(8)	3(6)	-18(6)
O9'	48(12)	34(10)	22(11)	5(8)	-8(8)	-22(10)
C19'	54(8)	35(8)	27(7)	13(5)	-10(5)	-25(6)
S2	28(1)	26(1)	14(1)	0(1)	2(1)	-12(1)
F4	118(3)	90(2)	35(1)	-29(1)	0(2)	-42(2)
F5	45(2)	81(2)	104(4)	-17(2)	37(2)	-4(2)
F6	103(3)	38(2)	78(3)	-15(2)	22(2)	-38(2)
O10	42(3)	74(4)	23(2)	15(2)	-12(2)	-40(3)
O11	29(2)	46(2)	32(2)	6(1)	-7(1)	-10(1)
O12	68(3)	47(2)	24(1)	6(1)	8(2)	-32(2)
C20	49(2)	39(2)	41(2)	-7(2)	12(2)	-14(2)
S2'	35(2)	35(2)	16(2)	3(1)	-1(2)	-8(2)
F4'	64(8)	83(8)	44(5)	-24(4)	8(5)	-30(6)
F5'	37(4)	56(6)	69(8)	-2(5)	6(4)	-8(4)
F6'	31(5)	34(6)	85(11)	-2(6)	10(6)	1(4)
O10'	38(11)	16(7)	21(8)	6(4)	-5(5)	-10(6)
O11'	21(6)	77(8)	44(9)	16(7)	-9(5)	-12(5)
O12'	39(6)	49(7)	21(5)	15(5)	-6(5)	-22(6)
C20'	35(6)	38(6)	48(6)	-12(4)	7(4)	-6(4)
O13	49(12)	75(15)	39(11)	-19(9)	6(8)	-41(11)

Table S54. Hydrogen coordinates (x 10^4) and isotropic displacement parameters (Å^2 x 10^3) for jonap23.

	x	y	z	U(eq)
H1	7760(40)	5850(40)	6740(20)	52(10)
H6A	9580(50)	2590(40)	6289(19)	44
H6B	8060(40)	2860(30)	6000(20)	44
H1A	5016	6952	7265	23
H1B	6141	7751	7509	23
H3A	7840	6521	8663	24
H3B	6209	7803	9145	24
H4	6423	3966	9512	19
H5A	3523	8065	9155	27
H5B	3032	7766	8330	27
H6	4331	4071	8943	20
H8	9429	3906	9948	27
H9	12305	2497	9547	30
H10	13262	1427	8265	28
H12A	13166	547	6332	49
H12B	13408	0	7195	49
H12C	13753	1384	6930	49
H14	1179	5793	8305	31
H15	290	5589	7111	38
H16	2278	4491	5994	35
H18A	6162	2757	4811	58
H18B	4369	4080	5013	58
H18C	4601	2508	5284	58
H13A	1712	7873	6220	76
H13B	1440	8449	6983	76

Table S55. Torsion angles [°] for jonap23.

Zn1-O1-C1-C2	46.71(17)	C2-N1-C4-C7	-103.55(16)
Zn1-N1-C2-C1	12.98(19)	C2-N1-C6-O3	-34.16(18)
Zn1-N1-C2-C3	-110.36(13)	C2-N1-C6-C13	85.79(17)
Zn1-N1-C2-C5	134.25(13)	C3-O2-C4-N1	-38.19(18)
Zn1-N1-C4-O2	140.43(11)	C3-O2-C4-C7	83.26(17)
Zn1-N1-C4-C7	21.09(17)	C3-C2-C5-O3	-101.52(18)
Zn1-N1-C6-O3	-157.27(11)	C4-O2-C3-C2	44.91(18)
Zn1-N1-C6-C13	-37.32(17)	C4-N1-C2-C1	134.35(16)
Zn1-N2-C7-C4	1.1(2)	C4-N1-C2-C3	11.02(17)
Zn1-N2-C7-C8	179.09(15)	C4-N1-C2-C5	-104.37(16)
Zn1-N2-C11-O4	1.8(2)	C4-N1-C6-O3	79.77(18)
Zn1-N2-C11-C10	-177.98(14)	C4-N1-C6-C13	-160.28(15)
Zn1-N3-C13-C6	-1.7(2)	C4-C7-C8-C9	177.38(18)
Zn1-N3-C13-C14	179.26(16)	C5-O3-C6-N1	39.90(19)
Zn1-N3-C17-O5	-1.0(3)	C5-O3-C6-C13	-79.60(18)
Zn1-N3-C17-C16	179.97(16)	C5-C2-C3-O2	76.11(18)
O1-C1-C2-N1	-38.4(2)	C6-O3-C5-C2	-28.6(2)
O1-C1-C2-C3	77.2(2)	C6-N1-C2-C1	-105.81(17)
O1-C1-C2-C5	-154.38(16)	C6-N1-C2-C3	130.85(15)
O2-C4-C7-N2	-133.18(16)	C6-N1-C2-C5	15.46(18)
O2-C4-C7-C8	48.7(2)	C6-N1-C4-O2	-97.34(17)
O3-C6-C13-N3	144.19(16)	C6-N1-C4-C7	143.32(15)
O3-C6-C13-C14	-36.7(2)	C6-C13-C14-C15	-178.33(19)
N1-C2-C3-O2	-33.89(17)	C7-N2-C11-O4	179.54(16)
N1-C2-C5-O3	7.43(19)	C7-N2-C11-C10	-0.2(3)
N1-C4-C7-N2	-15.5(2)	C7-C8-C9-C10	-0.6(3)
N1-C4-C7-C8	166.46(17)	C8-C9-C10-C11	1.4(3)
N1-C6-C13-N3	26.8(2)	C9-C10-C11-O4	179.30(19)
N1-C6-C13-C14	-154.11(18)	C9-C10-C11-N2	-1.0(3)
N2-C7-C8-C9	-0.6(3)	C11-N2-C7-C4	-176.98(16)
N3-C13-C14-C15	0.7(3)	C11-N2-C7-C8	1.0(3)
C1-C2-C3-O2	-154.71(15)	C12-O4-C11-N2	-178.61(18)
C1-C2-C5-O3	127.95(17)	C12-O4-C11-C10	1.1(3)
C2-N1-C4-O2	15.79(17)	C13-N3-C17-O5	179.14(17)

C13-N3-C17-C16	0.2(3)	O8'-S1'-C19'-F2'	-173.3(16)
C13-C14-C15-C16	0.3(3)	O8'-S1'-C19'-F3'	62(2)
C14-C15-C16-C17	-1.0(3)	O9'-S1'-O7'-Zn1	-122(3)
C15-C16-C17-O5	-178.1(2)	O9'-S1'-C19'-F1'	69(2)
C15-C16-C17-N3	0.8(3)	O9'-S1'-C19'-F2'	-47(2)
C17-N3-C13-C6	178.13(17)	O9'-S1'-C19'-F3'	-172(2)
C17-N3-C13-C14	-0.9(3)	C19'-S1'-O7'-Zn1	132(2)
C18-O5-C17-N3	173.3(2)	O10-S2-C20-F4	179.8(4)
C18-O5-C17-C16	-7.7(3)	O10-S2-C20-F5	61.8(4)
O7-S1-C19-F1	-170.2(2)	O10-S2-C20-F6	-57.3(5)
O7-S1-C19-F2	69.7(2)	O11-S2-C20-F4	-60.3(3)
O7-S1-C19-F3	-50.8(2)	O11-S2-C20-F5	-178.2(3)
O8-S1-O7-Zn1	159.6(3)	O11-S2-C20-F6	62.6(5)
O8-S1-C19-F1	-52.2(2)	O12-S2-C20-F4	59.3(4)
O8-S1-C19-F2	-172.4(2)	O12-S2-C20-F5	-58.7(4)
O8-S1-C19-F3	67.2(2)	O12-S2-C20-F6	-177.8(4)
O9-S1-O7-Zn1	23.3(4)	O10'-S2'-C20'-F4'	176.2(15)
O9-S1-C19-F1	70.8(2)	O10'-S2'-C20'-F5'	57.6(16)
O9-S1-C19-F2	-49.4(2)	O10'-S2'-C20'-F6'	-65.7(18)
O9-S1-C19-F3	-169.9(2)	O11'-S2'-C20'-F4'	-66.0(13)
C19-S1-O7-Zn1	-88.8(3)	O11'-S2'-C20'-F5'	175.5(11)
O7'-S1'-C19'-F1'	-173.4(19)	O11'-S2'-C20'-F6'	52.2(16)
O7'-S1'-C19'-F2'	70.6(17)	O12'-S2'-C20'-F4'	59.9(13)
O7'-S1'-C19'-F3'	-54.0(19)	O12'-S2'-C20'-F5'	-58.6(13)
O8'-S1'-O7'-Zn1	23(3)	O12'-S2'-C20'-F6'	178.1(15)
O8'-S1'-C19'-F1'	-57(2)		

Table S56. Hydrogen bonds and close contacts for jonap23 [Å and °].

D-H...A	d(D-H)	d(H...A)	d(D...A)	<(DHA)
O1-H1...O12	0.81(4)	1.85(4)	2.659(3)	177(4)
O1-H1...O12'	0.81(4)	1.85(4)	2.639(13)	163(4)
O6-H6A...O10#1	0.85(4)	2.02(4)	2.783(7)	149(3)
O6-H6A...O10'#1	0.85(4)	1.98(4)	2.75(3)	150(3)
O6-H6B...O5	0.90(3)	2.05(3)	2.717(2)	130(3)
O6-H6B...O11#1	0.90(3)	2.25(3)	2.900(4)	129(3)
O13-H13B...F3#2	0.85	2.01	2.856(18)	179.8
O13-H13B...O8'#2	0.85	2.13	2.93(3)	156.7

Symmetry transformations used to generate equivalent atoms:

#1 -x+2,-y+1,-z+1   #2 x-1,y+1,z
